# Disparities in pathways for anaemia care and recovery among women after major abdominal surgery: secondary analysis of a prospective cohort study in 29 countries

**DOI:** 10.1093/bjsopen/zrag125

**Published:** 2026-09-29

**Authors:** Sivesh Kathir Kamarajah, Dharani Hapangama, STARSurg Collaborative, Dania Badran, Omar Kouli, Ieva Jakaitytė, Setthasorn Ooi Zhi Yang, Aneel Bhangu, Kariem El-Boghdadly, James Glasbey, Ana Minaya Bravo, Gregory Lip, Shakila Thangaratinam, Dharani Hangapama, Sivesh K Kamarajah, Omar Kouli, Sivesh Kamarajah, S K Kamarajah, R Blanco-Colino, N Brindl, S Brown, W A Cambridge, D Chaudhry, K Gressmann, R R Gujjuri, A Hilder, A Jaffer, I Jakaityte, M Kawka, O Kouli, E Matey, K A McLean, A Mergo, E C Mills, V Murray, S Z Y Ooi, I S Pereira, A M Riad, A Sgrò, S Q Shafi, I Trout, A Turňa, C Varghese, W Xu, S K Kamarajah, B M Biccard, A Docherty, J Martin, K El-Boghdadly, M Phull, R Mouton, A Bhangu, J C Glasbey, E M Harrison, K A McLean, N Smart, S Moug, T Pinkney, T Richards, S K Kamarajah, I Dajti, M de Cillia, G Van Ramshorst, S Delibegovic, H Mughal, J Mihanovic, N Gouvas, P Kocian, V Levesen, J Kaupilla, N Brindl, D Merz, M Joos, A Ioannidis, G Nnaji, F Pata, G Pellino, A Gori, M Podda, C Riboni, E Fekaj, S Oliver Senica, A Dauksa, J Psaila, F de Ruitjer, P Major, I Santos, M Sampaio Alves, J Simões, E A Bonci, A Pașca, A Novikova, B Kovačević, V Milosavljevic, B Tadic, J Kosir, R Blanco-Colino, S Pérez Ajates, M E Ossola Revilla, A Papadia, C Riboni, M L Gasparri, M K Aktas, B E Baki, M D Tepe, A U Mutlu, S K Kamarajah, A Singal, J Osei-Bonsu, H Lacey, S W Chan, M Allison, K Duah-Asante, D Chen, N Ahmed, A Ejiz, C Takyi, M Mujeeb, N Ravikumar, M Khan, J Hayes, J Mckenna, J Wang, N Essa, H Xianghan, L Ko, Y Aldabbagh, J Plascevic, N Zia, R Ismail, Y Kamel, I Epanomeritakis, R Tan, N Chiu, A Naeem, M Kakwani, R Mehra, K Feeney, C L Yan Naing, A Qureshi, A Richens, H Li, R Ahmed, L Wilson, S Abraha, I Dajti, S Mikalauskas, J Kahn, D Kniepeiss, M Kušar, A Belarmino, J Frenk, I Mikalauskiene, A Angelis, J E Waha, S Al-Sharafy, M Sandano, P Schemmer, C Reiterer, K Horvath, A Taschner, S Riss, F Harpain, C Dawoud, N Hantáková, N Adamowitsch, S Schallmeiner, T Christian, V Xu, M M Kuhrn, K M Widmann, B Capek, B Kama, S Turgut, L Hauptmann, M de Cillia, M Grünbart, H Hoi, A Binder, T Gürtler, P Riedl, D Mayer, K Van Belle, O Wautelet, E Dekkers, E Van Daele, T Apers, G Van Ramshorst, F Berrevoet, N Rennie, P Dries, P Panta, A Denys, W Pype, T Bauwens, N Renard, S Violon, J Stijns, T Van De Winkel, E Van Eetvelde, M Salibasic, S Delibegovic, S Pusina, E Hodžić, M Kruščica, S Žilić, E Bicakcic, A Rovcanin, E Kulovic, E Halilovic, A Vinčević Hodžić, E Kalbić, S Delibegovic, A Mujić, M Mešan, E Matović, S Delibegovic, A Kesetovic, D Dardanov, E Arabadzhieva, B Markov, К Спасова, C Biji, N Oliveira, V Miroslavov, G Korukov, K Ravendran, N Shah, Y Mitkov, K Arivanandan, R Gaikwad, S Gotru, R Saraff, N Sain, D Stanchev, L Gaydarski, U Ali, L J Kandathil, A Krishnaveni, I Ivanov, I Ganev, Т Krapeshki, M Miza, M S K Mumu, I Bashliev, A Sajjad, L Chandel, D Yordanov, E Hristova, K Spassov, T Tsankov, T Ivanov, E Filipov, D Georgiev, V Neykov, E Daleva, I Ilieva, D Krasimirova, H Hristov, М Арсова, O Soladoye, A Biswakarma, R Raj, A Negi, A Dey, J K Adidela, U G Alionye, A Ndukwe, R Dimov, V Ivanov, S Sulcheva, S Mitrev, V Nikolov, M Karamanliev, D Dimitrov, A Yordanov, P Vladova, M Shoshkova, K Karakadieva, A Gabarski, A Shanker, H Ehtisham, S Parambi, L Tranchev, A Vasileva, J Kareem, J Ezeabasili, O L Madueke-Ediae, T Muradia, A Saini, I Rana, R Kanagaratnam, L S Thomas, M Galasyuk, J J Costa, N Majid, P Reddy, M Abdullahi, S Koshy Thomas, A Mehta, A Biswakarma, R Raj, S Shittu, J Theophilus, J Theophilus, R Ilyas-Uddin, I Ogunleye, E Naghavi, A Kiran, A Sundar, A Joy, F Ali, I Angelova, M Slavchev, N Belev, B Atanasov, P Krastev, T Yotsov, D Dimitrov, P Kamenova, I Mihaylov, A Stavrov, A Vricheva, G Šantak, L Penezić, Z Kastelan, Z Zimak, H Saić, N Knezevic, B Cikic, T Zekulić, T Hudolin, I Juric, J Anđelić, T Kulis, M Maric, J Mihanovic, T Soric, D Surić Grčić, L Blagus, P Kovačević, I Vidić, A Miočić Juran, C Ergatidou, S Gravas, A Yiallourou, S Achilleos, M Vardas, G Kokkinos, C Panagiotou, M Kyprianou, K Kyriacou, P Papatheodorou, K Konstantinou, D Onisiforou, F Tsoutsouki, A Kasapi, E Demetriou, A Stylianou, E Xenophontos, P Georgiou, P Makrides, I Pozotou, M Constantinou, T Evangelou, A Varavina, S Charitonos, M Lampi, M Attaalla, T Dušek, M Ndukwe, L Kuzmane, E Moorlata, H Sillah, J Kotek, J Fazal, E Ssali, V Steingauer, K Vinklerova, O Ahmad, Martínez Á Rodríguez, E Hiebert, M Srivastava, A S Ramesh Babu, O Dobra, A Akiba, M F Kalou, T Sokolová, N Nepeřená, P Novotný, J Sedlackova, F Philips, H Al Atassi, P Jose, A Sunil Nair, L Ntashamaje, A Kaddah, M A Antabi, P Kocián, R Fiala, T Harustiak, S Vesely, A Haluza, A Štekrtová, V Král, K Zdichová, J Pastor, A Gorchakov, H Novák, V Novák, J Hornak, M Havova, M Marková, T Fořtová, D Lérias Bento, R Novysedlák, J F Smetana, T Novák, T Zdobinska, M Švarc, E Hejduková, K Havlová, A Vaishevich, J Čorňáková, P Zajíc, P Francúz, O Příman, Š Havlíková, B Zemlickova, A Whitley, R Gurlich, P Balaz, I Tomyak, M Kútik, O Molva, D Šturc, M Belbl, T Vinklarkova, V Villefranque, A Police, E Mikhael, A Mabilia, L Charre, E Volpin, H Braham, R Montero Macías, D Krief, A Boyer De Latour, M A Angeles, A Martinez, A Navarro, G Ferron, M Del, M Ghiani, F Migliorelli, M Danguy Des Déserts, S Johan, B Olivier, C Andro, D Von Wedel, C Kamphues, M Thiele, J Felber, J Neudecker, C Schineis, J C Lauscher, R Stephan, L P Meyer, C Wolff, B Bombera, J Rolinger, A Kirschniak, P Wilhelm, S Göller, L Van Den Hil, D Reim, M Kießler, C Buschhaus, S Naisar, N Zahlmann, R L Walter, M Weber, K Müller, A Schweden, M Berlet, N A Jorek, S Seyfried, N Rahbari, E Birgin, C Reissfelder, A Reeg, P Téoule, E Rasbach, N Pyrgidis, G Hatzichristodoulou, I Sokolakis, J J Strotmann, T Fahlbusch, P Höhn, J Hinrichs, J Horn, S Jollet, J Binder, T Förtsch, M Winterstein, D Hackner, A Girard, M Wittmann, T O Vilz, F Recker, M Velten, G Massoth, A Delis, M I Brugués Villalba, M Willis, S Soltau, S Kudaliyanage, R L Eymael, I Syring, R Neubauer, N Dahmen, A Zimmermann, N Straßberger-Nerschbach, A Puskarevic, X Wang, L Peyman, R A Philippi, K Schmidt, U Bork, F Schepp, S Korn, O Radulova-Mauersberger, J Von Dem Bussche, F Von Bechtolsheim, C Praetorius, N Knobloch, F Oehme, J Weitz, M Mansfeld, M Distler, F Klingler, G Fluegen, S Dávid, M Schneider, K A Baba, U Ronellenfitsch, J Kleeff, J Ukkat, J Klose, O Bayram, M Sommerer, A Rebelo, I Gockel, E Gentsch, N Rayes, R Thieme, N Kreuser, B Aktas, D Branzan, D Seehofer, M R Mallmann, C Mallmann, S Woldu, F Balci, C Domröse, M Diekhöner, G Micha, K Stroumpoulis, L Dritsoulas, K Kalopita, G Bitzi, P Thessalonikefs, E Fradelos, D Korkolis, A Sarafi, D K Manatakis, N Tasis, A Prountzopoulou, E Tziava, K Pikoulas, D Paramythiotis, M Tishukov, K Papadopoulos, G Chatziantoniou, G Tzikos, S Papamichail, S Bareka, A Sarafis, D Haidopoulos, K Angelou, A Prodromidou, E Stamatakis, N Alexakis, V Pergialiotis, A Rodolakis, N Thomakos, N Memos, N Vlahos, I Koutalas, T Kotsis, E Kalampokas, L Chardalias, C Kontopoulou, C Lignou, P Lykourgioti, A Bagiasta, A Petrolekas, O Savranakis, T Kozonis, V Themelidi, M Mccormac-Prekeze, C Nikolaou, E Bletsa, N Melissaridou, K Flamourakis, P Rammos, T Hadjizacharias, N Provata, D Politis, A P Gkioulekas, D Massaras, I Vakos, A Antonoglou, P Dimitra, A Kotzadimitriou, C Evangelou, D Psychogios, M K Konstantinidis, K Apostolou, K Konstantinidis, N Patelis, S Konstantinidou, P Kokoropoulos, N Michalopoulos, Z Kratiras, P Drakakis, T Sidiropoulos, M Papadoliopoulou, P Vassiliu, N Arkadopoulos, C Koratzanis, N Vrachnis, E Dylja, I Chatzialis, D Sampanis, N Danias, A Fotiou, S Stavros, Z Petropoulou, V Tsaousis, D Papakonstantinou, P Lykoudis, C Nastos, A Charalampopoulos, I E Papiri, I Hatzaras, K I Paraskevas, G Petrakis, E Polenta, E Kaparounaki, M Sotiropoulou, S Kapiris, E Mavrodimitraki, A Paraskeva, A Kolinioti, M Psarologos, D Stergiou, P Metaxas, K Stamatis, C Kyzeridis, E Kefalou, G Z Vrakopoulou, A Larentzakis, E Menenakos, V R Maravgaki, C Georgiadou, M Koutrouli, A Papageorgiou, A Skandali, G Tzovaras, I Baloyiannis, V Tzortzis, G Christodoulidis, K Perivoliotis, E Arnaoutoglou, M P Ntalouka, A Chatzis, A Daponte, A Samara, C Donoudis, D Zacharoulis, F Mulita, K Bouchagier, G Verras, L Tchabashvili, O Ioannidis, A Koltsida, A Malliora, D Paparouni, S Bitsianis, L Loutzidou, E Anestiadou, Χ Χατζηανεστιάδου, S Simeonidis, A Tekelidis, K Zapsalis, S Skalidou, N Ouzounidis, G Ntampakis, A Kelepouri, F Kontidis, V Foutsitzis, O Kontaxi, G Barakakis, O M Valaroutsou, C Athanasiou, E Kotidis, M Pramateftakis, I Mantzoros, K Toutouzas, M Frountzas, T Triantafyllou, A Triantafyllou, T Dagklis, G Katsanos, S Petousis, A Athanasiadis, G Tsoulfas, K Dinas, I Tsakiridis, A Mamopoulos, I Kalogiannidis, F Rao, C Christou, S Vasileiadou, G Kapetanios, N Tsakiridis, S Kopatsaris, E Papanikolaou, K E Karakasi, T A Tataridou, N Antoniadis, F Zachomitros, A Arvanitaki, K Tsakiridis, M Anemoulis, S Neiros, K Ouranos, E C Tampaki, C Maltezos, K Maltezos, C Anastasiadou, A Chaveles, A Pachi, P Tsiantoula, K Roditis, A Antoniou, N Bessias, T Papas, S Tzamtzidou, D Maras, V Papaioannou, G Koukoulis, K Bouliaris, K Skriapas, G Kontopoulos, C Doudakmanis, C Kolla, M Efthimiou, C Kalfountzos, D Mitsakou, C Kardasi, S Zourntou, L I Fountarlis, A Bakalis, M Samarinas, P L Chatzilamprou, A Migdanis, K Karvouni, K Katsiafliaka, C Arvaniti, A R Papazisi, V Markatou, K Zervas, K Marsitopoulos, N Machairas, P Dorovinis, E Kotsifa, M D Keramida, D Schizas, M Vailas, A Syllaios, E Mela, N Hasemaki, A Skotsimara, A Katsargyris, S Kykalos, N Tomara, I Palios, I Karniadakis, A Charalabopoulos, P Sakarellos, S Davakis, N Kydonakis, G Tsourouflis, P Stamopoulos, K Laios, A Kozadinos, I Katsaros, E Kontis, C Iavazzo, L Katsiaras, E Kaouras, P Manikis, K Kokkali, G Theodorou, A Dragi, G Vorgias, L Tzelves, A Skolarikos, I Manolitsis, M Spartalis, I Tzima, A Anastasiou, P Pantelidis, G Schismenou, E Spartalis, G E Zakynthinos, K Lasithiotakis, G Petra, M Lampou, D Toth, Z Varga, J Pósán, L Illésy, C Váradi, M Santarelli, L Puca, L Brignone, R Lisa Marie, L Silvia, P Silvia, A Ferguglia, C Piceni, M Improta, F Assanti, E Montanari, G Ettore, F Cannone, L Cormaci, V Nicastro, R De Carlis, G Ferrari, A Giani, S Grimaldi, L Gregorio, M Mazzola, L Ripamonti, L Lorusso, G A Tartufari, C Magistro, A Benedetti, A Gonta, J Maesano, C L Bertoglio, I Giusti, O Quagli, F Brucchi, E Bevilacqua, L De Carlis, A Lauterio, R Cerchione, M Migliorini, N Incarbone, L Centonze, S S Darwish, I Vella, V Buscemi, A Podestà, M Desio, M Berselli, S Megna, E Cocozza, L Livraghi, V Quintodei, V Marchionini, G Borroni, C Peverelli, M Corbella, F Ferrari, F Odicino, E Gozzini, F Cisotto, G Baronio, R Del Giudice, G Sala, A Iacomino, G Vennarecci, D Ferraro, Martino M Di, D Pisaniello, F Falaschi, L Petagna, A Giuliani, P Di Lascio, M Coluzzi, G Pascale, P Gallicchio, G Frezza, G Dinatale, G Cerino, A Bottari, J Martellucci, G Maltinti, M Scheiterle, L Fortuna, F Staderini, F Coratti, M Veroux, A Giaquinta, G Roscitano, A Volpicelli, P Veroux, M Palumbo, G Riccioli, L Stella, C Distefano, R Gioco, G Lomeo, E Lomeo, M Cavallo, C Virgilio, C Molino, M M Giambra, D Zerbo, R Santini, S Costa, G G Incognito, D C Centonze, G Currò, M Ammendola, R Filippo, G Ammerata, R Balestri, L Morelli, D Tartaglia, M Puccini, V F Asta, V Conte, P Buccianti, B Sargenti, G Boni, S Signori, D Pezzati, L Urbani, G Casale, G Taddei, G Di Franco, M Bianchini, C Carpenito, N Furbetta, A Comandatore, F Tarasco, S Guadagni, M Mastrangelo, C Gianfaldoni, M Palmeri, L M Fatucchi, A B Boato, D Gianardi, M Stingone, L Sacco, S Giaquinto, L Lami, M Iuliano, E A Annunziata, M Chiarugi, S Strambi, F Coccolini, G Anania, M Chiozza, A Campagnaro, V Nevoso, L Carbone, L Marano, G Micheletti, A Fontani, S Malerba, M Gambelli, A Bombino, G Grassi, V D Mandato, L Aguzzoli, V Mastrofilippo, N Fabbri, C V Feo, M Ginestri, D Oppici, M Torchiaro, A Pesce, F Catena, C Vallicelli, V Murzi, M Podda, A Pisanu, F Campus, T Pilia, A Saba, C Piras, M Pisano, E Locci, P Marongiu, E Gessa, Mario D’Oria, A Biloslavo, S Lepidi, P Germani, C J Nappi, B Grando, A Lauretta, S Pollesel, P P Brollo, G Gallo, M Trompetto, A Realis Luc, V Tiesi, A Porcu, T Perra, M Madonia, A Tedde, F Scognamillo, P L Tilocca, D Delogu, G Mucci, G Drocchi, M Tedde, A M Scanu, G Rizzo, A Fancellu, C F Feo, G Farina, R Casu, M L Cossu, G C Ginesu, M Anania, S Dessole, G Capobianco, M Petrillo, F Dessole, I Angelone, A Barberis, A Azzinnaro, A Petrungaro, E Palli, E Mina, N S Pipitone Federico, A Muratore, M Calabrò, M De Zuanni, E Herranz Van Nood, L Licari, C Callari, D Di Miceli, S Viola, M Manigrasso, C Caricato, M Milone, P Anoldo, S Vertaldi, A D’amore, G D De Palma, A Marello, C G Cantore, A Chini, F Maione, G Luglio, G Pagano, F P Tropeano, M Cricrì, G Aprea, G Palomba, M Capuano, R Basile, U Bracale, R Peltrini, M Visconti, G Magno, A G Di Santo Albini, P Fransvea, G Sganga, G Tropeano, C Puccioni, G Altieri, V Fico, P Mirco, V Bianchi, V Cozza, G Pepe, S Alfieri, F Rosa, C A Schena, V Laterza, M M Pascale, S Agnes, G Bianco, F Frongillo, F Ferracci, F Santullo, A Balla, P Lepiane, F Saraceno, R Mastroianni, G Simone, G Tuderti, F Marino, G Lantone, G Lantone, R Isernia, F Pezzolla, F Aquilino, D Raimondo, A Gori, R Seracchioli, M Rottoli, A Belvedere, L Maurino, I S Russo, B Orsini, M Maletta, A Romano, P Bernante, T Violante, D Morezzi, E Degli Esposti, A Raffone, G Poggioli, A Canavese, G Dajti, S Cardelli, P Casadio, A Arena, G Sanna, D Cuicchi, L F Angelicchio, C Catalioto, B Torre, P De Iaco, M Tesei, E De Crescenzo, C Larotonda, F Greco, M Minghetti, L Sissa, A M Perrone, G Dondi, M Di Stanislao, L Gaetani, L Serafini, E Prosperi, D Perone, C Isopi, F Bruno, I D Alexa, P Milito, A Lovece, G Chiappini, C Froiio, T Panici Tonucci, G Saletta, A Scardino, A Belli, F Izzo, R Patrone, C Cutolo, R Palaia, V Granata, V Albino, M Leongito, M Piccirillo, G Pasta, P Delrio, D Rega, M Rho, A Aversano, I A Angelone, M D’amico, M Di Marzo, S De Franciscis, P Cianci, E Restini, I Conversano, G Scialandrone, S Di Saverio, L Cardinali, G Travaglini, E Sebastiani, I Marziali, A B Bellocchia, I Raimondo, D Di Giorgio, G Garganese, V Tondolo, A Dore, G Pacini, L C Turco, P Campennì, M A Amara, A Rubattu, D Verri, G Sole, S Bove, F Inzaina, F Franceschi, G Grande, D P Pili, F Campus, G Delogu, V Sula, O Ez Zinabi, M Congiu, E Pira, G Ercolani, F D’acapito, D Annunziata, L Solaini, A Giordano, S Cantafio, S Novello, M Romano, C Armellin, G Zanus, F Milardi, U Grossi, R Baldan, M Brizzolari, F Scolari, A Brun-Peressut, A Broglia, M Martorana, N Fazzini, C Marafante, M Garino, E Moggia, S C Agosti, A Battaglia, A Borello, S L Birolo, R Barone, C Mosca, K Shakhova, A L Apostu, S Giaccari, M Pavanello, E Migliori, D Sambucci, C Corbellini, G M Sampietro, G Germiniasi, M Bischeri, F Colombo, P Danelli, F Cammarata, R S Zingale, I Pezzoli, M Carbonaro, F Albanesi, F Cozzani, M Rossini, F De Gennaro, M Giuffrida, P Del Rio, M Inama, G Moretto, L Iudici, A Vitali, M Creciun, H Impellizzeri, M Piazza, A Biancafarina, M De Prizio, R Malatesti, R Sulce, G A Pellicano’, A D’ignazio, V Mariottini, G Mura, M Angelini, M Scricciolo, F Barbara, F Tofani, L Nenci, V Borgogni, C Vece, A Sagnotta, L Solinas, M Rossi, S Mancini, R Fruscio, C Ciulli, M Braga, F Romano, M Garancini, F Carissimi, E Vico, C D G Delle Grazie, S Negri, J Corti, N Tamini, L Cigagna, R Giordano, A Davolio, G Trezzi, T Grassi, C Procaccianti, L C Nespoli, L Pitoni, A Fogliati, G Cordaro, P Passoni, M Fantauzzi, S Villa, M A Scotti, G M Di Lucca, F Benedetti, P Masseria, M Frigerio, M Barba, I Re, M Ceresoli, F Ferraina, S K Adjei Antwi, C Fumagalli, G Di Martino, M L Di Meo, L Bazzano, T Nelli, F Masciello, G Canonico, E Chisci, E Adinolfi, R Borreca, A Damigella, C Di Martino, G I L Mottola, G Fontani, S Michelagnoli, M Fedi, F Leo, R De Vincenti, C Cecchi, L Piombetti, S Giannessi, B Benedetta, M Pagani, L Epis, A Percivale, M Malerba, V Tonini, M Cervellera, J Shahu, L Sartarelli, A Luzzi, E Romairone, S Carrabetta, F Tuminello, F Floris, F Ré, S Marzorati, F Ballari, C Meola, A Filippelli, U G Ribeca, A Viacava, V Lizzi, M Montagna, A Giuliani, N Tartaglia, F Vovola, F Maffei, A Cotoia, M Pacilli, G Pavone, G Berardi, N Depalma, M Maruccio, M G Spampinato, S D’ugo, T Marchese, F Basurto, C De Giorgi, S Garritano, F Manoochehri, F Perrone, I Botrugno, A Rizzi, W Sergi, G I Russo, A Cappellani, S Cimino, M G Asmundo, M G Matarazzo, M Di Vita, P Venturelli, G Cocorullo, F Zarcone, D M Dominici, G Salamone, R Tutino, A Corigliano, R Guercio, C Giuseppe, F D’arpa, G Melfa, M P Proclamà, G Scerrino, I Canfora, M Marcianò, G Guercio, S Barbera, C Amato, A V Agostara, G Pantuso, G Orlando, N Finocchiaro, A Picciariello, D F Altomare, L Vincenti, G Tomasicchio, N Paradiso, A Dezi, E Pinotti, M Montuori, L Siragusa, C Pathirannehalage Don, G Sica, G Costanzo, V Usai, I Carrubba, M Pellicciaro, M Franceschilli, B M Pirozzi, F Santori, M B Busso, A Mariani Ivanikhin, A M Guida, B Sensi, A Divizia, F Blasi, V Giudice, L Orecchia, R Angelico, G Bacchiocchi, L Tariciotti, F Billeci, C Quaranta, C Cascone, M Materazzo, L Toti, T M Manzia, N Di Lorenzo, A Monaco, D Pedini, G Bagaglini, L Keçi, P Lapolla, A Mingoli, G Brachini, B Cirillo, P Sapienza, P M Cicerchia, C Leonardo, E Bologna, L C Licari, M Zambon, G Mazzarella, A Falasca, G Duranti, R S Flammia, V Asero, A Bernardotto, F Scarno, S Meneghini, S Giovampietro, B Binda, A Tufano, V Palombi, E De Meis, M Rocchetti, C De Padua, M Mansi, P Bruzzaniti, P Familiari, S A Nottola, F Fleres, E Cucinotta, S A Biondo, F Viscosi, N Catarsini, C Mazzeo, M Iannello, V Testa, A Gattolin, R Rimonda, F Riente, E Travaglio, F Allisiardi, P Guffanti, F Ferrara, M R Barbieri, A Puzziello, A Mollo, E Donnarumma, F Steccanella, A Oliviero, G Lopez, C M Di Maio, S Olmi, F Di Capua, A Camillo’, A Carresi, M Uccelli, C Rubicondo, R Rosati, U Elmore, F Puccetti, D Socci, M Clementi, A Giuliani, S Tontoli, A Nisi, A Grasso, S Derraj, J Di Biasi, I Tucceri Cimini, C De Nunzio, S D’annunzio, A Nacchia, G Gallo, G Lisi, D Spoletini, S Signore, R Menditto, M Carlini, D Sasia, E Dalmasso, A Trucco, E Olearo, M C Giuffrida, I Morra, M Maione, A Puppo, A Marano, G Preve, B Vercellone, E Frola, E Beltrami, I Peluttiero, G Giraudo, M Migliore, S Armentano, E Alessandria, A Daniele, P Elisa, L Bonino, M Borghese, D Bono, G Canova, M Zago, A Nicotera, L D Bonomo, L Gattoni, N Cillara, A Deserra, A De Lisa, E Coccollone, A Cannavera, R Cabula, F D’agostino, M Deplano, C Chillura, M L Robuschi, A Borzacchelli, C Margiani, M C Murru, G Pellino, E Lieto, F Cardella, A Auricchio, S M C Erario, G Del Sorbo, O Lidia, M D’ambrosio, F Menegon Tasselli, S Mastroianni, F Selvaggi, G Calini, G Terrosu, L Clocchiatti, V Morinelli, C Valotto, F Traunero, G Vizzielli, S Restaino, E De Gennaro, U Baccarani, A Andriani, J Mauro, P Frigatti, E Martin, S Pregnolato, L Driul, A Zullo, G Carcano, L Latham, D Inversini, A Vigezzi, G Ietto, R Marta, A Marzorati, E M Colombo, S Garbarino, N Palamara, M Tozzi, G Piffaretti, M Franchin, L F Festi, M Odeh, M C Fanelli, J Costa, V Pappalardo, A Scorza, M Cannavò, A Ballabio, A Barina, B Franzato, L Rubin, D Brugnolo, O De Simoni, S O Senica, A Ozoliņš, E Vjaters, N Jain, R R Apse, A Truskovs, I A Apse, P Jukonis, J Jukonis, A Tumova, H Sivapalan, O S S Piirtola, A E Berga, H Tumegård, N Samalavicius, V Nutautiene, O Aliosin, V Eismontas, A Dauksa, L Venclauskas, K Jasaitis, D Lazauskaitė, K Urbonas, V Šlenfuktas, I Grikytė, M Jokubauskas, V Simanaitis, Z Dauksa, R Gudaityte, R Riauka, U Krunkaitytė, S Svagzdys, A Šikarskė, J Pakrosnyte, E Stratilatovas, A Piscikaite, G Žaldokaite, T Poskus, V Olekaitė, J Psaila, P Andrejevic, C Cini, K Cassar, S Mattocks, M Sammut, S Mizzi, L Abela, A Micallef, K Pace, B Schembri, R Cini Custo, M Harmsworth, B Farrugia, F Theuma, R Gatt, G Montebello, N Spiteri, J Galea, L Vassallo, L Fava, A Ebejer, K Carabott, K Iles, L Casingena, G Apap Bologna, G Debono, J Galea, M Sammut, S Huisman, W Leclercq, F De Ruijter, J Konsten, D Adramanova, A Nikolovski, A Otljanski, M Kisielewski, K Richter, N Kłos, T Stefura, K Macheta, I Alsoubie, J Tempski, N Wolińska, W Wysocki, E Starek, N G Nowak, R Tarkowski, G Wallner, J Kobak, E Mączka, K Żak, A Ziółkiewicz, K Kułak, E Langa, Ł Łaba, I Krzesińska, M Bobiński, K Frankowska, S Dziurda, J Martyna, J Radulski, O Lulko, J Tomczyk, M Jasiński, P Major, J Rymarowicz, Z Zielińska, K Siuda, M Kęska, P Wites, P Błasiak, A Sierżęga, J Kuciel, D Tomczak, B Molasy, P Nieroda, E Buras, R Gonçalves Pereira, S Patrocínio, S Reis, C Rolo Santos, L Moniz, P Bernardo, C Osório, L Matos, L Carvalho, J Marques Antunes, N Alçada, A Marçal, N Tenreiro, D Martins, C Leal, C Ferreira, R Marques, F Freitas, C Marques, A Melo, S Silva, B Vieira, U Fernandes, B Freire, S Fonseca, M Reis, C Macedo Cardoso De Oliveira, J Mendes, M Carvalho, J Macedo, J Oliveira, C Mexedo, D Sousa Silva, J Davide, E Emanuel Do Vale Gonçalves De Castro Alves, M Ginestal, M D Santos, J Santos, C Robalo, V Valente, J P Fernandes Dos Santos, I Mesquita, B Teixeira, M Marques Monteiro, M I Silva, M Nunes Coelho, M Teixeira, R L Silva, L Lopes, A Ribeiro, C Lima Da Silva, T Correia De Sá, M Martins, M Costa, C G Gil, M Barros, R F Santos, A Silva, J Pedro, M Costa, T A Ventura Antunes, M Pascoal, R Andrade, M Duque, I Prior, M Lemos, P Pinto, P Guerreiro, S Castanheira Rodrigues, E Barbosa, A L Martins, A Pereira, S Vaz, A Fareleira, J Rocha-Neves, L Dias, F Girão De Caires, A Pereira-Neves, A I Oliveira, J P Araujo Teixeira, J Ferreira, M Almeida, M Vb Machado, J Nogueiro, F Gomes, E Campos, F Jácome, C Coutinho, R Ribeiro Dias, J P Vieira De Sousa, E Francisco, C Borges, S Pereira, C Pereira, N Machado, R Calaia, J Pinto, T Corvelo Pavão, L G Santos Sousa, F Cunha, D Melo Pinto, R Pereira, I Dinis, C Ferros, J L Pinheiro, A J Santos, B Barbosa, D Gaspar, M Pinto, D Pereira, N Araújo, B Alves, H Barbosa, D Silva Araújo, M Afonso, B Guimarães, M Campos Coroa, M J Susano, J Azevedo, M Pereira, P Miranda, R Garrido, M Oliveira Mourão, M Ferreira, A Duarte, P Félix, I Antunes, N Fernandes, C Gil Corrêa Figueira, I Figueiredo, M Gututui, S Oliveira, A Silva, F Madeira Martins, A Cabral, C S Rodrigues, B Gama, R Silva Borges, R Lourenço, A C Longras, R Araújo, I Peixoto, A Santos, D Matos, A Lopes, L Claro, D Cardoso, A Martins, M Silvestre, G Sousa, M Santos Bessa, V Cardoso, C Velez, A Machado, R Pedroso De Lima, M I Matias, S V Matos, M Rocha Melo, J Bolota, M Rente, A Santos, M L Antunes, A Bárbara, E Rosin, J Oliveira, S Leandro, M Damasio Cotovio, S Morgado, G Fialho, N Andrade, F Valente Costa Pinto, H Capote, S Morais, M Buruian, F Taré, G Santos, D Rosado, C Costa, T Mogne, N Pratas, B Cordeiro, M Brito, D Salvador, S Carvalho, V Capella, M Carracha, R Alves, C Miranda, F Rebelo, T Teles, M Ferreira, B Cismasiu, P M Costa, S Henriques, M Trindade, J Vaz, M Gomes Vieira, M André, A Macedo, J G Gonçalves Nobre, J Prosil Sampaio, A R Mira, A L Preto Barreira, P Botelho, D Melo Pinto, C Quintela, M J Quelhas, D Pereira, A Cruz, C Mesquita Guimarães, C Pinto, F Maldonado, F Sales, F Marrana, A R Reis Aguiar, L Freire, L Farias, G Faria-Costa, A Castro, T Moreira Marques, G Cardoso, J Ribeiro, M Fragoso, C Silva, M Vasconcelos, M Picciochi, A Sampaio Soares, J Frazão, R Miranda Pera, F Gaboleiro, F Ramalho De Almeida, A Mendes, F Afonso, J Fontaínhas, M Reia, M Angel, C Aguero, M Guerrero, J Dominguez, I Borges Da Costa, L Ramos, J Fernandes, C Assis, F Fonseca, S Carvalhal, A Caiado, F Brito Da Silva, B Miguel, J Moniz, M Pires, B Leal, J Nunes, F Matos Sousa, T Vieira Caroço, M Romano, M Ângelo, A Rodrigues Ferreira, A R Prata Saraiva, F Mano, F Rodrigues, T Branco, S Gaspar, F Neves, M Alves, A P Ferreira Pinto, M Peyroteo, C Baía, M Marques, A M Correia, J O Silva, R Monteiro, P Silva-Vaz, J Gomes, M J Moutinho Teixeira, T Brito Neves, F Meruje, C Sheahan, D Macnamara, J E Linares Gómez, S Clare, A Walmsley, A Tiwari, T Khan, G Dowling, K Hudak, E Craig, A Dhannoon, S Saeedi, J Shah, M Jordan, T Buckley, J Engler, M Reilly, J Ariaratnam, M A Hinn, K Benn, S Petropoulos, H S Hansen, S Browne, S Keelan, M Basta, R Pornsakulpaisal, G Tan, A N Niyaz, N Lakic, T Warhust, Y Q Chang, Y Qaoud, D Mwipatayi, D Kearney, C O’reilly, Y Gamper, R Dhillon, V Patel, K Gallo, H Mulvaney, P Edwards, S Tiller, E Gorecki, M Jordan, S Daswani, V Jones, I Asekomhe, N Clausen, M Khan, A Feldiorean, A Pezeshki, T Khedro, J Beyer, G Byrne, W Irfan, A Poluha, C Fager, L Gilligan-Steinberg, J Yarp, E Byrne, M Lorico-Rappa, A Hassan, T Keogh, W Denning, K Horton-Schleicher, T Deane, A Haren, A D Md Hamsan, N Mukerji, M Dwivedi, S M L Cheah, P X Kwek, S Arshad, A Alzaki, I Cornila, H M Feizal, M Barry, A Semar, P Gorman, A Gordon, C Mcsweeny, N Layyous, D Peiris, S Pan, J Larkin, M M Umar, C Doherty, C Mitchell, H Mcelvaney, Z Sabz Ali, S Adesokan, A Murphy, A Yeo, L Hayes, C Owens, N Crawley, E Macinnis, S Elekes, K E Oderoha, I Mac Mahon, B Moran, P Matreja, K Conlon, P Piankova, M Z Farran, S V Kashyap, M Puntambekar, M Sampat, S Heaslip Owens, A Maheshwari, A Soo, J Butt, K O’brien, M Almutairi, C Mcfeetors, M Kerin, M G Davey, W Murray, A Nasehi, A Graham, C Mathew, S Azam, K Chua Vi Long, S Stafford-Johnson, D Ejaz, Z Siddiqi, A M H Kon, M Parmar, C Peirce, D Shomoye, A Alabi, N Salzberg, V Gallippi, J Aziz, C Melly, H Gill, H W Yang, S E Eltahir Ibrahim, J S K Rugber Singh, N Bacalbasa, I Balescu, M Muresan, V Dudric, I Imihteev, V Chelaru, T M Băiceanu, M Alexandra, A Bashimov, M Voda, D Muresan, R Varadi, M Muntean, Ș Macovei, R Checiu, D Buf, F Grama, A Chitul, C Bezede, E Ciofic, A Beuca, V Bintintan, D Muresan, A Raluca-Cristina, R Rad, S Șușman, C Suciu, J Abu Arif, A David, M Blaga, B Olesea, D A Butnariu, R R Scurtu, R Rad, P Claudia-Mihaela, M Girlovanu, D Costina, R Drasovean, G Tartiu, S Tache, N Irina, A Streang, A Trif, I Romascan, E Stănică, E Vass, C Cucoreanu, E A Toma, V Calu, O Enciu, A Laceanu, R Maria-Zamfirescu, I M Matache, I Mușat, D C Piriianu, A A Georgescu, A Miron, M Tartalea, N Al-Ugeily, B Bogdan-Gabriel, A Alexiadi, A Zaharescu, C Ciubotaru, I Negoi, M Paunescu, I Iancu, I V Pop, B Diaconescu, P Dao-Zamfirescu, N B Patel, S Rafi, D Popescu, M Girbaciu, A Rădoi, C Breuer, A C Braun, B Stoica, I Gîlia, A Gheorghe, E C Popa, F Brichius, A Bucurica, V M Negoita, M Radu, R Bianca Ștefania, C L Socol, A M Baiceanu, S I Bubenek Turconi, L Valeanu, M Girel, M R Gavrila, M Pirvu, B Morosanu, M Ioniana, A Barbu, R Cornel, B Bogdan, T Bute, L Madec, E Burla, C Balan, E Alexandru, D Tomescu, M Popescu, M Dumitru, A Marinescu, I Petrusca, A Pasca, P Achimas-Cadariu, A Irimie, A Petrusan, M Puscas, S Titu, N M Jiboc, I C Vlad, D T Eniu, P Ciubotariu, V A Gata, A Rancea, C Iulia, D S Morariu, C C Nistor-Ciurba, I Gale, G Lazar, E Bonci, F Ignat, C Lisencu, C Dumitraș, F I Faur, M O Butuza, M C Marian, A Paul Luchian, H Bocse, I C Puia, C Andra, P Pop, S Moldovan, S Ursu, M Sorin, S Lunca, M Dimofte, A Musina, N Velenciuc, C E Roata, S Morarasu, M Velescu, F Mureșan, I Zaha, I M Rusu, B Mircea, D G Tauberg, M Kirov, V Kuzkov, A Nikonov, A Tishchenko, D Kulin, V Mironov, A Litvin, I Gunko, I Mazhega, R Bilenko, G Khrykov, Н Манкевич, M Karpenko, E Zagaynov, A Shilyaev, A Butyrskii, A Tatidze, Z Seytnebieva, A Aliev, M Rumyantseva, A Yanishev, A Abelevich, S Doktorov, K Zuev, A Lazareva, A Malov, D Borisov, Е Борисов, И Андрийчук, Z Galchikova, M Shemetova, K Maltsagova, A Bedzhanyan, K Temirsultanova, D Efremov, A Volkova, Y Frolova, A Minenkova, K Petrenko, A Sumbaev, M Bredikhin, E Bedzhanyan, A Mikhailova, В Тен, Ю Кудрявцев, A Novikova, R Pavlov, M Chernykh, N Boiko, В Данилин, G Stanojevic, B Brankovic, M Nestorovic, N Milutinovic, A Vukadinović, A Vukadinović, B Stojanovic, D Radovanovic, A Cvetkovic, I Radosavljevic, M Sreckovic, B Milosevic, N Markovic, M Spasic, M Pavlovic, D Lazic, R Vucic, B Tadic, B Kajmaković, Z Vilendecic, D Knezevic, U Bumbasirevic, A Stefanovic, P Gregoric, Z Perišić, D Micic, S Ratkovic, J Vasiljević, P R Bulat, V Jovanovic, O Mitrović, J Gunjic, D Potkonjak, N Grubor, M Reljic, I Palibrk, V Milutinovic, M Zivkovic, D Škrijelj, Ž Grubač, S Kadija, R Cerovic Popovic, L Andric, L Aleksić, S Kmezić, I Pavlovic, M Djukanovic, B Milojevic, Z Dzamic, M Petrovic, K Jeremic, I Pilic, B Milosevic, J Krstic, V Šljukić, M Veselinović, N Ivanović, A Janičić, A Jovanovic, D Cvijanovic, I Ladjevic Likic, M Radojevic, J Ćupić, I Dimitrijevic, M Miladinov, A Sekulić, D Nektarijevic, S Pantovic, D Šljivančanin, S Dugalic, A Đermanović, Z Radovanovic, D Radovanovic, M Đurić, S Zahorjanski, V Milosavljevic, D Zdravkovic, B Toskovic, U Marjanović, M Zdravkovic, S Petricevic, B Crnokrak, S Maric, A Sekulic, N Colakovic, B Kovacevic, I Krdzic, J Bojičić, T Sparic, A Ostojic, A Cokan, J A Košir, M Pakiž, S Potrč, N Kavčič, A Ivanecz, M Horvat, T Jagric, U Kacjan, I Perić, E Timošek, I Plahuta, Š Turk, B Crnobrnja, R Kovačič, A Dovnik, L Al Mahdawi, J Knez, J Grosek, T Košir Božič, A Tomazic, L A Suarez Gonzalez, S Busto Suarez, C De La Infiesta García, O Arencibia, D Gonzalez Garcia-Cano, M Laseca, A Martin Martinez, J A Guijarro Guedes, A F Rave Ramirez, Y Gil Gonzalez, A M Hernández Socorro, D Ponce Arrocha, A Cruz García, C Mendoza Rodriguez, E Catala, C S Romero Garcia, A García Trueba, V Georgieva, A I Hernández Álvarez, C Martinez-Perez, E De Valera, J J Gascón, E Lopez Alcina, L Samper Monton, K Aghababyan, A Valero Tarin, C Salinas Lozano, A Cervera, J C Catalá Bauset, B Ramia, F Marques Peiro, J De Andres, S P Iserte Juan, M Peñalver Gaspar, J Gilabert-Estellés, M De La Rosa-Estadella, A Fernandez-Colorado, M Serrano-Martin, A Gasulla-Rodriguez, M Juarez-Pomes, M E Ossola, G Sanz Ortega, J Gómez, M Del Campo, R Corrochano, M Galan Hernandez, F J Molano Nogueira, L Ibañez Vazquez, J Dziakova, A Zarza Martín, S Infante, D Moro-Valdezate, L Pérez Santiago, A I Molina, A Vinuesa Huesca, R Gadea Mateo, L Navarro-Sanchis, P Aracil-Boigues, C Jezieniecki, B De Andrés-Asenjo, Á Zamora Horcajada, F Natal Álvarez, L A Cuellar Martin, M Montes-Manrique, G Cabezudo, T Gómez Sanz, A Herranz Arriero, N Sierrasesumaga, M Rodriguez-Lopez, M Ruiz Soriano, A Sánchez Gollarte, A M Minaya Bravo, C Guijarro Moreno, A Galvan, E González, M Á García Ureña, A Robin Valle De Lersundi, J Ruiz-Tovar, A López Campillo, M Jimenez Toscano, S Salvans, S M Jaume Böttcher, À Godó, G Busquet Raich, C Téllez, J A Sánchez García, M Ribas Ardanuy, P Saavedra, A Sánchez Cabrero, J Clivillé-Pérez, M Durante Escutia, A Rabasa Rodríguez, P Aguilera, C Giménez Francés, M Ruiz-Marín, D García Escudero, M Valero Soriano, M Ramirez Faraco, S Galán Jiménez, P Alcón Cerro, J M Rodríguez Lucas, M Carrasco Prats, E Peña Ros, M D P López Sánchez, V J García Porcel, M Tamayo, J M Muñoz Camarena, R Albarracin Garcia, O Molina, P Moreno Sánchez, I Jiménez, P Pastor Perez, M B Agea Jiménez, D Candela Mas, M Artés Artés, J A Benavides Buleje, F M González Valverde, P López-Morales, A Sanchez, E R Bobadilla Romero, M Allue, L Ponchietti, R Latorre Tomey, D Torres Ramos, L M Jimenez-Gomez, S Valdés López-Linares, Y T Moreno Salazar, J Soto Galán, C Moreno De Alboran, A Prosperi, J J Osma, A Blanco, M Sánchez Rodríguez, E Valdivielso, Á Cejudo, C Perez Carpio, F Vasques Seabra Águas, E Sánchez Martín, S Pérez-Ajates, E Macarulla, A Álvarez Torrado, C Galmés, M Artigot, J Marin Garcia, J R Oliver Guillen, A López De Fernández, B F Fernández-Velilla San-José, D Ambrona Zafra, S Pérez Farré, J Ortega Alcaide, L Codina Corrons, E Sisó Soler, F D Gómez Báez, G López-Soler, E Gutiérrez Pérez, S Aix Molina, M Vallve-Bernal, A Varona Mancilla, Á M Aldama Martín, R Casanova, L Sevillano, J J Muela, M Rodrigo Rodrigo, I Goujon, C Fructuoso Iniesta, S Pérez-Bertólez, E Monclus, L Fernández, M G Silva Hernández, F Vicario, P Garcia Pimentel, R García Álvarez, N Bouzó Molina, Á Ramiro, Z A Calderon Barajas, R Sanz, Z Xia, P Rodríguez, C Sarrais Polo, A Martínez López, C Estrada, P E Gómez De Castro, D Fernández Martínez, L J García Flórez, I De Santiago Alvarez, L García Alonso, G García-Santos, L A García González, B Carrasco, D W Silva-Cano, P Del Val Ruiz, G Martínez Izquierdo, A Corteguera, J Iturbe, E López-Negrete Cueto, A Cembellin, C Ramos Montes, G P Ibero Casadiego, M Prieto, I Villalabeitia Ateca, T Pascual Vicente, B Villota Tamayo, A Perfecto, S Mambrilla, I L Ramos Del Moral, V Jiménez Carneros, P Pastor-Riquelme, A Franco Lozano, L Alonso-Lamberti, M Lozano, J García-Quijada, R Zhang, A García, M M Martín, M Yeh Ahumada, A Carreño Pallarés, Á Pérez Rubio, R E Goran, N Gómez Diez, G Zomeño Bravo, J C Bernal-Sprekelsen, G S Martínez Fernández, R Marquina González, C Toribio-Vazquez, A Eguibar, H Perez-Chrzanowska, P Serrano Méndez, S Valderrabano Gonzalez, F J Reinoso, A Yebes, M B Alonso Bartolomé, H R Ayllón Blanco, I Cristóbal, I Pellicer, R Arenal González, J D Zafra Angulo, V Duque Mallén, C Gracia-Roche, I Gascon Ferrer, D Aparicio-López, M Antonio, M Herrero Lopez, S Saudí-Moro, M Á Gascón Domínguez, T Gimenez Maurel, B Matías-García, N Morales Palacios, M Diez Alonso, A Gutiérrez, S Soto Schütte, M Bru-Aparicio, R Jiménez Martín, A Sanchez Pellejero, S Vázquez Valdés, C Vera Mansilla, A Quiroga, F Mendoza-Moreno, L Diego García, E Serrano Yébenes, F Mañes Jiménez, I Lasa, E Ovejero Merino, L Casalduero, Y Allaoua, A Blazquez Martin, D Córdova García, R Alvarado Hurtado, P Laguna Hernández, F Garcia-Moreno Nisa, H Juara, C Aldecoa, F J Tejero-Pintor, M García, E Aguado Saster, E Ruiz De Santos, A D Bueno Cañones, S Pérez Fernández, M G Alija Garcia, R Urruchi, R Rioja Garrido, M Madrid, A Bordell, I Arranz Chamorro, P Rodriguez Cañal, D Pacheco Sánchez, P Marcos-Santos, S Veleda Belanche, J L Maestro De Castro, M J Blanco García, E Laita Jiménez, L Leal, L Vaquero Perez, C Barbosa, M Ramos Carrasco, I Garcia-Saiz, P Rodicio, F Acebes García, A Lizarralde, A Sanchez Diez, E Ferrer-Inaebnit, J J Segura-Sampedro, M Alfonso Garcia, G G C Gutiérrez-Cañadas, A Oseira, N Torres Marí, J Loyola, B Villalonga, P Camporro, N Pagès, E Colás-Ruiz, J A Cifuentes Rodenas, M Castro Suárez, R Moll-Amengual, J Mata, J Fernández Manzano, B Gómez Pérez, J Gil Martínez, Á Cerezuela Fernández De Palencia, A Aliaga Rodriguez, P Gómez Valles, A Delegido García, E Gil Gómez, M J Montoya, A Navarro-Barrios, V Cayuela, J J Ruiz Manzanera, M I Jiménez Mascuñán, A I Gutiérrez Fernández, I Sánchez Esquer, P J Gil Vázquez, D Ferreras, A Balaguer Román, F Gómez-Bosch, F Alconchel, A Romero, V M Durán Muñoz-Cruzado, C J García Sánchez, A Tejero Rosado, B Ruiz, M D C Roman, J J Rubio Fernández, Á De Jesús, M Ostos Diaz, C Quintero-Pérez, A Sánchez Marín, M Alvarez Aguilera, M Leal Mérida, I Ager, J Sancho-Muriel, M Frasson, B Castro, P Guerrero, M Nieto-Sanchez, Q Cruz, H Cholewa, L Hurtado-Pardo, D Plazas, M Serrano-Navidad, J Castillo, R Ballestero, V Valbuena Jabares, C García, N Garcia Formoso, E Herrero Blanco, N Suarez Pazos, C Cagigas Fernandez, M Gomez Ruiz, Y T Benic Yoris, M Merayo, M E Gonzalez Fernandez, S Alonso Batanero, J Rodríguez Gómez, A Landaluce-Olavarria, B Estraviz, I Markinez, D Gómez, L Fernández Gómez Cruzado, M González De Miguel, J M De Francisco Rios, A Urigüen, A Gómez Del Pulgar, E Espin-Basany, A Gil-Moreno, S Bellmunt-Montoya, C Dopazo, S González-Suárez, R Blanco-Colino, P Olid Mayoral, M González Antúnez, E Del Barco Martínez, Y Bejar Dolcet, D Gil-Sala, C Marrero Eligio De La Puente, L Sánchez Besalduch, M Barrio Zaragoza, J F Lopez Lozano, C Olaria, S Catalan Sanz, E De Ciria, L Segura Farran, N Montes, J M Zanca Gómez, I Puig Escobar, M Armengol Alsina, M San Nicolas, M Escura, M Sánchez, M Rivas Agudo, L Aalouf, D Maya Salas, L Hernando Marín, N Umpiérrez Mayor, J L Sanchez Iglesias, Y Fernández Henriquez, I Feixa Molina, M J Gomez-Jurado, V Bebia, M Racine, M Sauvain, A Mujcinovic, D Bolla, Y Dimitrov, N Arenja, C Riboni, J N Marx, M Kalisvaart, C Andreou, P Brandts, U Pfefferkorn, L Eisner, U Dietz, J Gass, J Metzger, A Scheiwiller, V Kremo, D Gero, M Bueter, F M, R Schulz, F Mongelli, I Soave, D La Regina, C Canonica, F Sabbatini, P Gaffuri, S Spampatti, C Santarelli, M Di Giuseppe, M Marengo, S Passoni, G Dellaferrera, D Provenzi, L Bernardi, A Papadia, A Cristaudi, M L Gasparri, P Aurora, S G Popeskou, P Christian, M Antonilli, F A Scalvi, V Sevas, G Palumbo, L Maoloni, C Cencetti, G Pagnani, M Hitz, J Dürmüller, S Däster, G F Hess, N L E Aegerter, P Sedlaczek, S Soysal, J Zeindler, F Angehrn, L Müller, S Taha-Mehlitz, F Haak, F Nocera, T Karli, I Lazaridis, A Tampakis, A Lalos, U Friedrich, B Wiesler, N Varathan, R Frey, G K Kurtoglu, A Aghayeva, C Turam, G M Kurtoglu, M Maarouf, B Baca, S D Ilhan, Y O Koyluoglu, M E Seker, M Ulufi, M Erkaya, F D B Kılıçkan, S Bayrakceken, B B Ozmen, B Togay, M Doğan, T Çetinkaya, E Tuzuner, T Karahasanoğlu, I E Yavuz, I A Bilgin, S S Kekik, C Turam, E Dönmez, M Dikeç, N Ramoğlu, N Yurtseven, O Takmaz, Ö Orhun, N Karadeniz, O Saylık, E M Uğur, E Ada, A Durbas, I Hamzaoglu, K Uzun, M Gungor, I Y Gebedek, E Ç Hayırlıoğlu, M Halıcı, G O Ceyhan, A U Mutlu, E Aytac, M Gulmez, E Capkinoglu, T U Yilmaz, A Ozer, S Keçeoğlu, Z C Eraslan, V Ozben, O Dülgeroğlu, C Saraçoğlu, M Erkan, C Uras, H D Copur, N U Dogan, E Topal, I Demirsoy, D Korkmaz, U Can, D Buğra, E Sobutay, H Çakıt, E Ergün, S Zenger, C Bilgic, B Gurbuz, K Sünter, I Gecim, S Sefer, B Inceöz, M B Genc, A K Uygun, E Yücel, A Barcin, Y Altinel, Y E Aktimur, S Meriç, K Özdoğan, A I Sayar, A G Durmaz, I Çakır, E S Ünlü, G Kiran, A Dal, E Ozkan, S Kalkan, M M Öncel, Ç Çetin, G Yılmaz, H Karimi, Y Iskurt, E Yardimci, M Eker, A O Balkan, H B Gönül, E Herdan, M Ertugrul, T Takmaz, S S Kilinç, M S Pinar, F Yıldıztekin, E C Coşkun, I Olgun, G Ince, O Isik, M Şen, R Özata, B Eroğlu, B Büyükpolat, G Ishakoğlu, D Özen, M O Aktaş, B Kılıç, T Yılmazlar, M S Koçbey, N Işıklı, R G Yıldız, T Baghirov, E Uygun, M Yugaç, E Keskin, B E Bozkurt, S Tas, Ö Ö Türkmen, B Ata, B Alan, N Işık, T Göver, T Bisgin, E Özalp, S Sökmen, B Manoğlu, C Bakar, O Bozbiyik, U Zorlu, N N Zengin, B E Baki, E S Akbulut, S Tunali, B Kutlu, Y A Oğuz, B Küçükateş, E Yildiz, A N Sakmar, T Yoldas, Z E Akgün, M A Korkut, C Çalışkan, E Aksit, E F Aksalman, A O Koçoğlu, F Basci, B Yigit, O Ilhan, R K Liman, M Uzun, I Ağaçkıran, C Şahin, S Leventoğlu, Ö Kubat, I Genç, A Oyanik, F I Gurbuzer, N Satilmis, S Cam, M Ekinci, M Gönül, D Chasan, S G F Zara, Ş Sök, A Yalcinkaya, E T Acıpınar, U Timurçin, N B Afzal, S H A Gillani, S Gillani, S Yazıcı, O Cennet, M S Suer, Ö Kaya, E Z Uslu, A Tanrisever, C Yildirim, I N Gunenc, B Gül, I Özkal, H I Tileklioglu, M A Korkmaz, I Şirin, U Özbayrak, A Mıdık, E Domaç, A Karakoç, S Urek, A Yıldırım, M Ugur, M Doğru, E R Arslan, B Ular, M E Duymuş, A B Turhan, T Dogan, M B Ozkan, D E Benek, M Arslangilay, M B Yildirim, Y Yazgan, H N Tıraş, B I Şahin, M A Çapar, C Tatar, A Arı, O Batikan, M Güler, A E Naycı, M M Sevinc, H Şevik, C Oğuz, O O Karagülle, U O Idiz, A Demir, E Çakır, C Cakir, S Doğan, I Yıldız, M Gürlük, M B Cengiz, M Toptaş, O Akay, R Kaya, E Aydin, M Ç Çakıcı, A Yildirim, Ö Efiloğlu, B Demirtaş, A Iplikçi, G Atis, F Keser, M Culpan, M Çiçek, A Tahra, M K Akalin, A Izgiş, M Soytas, N Okkabaz, A Saylar, E Onaran, A E Askin, E Hashimov, S Bektas, K Sabuncu, A C Alagöz, I Karatas, A F K Gok, E Simsek, Z I Altunay, E Koç, Y Iscan, I C Sormaz, B I Yabaci, M S Ertürk, M Nazli, I E Saglam, E Erginöz, M F Ozcelik, M Gokden, C Guler, F I Gunaydin, S S Uludağ, B Ibis, H O Bozkir, M B Karaca, M C Ulucay, K B Oner, S Akbas, F Z Calikusu, H Akcan, S Ugural, A K Zengin, Z Ozdemir, I Demirbas, A Guner, M D Tepe, N Kıralı, B C Karabağ, D Pehlivan, A N Yuzgec, B Yıldız, B Akin, M Aktaş, E Karabulut, H Cepe, H Küçükaslan, S S Salih, M Uzun, Ö Yücesan, M E Reis, M Ulusahin, K Aşcioğlu, E Tufan, Q Saleem, K Saracoglu, M Ozbilen, A Kale, M E Geçici, Y Bulun, Ö Uysal, A Atay, B Şuataman, C Guducu, T N Çinar, H Kul, D Canpolat, M Ipekoğlu, M Aydemir, F Karahan, K Hizli, B Kaya, O C Yenen, B Gümüş, K Turmuş, S Vatansever, I Candan, S Sucu, E Balik, E Bozkurt, I H Özata, E Ozoran, T Tüfekçi, S N Karahan, A F Sarıkaya, O Agcaoglu, S Danacı, M E Ulutaş, I Hasirci, K Arslan, Ş H Metin, A Yılmaz, E Turan, G Şimşek, A Kılınç, M E Şahin, S Ozden, Y A Bayraktar, A S Maden, A Şahin, N Acar, O Erşen, E Balcı, H Yaldız, C E Guldogan, M M Ozmen, E Gundogdu, M Moran, B Celik, E Sivrikoz, K Karabulut, A C Dural, T Ikizoğlu, H Aydede, T Yalçın, A Dalkıran, H B Yapici, T K Uprak, Y Tatar, Y Aksahin, C Aral, S Z Cetin, D Artvin, Z Tatar, I S Karakuş, S Çatal, B Nalbantoğlu, S Bettar, Z Düzyol, B C Demir, M F Kırcalı, M E Taşcı, F Secil, E N Akkaya, I Kayılıoğlu, B Hekmatjou, A Çağlayan, M A Dadaşoğlu, C Fergar, M S Beden, E Kayhan, I Arslan, G Z Koçyiğit, B Beyoğlu, C Varan, U Sungurtekin, H Sungurtekin, U Özgen, B Çetin, A Pergel, M Uyan, E Erata, E Aldhahebi, B Aslan, Ş Kabalı, F Köse, I Keşaplı, U C Dulger, F Altintoprak, M Akçay, E S Cünük, M B Kamburoğlu, I F Küçük, M Yigit, E Sabuncu, A T Harmantepe, B U Aka, E Baş, T Yavuz Akça, A C Sarı, E Colak, M Candan, M E Kara, A B Ciftci, K Yemez, M S Uyanik, S Polat, G O Kucuk, S Ocak, Y Tosun, E Unal, C B Ofluoğlu, C Üstbaş, Ö Gangal, A N Sanli, S Sayır, C Karslioglu, I Ulusoy, A E Tufan, U Demir, M F Celayir, E Baran, A B Aksoy, D B Fırat, A Yüksel, O Güven, I Ertaş, Z S Kuzu, H M Köksal, M Gok, M N Görgün, P Yazici, S Ömeroğlu, B B Özdemir, E Kabul Gürbulak, M Ajredini, I E Cakcak, I H Atakan, A Göztepe, O Budak, E O Aydoğdu, E Erdem, C Yılmaz, J Özdemir, B Akin, D Alkan, E Cengiz, C N Donmez, E Akyüz, Y H Ergun, M G Ozer, Ç Ak, F Bolat, E Kasapoğlu, A Tatlisu, Y Yorulmaz, A Enez, B Ozkan, Z E Yilmaz, S N Altin, B Gunes, B Kaban, O Korkmaz, I B Demir, G A Öz, A B Tuluce, Z N Turkmen, F Yildiz, B Kandil, A Ulkucu, G Kıral, D Yavuz, Ü C Köksoy, Z C Kus, M B Erten, M Mutlu, F Hökelekli, T Aslan, Ş Orçan Akbuz, E Durmuş, T E Gökçek, H S Ulgur, O F Ozkan, H K Karakullukcu, A Yıldız, M Ceyhan, M Ş Çelik, M Kalın, E F Kirkan, A Demirbilek, M N Çelik, Ö Karbuş, S Demirli Atici, B Abud, H Erdinç, K B Ön, B Sevinç, N Damburacı, E Altiner, B Özdemir, Ö Manisalı, G Ural, M K Topal, T Can, S Ercan, S Kaynar, O Emanet, M Javadov, U B Demir, A Erdem, F Demircan, M Yelmenoğlu, E Seçen, B Alkışer, F Arık, E E Kaya, D Akkad, D Karaçam, U Karacam, M A Yücel, A Hatipoğlu, N Mumcu, O Karima, M B Dal, M Aljobbeh, J Montaser, M A Kara, F A Gultekin, O Deniz, N Kavak, B Kum, M M Ecir, Y G Yavuzer, Z Sezgin, U Koçdemir, B Yirmibeş, Ş Atalay, A May, S Varna, I Aydoğdu, A Abdel-Fattah, G Ramsay, M A Bin Badekruzaman, M Karvelyte, M Siamisang, S Clunie, M Mikalauskaite, A Nessa, C Joshi, N Jodeh, R Zafar, J Miller, R Basharat, T Singh, O Weston, R Loy, E H C Tsoi, A Chandiramani, R J Sumarlie, L Mitchell, M Yousif, M Qadir, M Gannon, E Y H Tong, J Tsang, M Elmarghani, J Kaczmarek, J Luangboriboon, R Mcewen, B Tse, M C Wong, Z X Wong, T Diffley, O Jaruwattanapradid, N Ng, L Mchaffie, C Wright, D Joyce, V May, S Emmanuel, A D’costa, A Kumar, C Hanganu, S Gourgiotis, A Townson, C Y Williams, E Baines, L Smith, N Elks, N Simon, R Chintapalli, A Banerjee, S Bhogle, B Ryan, D Maghsoudi, E Clifton, T Nott, H Vickers, G D Stewart, Z X Zhong, K Ioannou, C K H Ne, R Conci, E Fitzsimons-West, X L Ling, R Patel, R Sanghera, I Dokubo, I Seago, O Fairhurst, B Amin, M Aniq, E O’keeffe, S James, M Chowaniec, L Rutigliani, M Hu, A F Ferreira, M Kalogeropoulou, G Hui, L Coakham, S Healy, K Gilanliogullari, G Nishimura, M Choi, R Lunevicius, D Aje, R Mcnicholas, C Newman, E Bollen, A Kumar, I Ferreira Xavier, L G Baloch, N A Koduah-Sarpong, G O’sullivan, S Saseendran, A Bin Sahl, E Murphy, H Hussain, A Nicholls, S O’dolan, A Gidwani, E Patterson, L Loughrey, K Donaghy, K Hana, M Monaghan, S Ni Shandair, N Kulasingha, N Gormley, M Mcglinchey, E Dunlop, B Dunwoody, S H Lian, S C Jayasangaran, E Logue, P Goswami, P Ann Jacob, C O’kane, A S Ab Razak, K Govender, E J Mccann, S Perry, S Mckendry, A Pachchigar, A Singh, S Dindyal, S Pendyala, V Venkateswaran, L Alhoussan, F Feil, T Hughes, Y Gerçek, M Choudry, O Haidar, T Rujeedawa, T Speirs, J Odendaal, J Chu, A Shahab, S Ranjithkumar, V Pillai Rajendra Prasad, T Linn, J Clark, M Sharma, S Lockwood, S Chawla, R Deshpande, N Long, J Rees, M Kobetic, D Fawcett-Till, O Ferris, G Harrison, T Hibbs, C T W Tsang, L Hurley, T Jichi, C Badrinath, L Applebee, K Ecott, T Sullivan, B Piecha, A Jagadish, Z Sajjad, R Griggs, S Joyce, R Spurgeon, M Ali Khan, M Hobrok, R Roberts, J Mckenna, D Davies, P Eaton, L Bishop, S Magaway, K Denholm, S Doyle, R Deshpande, M Salter, V Chan, K Mockford, J Heinz, I Ahmed, M Coverdale, R J McKillen, M Maguire, N Mclees, J Lau, L Mcguigan, A O‘Neill, D O’Hare, C Beggs, O Owolabi, J Hunter, J Kinross, J Kotecha, R Doherty, V Patel, E Wagner, L Hodges, H Hassan, K Sribaskaran, K Pouris, S Jain, A J Kim, S Gill, S Nand, O Toutouza, A Joshi, L Thornley, R Chen, M Ogunjimi, A Mahmood, R Chong, M Yanai, D Bae, J Dhaliwal, A Lovejoy, A R Akhbari, N Mayor, C Kontovounisios, S H K Yap, M Damarla, B Ooi, M A Ng, A Vargas Zhang, S Limbu, K Nyamakope, Y Agarwala, N Zingas, C Cleasby, E Smyth, J Drmota, Q Z C Yang, C Vedi, S Samarendra, V Nayak, S Rajendra, M Choudhry, K De Stadler, S Bandyopadhyay, G Bond-Smith, O Collart, K Baffour-Awuah, R Shah, J Mcnamara, G Tadikamalla, B Wilson, C Cossins, H Li, L Ismail, H Soleymani Majd, E Chang, A Jallow, S Baldelli, P Alberti, O Grant, M Doody, Z Borawska, A Bowman, H Clay, Y Petit, J G Kimani, M James, S F Hussain, A Nezhentsev, M Emmerson, R Vijjhalwar, G Shaw, C Holmes, Y Ying, L Farache Trajano, A Anis, J Dequaire, A Hunter, R Danvers, M El-Nemr, C Hammett, M Pikoula, S Thompson, B Lander, S Dierksmeier, N Sadeghi, R Suribhatla, R Ahmed, S Pandit, W Thornton, T Thornton-Swan, A Johal, M Khan, A More, Y Tilahun, A Hauperich, R Gidda, I Vorley, Z Khan, M Sintler, A Van Den Broeck, M Georgiou, N Eardley, H Salem, S Yemparala, L Whittle, C Jagger, K Turner, L Vernon, K Aldred, P Manokar, A Moore, N Mistry, J Zgliczyńska, C Haylett, A Adeyanju, E Headford, R Khanna, M Deef, T Durkin, E Trayling, R Cowell, Z Khan, A Turner, K Noureldin, W Down, A Cyril, C O’halloran, M Shirke, M Epanomeritakis, A Karnati, G Sreejith, C Sinton, L Oliver, H F Ali Azamatullah Khan, E O’kane, J Trouton, B French, I Campbell, R Curran, C Brines, A Mcdermott, N Yang, S Vig, S Chowdhury, R Valecha, R Lau, M Nafis, S Thavanesan, J Marks, L Akaje-Macauley, N Patel, E Beard, A Nanda, C Mills, A Cheung, J Amalendran, L Todd, S Smolarek, T Warrener, S Sen, A Mudehwe, S Khan, B Wisden, G Lau, L Schanzer, Z King, K Giridhar, M Reed-Embleton, L White, P Filippidis, L Lee-Smith, O Carless, C King, M Herriott, N Saju, Y M Tin Maung, A Deligianni, A Mumtaz, S Khan, H Hill, S Sahdev, I Thomas, A Shah, S Saji, J Hargreaves, R Khan, A Tharani, R Ullah, J Ludgate, S Shrestha, E Bota, A Abdullah, M Allison, Z Patel, A Clark, I Suchett-Kaye, B Stubbs, B Holmes, A Santaniello, T Watkinson, A Crimmins, M K Gupta, S Abraham, A Bellringer, R Clowes, D Chatzopoulou, A Kiran, J Eid, N Rao, J Caterson, F Soggiu, H Malik, I Jose, K Kavallieros, C Rizk, B Thummala, K Li, A Goel, R Pantula, J Toh, M X Fu, C C Ho, K Vivek, E Owen, A Day, A Jamieson, H Hassani, T Thavarajah, M Vivekanandan, A Muneer, M Chauhan, D Veeramootoo, S Ali, J Dosanjh, F Newing, E Jose, H Chauhan, A Kulshreshtha, E Thomas, P Patel, R Chhabra, B Sajan, S Ragavan, R Hariharan, I Lam, B Fleet, N O’hara, A Wright, V Reddy, H Darweesh, A Khan, S Handa, D Kewada, F Rana, H Williams, F Bombieri, N Shah, D Pestotnik Stres, A Menon, L Selvam, A Pusok, C Street, M Zohdy, B Aguirrezabala Armbruster, I Okoye, L Potts, M Fayyad, K Bajaj, H Alfa, N Sivakumar, N Duncan, C Roxburgh, L Huang, K Lukito, X Huang, S Baig, C Y Chan, R Philip, L Shaheen, J Dodds, H X Yeow, M Devindran, Z C Sia, J H Park, B Furze, N Yung, M Vipond, M Jones, F Asekun, A Dembinska-Kenner, R Saleh, E Larkai, J Harris, M Cherian, H Louden, V Bisbinas, A Behl, R Hughes, R Smith, Z Zaman, O Hoskyns, H Carlton, C Thorn, M Tourky, J Tan, K Sen, J Elsey, J Bevan, N Ko, S Kalidindi, M Rooney, K El-Boghdadly, S Zafar, A Wyncoll, P Morillon, M Sennaraj, H Mahfouz, A Afzal, R Sibal, T Suji, H Headon, A Abdul, S Ahmed, J P Mcnally-Reilly, K Omran, C Mulcahy, M Aftab, M Haghighat Ghahfarokhi, M Farhangi, W Y To, M Ho, T Patel, A Siu, H Choudhry, M Huntley, Z Hussain, G Wong, R Maguire, M Khan, A Potts, M Ahmed, G Ramesh, O Kolade, M H Siddique, E Griffiths, M Qureshi, M Hoque, S Laulloo, K Das, N Waheed, T Y T Tang, R Ahmed, R Habib, R Vyas, S Watson, K Theodoropoulou, M Siu, N Yu, N Islam, J Burnett, A Besso-Cowan, D Hirani, I Zsolnai, Y K Chen, O Dupere, L Keitley, C Park, I Verma, J Chan, J Tavner, M Nicolaou, N Y S Lee, R Hegy, R B Y Lee, K Y L Yi Lun, D Reyes, S Dong, J Thornton, P Eaton, L Bishop, S Magaway, K Denholm, S Doyle, R Deshpande, M Salter, M Weir, A Gibbs, A Al-Shaye, M Alwahid, A Tait, S Smith, A Doye, K L Law, T G Groot-Wassink, G Geller, K Seebah, O Cox, M Kalogeropoulou, A Shetty, D C J Oh, E Lee, B Packham, M Aarons, K Saadeh, J Mui, R Huynh, M Eid, N Honey, J Kaur, R Hand, L Lai, C Koubaesh, H Maqsood-Shah, J Suresh, O Collart, G Bond-Smith, R Shah, J Mcnamara, G Tadikamalla, B Wilson, C Cossins, H Li, H Soleymani Majd, L John, E Chang, J G Kimani, A Jallow, S Bandyopadhyay, S Baldelli, O Grant, M Doody, Z Borawska, A Bowman, C Foster, H Clay, Y Petit, M James, S F Hussain, A Nezhentsev, M Emmerson, R Vijjhalwar, G Shaw, C Holmes, Y Ying, L Farache Trajano, A Anis, J Dequaire, A Hunter, R Danvers, M El-Nemr, C Hammett, M Pikoula, S Thompson, R Shah, S Dierksmeier, N Sadeghi, B Lander, R Suribhatla, R Ahmed, S Pandit, W Thornton, T Thornton-Swan, A Johal, M Khan, A More, Y Tilahun, A Hauperich, R Gidda, I Vorley, E Lewis-Orr, S Reilly, A Bhatia, K Goves, M Blesson, L Purser, L Cobb, M H Sarker, N De Sousa, S Syed, N Rajendran, J Tan Sue Wei, G Navakumar, V Butnari, M Senthilkumar, Z Q Chew, L Zekaite, S Paranietharan, M Haque, S Balenthiran, K J Chin, S Ahuja, T Moothathamby, F Moniati, Y Z Kong, E J H Lee, L Sharma, P Found, Y Sivakumaar, H Senior, N Simeen, A Arora, A Chu, R Mizori, T Beazer, N Patel, J M Dudziak, T Squeri, F Weston, M Zhou, J Van Ross, S Tian, J Bedford, R Lam, F Karami Tireh Shabankareh, H Donkin Everton, K Wilson, A Richens, D Bragg, A Akbari, A Awodiya, S A Osula, F Gerges, I Gerogiannis, L Daniels, S Seth, Z Baxter, E Nour, L Fitchford, C Perrott, M Abuelgasim, J Flanagan, S Gillani, H Lewis, O Dunscombe, P Steven Goodwin Moughton, A Mahmood, J Hirniak, A Moses, P Kapsampelis, A Ahmed, D Burke, C Anderton, E Lee, M Conley, L Khan, R Surti, F Waseem, M Sharma, A Imran, R Motiwale, B Singh, S D Sa, S Charuvila, M Z Lorgat, R Chowdhury, P Gogineni, A Finney, A Tzortzi De Paz, S Panesar, A Sidki, C Warwick, S Kadambande, J Patel, A Rao, A Sharma, S Ramewal, P Ramesh, M Norwood, P Mehta, M Bhatia, S Tiwana, E Foote, F Rushton, S Asi, R Notaney, R Sinha, J Vibhishanan, S Gupta, R Conci, R Chintapalli, S Ahmed, J Sagar, N Ragge, S S Rizvi, M Ashraf, N Cirocchi, R West, E Obiri-Darko, Y Rai, S Hussain, N Ul Ain, F N Amir, C Smart, R Melomud, D Saad, S Bengeri, S Kuna, M Chaudhary, O G Olanite, F Khan, S Nausheen, R Dodds, H Boyden, D Donnelly, O Adegboye, L Osborne, S El-Barraj, J Shukla, N Raza, A Khalid, D Agrawal, K T Kyaw, S Borhan, G Pangrazi, I Ioannou, A N Ahmed, G Savvides, M White, S J Puthur, S Bridgewater, N Waraich, R Bryce, R Khosla, L Penhaligan, A Desai, Z Ehsan, O Mostafa, Y Kamel, B Keeler, R Rajivan, V Omar, S Gamadia, D Rana, S Srikumar, A Heidari, A Ahsan Akhtar, A Tarafdar, J Barry, D Davies, A Curr, I Jimenez-Reid, J Mckenna, E Garry, N Lallmahomed, K O’leary, E Barker, D R Jones, R Tabatabai, J Garg, E Henshaw, M Abdulshafea, S Prabaharan, H Wiles, R Bamford, F Blest, L Acquah, H Simpson, J Guerrero Enriquez, H Pringle, M Shahid, O Wharf, M Kumar, K Buadooh, M Hanson, I Mutanga, A Hardy, H Usman, S Shams, N Schottler, A Garg, V Sarodaya, S Ali, I Cullen, L Pregil, M Fernandes Silva Ramos, Y Hao, A C Das Chagas E Silva, S Sellahewa, J Li, J Cox, P Sinha, I J J Lee, E Voniati, H Phillips, O Mooney, M Sockett, P Patil, M Elsllabi, W K M Chan, L Grimble, H Richardson, A Johnston, T Kouli, J F M De Sousa, Y K Goh, C Grant, L Martin, Z H Peh, T Yap, E T W Tang, H Y M Lai, T Berry, M Rokia, E Tennant, I Lee, M J Rahman, A Kamal, S Ali, H X Hau, I Harten, M Eloofy, C Caldwell, R Leckie, F Tasnim, K Ravintharan, J Mcauley, F Cameron, R B Veerni, K Y Looi, A Ismaili, R Gresz, V Chong, L K Tan, K Singh, S Ashburn, R Shantha Kumar, M Kamal, H Kamal, I Aziz, P Stather, L Mylvaganam, E Deliyannis, B Tompkins, E Sikorski, F Harris, I Sanders, M Fakhrul-Aldeen, K Cross, A Tahir, P Yim, I Mayne, S Mahapatra, C E Ng, A Ortega, S Supparamaniam, M Bowman, M Sadler, M Rogger, A Gendia, J Ahmed, P Sivakumaran, C Brick, E Ansong, H Ha, A Sharma, N Wilson, B Manavi, L Zeze, N P Gupta, S Al-Hassani, C Williams, M Ramesh, B Shukla, E Drye, H Hussain, E Ghatauray, A C W Tan, F Adamu-Biu, J Arora, T Mcallister, S Fairclough, A Economou, S Utulu, K W Fung, I E Epanomeritakis, S S Zaman, E Olszewski, P Ballesteros, E C Okpii, E Walkeden, C Macklin, S Jamil, S Zhen, O Ahmed, A Saravana Kumar, H Uddin, R Hakim, M Mossanen Parsi, O Awoloto, K Rowe, R Mashadi, Z Mohammed, L C Chong, S Fairlie-Vogt, O Russell, W Ozarek, A Little, C O’farrell, R Miller, J Froud, K Thippeswamy, R Thomas, A Fitzpatrick, S Yoganathan, M Dunlop, K Ngai, T Liddell-Lowe, A Barrow, L Sharma, P Found, R Mizori, S Rabas, R Al-Housni, M Zhou, A Marton, P Chen, H Hasan, C F B Chan, W W Win Mar, A Kisiel, E Griffiths, M Yusuf, A Sinha, J Blenkinsop, K Rawlings, T Chaudri, G Westland, H Umar, S Lee, J Cherian, Z Gurhan, T Chaudri, M S Sheraz, D M I Khan, A Kucukmetin, D Malone, C Betts, M Ali, M Giblin, L Nowicki, P Korompelis, O Farley, M Fouweather, C Ioannides, H Yilmaz, R Landais, E X Ngeyu, C Erinjeri, D Badran, P Glen, J Aamir, A Aziz, P L Su, I Choong, M Reece, A Kulasekaran, T N S Tengku Saifudin, J Luckhurst, R O’hare, R Smith, Y T Siu, P H Kwok, V Sood, A Imran, K Fatima, Z Munir, T Kisova, N Rajendran, F Islam, E Teehan, H Sadik, U Patterson, N Rahman, H Harrisrhaj, D Govardhan, S Rajesh, Z Mumtaz, O S Ghori, H Nawaz, A Lysomirski, V Pikoula, R M A B Qadir, A Jones, Y Chiang, H Morsy, C Pazaiti, A N Asardag, I Ali, J Althonayan, A Badri, J Evans, E Chung, L Harriss, L Sinan, C Maxwell-Armstrong, A De, R Altman, F Varghese, W Chua, S Ahmed, M Javed, R Aljubure, A Bradley, S Moug, O E H Kemmett, I Underwood, I Fitzpatrick, L S Wong, B Vakeesan, K Potter, T Varghese, S Daren, A Hazrin Fazail, S Farajzadeh Asl, H Nautiyal, A Saadeh, I Vial, R Karimi, A Akthar, A Egiz, R Masood, A Qadri, T Chawla, H Yoon, X Liu, N Battersby, E Davies, R Chhetri, R Mclean, E Leaper, C Taylor, C Nicolaisen, L Cochetti, E A Zoumi, D Annable, H Thomas, Z James-Knights, C Lee-Kim-Koon, E Akpinar, M J Coelho, E Darke, T Morris, F Mayer, J Forbes, S Gold, H Sinha, E Batchelor, L White, J Lund, S Forrest, H Dial, F Chishty, M Oliver, J Meyer, N Gokhare Viswanath, A Ammar, A Lopez, N Minhas, N Sunny, K Haynes, A Alexiadis, S Doski, R Arbuthnott, R Memon, K Bukhashem, A Chaudhary, A Sheik-Ali, A Sebastiao, C Taylor, E Clarke, S Misztal, D Raja, S K Chui, I Khalil, A Metsel, M Whelan, S Forrester, O Oboh, B Bayley-Skinner, R Eastwood, E Akapo, M Jamshaid, G Osborn, R Woods, O Wooler, S Chumley, E Cotton, N Jarrett, N Amanda, J Johnson, M Quhill, M Al-Ani, S Kandanearachchi, S Khan, A Barrow, M Dunlop, G Williams, S Phillips, F Faryad, A Hughes, S Wong Ching Hwai, C Johnston, R Guest, A Kaddouri, L Ernst, A Szasz, C Gafrey, G Loy, E Roberts, S Baker, A Davies, Z Khan, M Leonidou, J K Chow, R Murugan, A Baker, R Burns, Y C Foo, F Sikora, L Chong, C Chan Ah Song, C Cosgrove, O Reeve-Chen, J Ma, L Mccolm, M K Zhang, F Hussain, X Y Ng, B Ingabire, B Wagner, T Bain, R Kovacs, E Small, S Lam, L Yao, L Ho, H G Paremes Sivam, K Dahal, A Stanley, M White, L Hayois, M Thillai, T Tay, E Davies, N Kamaruzaman, L Robinson, R Sherrington, Z Abid Sohail, S Lawrence, A Sohail, D Brown, S Parker, M Ohr, D Jasniak, B Crowther, F Jadoon, L J Melo, H Amin, R Q H Lim, M A Thaha, M Bath, S A Saadqain, Y Benallal, Z Jin, A Jackson, A Kuri, M Malik, H Jos, E T Goh, M E Paraskevopoulou, A Kythreotis, Y Naim Ahmed, S P Glynou, H Rehan, A Pereira Pai, A Georgiannakis, N Dworschak, S Cho, B J Chow, Y Khan, T Enthoven, A Malik, I Latif, A H Lakhani, N Rahman, K Morgan, S Gkolia, N Khalessi, P L Z May, A Lwin, P Kanesaratnam, M Tudgee, R English, K Hu, N Pathanchaly, D C S Chien, M Holloway, K Gao, G Lekka, R Srinivasan, S Zafar, M R Peris, K Keiarash, H Kanesan, B Chong, M Kobus, D Dominic, D A Do, D Dennis, S Frankland, V Shah, B Youssef, U Sadia, A Hassouneh, A Jaipersad, T Chen, M Fulford, A Dawson, K Kapur, T Jha, P Zope, M Wilkinson, V Bhatnagar, J Knight, T K Madhuri, T Katsiari, N Pasternak-Albert, E Moussa, L Emms, M Lamah, Z Batool, A Sabesan, S Islam, M Baig, M Dave, X Liu, L Stuart, S Harvey, V Dam, P Ezuma, J Lee, S Suntharalingam, S Singh, P Preston, F Rickard, T Gupta, S Madden, C Jones, R Gidwani, X Wang, M Mcconnell, S Hanna, A Mcgettigan, K Brown, A Cios, C Ward-Bradley, O Hurrell, V Paice, N Mutsonziwa, S Pogoson, R Coulter, J Corry, L Mcclean, F Keenan, S O’dolan, A Plonkowski, R Brady, R Ooi, D Iles, L S Guillemot, C Low, S El-Barraj, J Epstein, M Pressler, I Ioannou, N Soliman, A Ktayen, A Macconnachie, J Menendez Lorenzo, J Tooke, K Noman, L Wilkins, J J Warner-Levy, R Evans, A Torrance, C Fear, H Vidis Humphries, G Velayudham, Z Jefferson-Pillai, J Brady, R Oza, D Singh, A Hamid, P Hartop, P Shojaie, J Mano, T Rajah, Y M C Cabdi, A Singal, M Afzali, J M Lee, D Bandyopadhyay, J Bennett, P Mendenhall, A Millett, S Davidson, R Kallam, M Pishia, H Butler, S Isidore, D Boutsias, K Bryce, T De Rancourt, J Barry, H Henry, A Aspinall, L Smith, C Conway, H Shanmugathas, C Huang, M Doherty, K Ahmed, T Chung, T Benson, A Habiba, J Singh Bhangu, B Bingor, L Fice, H Premanandhan, J Mckenna, I Jimenez-Reid, M Hoque, E Griffiths, J Loughran, M Laskowski, A Mehmood, R O’kane, M Mullan, R Mcclenaghan, G Kettyle, F Hosty-Blaney, X H F Chan, S Sunny, M Kaur, S Vose, V George, D Killoran, R Doherty, Z Zagorac, A Pullyblank, R Ismail, N Anderton, L Dwyer-Joyce, R Morgan, N Zhang, B French, H Cox, J Tetro, A Clarke, K Shaw, R Iau, E King, S Patel, M Ovakimian, A Zahid, G Rowley, R Tanner, K Davoudi, B Shear, M Xu, K Karan, A O’reilly, S Ahmed, T Iskrenova Kirilova, A Ezekiel, M Shapland, M Mitra, T Krum, G Higginbotham, R Winayak, J Mustow, L Yorke, C Gibb, A Ilyas, M Pound, A Abouharb, D Burke, A Khan, K Aimar, J Bennett, E Kerman-Fiore, S Rao, A Dayal, S Chawla, L Wadey, C Smith, K Desor, R Surti, Z Gul, V Gourgiotis, L M Kaselampao, C Nguik, R Agrawal, Q Y Liaw, E Mckeown, A Hassan, K Krishnan, H O Glover, M Devassy, D Ogunyanwo, G Dickenson, G Elder, G Valdez, B White, I Hamilton, S Shah, S Sinha, K Punwani, I Peat, M Hayat, N Khalid, E Frankel, H Macgowan, A Braka, A Byfield, J Brooks, A Kashif, R Archer, S Akkaya, M Sood, S Akther, O Birkett, T Kadri, S Abdulmula, P Psefteli, S Chadha, G Reese, C Mark, M Asunramu, T Hess, S Mehta, C Tsang, K Syed, A Alocious, M Steinruecke, M Ashley, R Newton, J Wainwright, J Ayathamattam, H Wong, Z Elahi, M Fleming, T Ali, D Lloyd, K Brooks, C Kwon, A Mckerrell, T Jamadar, M D Barcelona, D Polluk, J Kwon, F Henry, S Ayirookuzhi, M Mwipatayi, M Cheruiyot, S Adam, T Cruickshank, K Gollub, M Revell, S Taiwo, A Atchade, A Harewood, A Niina, D Bosanquet, O Mckeon-Williams, T Szakmany, E Badhams, N Christensen, J Sammut, N Tay, J M Pollok, D Raptis, M Varcada, S Ganesananthan, R Chandrasekar, A Deshpande, L P Ghoora, G Hogg, S Staubli, M Banerjee, S Sunil Menon, A Sanz Pena, R Rana, A Mohamed, P Crabtree, A Flower, A Banerjee, Y Talabi, N Bishop, N Kupfuwa, D Ilangovan, H Qureshi, I Minty, R Baron, L Greasley, P Birkenhead, R Baron, M Shahid, N Rajasivam, W L Chu, N Punnoose, V Sundaresan, M El-Galley, K Prasansapakit, S Mitra, M Garlick, M Prakash, H Gao, C Parmar, F Lee, A Aich, A Ismail, M Kirupaharan, S Khan, K Patel, D Mclaverty, G Hammerton, H Walford, H Roberts, P Cautivo, H Abdel Kader, A Kimble, K Manaf, R Mackonochie, V Mitchell, S Ghaznavi, A Taheem, E Firth, H Chandler, K Eldessouki, J Fyfe, M Bhat, J Cavlin, E Pearce, S Patterson, C Johnson, K El-Badawi, M Gariballa, E Hale, H Younis, E Brackenbury, A Chappell, T Poulton, E Dawson, A Murray, I Mcallister, A Hassan, B Cunningham, T Chan-A-Sue, N Rajpal, P Keane, K Walsh, L Mcgeoghan, M Neeson, H Brown, S M Adams, S Ladha, A Walsh, M Alradhawi, R Tarighi, M I Miah, K Dawas, A Mojadady, A Priestman-Degano, S K Vellore Sasikumar, B Suresh, M Fornasiero, D Csvila, A Kumar, S T Adil, H S Adil, U Kataria, R Jaibaji, N Patel, A Goch, P Quaye, A Gupte, H Mustafa, V Otti, D Dewantoro, M Ali, M Sood, H Wright, L Abusheba, L Wong, M Docksey, A Abdinasir, M O Karim, V Kolaityte, M Vasileiadou Pelling, A Abdel Basit, E Pearson, E Hughes, A S Millington, L Taylor, C Borg, K Jagic, A Waheed, K Singh, V James, T Chowdhury, A Saxena, A Georgiou, T Jones, C Carpenter, I Hughes, Z Aloul, C Hanna-Davies, A Jacob, A Puthiyakunnel Saji, A Qureshi, Z Ulfin, M Dunlop, R Woods, N Hill, N Mohamed, A Vora, H Asharaf, E Maye, U Arif, S Srinivasan, S Prasad, B John, V Shivanand, A Singh, A Zahid, E Stewart, M Raketla, O Kokoricha, D Karwa, J Crisp, R Shehadeh, J Lee, B Webb, S Lepping, F Yusuf, E Pak, B Soo, P Pemmasani, S Goyal, A B Binti Azad Bashir, S Komolafe, A Naeem, H R Anbananthan, L K Au Yong, S Mookerjee, N Ward, M Aniq, J Davis, A Al-Sukaini, J Anthony, J Boyle, A Laird, D Speake, C Beagan, S Stewart, O El-Koubani, R Chan, M Viswanath, S Janssens, I Shah, V Nguyen, E Mckee, C W Ng, M Balać, D I Suresh Kumar, S Seeva Balan, R Kirk, B Miles, L A Kovacic, V G Collins, J Low, W Sim, S J Chua, N Narayanan, T Y Lee, C M N Lo, M Chauhan, J Elkafsi, S Rasul, R Yammine, Z Sattar, D Dixit, N Rahman, J Davies, S Dindyal, L Osborne, L Nip, N Wilding de Miranda, A Stevens, B Al-Diri, A Lim, G Kallikas, Y Kim, M Anjum, P Jeyapahan, M Hakim, S Singh, E Jamileh, C Sohrabi, S H Baek, N Sadik, A Mohammed, D Aje, R Clifford, C O’halloran, K Mahajan, N Darke, S Lloyd, M Mlotshwa, K Melhuish, J Derex-Briggs, M Bell, P Varma, K Fray, Y Garg, K Datta, T Mantel-Cooper, M Goodfellow, B Kazi, N Sievers, K Telford, Z R Almansoor, K Craddock, J L Tan, L St John, A Singhania, S Dosani, S Mughal, N Bokhari, L Brooks, I Laid, A Lala, C S Ong, S Wakefield, S Phillips, H Unwin

**Affiliations:** Department of Applied Health Sciences, School of Health Sciences, College of Medicine and Health, University of Birmingham, Birmingham, UK; Department of Health and Life Sciences, University of Liverpool, Liverpool, UK

**Keywords:** surgical systems, outcomes, healthcare quality systems, quality, surgery

## Abstract

**Background:**

Anaemia is a common modifiable risk factor in surgery, yet how it is recognized and managed in women across different surgical pathways is unknown. This study aimed to evaluate anaemia prevalence in women and its management across surgical pathways and life stages, examine associations with 30-day outcomes, and estimate the potential benefit of anaemia correction.

**Methods:**

A secondary analysis was conducted of a prospective cohort study from 427 hospitals in 29 countries, including women having elective major abdominal surgery. The primary exposure was surgical pathway (gynaecology *versus* non-gynaecology), used as a pragmatic proxy for pathway design. Preoperative anaemia and perioperative iron therapy were conceptualized as mediators; menopausal status (pre- *versus* postmenopausal, by age) was a prespecified effect modifier. Coprimary outcomes were 30-day unplanned readmission and days alive and out of the hospital 30 days from surgery (DAOH_30_). Mixed-effects logistic regression was used with a hospital random intercept for readmission, and quantile regression for DAOH_30_. A 2000-iteration cluster bootstrap estimated absolute risk reduction and number needed to treat (NNT = 1/absolute risk difference) for counterfactual anaemia correction in key subgroups. Natural-effects models assessed whether recorded perioperative iron therapy partly explained the pathway differences in readmission.

**Results:**

A total of 10 114 women (median age 57.6 years (interquartile range 48.0–69.0)) were included; 3590 (35.5%) were premenopausal and 3214 (31.8%) underwent gynaecological surgery. Preoperative anaemia was present in 29.0% and was more frequent in non-gynaecology than gynaecology (31.9% *versus* 22.8%). Thirty-day unplanned readmission occurred in 7.2%. In adjusted models, the gynaecology pathway had lower odds of readmission than non-gynaecology (adjusted odds ratio 0.78, 95% confidence interval 0.62 to 0.97), whereas preoperative anaemia was associated with higher readmission (adjusted odds ratio 1.28, 1.06 to 1.56). Lower perioperative iron use in non-gynaecology partly mediated the pathway gap (indirect effect odds ratio 1.06, 1.02 to 1.09; proportion mediated 15.2%). The largest model-based absolute risk difference was observed among women of premenopausal age undergoing non-gynaecological surgery: predicted readmission was 11.8% with anaemia *versus* 6.4% without anaemia, corresponding to an absolute risk difference of 5.32% and an NNT estimand of 19. Median DAOH_30_ was 26 days (interquartile range 23–28). Anaemia was associated with fewer days at home, with the largest decrement among those with poorest recovery (10th percentile β −3.0 days, 95% confidence interval −4.0 to −2.0), a smaller reduction at the median (β −1.0 days, −1.3 to −0.72), and no difference at the 90th percentile.

**Conclusion:**

For women, pathway design, not only patient risk, shapes whether anaemia is recognized and treated. The greatest avoidable harm falls on premenopausal women outside gynaecology. Health systems should embed universal screening and optimization, adopt standardized iron protocols across all pathways, and track life-stage-stratified performance metrics to close this gap and increase days at home.

## Introduction

Anaemia is a leading cause of disability in women. It affects over a third of those of reproductive age and remains common after menopause^1^. In surgical patients, anaemia is associated with more complications, more transfusions, longer hospital stays, and higher mortality^2,3^. Even in non-surgical patients, anaemia can impact on clinical outcomes in chronic long-term conditions, such as atrial fibrillation, where higher incidences of major bleeding, heart failure hospitalization, and mortality have been shown in association with anaemia severity^4^. Notably, the causes of anaemia differ by life stage: menstrual blood loss and iron deficiency before menopause; chronic disease, nutritional deficits, and occult bleeding after menopause^5,6^. However, in all groups, anaemia is modifiable with interventions such as oral or intravenous iron, which can improve haemoglobin levels, reduce transfusion requirements, and enhance postoperative recovery^7,8^.

Despite clear guidance, perioperative anaemia care is uneven. Patient blood management (PBM) guidelines^3^ recommend universal preoperative screening and optimization before major surgery, yet delivery varies across services. Gynaecological surgical pathways, anchored in women’s health, may include routine haemoglobin testing, iron supplementation protocols, and continuation of therapy after surgery. In contrast, non-gynaecological pathways may lack such integration, with screening and treatment delivered inconsistently or too late to influence outcomes. The result is a structural gap in pathway design: access to evidence-based anaemia care may depend more on the pathway a woman enters than on her clinical need. Whereas sex differences in surgical outcomes are well described^9–11^, far less is known about within-women inequities across specialties and life stages. Existing research and knowledge are based on single specialties such as obstetrics, gynaecologic oncology, or cardiac and rarely consider life stage.

This study addresses a critical evidence gap: no previous large-scale analysis has compared anaemia prevalence, management, and outcomes in women across gynaecological and non-gynaecological pathways, stratified by menopausal status, nor quantified the absolute benefit of anaemia correction in key subgroups. The aim was therefore to compare the burden of anaemia and its management by life stage and pathway among women having major abdominal surgery; examine associations with two coprimary outcomes, 30-day unplanned readmission (clinical destabilization) and days alive and out of hospital up to 30 days from surgery (DAOH_30_; time at home); and estimate absolute risk reduction and number needed to treat (NNT) associated with correcting anaemia in high-yield subgroups. Addressing this gap will evaluate whether disparities are structural and modifiable, and whether targeted optimization can reduce readmissions and increase time at home.

## Methods

### Data source, study design and population

A secondary analysis was performed of the CASCADE cohort (CArdiovaSCulAr outcomes after major abDominal surgEry)^12^, a prospective study of postoperative outcomes after major abdominal surgery conducted across 427 hospitals in 29 European countries. This secondary analysis was prespecified in the main study protocol^12^. CASCADE enrolled consecutive adults (≥ 18 years) undergoing elective or emergency major abdominal surgery, defined as abdominal or pelvic visceral resection, open vascular, or transplant surgery across five surgical specialties and any operative approach. Exclusions were trauma, day-case procedures, and operations without visceral resection. Data were collected prospectively over five predefined 2-week periods between 24 January and 3April 2022, with 30-day follow-up. Ethical approval was granted by the NHS South East Scotland Research Ethics Committee (NR/161AB6), with exemption from individual consent owing to use of anonymized routine data; local approvals were obtained per national regulations. A summary of the country-level approvals are provided in *Appendix S1*. A detailed description of the CASCADE cohort study is presented in *Appendix S2*. Reporting follows STROBE^13^ guidance *(Appendix S3)*. The CASCADE protocol^12^ and primary results^14^ are published elsewhere. For this analysis, women undergoing elective major abdominal surgery were included; emergency cases were excluded.

### Data collection

A prespecified case report form (CRF) was developed drawing upon templates successfully used in previous multinational cohort studies conducted within the STARSurg global collaborative research network^15,16^. The CRF was developed in collaboration with a Study Management Group comprising surgical, perioperative, and geriatric care experts. Several iterative rounds of expert review and refinement ensured that the CRF captured relevant patient- and operative-level data aligned with the preplanned primary and secondary analyses. This process also ensured the content validity of the CRF, as it was informed by existing literature and clinical expertise. At each participating hospital, clinical data were prospectively collected and entered into the CRF by local research teams, which consisted primarily of trainee doctors and consultants. All collaborators received online training on data definitions, and additional training or troubleshooting was available from national and study leads. The CRF was designed for use by clinicians to record routinely collected clinical data on all eligible patients. A standardized 30-day follow-up was conducted in person, via telephone or through chart review, using structured protocols. The CRF was translated into local languages by national study leads to ensure contextual appropriateness. Although formal psychometric validation of the CRF was not undertaken, translations were based on previously validated templates used in multinational studies conducted by the research network. To ensure data integrity further, the study leveraged the well established research infrastructure of the STARSurg global network. This included centralized data storage using the Research Electronic Data Capture^17^ web application hosted at the University of Birmingham (UK), which facilitated secure and standardized data management. Central monitoring of data completeness and logic was performed throughout the study, and only data collection periods achieving > 95% completeness were accepted for inclusion in the pooled analysis. However, no data validation was performed on ascertainment and accuracy, as previous studies conducted by the research network have highlighted robust data quality. Based on previous studies^18,19^, data ascertainment and accuracy are as high as 96%.

### Exposures

The primary exposure was surgical pathway, defined by operating specialty (gynaecology *versus* non-gynaecology). Surgical pathway was treated as a pragmatic bundle exposure, capturing pathway organization alongside differences in case mix, procedure type, operative complexity, and perioperative infrastructure, rather than as a direct measure of any single pathway component. This was treated as a proxy for perioperative pathway design, reflecting systematic differences in PBM. Gynaecological services more often embed routine haemoglobin (± ferritin) testing, explicit treatment thresholds, and direct access to iron therapy^20–22^, whereas non-gynaecological services show later and more heterogeneous PBM adoption, with anaemia assessment variably devolved to general medicine or primary care^3,23^. Treating specialty as a pathway proxy therefore captures organizational design and protocolization differences plausibly upstream of screening and treatment delivery, beyond case mix. Perioperative anaemia and its management were conceptualized as mediators on the causal pathway between surgical specialty and outcomes. Preoperative anaemia was defined by World Health Organization^24^ criteria for non-pregnant women (haemoglobin < 120 g/l). The preoperative haemoglobin was the last value recorded before incision, typically at preassessment or on admission. For descriptive analyses, postoperative nadir haemoglobin (lowest inpatient value) and discharge haemoglobin (last inpatient value before discharge) were also defined. Perioperative iron therapy (oral or intravenous) was included as the principal treatment mediator, representing a direct, modifiable mechanism through which pathway design could alter outcomes. Menopausal status was not directly recorded in CASCADE. Age was therefore used as a pragmatic proxy for life stage, defining women aged < 51 years as being of premenopausal age and women aged ≥ 51 years as being of postmenopausal age. This threshold was selected to approximate the median age at natural menopause^25,26^. These cut-offs reflect median age at natural menopause in high-income settings. All pathway models were adjusted for case mix and included a random intercept for hospital. Prespecified covariates included frailty, American Society of Anesthiologists (ASA) grade, multimorbidity (≥ 2 long-term conditions)^27^, operative indication (benign *versus* malignant), and approach (open *versus* minimally invasive).

### Study outcomes

Two coprimary outcomes were prespecified to capture complementary dimensions of recovery and health-system use: 30-day unplanned readmission and DAOH_30_. Unplanned readmission was any unanticipated inpatient admission (index or non-index hospital) after discharge and within 30 days of the index operation. Planned or staged readmissions (for example chemotherapy, planned stoma reversal), interhospital transfers during the index admission, and emergency department attendances without admission were excluded. Ascertainment used hospital records and structured patient contact. DAOH_30_was calculated as 30 minus total inpatient days (index stay plus all readmissions) within 30 days. A minimum of 1 inpatient day was counted for the index admission. Patients who died during the index admission or remained hospitalized for the full 30-day window were assigned DAOH_30_ = 0. For patients discharged who died within 30 days, DAOH_30_ equalled days alive after discharge. A detailed description of different scenarios to calculate DAOH_30_ is presented in *Fig. S1*. Readmission provides a system-level signal of adverse postoperative course, and DAOH_30_ captures patient-centred recovery. DAOH_30_ is a patient-centred metric^28^ integrating survival, length of stay, and readmissions into a single interpretable measure^29^; it is routinely derivable from administrative data and facilitates robust comparisons across hospitals and pathways^29^. Using both outcomes minimizes reliance on any single proxy for recovery, improves interpretability across surgical pathways, and directly serves the study objective of identifying pathway-driven, modifiable differences in perioperative care for women.

### Sample size

As a secondary analysis, a precision check against expected readmission differences was conducted. Using published estimates^30^ (12.3% with anaemia *versus* 6.0% without anaemia; absolute difference 6.3% points), 3240 women per group would provide 80% power (two-sided α = 0.05). Allowing 10% missingness, approximately 7200 women would undergo surgery. The cohort of 10 114 women undergoing surgery provides ample precision for subgroup and interaction estimates.

### Statistical analysis

Baseline characteristics are reported as median (interquartile range (i.q.r.)) or counts (%). Between-group comparisons used Mann-Whitney *U* and χ^2^ tests. All tests were two-sided (α = 0.05). Primary inferential analyses used complete cases for the relevant model variables. Missingness was low for the main exposure and coprimary outcomes, but was higher for some descriptive variables, including postoperative nadir haemoglobin, discharge haemoglobin, and smoking status. Postoperative and discharge haemoglobin were therefore interpreted descriptively and were not used as core adjustment variables in the primary readmission model. Effect sizes and 95% confidence intervals (c.i.) were emphasized; no multiplicity adjustment was applied to secondary or exploratory analyses. Analyses used R 4.2 (R Foundation for Statistical Computing, Vienna, Austria) using the packages *finalfit, mgcv, tidyverse,* and *quantreg.*

For the outcome of 30-day unplanned readmission, multilevel logistic regression with a random intercept for hospital to account for clustering was used. A hospital-level random intercept was included because patients were clustered within hospitals and were therefore unlikely to represent statistically independent observations. This term allowed baseline readmission risk to vary between hospitals, accounting for shared institutional factors such as perioperative protocols, discharge thresholds, readmission practices, and access to anaemia optimization services. The resulting odds ratios (ORs) should therefore be interpreted as hospital-conditional associations, estimated after accounting for between-hospital differences in baseline readmission risk. Fixed effects included multimorbidity, ASA grade, frailty, surgical approach, and operative indication. Results are presented as adjusted ORs with 95% c.i. Model assumptions were assessed including collinearity, calibration, discrimination, and random-effects variance. To evaluate whether effects differed by life stage and anaemia status, a generalized additive model with a prespecified three-way interaction (pathway × preoperative anaemia × menopausal status) was fitted. Marginal predicted risks under counterfactual anaemia states were estimated by g-computation, from which absolute risk differences were derived. An NNT-type estimand as the inverse of the absolute risk difference was also calculated. Because this was an observational analysis, this estimate should not be interpreted as a trial-derived clinical NNT or a direct treatment target. Rather, it represents a model-based counterfactual estimand reliant on standard causal assumptions, including no residual confounding, correct model specification, consistency, and adequate overlap between comparison groups. Its purpose was to aid interpretation of absolute risk differences and identify subgroups with the greatest potential yield from anaemia optimization. Uncertainty was quantified with 2000 hospital-cluster bootstrap replicates. Whether perioperative iron therapy explained part of the pathway–readmission association was tested. Iron therapy was selected as the mediator because it represents a direct, modifiable mechanism through which pathway design could alter outcomes. A natural-effects modelling approach with two logistic models (outcome and mediator) was applied^31^. Estimates included natural direct and indirect effects, and the proportion mediated. Identification assumptions were prespecified (no unmeasured confounding of exposure-outcome, exposure-mediator, or mediator-outcome; no mediator-outcome confounder affected by exposure).

### Sensitivity analysis by operative specialty

To address heterogeneity within the non-gynaecology group, two sensitivity analyses were performed. First, anaemia burden, estimated blood loss, perioperative iron therapy, and 30-day unplanned readmission were summarized by granular operative specialty. Vascular and transplant surgery were combined because of small numbers. Second, because gastrointestinal and liver surgery was the largest non-gynaecological subgroup, the counterfactual interaction analysis was repeated restricted to women undergoing gastrointestinal and liver surgery, using the same modelling approach as the primary analysis. Predicted probabilities of unplanned readmission were estimated under anaemic and non-anaemic states, stratified by menopausal status, with absolute risk differences and numbers needed to treat derived from 2000 bootstrap replicates.

For DAOH_30_, quantile regression was used due to the skewed, non-normal, and zero-inflated distribution of this outcome^32,33^. This approach estimates effects across the outcome distribution rather than at the mean, providing insight into heterogeneity of recovery^34^. Models were adjusted for the same covariates as above. Effect estimates are reported at the following percentiles: 5th (patients with poorest recovery) 10th, 25th, 50th (median), 75th and the 90th percentile (patients with the best recovery). Results are expressed as β coefficients with 95% c.i.

### Role of the funding source

The sponsor of the study had no role in study design, data collection, data analysis, data interpretation, or writing of the report. The corresponding author had full access to all the data in the study and had final responsibility for the decision to submit for publication.

## Results

### Cohort characteristics

A total of 10 114 women undergoing elective major abdominal surgery from 427 hospitals in 29 European countries were included (*Fig. 1*). Of these, 3214 (31.8%) underwent gynaecology surgery and 6900 (68.2%) underwent non-gynaecology surgery. Median age was 52.0 years (i.q.r. 44.0-62.0) in women undergoing gynaecology surgery and 62.0 years (i.q.r. 49.0–72.0) undergoing non-gynaecology surgery. Compared with women undergoing non-gynaecology surgery, women undergoing gynaecology surgery were more likely to be younger and consequentially premenopausal (48.8% *versus* 29.3%; *P* < 0.001), had lower ASA physical status (ASA I–II 82.5% *versus* 62.8%; *P* < 0.001), less multimorbidity (25.4% *versus* 40.8%), and lower frailty (frailty 4-9: 8.5% *versus* 15.6%). Most procedures were for benign indications (59.7%), and 61.0% used minimally invasive approaches (*Table 1*).

**Figure 1:**
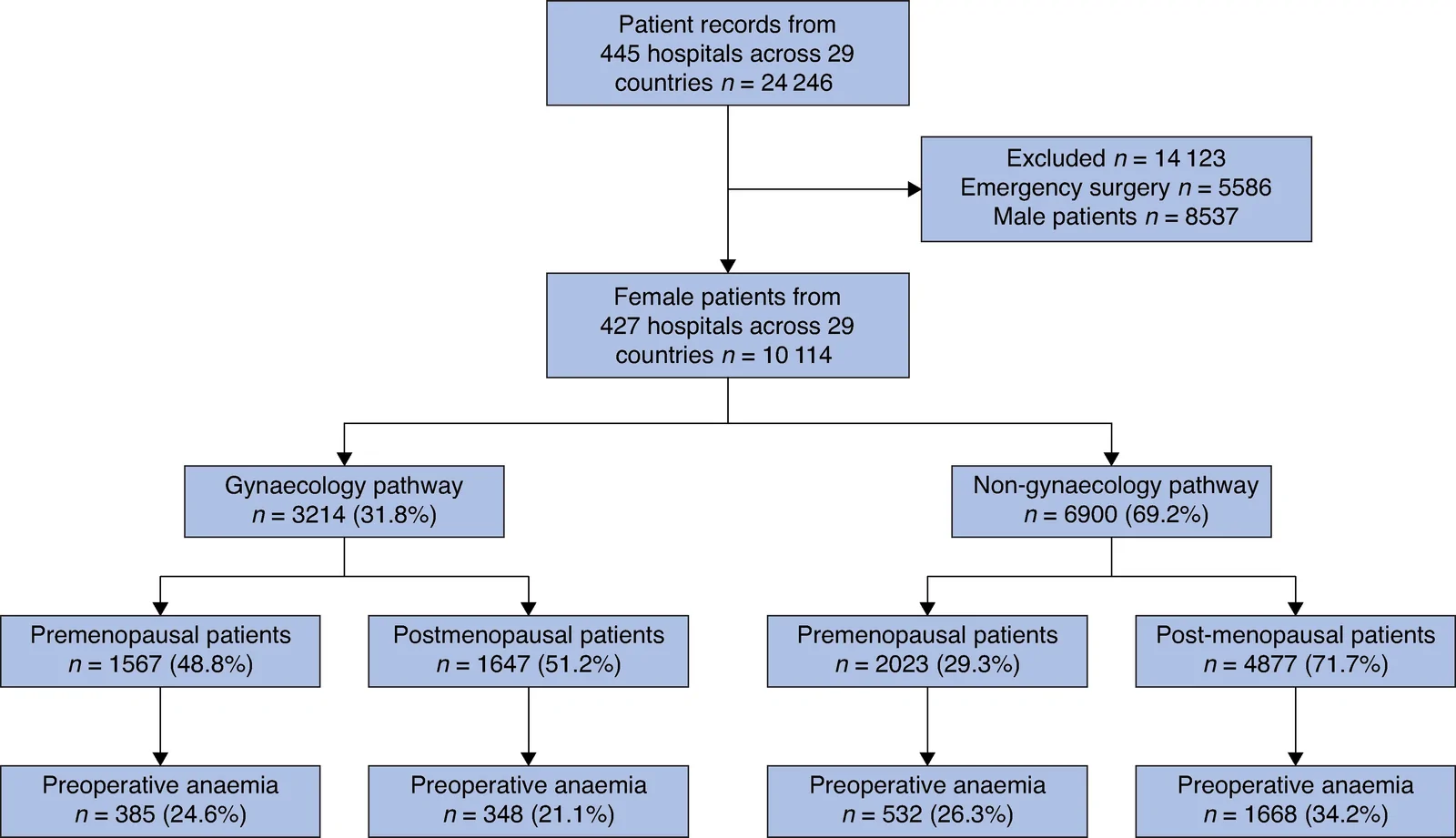
Study flowchart

**Table 1:** Baseline patient- and operative-level characteristics of women undergoing elective abdominal surgery by surgical pathways

	Gynaecology (*n* = 3214; 31.8%)	Non-gynaecology (*n* = 6900; 68.2%)	Total (*n* = 10 114)	P*
Age (years), median (i.q.r.)	52.0 (44.0–62.0)	62.0 (49.0–72.0)	58.0 (47.0–69.0)	< 0.001
**Menopause status**				
Premenopausal	1567 (48.8%)	2023 (29.3%)	3590 (35.5%)	< 0.001
Postmenopausal	1647 (51.2%)	4877 (70.7%)	6524 (64.5%)	
(Missing)	0 (0.0%)	0 (0.0%)	0 (0.0%)	
**ASA physical status**				
I–II	2651 (82.5%)	4678 (67.8%)	7329 (72.5%)	< 0.001
III–V	494 (15.4%)	2080 (30.1%)	2574 (25.4%)	
(Missing)	69 (2.1%)	142 (2.1%)	211 (2.1%)	
**Smoking status**				
Never smoked	1809 (56.3%)	3985 (57.8%)	5794 (57.3%)	0.010
Current	528 (16.4%)	992 (14.4%)	1520 (15.0%)	
Ex-smoker (< 6 weeks)	27 (0.8%)	70 (1.0%)	97 (1.0%)	
Ex-smoker (6 weeks–1 year)	39 (1.2%)	115 (1.7%)	154 (1.5%)	
Ex-smoker (> 1 year)	368 (11.4%)	889 (12.9%)	1257 (12.4%)	
(Missing)	443 (13.8%)	849 (12.3%)	1292 (12.8%)	
**Clinical frailty**				
1–3	2558 (91.5%)	5342 (84.3%)	7900 (86.5%)	< 0.001
4–6	225 (8.1%)	952 (15.0%)	1177 (12.9%)	
7–9	12 (0.4%)	41 (0.6%)	53 (0.6%)	
**Number of comorbidities**				
None	1407 (43.8%)	1805 (26.2%)	3212 (31.8%)	< 0.001
One	984 (30.6%)	2279 (33.0%)	3263 (32.3%)	
Two	515 (16.0%)	1594 (23.1%)	2109 (20.9%)	
Three	223 (6.9%)	756 (11.0%)	979 (9.7%)	
Four or more	81 (2.5%)	462 (6.7%)	543 (5.4%)	
(Missing)	4 (0.1%)	4 (0.1%)	8 (0.1%)	
**Indication**				
Benign	2095 (65.2%)	3945 (57.2%)	6040 (59.7%)	< 0.001
Malignant	1118 (34.8%)	2946 (42.7%)	4064 (40.2%)	
(Missing)	1 (0.0%)	9 (0.1%)	10 (0.1%)	
**Operative approach**				
Minimally-invasive	1835 (57.1%)	4330 (62.8%)	6165 (61.0%)	< 0.001
Open	1378 (42.9%)	2570 (37.2%)	3948 (39.0%)	
(Missing)	1 (0.0%)	0 (0.0%)	1 (0.0%)	
**Preoperative anaemia**				
No	2328 (72.4%)	4474 (64.8%)	6802 (67.3%)	< 0.001
Yes	733 (22.8%)	2200 (31.9%)	2933 (29.0%)	
(Missing)	153 (4.8%)	226 (3.3%)	379 (3.7%)	
**Postoperative anaemia (at nadir)**				
No	690 (21.5%)	1217 (17.6%)	1907 (18.9%)	< 0.001
Yes	1538 (47.9%)	3961 (57.4%)	5499 (54.4%)	
(Missing)	986 (30.7%)	1722 (25.0%)	2708 (26.8%)	
**Postoperative anaemia (at discharge)**				
No	640 (19.9%)	1277 (18.5%)	1917 (19.0%)	< 0.001
Yes	1230 (38.3%)	3149 (45.6%)	4379 (43.3%)	
(Missing)	1344 (41.8%)	2474 (35.9%)	3818 (37.7%)	
**Preoperative anaemia iron treatment**				
No	2946 (91.7%)	6349 (92.0%)	9295 (91.9%)	0.570
Yes	268 (8.3%)	551 (8.0%)	819 (8.1%)	
(Missing)	0 (0.0%)	0 (0.0%)	0 (0.0%)	
**Postoperative anaemia iron treatment**				
No	2836 (88.2%)	6446 (93.4%)	9282 (91.8%)	< 0.001
Yes	378 (11.8%)	454 (6.6%)	832 (8.2%)	
(Missing)	0 (0.0%)	0 (0.0%)	0 (0.0%)	
**Postoperative transfusion**				
No	3122 (97.1%)	6435 (93.3%)	9557 (94.5%)	< 0.001
Yes	92 (2.9%)	465 (6.7%)	557 (5.5%)	
(Missing)	0 (0.0%)	0 (0.0%)	0 (0.0%)	

Values are *n* (%) unless otherwise stated. i.q.r., interquartile range; ASA, American Society of Anesthiologists. *

### Preoperative anaemia and management

Preoperative anaemia was less common in gynaecological than non-gynaecological surgery (22.8% *versus* 31.9%; *P* < 0.001). After surgery, anaemia remained less common in patients undergoing gynaecological than non-gynaecological surgery at haemoglobin nadir (47.9% *versus* 57.4%; *P* < 0.001) and at discharge (38.0% *versus* 43.5%; *P* < 0.001). Although preoperative iron treatment was similar across the pathways, postoperative iron therapy use remained higher in gynaecological than non-gynaecological pathways (11.8% *versus* 6.6%; *P* < 0.001) (*Table 1*). Differences in baseline characteristics by preoperative anaemia status is presented in *Table S1*.

### Unplanned 30-day readmission

Overall, 724 of 10 114 (7.2%) women were readmitted within 30 days. Readmission was more frequent in non-gynaecology than gynaecology pathways (7.5% *versus* 6.3%; *P* = 0.030), in women with anaemia than those without (9.2% *versus* 6.4%; *P* < 0.001) and in premenopausal than postmenopausal women (8.6% *versus* 6.4%; *P* < 0.001). Differences in patient- and operative-level characteristics by unplanned 30-day readmission are presented in *Table S2*. In a multilevel logistic regression, women undergoing surgery in gynaecological pathways had lower odds than those in non-gynaecological (OR 0.78, 95% c.i. 0.62 to 0.97) for 30-day readmission. However, preoperative anaemia was associated with higher 30-day unplanned readmission (OR 1.28, 1.06 to 1.56) (*Fig. 2*). Women of postmenopausal age were associated with lower odds of 30-day unplanned readmission (OR 0.77, 0.62 to 0.95) than those of premenopausal age. The full model is presented in *Table S3*.

**Figure 2:**
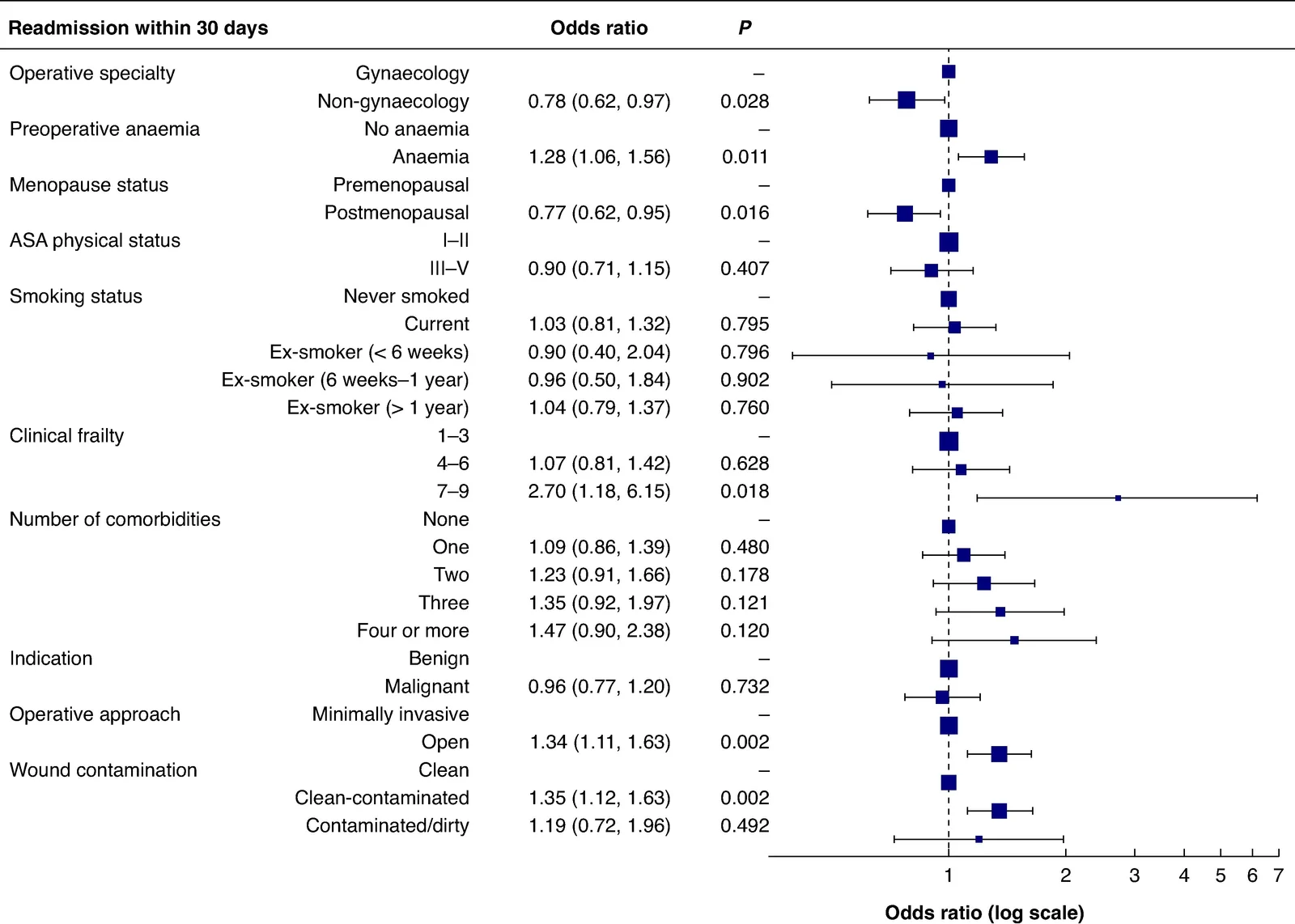
Multilevel logistic regression of patient, and operative characteristics on unplanned readmission to hospital within 30 days from surgery in patients undergoing elective abdominal surgery. Multilevel model includes hospital and country identifier as a random effect.

To understand the influence between surgical pathways (exposure) and perioperative anaemia treatment (mediator) on 30-day unplanned readmission, a mediation analysis was performed. A total of 15.2% of the pathway difference in 30-day unplanned readmission was mediated by lower perioperative iron therapy use in non-gynaecology (natural indirect effect OR 1.06, 95% c.i. 1.02 to 1.09; *P* = 0.001) (*Fig. S2*). The remaining pathway association was not explained by perioperative iron therapy, indicating that iron treatment captured only a modest, measurable component of the pathway difference in readmission, with other pathway-level mechanisms likely contributing. Interactions between menopausal status, pathway, and anaemia were explored using a generalized additive model with cluster bootstrap. Absence of preoperative anaemia was associated with a lower predicted probability of readmission across strata (*Fig. 3a*). The largest absolute difference was for premenopausal women in non-gynaecological surgery: predicted readmission 11.8% with anaemia *versus* 6.4% without anaemia; absolute risk reduction 5.32% (95% c.i. 3.59 to 7.36; *P* < 0.001), corresponding to a NNT of 19 (18.8, 95% c.i. 13.6 to 27.9) to prevent one readmission by correcting anaemia (*Fig. 3b*; *Tables S4*, *S5*).

**Figure 3:**
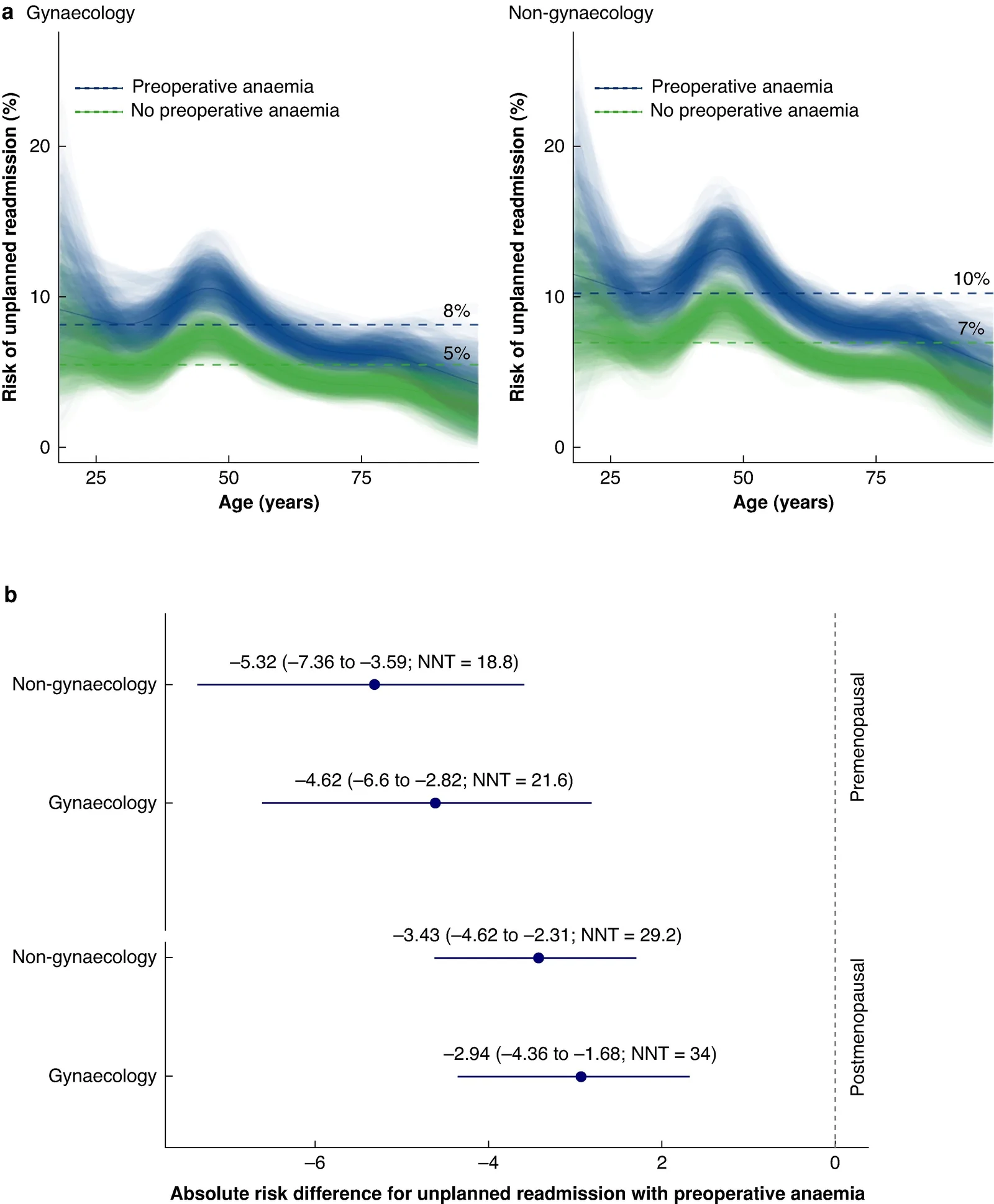
Relationship between anaemia, menopausal status, surgical pathway on unplanned readmission. **a** Generalized additive model with each line representing a bootstrap (*n* = 100). **b** Absolute risk difference between presence and absence of preoperative anaemia for unplanned readmission. Bootstrap replications of the interaction models (*n* = 2000) were used to compute conditional probabilities and absolute risk differences were calculated. Number-needed-to-treat (NNT) is shown for each subgroup and calculated as 1/absolute risk difference.

### Sensitivity analysis by operative specialty

Specialty-stratified sensitivity analysis confirmed heterogeneity within the non-gynaecology group. Gastrointestinal and liver surgery was the largest non-gynaecological subgroup (6208), with preoperative anaemia in 1950 of 5995 women (32.5%), preoperative iron therapy in 495 of 6208 (8.0%), postoperative iron therapy in 396 of 6208 (6.4%), and 30-day unplanned readmission in 464 of 6208 (7.5%) (*Table S7*). By comparison, gynaecological surgery had lower preoperative anaemia prevalence (733 of 3061, 23.9%) and similar preoperative iron therapy use (268 of 3214, 8.3%), but higher postoperative iron therapy use (378 of 3214, 11.8%). In the gastrointestinal and liver surgery-only counterfactual analysis, the findings were consistent with the main non-gynaecology analysis (*Table S5*).

### Days alive and out of hospital at 30-days

Median DAOH_30_ was 26 days (i.q.r. 23–28). Premenopausal women had more days alive and out of hospital than postmenopausal women (median 27 (i.q.r. 24–29) *versus* 25 (22–27); *P* < 0.001). Patients with anaemia had fewer days alive and out of hospital than patients without anaemia (*Fig. 4a*). In multivariable quantile regression, the association between gynaecological pathways and lower DAOH_30_ was greatest across all percentiles from the 5th percentile (β −4.3 days, 95% c.i. −5.1 to −3.5; *P* < 0.001), and to the 90th percentile (β −1.0 days, −1.9 to −0.1; *P* = 0.030) (*Fig. 4b*). The association between preoperative anaemia and lower DAOH_30_ was greatest at the 5th and 10th percentiles (β −3.0 days, −4.0 to −2.0; *P* < 0.001), smaller at the median (β −1.0 days, −1.3 to −0.72; *P* < 0.001), and absent at the 90th percentile (β 0.00 days, −0.85 to 0.85; *P* > 0.900). However, women of postmenopausal age were not associated with DAOH_30_ across all percentiles (*Fig. 4d*). The detailed model of the quantile regression is presented in *Table S6*.

**Figure 4:**
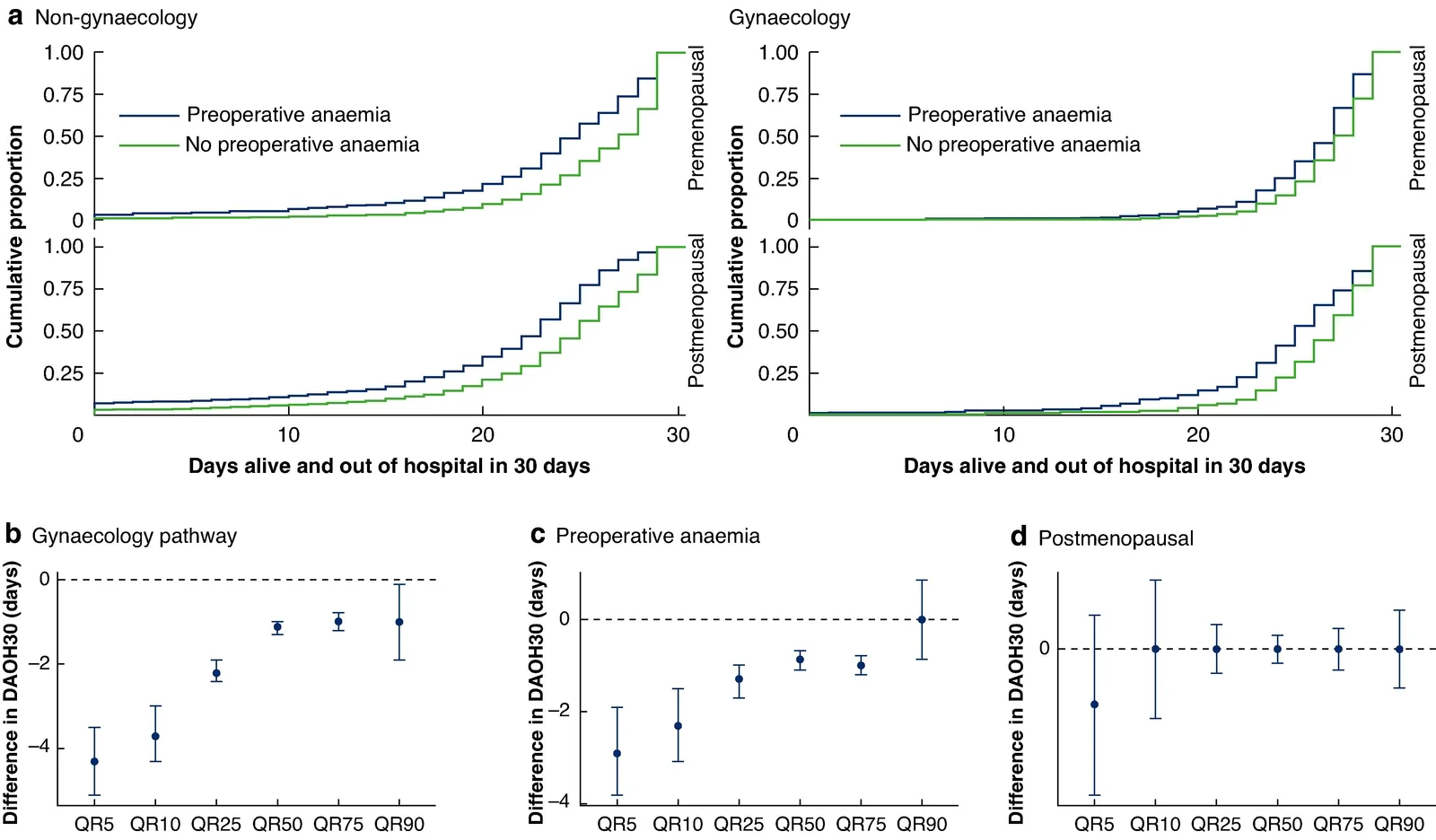
Impact of preoperative anaemia, menopause status and surgical pathway on days alive and out of hospital at 30 days (DAOH30) **a** Cumulative distribution curves of DAOH30 by preoperative anaemia status, stratified by menopausal status and surgical specialty. Multivariable quantile regression estimates at the 5th, 10th, 25th, 50th, 75th and 90th percentiles, adjusted for key patient- and operative-level characteristics for **b** gynaecological pathways; **c** pre-operative anaemia; and **d** women of post-menopausal age.

Although gynaecological pathways were associated with lower odds of 30-day unplanned readmission, they were also associated with fewer days alive and out of hospital across the DAOH_30_ distribution. These findings should not be interpreted as contradictory, as the two outcomes capture different aspects of recovery. Readmission reflects postdischarge clinical destabilization, whereas DAOH_30_ is strongly influenced by the duration of the index admission, postoperative monitoring, discharge policy, and procedure mix. Therefore, lower readmission can coexist with lower DAOH_30_ where inpatient stays are longer or discharge thresholds differ across pathways.

## Discussion

In this study, across 10 114 women in 427 hospitals in 29 European countries, the principal findings are as follows: perioperative anaemia care systematically varies by surgical pathway; preoperative anaemia was common, especially outside gynaecological surgical pathways, and associated with poor 30-day outcomes; and gynaecological pathways used iron therapy more consistently, whilst non-gynaecological pathways treated less despite higher need, and part of their higher readmission risk was explained by lower use of perioperative iron therapy.

To the authors’ knowledge, no studies have evaluated perioperative anaemia across surgical pathways that differ in their alignment with women’s health priorities, quantified differences in treatment, or assessed the consequences for outcomes across menopausal status. The largest, most actionable gain remains with premenopausal women in non-gynaecological surgery, where correcting anaemia could reduce readmissions by about 5.3% (NNT of 19). Findings from this study emphasize the need for health systems to embed anaemia optimization as a default process in every pathway, track it with simple metrics, and close the gap for higher-risk groups.

Existing research has examined mixed-sex populations^2,35^, where sex-specific and life stage-specific analyses are absent, or have been restricted to a single specialty such as obstetrics or gynaecology^3,36,37^. The adverse association between preoperative anaemia and surgical outcomes is well established, with meta-analyses linking anaemia to increased complications, longer hospitalization, and greater mortality^2,36,38,39^. In contrast, the present analyses compares gynaecological *versus* non-gynaecological pathways, stratifies by life stage, quantifies absolute benefit, and tests a plausible mechanism, lower perioperative iron use, behind pathway differences in readmission. The present data suggest that for women having major abdominal surgery, whether anaemia is recognized and treated depends as much on how care is organized as on clinical risk. The two coprimary outcomes point to complementary truths. Readmission signals clinical destabilization and service failure; DAOH_30_ captures time at home, integrating survival, length of stay, and readmissions. Anaemia raised readmission risk and reduced DAOH_30_, with the largest shortfall among women with poorest recovery. These patterns support PBM as a system priority, not an optional add-on.

A central finding is that perioperative anaemia care and its association with postoperative outcomes differ across heterogeneous surgical pathways. Surgical pathways should therefore be interpreted as a bundle exposure. The observed associations may reflect differences in anaemia-management infrastructure, but also residual differences in case mix, procedure type, operative complexity, blood loss, discharge practices, and other specialty-specific processes. Accordingly, these findings suggest pathway-level variation in anaemia care and recovery, but do not prove that pathway design alone caused the observed differences. Gynaecological and non-gynaecological surgeries represent distinct perioperative care models, with different degrees of integration of women’s health priorities, PBM protocols, and access to timely iron therapy. These structural differences translate into heterogeneous risk profiles and modifiability of outcomes.

Although preoperative anaemia was consistently associated with worse outcomes across pathways, its absolute impact on 30-day readmission varied substantially between gynaecological and non-gynaecological surgery, with the largest absolute benefit from correction concentrated among premenopausal women undergoing non-gynaecological procedures. This reflects both higher baseline risk and lower treatment uptake in these pathways, illustrating how the same biological exposure can have different system-level consequences depending on pathway design.

Heterogeneity was also evident in postoperative recovery, as assessed by DAOH_30_. Using quantile regression, anaemia was associated with markedly fewer days at home among women with the poorest recovery, with smaller effects around the median and no clear association among those with the best recovery. This distributional heterogeneity suggests that anaemia disproportionately affects vulnerable recovery trajectories, particularly within less protocolized pathways. Residual heterogeneity is likely across hospitals and countries, reflecting variation in screening thresholds, iron availability and timing, perioperative governance, and discharge or readmission practices. Although multilevel modelling accounted for clustering, such contextual differences may continue to influence both treatment delivery and outcome ascertainment. Residual heterogeneity is also likely across hospitals and countries, reflecting variation in screening thresholds, iron availability, perioperative governance, and discharge or readmission practices. The hospital random intercept accounted for clustering and allowed baseline readmission risk to vary between hospitals, but it did not identify which institutional factors explain this variation. Therefore, the readmission estimates should be interpreted as hospital-conditional associations rather than direct measures of specific hospital-level processes.

Finally, the two coprimary outcomes provided complementary, but not identical, signals. Gynaecological pathways were associated with lower odds of 30-day unplanned readmission, but also with fewer days alive and out of hospital. This distinction is important. Readmission captures postdischarge clinical destabilization and health-system use, whereas DAOH_30_ integrates survival, index length of stay, and readmissions. A lower DAOH_30_ in gynaecology therefore does not necessarily contradict more consistent anaemia management; it may reflect longer planned inpatient recovery, greater representation of gynaecological oncology procedures, pathway-specific postoperative monitoring, or more conservative discharge policies. The findings suggest that anaemia treatment may be more consistently delivered in gynaecology, whereas overall recovery time remains shaped by procedure mix, cancer pathways, inpatient care models, and institutional discharge practices.

The differences observed are likely driven by variation in the design and implementation of perioperative pathways. In gynaecological pathways, high iron-deficiency burden may have driven embedded protocols, which may include routine haemoglobin and/or ferritin testing, clear thresholds, and ready access to perioperative iron treatment. In contrast, non-gynaecological pathways may devolve anaemia to primary care or general medicine, resulting in late or inconsistent screening and treatment. The persistence of differences across menopause suggests a structural cause, not simply variation in aetiology. Lower perioperative iron therapy use in non-gynaecological pathways mediated 15.2% of the pathway difference in 30-day readmission, suggesting that iron treatment is a measurable and modifiable contributor, but not the dominant explanation for the pathway gap. The mediation analysis was consistent with partial mediation by recorded perioperative iron therapy but should be interpreted as hypothesis-generating rather than mechanistic. Receipt of iron therapy may reflect anaemia severity, anaemia aetiology, timing of surgery, clinician decision-making, and local service availability, which may also relate to readmission. The remaining direct association should not be interpreted simply as ‘unmeasured confounding’, but rather as the component not explained by the measured mediator. This may include other pathway-level factors such as timing of anaemia screening, access to ferritin testing, preoperative assessment infrastructure, thresholds for intervention, transfusion practice, postoperative monitoring, discharge and readmission thresholds, institutional resources, surgical complexity, and residual confounding. Therefore, standardizing iron therapy is likely to reduce only part of the disparity and should be embedded within broader perioperative pathway redesign. This mechanistic interpretation is further limited by the absence of ferritin or transferrin saturation data. Perioperative iron therapy should therefore be interpreted as a measurable marker of anaemia-directed care, rather than proof that the observed pathway association was mediated specifically through correction of iron-deficiency anaemia. Further, the higher prevalence of anaemia in the premenopausal population may be linked to the high prevalence of unrecognized and untreated menstrual disorders in this group, representing a silent burden. A further caveat is that anaemia is generally associated with poor overall health and adverse socioeconomic indices.

These findings translate into clear priorities for practice and policy. First, the high prevalence of anaemia across all groups, and its disproportionate burden in non-gynaecological surgery, supports the adoption of universal perioperative screening and optimization in all surgical specialties, consistent with international PBM guidelines^20^. Embedding haemoglobin assessments into preoperative workflows and monitoring compliance through quality systems would operationalize this recommendation. Second, the lower use of iron therapy in non-gynaecological pathways despite greater need highlights the importance of standardized treatment protocols, covering both oral and intravenous regimens, with defined thresholds and timelines for intervention^40^. This is to ensure timely preoperative correction and appropriate postoperative continuation^41^. Third, targeted optimization for high-risk groups, particularly premenopausal women in non-gynaecological surgery, could include early identification at surgical listing, fast-track access to intravenous iron, and coordinated follow-up, offering rapid and measurable reductions in readmissions. Finally, pathway-level performance metrics stratified by menopausal status including screening rates, time to treatment, and rates of preoperative optimization should be incorporated into perioperative governance to enable benchmarking, guide resource allocation, and support continuous improvement.

This study has several strengths. These include prospective data, including a large sample size, harmonized definitions, and a focus on women with a life-stage lens. Using both a system outcome (readmission) and a recovery metric (DAOH_30_) increases policy relevance. Multilevel modelling accounted for clustering; interaction modelling and g-computation provided interpretable absolute effects; mediation offered mechanistic insight. Several limitations should be acknowledged. First, as an observational analysis, causality cannot be inferred, and residual confounding from unmeasured factors such as surgical complexity, intraoperative events, institutional resources, and social determinants remains possible. Although the mixed-effects model accounted for hospital clustering, the random intercept captures unmeasured between-hospital variation rather than explaining it. Residual institutional confounding may therefore persist if hospital-level factors, such as anaemia screening pathways, intravenous iron access, discharge policy, or readmission thresholds, are associated with both surgical pathway and outcome. Second, menopausal status was inferred from age, introducing inevitable misclassification. Third, anaemia definitions were standardized using the World Health Organization criteria, but variation in local laboratory thresholds, measurement timing, and treatment triggers across countries may have introduced heterogeneity. Relatedly, because iron treatment may reflect unmeasured anaemia severity, aetiology, clinician judgement, and service availability, these findings should be considered hypothesis-generating rather than definitive evidence of mechanism. Missing data may also have introduced bias. Although missingness was low for the main exposure and coprimary outcomes, postoperative nadir haemoglobin, discharge haemoglobin, and smoking status had more substantial missingness. Missing postoperative haemoglobin values are unlikely to be completely random, as testing may reflect clinical concern, postoperative complications, local monitoring protocols, and discharge practices. These variables were therefore interpreted cautiously, particularly where presented descriptively, and residual bias from non-random missingness cannot be excluded. Fourth, classification of surgery as gynaecological *versus* non-gynaecological was used as a pragmatic marker of pathway design, but inevitably oversimplifies variation in procedure type, expected blood loss, operative complexity, and perioperative infrastructure. Sensitivity analyses were therefore performed using granular operative specialty categories and a focused gastrointestinal and liver surgery-only analysis. These showed heterogeneity across specialties but did not materially alter the overall interpretation. Nevertheless, residual heterogeneity within specialties and between hospitals may persist despite adjustment for operative indication, approach, frailty, multimorbidity, and hospital-level clustering. Fifth, treatment classification lacked detail on timing, dose, formulation, and adherence, and it was not always possible to distinguish optimization from reactive postoperative treatment. Sixth, unplanned readmission, although widely used in quality reporting, is a composite outcome influenced by multiple factors beyond anaemia; reasons for readmission were not always available. Days alive and out of hospital can also be affected by local discharge and readmission policies. Seventh, The NNT estimates should be interpreted cautiously. They were calculated from model-based counterfactual absolute risk differences in an observational data set and therefore depend on strong assumptions, including no residual confounding, consistency, adequate overlap, and correct model specification. They should be viewed as NNT-type estimands useful for comparing the potential yield of optimization across subgroups, rather than as direct clinical treatment targets. Prospective interventional studies are needed to determine whether anaemia correction would achieve these absolute reductions in readmission. Finally, participating hospitals may be more research-active than non-participating centres, potentially underestimating the scale of disparity, and variation in intravenous iron availability across Europe may limit immediate feasibility of universal implementation.

Surgical pathways without embedded anaemia-management processes may under-recognize and undertreat perioperative anaemia, even when clinical need is high. This pathway-level variation is measurable, clinically important, and potentially modifiable. Universal screening and optimization, standardized treatment protocols across surgical pathways, and targeted strategies for high-risk groups, particularly women of premenopausal age undergoing non-gynaecological surgery, should be prioritized. Life-stage-stratified performance metrics within perioperative governance may help identify unwarranted variation, guide resource allocation, and improve consistency of surgical care and outcomes for women.

## Collaborators

Members of the STARSurg Collaborative

 

 

##### Writing group

Dania Badran*, Omar Kouli*, Ieva Jakaitytė, Setthasorn Ooi Zhi Yang, Aneel Bhangu, Kariem El-Boghdadly, James Glasbey, Ana Minaya Bravo, Gregory Lip, Shakila Thangaratinam, Dharani Hangapama^†^, Sivesh K Kamarajah^†^

*joint first authors. ^†^joint senior authors *Data analysis*

Omar Kouli, Sivesh Kamarajah (overall guarantor)

##### Steering Committee

Kamarajah SK (Chair), Blanco-Colino R, Brindl N, Brown S, Cambridge WA, Chaudhry D, Gressmann K, Gujjuri RR, Hilder A, Jaffer A, Jakaityte I, Kawka M, Kouli O, Matey E, McLean KA, Mergo A, Mills EC, Murray V, Ooi SZY, Pereira IS, Riad AM, Sgrò A, Shafi SQ, Trout I, Turňa A, Varghese C, Xu W

##### External advisory group

Kamarajah SK (Chair), Biccard BM, Docherty A, Martin J, El-Boghdadly K, Phull M, Mouton R, Bhangu A, Glasbey JC, Harrison EM, McLean KA, Smart N, Moug S, Pinkney T, Richards T

##### National leads

Kamarajah SK (Chair), Dajti I (Albania), de Cillia M (Austria), Van Ramshorst G (Belgium), Delibegovic S (Bosnia and Herzegovina), Mughal H (Bulgaria), Mihanovic J (Croatia), Gouvas N (Cyprus), Kocian P (Czech Republic), Levesen V (Denmark), Kaupilla J (Finland), Brindl N (Germany), Merz D (Germany), Joos M (Germany), Ioannidis A (Greece), Nnaji G (Hungary), Pata F (Italy), Pellino G (Italy), Gori A (Italy), Podda M (Italy), Riboni C (Italy), Fekaj E (Kosovo), Oliver Senica S (Latvia), Dauksa A (Lithuania), Psaila J (Malta), de Ruitjer F (Netherlands), Major P (Poland), Santos I (Portugal), Sampaio Alves M (Portugal), Simões J (Portugal), Bonci EA (Romania), Pașca A (Romania), Novikova A (Russia), Kovačević B (Serbia), Milosavljevic V (Serbia), Tadic B (Serbia), Kosir J (Slovenia), Blanco-Colino R (Spain), Pérez Ajates S (Spain), Ossola Revilla ME (Spain), Papadia A (Switzerland), Riboni C (Switzerland), Gasparri ML (Switzerland), Aktas MK (Turkey), Baki BE (Turkey), Tepe MD (Turkey), Mutlu AU (Turkey).

##### Regional leads

(Kamarajah SK - Chair)

UK: Singal A (Aston University Medical School); Osei-Bonsu J (Barts and The London School of Medicine and Dentistry); Lacey H (Brighton and Sussex Medical School); Chan SW (Cardiff University School of Medicine); Allison M (Hull York Medical School); Duah-Asante K (Imperial College London Faculty of Medicine); Chen D (Keele University School of Medicine); Ahmed N (King’s College London GKT School of Medical Education); Ejiz A (Lancaster University Medical School); Takyi C (Newcastle University School of Medical Education); Mujeeb M (Plymouth University Peninsula Schools of Medicine and Dentistry); Ravikumar N (Queen’s University Belfast School of Medicine); Khan M (Royal College of Surgeons in Ireland School of Medicine); Hayes J (St George’s, University of London); Mckenna J (Swansea University Medical School); Wang J (The University of Edinburgh Medical School); Essa N (Trinity College Dublin School of Medicine); Xianghan H (University College Cork School of Medicine); Ko L (University College Dublin School of Medicine); Aldabbagh Y (University College London Medical School); Plascevic J (University of Aberdeen School of Medicine and Dentistry); Zia N (University of Birmingham College of Medical and Dental Sciences); Ismail R (University of Bristol Medical School); Kamel Y (University of Buckingham Medical School); Epanomeritakis I (University of Cambridge School of Clinical Medicine); Tan R (University of Dundee School of Medicine); Chiu N (University of Exeter Medical School); Naeem A (University of Glasgow School of Medicine); Kakwani M (University of Leeds School of Medicine); Mehra R (University of Leicester Medical School); Feeney K (University of Limerick School of Medicine); Yan Naing CL (University of Liverpool School of Medicine); Qureshi A (University of Manchester Medical School); Richens A (University of Nottingham School of Medicine); Li H (University of Oxford Medical Sciences Division); Ahmed R (University of Sheffield Medical School); Wilson L (University of Southampton School of Medicine); Abraha S (University of Warwick Medical School).

##### Collaborators

Albania: Dajti I* (University hospital Koco Gliozheni).

Austria: Mikalauskas S*, Kahn J, Kniepeiss D, Kušar M, Belarmino A, Frenk J, Mikalauskiene I, Angelis A, Waha JE, Al-Sharafy S, Sandano M, Schemmer P (Medical University of Graz); Reiterer C*, Horvath K, Taschner A, Riss S, Harpain F, Dawoud C, Hantáková N, Adamowitsch N, Schallmeiner S, Christian T, Xu V, Kuhrn MM, Widmann KM, Capek B, Kama B, Turgut S (Medical University of Vienna); Hauptmann L*, de Cillia M, Grünbart M, Hoi H (Saint John of God Hospital Salzburg); Binder A*, Gürtler T, Riedl P, Mayer D (Universitätsklinikum Tulln).

Belgium: Van Belle K*, Wautelet O, Dekkers E (Europe Hospitals); Van Daele E*, Apers T*, Van Ramshorst G, Berrevoet F, Rennie N, Dries P, Panta P, Denys A, Pype W, Bauwens T, Renard N (University Hospital of Ghent); Violon S*, Stijns J, Van De Winkel T, Van Eetvelde E (UZ Brussel).

Bosnia and Herzegovina: Salibasic M*, Delibegovic S, Pusina S, Hodžić E, Kruščica M, Žilić S, Bicakcic E, Rovcanin A, Kulovic E, Halilovic E, Vinčević Hodžić A (Clinical Center University of Sarajevo); Kalbić E*, Delibegovic S, Mujić A, Mešan M (General hospital Travnik); Matović E*, Delibegovic S, Kesetovic A (University Clinical Center Tuzla).

Bulgaria: Dardanov D*, Arabadzhieva E, Markov B, Спасова К, Biji C, Oliveira N, Miroslavov V, Korukov G, Ravendran K, Shah N, Mitkov Y, Arivanandan K, Gaikwad R, Gotru S, Saraff R, Sain N, Stanchev D, Gaydarski L, Ali U, Kandathil LJ, Krishnaveni A, Ivanov I, Ganev I, Krapeshki Т, Miza M, Mumu MSK, Bashliev I, Sajjad A, Chandel L, Yordanov D (Alexandrovska University Hospital); Hristova E*, Spassov K, Tsankov T (Fifth City Hospital); Ivanov T*, Filipov E, Georgiev D, Neykov V, Daleva E, Ilieva I, Krasimirova D, Hristov H, Арсова М, Soladoye O, Biswakarma A, Raj R, Negi A, Dey A, Adidela JK, Alionye UG, Ndukwe A (Heart and Brain Hospital); Dimov R*, Ivanov V, Sulcheva S, Mitrev S, Nikolov V (Kaspela); Karamanliev M*, Dimitrov D, Yordanov A, Vladova P, Shoshkova M, Karakadieva K, Gabarski A, Shanker A, Ehtisham H, Parambi S, Tranchev L, Vasileva A, Kareem J, Ezeabasili J, Madueke-Ediae OL, Muradia T, Saini A, Rana I, Kanagaratnam R, Thomas LS, Galasyuk M, Costa JJ, Majid N, Reddy P, Abdullahi M, Koshy Thomas S, Mehta A, Biswakarma A, Raj R, Shittu S, Theophilus J, Theophilus J, Ilyas-Uddin R, Ogunleye I, Naghavi E, Kiran A, Sundar A, Joy A, Ali F, Angelova I (UMHAT ‘Georgi Stranski’); Slavchev M*, Belev N, Atanasov B, Krastev P (UMHAT Eurohospital Medical University of Plovdiv); Yotsov T*, Dimitrov D, Kamenova P, Mihaylov I, Stavrov A, Vricheva A (University Hospital Medika).

Croatia: Šantak G* (County General Hospital Pozega); Penezić L*, Kastelan Z, Zimak Z, Saić H, Knezevic N, Cikic B, Zekulić T, Hudolin T, Juric I, Anđelić J, Kulis T, Maric M (University Hospital Centre Zagreb); Mihanovic J*, Soric T, Surić Grčić D, Blagus L, Kovačević P, Vidić I, Miočić Juran A (Zadar General Hospital).

Cyprus: Ergatidou C*, Gravas S, Yiallourou A, Achilleos S, Vardas M, Kokkinos G, Panagiotou C, Kyprianou M, Kyriacou K, Papatheodorou P, Konstantinou K, Onisiforou D, Tsoutsouki F, Kasapi A, Demetriou E, Stylianou A, Xenophontos E, Georgiou P, Makrides P, Pozotou I, Constantinou M, Evangelou T, Varavina A, Charitonos S, Lampi M (Nicosia General Hospital).

Czech Republic: Attaalla M*, Dušek T, Ndukwe M, Kuzmane L, Moorlata E, Sillah H, Kotek J, Fazal J, Ssali E, Steingauer V, Vinklerova K, Ahmad O, Rodríguez Martínez Á, Hiebert E, Srivastava M, Ramesh Babu AS, Dobra O, Akiba A, Kalou MF, Sokolová T, Nepeřená N, Novotný P, Sedlackova J, Philips F, Al Atassi H, Jose P, Sunil Nair A, Ntashamaje L, Kaddah A, Antabi MA (Fakultní Nemocnice Hradec Králové); Kocián P*, Fiala R, Harustiak T, Vesely S, Haluza A, Štekrtová A, Král V, Zdichová K, Pastor J, Gorchakov A, Novák H, Novák V, Hornak J, Havova M, Marková M, Fořtová T, Lérias Bento D, Novysedlák R, Smetana JF, Novák T, Zdobinska T, Švarc M, Hejduková E, Havlová K, Vaishevich A, Čorňáková J, Zajíc P, Francúz P, Příman O, Havlíková Š, Zemlickova B (Motol University Hospital); Whitley A*, Gurlich R, Balaz P, Tomyak I, Kútik M, Molva O, Šturc D, Belbl M, Vinklarkova T (University Hospital Kralovske Vinohrady).

France: Villefranque V*, Police A*, Mikhael E, Mabilia A, Charre L, Volpin E, Braham H, Montero Macías R, Krief D, Boyer De Latour A (Hôpital Simone Veil); Angeles MA*, Martinez A, Navarro A, Ferron G, Del M, Ghiani M, Migliorelli F (Institut Claudius Regaud); Danguy Des Déserts M*, Johan S, Olivier B, Andro C (Military Hospital Clermont Tonnerre (Hôpital des Armées)).

Germany: Von Wedel D*, Kamphues C, Thiele M, Felber J, Neudecker J, Schineis C, Lauscher JC, Stephan R, Meyer LP, Wolff C, Bombera B (Charité University Medicine); Rolinger J*, Kirschniak A, Wilhelm P, Göller S, Van Den Hil L (Kliniken Maria Hilf); Reim D*, Kießler M, Buschhaus C, Naisar S, Zahlmann N, Walter RL, Weber M, Müller K, Schweden A, Berlet M, Jorek NA (Klinikum Rechts der Isar TUM School of Medicine); Seyfried S*, Rahbari N, Birgin E, Reissfelder C, Reeg A, Téoule P, Rasbach E (Mannheim University Medical Center (Universitätsmedizin Mannheim)); Pyrgidis N*, Hatzichristodoulou G, Sokolakis I (Martha-Maria Hospital); Strotmann JJ*, Fahlbusch T, Höhn P, Hinrichs J, Horn J, Jollet S (St. Josef); Binder J*, Förtsch T, Winterstein M, Hackner D, Girard A (Universitätsklinikum Erlangen); Wittmann M*, Vilz TO, Recker F, Velten M, Massoth G, Delis A, Brugués Villalba MI, Willis M, Soltau S, Kudaliyanage S, Eymael RL, Syring I, Neubauer R, Dahmen N, Zimmermann A, Straßberger-Nerschbach N, Puskarevic A, Wang X, Peyman L, Philippi RA, Schmidt K (University Hospital Bonn); Bork U*, Schepp F, Korn S, Radulova-Mauersberger O, Von Dem Bussche J, Von Bechtolsheim F, Praetorius C, Knobloch N, Oehme F, Weitz J, Mansfeld M, Distler M (University Hospital Carl Gustav Carus); Klingler F*, Fluegen G, Dávid S, Schneider M, Baba KA (University Hospital Duesseldorf); Ronellenfitsch U*, Kleeff J, Ukkat J, Klose J, Bayram O, Sommerer M, Rebelo A (University Hospital Halle); Gockel I*, Gentsch E, Rayes N, Thieme R, Kreuser N, Aktas B, Branzan D, Seehofer D (University Hospital Leipzig); Mallmann MR*, Mallmann C, Woldu S, Balci F, Domröse C, Diekhöner M (University Hospital of Cologne).

Greece: Micha G*, Stroumpoulis K, Dritsoulas L, Kalopita K, Bitzi G, Thessalonikefs P (‘Elena Venizelou’ General and Maternity hospital of Athens); Fradelos E*, Korkolis D, Sarafi A, Manatakis DK, Tasis N, Prountzopoulou A, Tziava E (Agios Savvas Anticancer Hospital); Pikoulas K*, Paramythiotis D, Tishukov M, Papadopoulos K, Chatziantoniou G, Tzikos G, Papamichail S, Bareka S, Sarafis A (AHEPA University General Hospital); Haidopoulos D*, Angelou K, Prodromidou A, Stamatakis E, Alexakis N, Pergialiotis V, Rodolakis A, Thomakos N (Alexandra General Hospital); Memos N*, Vlahos N*, Koutalas I, Kotsis T, Kalampokas E, Chardalias L, Kontopoulou C, Lignou C, Lykourgioti P, Bagiasta A, Petrolekas A, Savranakis O, Kozonis T, Themelidi V, Mccormac-Prekeze M, Nikolaou C, Bletsa E, Melissaridou N, Flamourakis K, Rammos P, Hadjizacharias T, Provata N, Politis D, Gkioulekas AP, Massaras D, Vakos I, Antonoglou A, Dimitra P, Kotzadimitriou A, Evangelou C, Psychogios D (Aretaieion Hospital); Konstantinidis MK*, Apostolou K*, Konstantinidis K, Patelis N, Konstantinidou S (Athens Medical Center); Kokoropoulos P*, Michalopoulos N, Kratiras Z, Drakakis P, Sidiropoulos T, Papadoliopoulou M, Vassiliu P, Arkadopoulos N, Koratzanis C, Vrachnis N, Dylja E, Chatzialis I, Sampanis D, Danias N, Fotiou A, Stavros S, Petropoulou Z, Tsaousis V, Papakonstantinou D, Lykoudis P, Nastos C, Charalampopoulos A, Papiri IE, Hatzaras I (Attikon University General Hospital); Paraskevas KI* (Central Clinic of Athens); Petrakis G*, Polenta E, Kaparounaki E (Chania General Hospital ‘St George’); Sotiropoulou M*, Kapiris S, Mavrodimitraki E, Paraskeva A, Kolinioti A, Psarologos M, Stergiou D, Metaxas P, Stamatis K, Kyzeridis C, Kefalou E (Evaggelismos General Hospital); Vrakopoulou GZ*, Larentzakis A, Menenakos E, Maravgaki VR, Georgiadou C, Koutrouli M, Papageorgiou A, Skandali A (Evgenideio Hospital); Tzovaras G*, Baloyiannis I, Tzortzis V, Christodoulidis G, Perivoliotis K, Arnaoutoglou E, Ntalouka MP, Chatzis A, Daponte A, Samara A, Donoudis C, Zacharoulis D (General University Hospital of Larissa); Mulita F*, Bouchagier K, Verras G, Tchabashvili L (General University Hospital of Patras); Ioannidis O*, Koltsida A, Malliora A, Paparouni D, Bitsianis S, Loutzidou L, Anestiadou E, Χατζηανεστιάδου Χ, Simeonidis S, Tekelidis A, Zapsalis K, Skalidou S, Ouzounidis N, Ntampakis G, Kelepouri A, Kontidis F, Foutsitzis V, Kontaxi O, Barakakis G, Valaroutsou OM, Athanasiou C, Kotidis E, Pramateftakis M, Mantzoros I (George Papanikolaou General Hospital of Thessaloniki); Toutouzas K*, Frountzas M, Triantafyllou T, Triantafyllou A (Hippocratio General Hospital); Dagklis T*, Katsanos G*, Petousis S*, Athanasiadis A, Tsoulfas G, Dinas K, Tsakiridis I, Mamopoulos A, Kalogiannidis I, Rao F, Christou C, Vasileiadou S, Kapetanios G, Tsakiridis N, Kopatsaris S, Papanikolaou E, Karakasi KE, Tataridou TA, Antoniadis N, Zachomitros F, Arvanitaki A, Tsakiridis K, Anemoulis M, Neiros S, Ouranos K (Hippocratio Hospital); Tampaki EC*, Maltezos C, Maltezos K, Anastasiadou C, Chaveles A, Pachi A (KAT Athens General Hospital); Tsiantoula P*, Roditis K, Antoniou A, Bessias N, Papas T, Tzamtzidou S, Maras D, Papaioannou V (Korgialenio-Benakio Hellenic Red Cross Hospital); Koukoulis G*, Bouliaris K, Skriapas K, Kontopoulos G, Doudakmanis C, Kolla C, Efthimiou M, Kalfountzos C, Mitsakou D, Kardasi C, Zourntou S, Fountarlis LI, Bakalis A, Samarinas M, Chatzilamprou PL, Migdanis A, Karvouni K, Katsiafliaka K, Arvaniti C, Papazisi AR, Markatou V, Zervas K, Marsitopoulos K (Koutlimbaneio and Triantafylleio General Hospital of Larissa); Machairas N*, Dorovinis P, Kotsifa E, Keramida MD, Schizas D, Vailas M, Syllaios A, Mela E, Hasemaki N, Skotsimara A, Katsargyris A, Kykalos S, Tomara N, Palios I, Karniadakis I, Charalabopoulos A, Sakarellos P, Davakis S, Kydonakis N, Tsourouflis G, Stamopoulos P, Laios K, Kozadinos A (Laiko University Hospital); Katsaros I*, Kontis E, Iavazzo C, Katsiaras L, Kaouras E, Manikis P, Kokkali K, Theodorou G, Dragi A, Vorgias G (Metaxa Cancer Hospital); Tzelves L*, Skolarikos A, Manolitsis I (Sismanoglio General Hospital of Athens); Spartalis M*, Tzima I, Anastasiou A, Pantelidis P, Schismenou G, Spartalis E, Zakynthinos GE (Thoracic Diseases General Hospital Sotiria); Lasithiotakis K*, Petra G, Lampou M (University Hospital of Heraklion Crete).

Hungary: Toth D*, Varga Z, Pósán J, Illésy L, Váradi C (Debrecen University Clinical Center).

Italy: Santarelli M*, Puca L, Brignone L, Lisa Marie R, Silvia L, Silvia P, Ferguglia A, Piceni C, Improta M, Assanti F, Montanari E (‘Città della Salute e della Scienza’ di Torino); Ettore G*, Cannone F, Cormaci L (ARNAS Garibaldi “Nesima”); Nicastro V*, De Carlis R*, Ferrari G, Giani A, Grimaldi S, Gregorio L, Mazzola M, Ripamonti L, Lorusso L, Tartufari GA, Magistro C, Benedetti A, Gonta A, Maesano J, Bertoglio CL, Giusti I, Quagli O, Brucchi F, Bevilacqua E, De Carlis L, Lauterio A, Cerchione R, Migliorini M, Incarbone N, Centonze L, Darwish SS, Vella I, Buscemi V, Podestà A (ASST Grande Ospedale Metropolitano Niguarda); Desio M*, Berselli M, Megna S, Cocozza E, Livraghi L, Quintodei V, Marchionini V, Borroni G, Peverelli C, Corbella M (ASST Settelaghi); Ferrari F*, Odicino F, Gozzini E, Cisotto F (ASST Spedali Civili Ospedale di Brescia); Baronio G*, Del Giudice R, Sala G (ASST Valcamonica Ospedale di Esine); Iacomino A*, Vennarecci G, Ferraro D, Di Martino M, Pisaniello D, Falaschi F (Azienda Ospedaliera di rilievo nazionale Antonio Cardarelli); Petagna L*, Giuliani A, Di Lascio P, Coluzzi M, Pascale G, Gallicchio P, Frezza G, Dinatale G (Azienda Ospedaliera Regionale ‘San Carlo’); Cerino G*, Bottari A*, Martellucci J, Maltinti G, Scheiterle M, Fortuna L, Staderini F, Coratti F (Azienda Ospedaliera Universitaria Careggi); Veroux M*, Giaquinta A*, Roscitano G*, Volpicelli A*, Veroux P, Palumbo M, Riccioli G, Stella L, Distefano C, Gioco R, Lomeo G, Lomeo E, Cavallo M, Virgilio C, Molino C, Giambra MM, Zerbo D, Santini R, Costa S, Incognito GG, Centonze DC (Azienda Ospedaliero); Currò G*, Ammendola M, Filippo R, Ammerata G (Azienda Ospedaliero Universitaria Mater Domini); Balestri R*, Morelli L*, Tartaglia D*, Puccini M, Asta VF, Conte V, Buccianti P, Sargenti B, Boni G, Signori S, Pezzati D, Urbani L, Casale G, Taddei G, Di Franco G, Bianchini M, Carpenito C, Furbetta N, Comandatore A, Tarasco F, Guadagni S, Mastrangelo M, Gianfaldoni C, Palmeri M, Fatucchi LM, Boato AB, Gianardi D, Stingone M, Sacco L, Giaquinto S, Lami L, Iuliano M, Annunziata EA, Chiarugi M, Strambi S, Coccolini F (Azienda Ospedaliero Universitaria Pisana); Anania G*, Chiozza M, Campagnaro A, Nevoso V (Azienda Ospedaliero Universitaria San’Anna); Carbone L*, Marano L, Micheletti G, Fontani A, Malerba S, Gambelli M, Bombino A, Grassi G (Azienda Ospedaliero Universitaria Senese); Mandato VD*, Aguzzoli L, Mastrofilippo V (Azienda Unità Sanitaria Locale); Fabbri N*, Feo CV, Ginestri M, Oppici D, Torchiaro M, Pesce A (Azienda Unità Sanitaria Locale di Ferrara); Catena F*, Vallicelli C (Bufalini Hospital); Murzi V*, Podda M, Pisanu A, Campus F, Pilia T, Saba A, Piras C, Pisano M, Locci E, Marongiu P, Gessa E (Cagliari University Hospital); D’Oria Mario*, Biloslavo A*, Lepidi S, Germani P, Nappi CJ, Grando B (Cattinara University Hospital); Lauretta A*, Pollesel S, Brollo PP (Centro di Riferimento Oncologico di Aviano (CRO)); Gallo G*, Trompetto M, Realis Luc A, Tiesi V (Clinica S. Rita); Porcu A*, Perra T, Madonia M, Tedde A, Scognamillo F, Tilocca PL, Delogu D, Mucci G, Drocchi G, Tedde M, Scanu AM, Rizzo G, Fancellu A, Feo CF, Farina G, Casu R, Cossu ML, Ginesu GC, Anania M, Dessole S, Capobianco G, Petrillo M, Dessole F, Angelone I (Cliniche San Pietro); Barberis A*, Azzinnaro A, Petrungaro A, Palli E, Mina E (E.O. Ospedali Galliera); Pipitone Federico NS*, Muratore A, Calabrò M, De Zuanni M, Herranz Van Nood E (Edoardo Agnelli); Licari L*, Callari C, Di Miceli D, Viola S (FBF Buccheri La Ferla Palermo); Manigrasso M*, Caricato C*, Milone M, Anoldo P, Vertaldi S, D’amore A, De Palma GD, Marello A, Cantore CG, Chini A, Maione F, Luglio G, Pagano G, Tropeano FP, Cricrì M, Aprea G, Palomba G, Capuano M, Basile R, Bracale U, Peltrini R, Visconti M, Magno G, Di Santo Albini AG (Federico II University of Naples); Fransvea P*, Sganga G, Tropeano G, Puccioni C, Altieri G, Fico V, Mirco P, Bianchi V, Cozza V, Pepe G, Alfieri S, Rosa F, Schena CA, Laterza V, Pascale MM, Agnes S, Bianco G, Frongillo F, Ferracci F, Santullo F (Fondazione Policlinico Universitario Agostino Gemelli); Balla A*, Lepiane P, Saraceno F (Hospital “San Paolo”); Mastroianni R*, Simone G, Tuderti G (IRCCS ‘Regina Elena’ National Cancer Institute); Marino F*, Lantone G, Lantone G, Isernia R, Pezzolla F, Aquilino F (IRCCS ‘Saverio de Bellis’); Raimondo D*, Gori A*, Seracchioli R, Rottoli M, Belvedere A, Maurino L, Russo IS, Orsini B, Maletta M, Romano A, Bernante P, Violante T, Morezzi D, Degli Esposti E, Raffone A, Poggioli G, Canavese A, Dajti G, Cardelli S, Casadio P, Arena A, Sanna G, Cuicchi D, Angelicchio LF, Catalioto C, Torre B, De Iaco P, Tesei M, De Crescenzo E, Larotonda C, Greco F, Minghetti M, Sissa L, Perrone AM, Dondi G, Di Stanislao M, Gaetani L, Serafini L, Prosperi E, Perone D, Isopi C, Bruno F, Alexa ID (IRCCS Azienda Ospedaliero); Milito P*, Lovece A, Chiappini G, Froiio C, Panici Tonucci T, Saletta G, Scardino A (IRCCS Policlinico San Donato); Belli A*, Izzo F, Patrone R, Cutolo C, Palaia R, Granata V, Albino V, Leongito M, Piccirillo M, Pasta G, Delrio P, Rega D, Rho M, Aversano A, Angelone IA, D’amico M, Di Marzo M, De Franciscis S (Istituto Nazionale Tumori IRCSS Fondazione G. Pascale); Cianci P*, Restini E, Conversano I, Scialandrone G (Lorenzo Bonomo); Di Saverio S*, Cardinali L, Travaglini G, Sebastiani E, Marziali I (Madonna del Soccorso Hospital); Bellocchia AB* (Maria Vittoria Hospital); Raimondo I*, Di Giorgio D*, Garganese G, Tondolo V, Dore A, Pacini G, Turco LC, Campennì P, Amara MA, Rubattu A, Verri D, Sole G, Bove S, Inzaina F, Franceschi F, Grande G, Pili DP, Campus F, Delogu G, Sula V, Ez Zinabi O, Congiu M, Pira E (Mater Olbia Hospital); Ercolani G*, D’acapito F, Annunziata D, Solaini L (Morgagni); Giordano A*, Cantafio S (Nuovo Ospedale di Prato); Novello S*, Romano M, Armellin C, Zanus G, Milardi F, Grossi U, Baldan R, Brizzolari M, Scolari F, Brun-Peressut A (Ospedale Ca’ Foncello); Broglia A*, Martorana M, Fazzini N (Ospedale Civile di Voghera); Marafante C*, Garino M, Moggia E, Agosti SC, Battaglia A, Borello A, Birolo SL, Barone R, Mosca C, Shakhova K, Apostu AL (Ospedale degli Infermi di Rivoli); Giaccari S*, Pavanello M, Migliori E, Sambucci D (Ospedale di Conegliano); Corbellini C*, Sampietro GM, Germiniasi G, Bischeri M (Ospedale Di Rho); Colombo F*, Danelli P, Cammarata F, Zingale RS, Pezzoli I, Carbonaro M, Albanesi F (Ospedale Luigi Sacco Milano); Cozzani F*, Rossini M, De Gennaro F, Giuffrida M, Del Rio P (Ospedale Maggiore di Parma); Inama M*, Moretto G, Iudici L, Vitali A, Creciun M, Impellizzeri H, Piazza M (Ospedale Pederzoli); Biancafarina A*, De Prizio M, Malatesti R, Sulce R, Pellicano’ GA, D’ignazio A, Mariottini V, Mura G, Angelini M, Scricciolo M, Barbara F, Tofani F, Nenci L, Borgogni V, Vece C (Ospedale San Donato USL Toscana Sud Est); Sagnotta A*, Solinas L, Rossi M, Mancini S (Ospedale San Filippo Neri); Fruscio R*, Ciulli C*, Braga M, Romano F, Garancini M, Carissimi F, Vico E, Delle Grazie CDG, Negri S, Corti J, Tamini N, Cigagna L, Giordano R, Davolio A, Trezzi G, Grassi T, Procaccianti C, Nespoli LC, Pitoni L, Fogliati A, Cordaro G, Passoni P, Fantauzzi M, Villa S, Scotti MA, Di Lucca GM, Benedetti F, Masseria P, Frigerio M, Barba M, Re I, Ceresoli M, Ferraina F, Adjei Antwi SK, Fumagalli C, Di Martino G, Di Meo ML, Bazzano L (Ospedale San Gerardo); Nelli T*, Masciello F*, Canonico G, Chisci E, Adinolfi E, Borreca R, Damigella A, Di Martino C, Mottola GIL, Fontani G, Michelagnoli S (Ospedale San Giovanni di Dio); Fedi M*, Leo F, De Vincenti R, Cecchi C, Piombetti L, Giannessi S, Benedetta B, Pagani M (Ospedale San Jacopo); Epis L*, Percivale A, Malerba M (Ospedale Santa Corona); Tonini V*, Cervellera M, Shahu J, Sartarelli L (Ospedale Santissima Annunziata); Luzzi A*, Romairone E, Carrabetta S, Tuminello F, Floris F, Ré F, Marzorati S, Ballari F, Meola C, Filippelli A, Ribeca UG, Viacava A (Ospedale Villa Scassi); Lizzi V*, Montagna M, Giuliani A, Tartaglia N, Vovola F, Maffei F, Cotoia A, Pacilli M, Pavone G (Ospedali Riuniti Azienda Ospedaliera Universitaria); Berardi G*, Depalma N, Maruccio M, Spampinato MG, D’ugo S, Marchese T, Basurto F, De Giorgi C, Garritano S, Manoochehri F, Perrone F, Botrugno I, Rizzi A, Sergi W (P.O.‘Vito Fazzi’); Russo GI*, Cappellani A, Cimino S, Asmundo MG, Matarazzo MG, Di Vita M (Policlinic Hospital ‘G. Rodolico’); Venturelli P*, Cocorullo G, Zarcone F, Dominici DM, Salamone G, Tutino R, Corigliano A, Guercio R, Giuseppe C, D’arpa F, Melfa G, Proclamà MP, Scerrino G, Canfora I, Marcianò M, Guercio G, Barbera S, Amato C, Agostara AV, Pantuso G, Orlando G, Finocchiaro N (Policlinico ‘P. Giaccone’); Picciariello A*, Altomare DF, Vincenti L, Tomasicchio G, Paradiso N, Dezi A (Policlinico di Bari); Pinotti E*, Montuori M (Policlinico San Pietro); Siragusa L*, Pathirannehalage Don C*, Sica G, Costanzo G, Usai V, Carrubba I, Pellicciaro M, Franceschilli M, Pirozzi BM, Santori F, Busso MB, Mariani Ivanikhin A, Guida AM, Sensi B, Divizia A, Blasi F, Giudice V, Orecchia L, Angelico R, Bacchiocchi G, Tariciotti L, Billeci F, Quaranta C, Cascone C, Materazzo M, Toti L, Manzia TM, Di Lorenzo N, Monaco A, Pedini D, Bagaglini G, Keçi L (Policlinico Tor Vergata Hospital); Lapolla P*, Mingoli A, Brachini G, Cirillo B, Sapienza P, Cicerchia PM, Leonardo C, Bologna E, Licari LC, Zambon M, Mazzarella G, Falasca A, Duranti G, Flammia RS, Asero V, Bernardotto A, Scarno F, Meneghini S, Giovampietro S, Binda B, Tufano A, Palombi V, De Meis E, Rocchetti M, De Padua C, Mansi M, Bruzzaniti P, Familiari P, Nottola SA (Policlinico Umberto I); Fleres F*, Cucinotta E, Biondo SA, Viscosi F, Catarsini N, Mazzeo C, Iannello M (Policlinico Universitario G. Martino of Messina); Testa V*, Gattolin A, Rimonda R, Riente F, Travaglio E, Allisiardi F, Guffanti P (Regina Montis Regalis Hospital); Ferrara F* (San Carlo Borromeo); Barbieri MR*, Puzziello A, Mollo A, Donnarumma E, Steccanella F, Oliviero A, Lopez G, Di Maio CM (San Giovanni di Dio e Ruggi d’Aragona); Olmi S*, Di Capua F, Camillo’ A, Carresi A, Uccelli M, Rubicondo C (San Marco Hospital GSD); Rosati R*, Elmore U, Puccetti F, Socci D (San Raffaele Scientific Institute); Clementi M*, Giuliani A, Tontoli S, Nisi A, Grasso A, Derraj S, Di Biasi J, Tucceri Cimini I (San Salvatore Hospital); De Nunzio C*, D’annunzio S, Nacchia A, Gallo G (Sant’Andrea Hospital); Lisi G*, Spoletini D, Signore S, Menditto R, Carlini M (Sant’Eugenio Hospital); Sasia D*, Dalmasso E*, Trucco A*, Olearo E*, Giuffrida MC, Morra I, Maione M, Puppo A, Marano A, Preve G, Vercellone B, Frola E, Beltrami E, Peluttiero I, Giraudo G, Migliore M, Armentano S, Alessandria E, Daniele A, Elisa P, Bonino L, Borghese M (Santa Croce e Carle Hospital); Bono D*, Canova G, Zago M, Nicotera A, Bonomo LD, Gattoni L (Santi Pietro e Paolo); Cillara N*, Deserra A, De Lisa A, Coccollone E, Cannavera A, Cabula R, D’agostino F, Deplano M, Chillura C, Robuschi ML, Borzacchelli A, Margiani C, Murru MC (Santissima Trinità); Pellino G*, Lieto E, Cardella F, Auricchio A, Erario SMC, Del Sorbo G, Lidia O, D’ambrosio M, Menegon Tasselli F, Mastroianni S, Selvaggi F (Universitá della Campania ‘Luigi Vanvitelli’); Calini G*, Terrosu G, Clocchiatti L, Morinelli V, Valotto C, Traunero F, Vizzielli G, Restaino S, De Gennaro E, Baccarani U, Andriani A, Mauro J, Frigatti P, Martin E, Pregnolato S, Driul L (University Hospital of Udine); Zullo A*, Carcano G, Latham L, Inversini D, Vigezzi A, Ietto G, Marta R, Marzorati A, Colombo EM, Garbarino S, Palamara N, Tozzi M, Piffaretti G, Franchin M, Festi LF, Odeh M, Fanelli MC, Costa J, Pappalardo V, Scorza A, Cannavò M, Ballabio A (University of Insubria Ospedale di Circolo e Fondazione Macchi (Varese)); Barina A*, Franzato B, Rubin L, Brugnolo D, De Simoni O (Veneto Institute of Oncology (IOV) IRCCS).

Latvia: Senica SO*, Ozoliņš A, Vjaters E, Jain N, Apse RR, Truskovs A, Apse IA, Jukonis P, Jukonis J, Tumova A, Sivapalan H, Piirtola OSS, Berga AE, Tumegård H (Paula Stradiņa Klīniskā universitātes slimnīca).

Lithuania: Samalavicius N*, Nutautiene V, Aliosin O, Eismontas V (Klaipeda University Hospital); Dauksa A*, Venclauskas L, Jasaitis K, Lazauskaitė D, Urbonas K, Šlenfuktas V, Grikytė I, Jokubauskas M, Simanaitis V, Dauksa Z, Gudaityte R, Riauka R, Krunkaitytė U, Svagzdys S, Šikarskė A, Pakrosnyte J (Lithuanian University of Health Sciences Kaunas Clinics); Stratilatovas E*, Piscikaite A (National Cancer Institute); Žaldokaite G*, Poskus T, Olekaitė V (Vilnius University Hospital).

Malta: Psaila J*, Andrejevic P, Cini C, Cassar K, Mattocks S, Sammut M, Mizzi S, Abela L, Micallef A, Pace K, Schembri B, Cini Custo R, Harmsworth M, Farrugia B, Theuma F, Gatt R, Montebello G, Spiteri N, Galea J, Vassallo L, Fava L, Ebejer A, Carabott K, Iles K, Casingena L, Apap Bologna G, Debono G, Galea J, Sammut M (Mater Dei Hospital).

Netherlands: Huisman S*, Leclercq W (Máxima Medical Center); De Ruijter F*, Konsten J (VieCuri Medisch Centrum).

North Macedonia: Adramanova D*, Nikolovski A, Otljanski A (University Surgery Clinic “Sv. Naum Ohridski”).

Poland: Kisielewski M*, Richter K, Kłos N, Stefura T, Macheta K, Alsoubie I, Tempski J, Wolińska N, Wysocki W (5th Military Clinical Hospital); Starek E*, Nowak NG*, Tarkowski R, Wallner G, Kobak J, Mączka E, Żak K, Ziółkiewicz A, Kułak K, Langa E, Łaba Ł, Krzesińska I, Bobiński M, Frankowska K, Dziurda S, Martyna J, Radulski J, Lulko O, Tomczyk J, Jasiński M (Independent Public Teaching Hospital No 1 in Lublin); Major P*, Rymarowicz J, Zielińska Z, Siuda K, Kęska M, Wites P, Błasiak P, Sierżęga A, Kuciel J, Tomczak D (Jagiellonian University Medical College); Molasy B*, Nieroda P, Buras E (St. Alexander Hospital).

Portugal: Gonçalves Pereira R*, Patrocínio S, Reis S, Rolo Santos C, Moniz L, Bernardo P (Centro Hospitalar Barreiro Montijo); Osório C*, Matos L, Carvalho L, Marques Antunes J, Alçada N (Centro Hospitalar de Entre o Douro e Vouga - Hospital de São Sebastião); Marçal A*, Tenreiro N, Martins D, Leal C, Ferreira C, Marques R, Freitas F, Marques C, Melo A, Silva S, Vieira B, Fernandes U (Centro Hospitalar de Trás-Os-Montes e Alto Douro); Freire B*, Fonseca S, Reis M, Macedo Cardoso De Oliveira C, Mendes J, Carvalho M, Macedo J (Centro Hospitalar do Medio Ave); Oliveira J*, Mexedo C, Sousa Silva D, Davide J, Emanuel Do Vale Gonçalves De Castro Alves E, Ginestal M, Santos MD, Santos J, Robalo C, Valente V, Fernandes Dos Santos JP, Mesquita I, Teixeira B, Marques Monteiro M, Silva MI (Centro Hospitalar do Porto); Nunes Coelho M*, Teixeira M, Silva RL, Lopes L, Ribeiro A, Lima Da Silva C, Correia De Sá T, Martins M, Costa M, Gil CG, Barros M, Santos RF, Silva A, Pedro J (Centro Hospitalar do Tamega e Sousa); Costa M*, Ventura Antunes TA, Pascoal M, Andrade R, Duque M, Prior I, Lemos M, Pinto P, Guerreiro P (Centro Hospitalar e Universitário de Coimbra - polo HUC); Castanheira Rodrigues S*, Barbosa E, Martins AL, Pereira A, Vaz S, Fareleira A, Rocha-Neves J, Dias L, Girão De Caires F, Pereira-Neves A, Oliveira AI, Araujo Teixeira JP, Ferreira J, Almeida M, Vb Machado M, Nogueiro J, Gomes F, Campos E, Jácome F, Coutinho C, Ribeiro Dias R, Vieira De Sousa JP (Centro Hospitalar e Universitário de São João); Francisco E*, Borges C, Pereira S, Pereira C, Machado N, Calaia R, Pinto J, Corvelo Pavão T, Santos Sousa LG, Cunha F, Melo Pinto D, Pereira R, Dinis I, Ferros C, Pinheiro JL, Santos AJ, Barbosa B, Gaspar D, Pinto M, Pereira D, Araújo N, Alves B (Centro Hospitalar Tondela); Barbosa H*, Silva Araújo D, Afonso M, Guimarães B, Campos Coroa M (Centro Hospitalar Vila Nova de Gaia/Espinho); Susano MJ*, Azevedo J, Pereira M (Fundação Champalimaud); Miranda P*, Garrido R, Oliveira Mourão M, Ferreira M, Duarte A, Félix P, Antunes I, Fernandes N, Gil Corrêa Figueira C, Figueiredo I, Gututui M, Oliveira S (Hospital Beatriz Angelo); Silva A*, Madeira Martins F, Cabral A, Rodrigues CS, Gama B, Silva Borges R (Hospital da Horta); Lourenço R*, Longras AC, Araújo R, Peixoto I, Santos A, Matos D, Lopes A, Claro L (Hospital da Senhora da Oliveira); Cardoso D*, Martins A, Silvestre M, Sousa G, Santos Bessa M, Cardoso V (Hospital de São Francisco Xavier); Velez C*, Machado A, Pedroso De Lima R, Matias MI, V. Matos S, Rocha Melo M, Bolota J, Rente M, Santos A, Antunes ML, Bárbara A, Rosin E, Oliveira J, Leandro S, Damasio Cotovio M, Morgado S (Hospital do Espirito Santo); Fialho G*, Andrade N, Valente Costa Pinto F, Capote H, Morais S, Buruian M, Taré F, Santos G, Rosado D, Costa C, Mogne T, Pratas N, Cordeiro B, Brito M, Salvador D (Hospital Doutor José Maria Grande); Carvalho S*, Capella V, Carracha M, Alves R, Miranda C, Rebelo F, Teles T, Ferreira M (Hospital Dr. Antonio José de Almeida); Cismasiu B*, Costa PM, Henriques S, Trindade M, Vaz J, Gomes Vieira M, André M, Macedo A, Gonçalves Nobre JG, Prosil Sampaio J, Mira AR, Preto Barreira AL, Botelho P (Hospital Garcia de Orta); Melo Pinto D*, Quintela C, Quelhas MJ, Pereira D, Cruz A, Mesquita Guimarães C, Pinto C, Maldonado F, Sales F, Marrana F, Reis Aguiar AR, Freire L, Farias L, Faria-Costa G, Castro A, Moreira Marques T, Cardoso G (Hospital Pedro Hispano); Ribeiro J*, Fragoso M, Silva C, Vasconcelos M, Picciochi M, Sampaio Soares A, Frazão J, Miranda Pera R, Gaboleiro F, Ramalho De Almeida F, Mendes A, Afonso F, Fontaínhas J (Hospital Professor Doutor Fernando da Fonseca); Reia M*, Angel M, Aguero C, Guerrero M, Dominguez J (Hospital Santa Luzia de Elvas); Borges Da Costa I*, Ramos L, Fernandes J, Assis C (Hospital Vila Franca de Xira); Fonseca F*, Carvalhal S, Caiado A, Brito Da Silva F, Miguel B, Moniz J, Pires M, Leal B, Nunes J, Matos Sousa F (Instituto Português de Oncologia de Lisboa Francisco Gentil); Vieira Caroço T*, Romano M, Ângelo M, Rodrigues Ferreira A, Prata Saraiva AR, Mano F, Rodrigues F, Branco T, Gaspar S, Neves F, Alves M (IPO Coimbra); Ferreira Pinto AP*, Peyroteo M, Baía C, Marques M, Correia AM, Silva JO (IPO Porto); Monteiro R*, Silva-Vaz P, Gomes J, Moutinho Teixeira MJ, Brito Neves T, Meruje F (Unidade Local de Saúde de Castelo Branco).

Republic of Ireland: Sheahan C*, Macnamara D, Linares Gómez JE, Clare S, Walmsley A, Tiwari A, Khan T, Dowling G, Hudak K, Craig E, Dhannoon A, Saeedi S, Shah J, Jordan M, Buckley T, Engler J, Reilly M, Ariaratnam J, Hinn MA, Benn K, Petropoulos S, Hansen HS, Browne S, Keelan S, Basta M, Pornsakulpaisal R, Tan G, Niyaz AN, Lakic N, Warhust T, Chang YQ, Qaoud Y (Beaumont Hospital, Dublin); Mwipatayi D*, Kearney D, O’reilly C, Gamper Y, Dhillon R, Patel V, Gallo K, Mulvaney H, Edwards P, Tiller S, Gorecki E, Jordan M, Daswani S, Jones V, Asekomhe I, Clausen N, Khan M, Feldiorean A, Pezeshki A, Khedro T, Beyer J, Byrne G, Irfan W, Poluha A, Fager C, Gilligan-Steinberg L, Yarp J, Byrne E, Lorico-Rappa M, Hassan A, Keogh T, Denning W, Horton-Schleicher K (Connolly Hospital, Dublin); Deane T*, Haren A, Md Hamsan AD, Mukerji N, Dwivedi M, Cheah SML, Kwek PX, Arshad S, Alzaki A, Cornila I, Feizal HM, Barry M, Semar A, Gorman P, Gordon A, Mcsweeny C, Layyous N, Peiris D, Pan S (St Vincent’s University Hospital); Larkin J, Umar MM, Doherty C, Mitchell C, Mcelvaney H, Sabz Ali Z, Adesokan S, Murphy A, Yeo A, Hayes L, Owens C, Crawley N, Macinnis E, Elekes S, Oderoha KE, Mac Mahon I, Moran B, Matreja P (St. James’ Hospital); Conlon K, Piankova P, Farran MZ, Kashyap SV, Puntambekar M, Sampat M, Heaslip Owens S, Maheshwari A, Soo A, Butt J, O’brien K, Almutairi M (Tallaght University Hospital); Mcfeetors C*, Kerin M, Davey MG, Murray W, Nasehi A, Graham A, Mathew C, Azam S, Chua Vi Long K, Stafford-Johnson S, Ejaz D, Siddiqi Z, Kon AMH (University Hospital Galway); Parmar M*, Peirce C, Shomoye D, Alabi A, Salzberg N, Gallippi V, Aziz J, Melly C, Gill H, Yang HW, Eltahir Ibrahim SE, Rugber Singh JSK (University Hospital Limerick).

Romania: Bacalbasa N*, Balescu I (Clinical Hospital Dr. Ion Cantacuzino); Muresan M*, Dudric V, Imihteev I, Chelaru V, Băiceanu TM, Alexandra M, Bashimov A, Voda M, Muresan D, Varadi R, Muntean M, Macovei Ș, Checiu R, Buf D (Cluj-Napoca Municipal Hospital); Grama F*, Chitul A, Bezede C, Ciofic E (Coltea Clinical Hospital); Beuca A*, Bintintan V, Muresan D, Raluca-Cristina A, Rad R, Șușman S, Suciu C, Abu Arif J, David A, Blaga M, Olesea B, Butnariu DA, Scurtu RR, Rad R, Claudia-Mihaela P, Girlovanu M, Costina D, Drasovean R, Tartiu G, Tache S, Irina N, Streang A, Trif A, Romascan I, Stănică E, Vass E, Cucoreanu C (County Emergency Hospital); Toma EA*, Calu V, Enciu O, Laceanu A, Maria-Zamfirescu R, Matache IM, Mușat I, Piriianu DC, Georgescu AA, Miron A, Tartalea M, Al-Ugeily N, Bogdan-Gabriel B, Alexiadi A, Zaharescu A (Elias Emergency Hospital); Ciubotaru C*, Negoi I, Paunescu M, Iancu I, Pop IV, Diaconescu B, Dao-Zamfirescu P, Patel NB, Rafi S, Popescu D, Girbaciu M, Rădoi A, Breuer C, Braun AC, Stoica B, Gîlia I, Gheorghe A, Popa EC, Brichius F, Bucurica A, Negoita VM, Radu M, Bianca Ștefania R, Socol CL, Baiceanu AM (Emergency Clinical Hospital Bucharest); Bubenek Turconi SI*, Valeanu L, Girel M, Gavrila MR, Pirvu M, Morosanu B, Ioniana M, Barbu A, Cornel R, Bogdan B, Bute T, Madec L, Burla E, Balan C, Alexandru E (Emergency Institute for Cardiovascular Diseases ‘Prof. Dr. C.C. Iliescu’); Tomescu D*, Popescu M, Dumitru M, Marinescu A, Petrusca I (Fundeni Clinical Institute); Pasca A*, Achimas-Cadariu P, Irimie A, Petrusan A, Puscas M, Titu S, Jiboc NM, Vlad IC, Eniu DT, Ciubotariu P, Gata VA, Rancea A, Iulia C, Morariu DS, Nistor-Ciurba CC, Gale I, Lazar G, Bonci E, Ignat F, Lisencu C, Dumitraș C, Faur FI, Butuza MO, Marian MC, Paul Luchian A (Prof. Dr Ion Chiricuta Institute of Oncology); Bocse H*, Puia IC, Andra C, Pop P, Moldovan S, Ursu S, Sorin M (Regional Institute of Gastroenterology & Hepatology “Prof Dr Octavian Fodor”); Lunca S*, Dimofte M, Musina A, Velenciuc N, Roata CE, Morarasu S (Regional institute of Oncology Iasi); Velescu M*, Mureșan F, Zaha I, Rusu IM, Mircea B, Tauberg DG (University Hospital).

Russia: Kirov M*, Kuzkov V, Nikonov A, Tishchenko A, Kulin D, Mironov V (City Hospital #1); Litvin A*, Gunko I, Mazhega I, Bilenko R (Immanuel Kant Baltic Federal University); Khrykov G*, Манкевич Н, Karpenko M, Zagaynov E, Shilyaev A (Leningrad Regional Clinical Oncology Dispensary); Butyrskii A*, Tatidze A, Seytnebieva Z, Aliev A, Rumyantseva M (Municipal Emegency Hospital No.6); Yanishev A*, Abelevich A, Doktorov S, Zuev K, Lazareva A, Malov A (Nizhny Novgorod regional clinical hospital); Borisov D*, Борисов Е, Андрийчук И (Northern Medical Clinical Centre of them. N.A. Semashko); Galchikova Z*, Shemetova M*, Maltsagova K (P. HERTSEN MOSCOW ONCOLOGY RESEARCH INSTITUTE); Bedzhanyan A*, Temirsultanova K, Efremov D, Volkova A, Frolova Y, Minenkova A, Petrenko K, Sumbaev A, Bredikhin M, Bedzhanyan E, Mikhailova A (Petrovsky National Research Centre of Surgery); Тен В*, Кудрявцев Ю (Private Healthcare Institution “Russian Railways-Medicine””); Novikova A*, Pavlov R, Chernykh M, Boiko N, Данилин В (Saint Petersburg State University Hospital).

Serbia: Stanojevic G*, Brankovic B, Nestorovic M, Milutinovic N, Vukadinović A, Vukadinović A (Clinical Center Nis); Stojanovic B*, Radovanovic D, Cvetkovic A, Radosavljevic I, Sreckovic M, Milosevic B, Markovic N, Spasic M, Pavlovic M, Lazic D, Vucic R (Clinical Centre of Kragujevac); Tadic B*, Kajmaković B*, Vilendecic Z*, Knezevic D, Bumbasirevic U, Stefanovic A, Gregoric P, Perišić Z, Micic D, Ratkovic S, Vasiljević J, Bulat PR, Jovanovic V, Mitrović O, Gunjic J, Potkonjak D, Grubor N, Reljic M, Palibrk I, Milutinovic V, Zivkovic M, Škrijelj D, Grubač Ž, Kadija S, Cerovic Popovic R, Andric L, Aleksić L, Kmezić S, Pavlovic I, Djukanovic M, Milojevic B, Dzamic Z, Petrovic M, Jeremic K, Pilic I, Milosevic B, Krstic J, Šljukić V, Veselinović M, Ivanović N, Janičić A, Jovanovic A, Cvijanovic D, Ladjevic Likic I, Radojevic M, Ćupić J, Dimitrijevic I, Miladinov M, Sekulić A, Nektarijevic D, Pantovic S, Šljivančanin D, Dugalic S (Clinical Centre of Serbia); Đermanović A*, Radovanovic Z, Radovanovic D, Đurić M, Zahorjanski S (Oncology Institute of Vojvodina); Milosavljevic V*, Zdravkovic D, Toskovic B, Marjanović U, Zdravkovic M, Petricevic S, Crnokrak B, Maric S, Sekulic A, Colakovic (Ristic) N (University Hospital Medical Center Bezanijska kosa); Kovacevic B*, Krdzic I, Bojičić J, Sparic T, Ostojic A (Zvezdara University Medical Center).

Slovenia: Cokan A*, Košir JA*, Pakiž M, Potrč S, Kavčič N, Ivanecz A, Horvat M, Jagric T, Kacjan U, Perić I, Timošek E, Plahuta I, Turk Š, Crnobrnja B, Kovačič R, Dovnik A, Al Mahdawi L, Knez J, Grosek J, Košir Božič T, Tomazic A (University Medical Centre).

Spain: Suarez Gonzalez LA*, Busto Suarez S, De La Infiesta García C (Complejo Asistencial Universitario de León); Arencibia O*, Gonzalez Garcia-Cano D, Laseca M, Martin Martinez A, Guijarro Guedes JA, Rave Ramirez AF, Gil Gonzalez Y, Hernández Socorro AM, Ponce Arrocha D, Cruz García A, Mendoza Rodriguez C (Complejo Hospitalario Universitario Insular); Catala E*, Romero Garcia CS, García Trueba A, Georgieva V, Hernández Álvarez AI, Martinez-Perez C, De Valera E, Gascón JJ, Lopez Alcina E, Samper Monton L, Aghababyan K, Valero Tarin A, Salinas Lozano C, Cervera A, Catalá Bauset JC, Ramia B, Marques Peiro F, De Andres J, Iserte Juan SP, Peñalver Gaspar M, Gilabert-Estellés J (Consorcio Hospital General Universitario); De La Rosa-Estadella M*, Fernandez-Colorado A, Serrano-Martin M, Gasulla-Rodriguez A, Juarez-Pomes M (Corporación Sanitaria Parc Taulí); Ossola ME*, Sanz Ortega G, Gómez J, Del Campo M, Corrochano R, Galan Hernandez M, Molano Nogueira FJ, Ibañez Vazquez L, Dziakova J, Zarza Martín A, Infante S (Hospital Clinico San Carlos); Moro-Valdezate D*, Pérez Santiago L, Molina AI, Vinuesa Huesca A, Gadea Mateo R, Navarro-Sanchis L, Aracil-Boigues P (Hospital Clínico Universitario de Valencia); Jezieniecki C*, De Andrés-Asenjo B, Zamora Horcajada Á, Natal Álvarez F, Cuellar Martin LA, Montes-Manrique M, Cabezudo G, Gómez Sanz T, Herranz Arriero A, Sierrasesumaga N, Rodriguez-Lopez M, Ruiz Soriano M (Hospital Clínico Universitario de Valladolid); Sánchez Gollarte A*, Minaya Bravo AM, Guijarro Moreno C, Galvan A, González E, García Ureña MÁ, Robin Valle De Lersundi A, Ruiz-Tovar J (Hospital del Henares); López Campillo A*, Jimenez Toscano M, Salvans S, Jaume Böttcher SM, Godó À, Busquet Raich G, Téllez C, Sánchez García JA, Ribas Ardanuy M, Saavedra P, Sánchez Cabrero A, Clivillé-Pérez J, Durante Escutia M, Rabasa Rodríguez A, Aguilera P (Hospital del Mar); Giménez Francés C*, Ruiz-Marín M, García Escudero D, Valero Soriano M, Ramirez Faraco M, Galán Jiménez S, Alcón Cerro P, Rodríguez Lucas JM, Carrasco Prats M, Peña Ros E, López Sánchez MDP, García Porcel VJ, Tamayo M, Muñoz Camarena JM, Albarracin Garcia R, Molina O, Moreno Sánchez P, Jiménez I, Pastor Perez P, Agea Jiménez MB, Candela Mas D, Artés Artés M, Benavides Buleje JA, González Valverde FM, López-Morales P, Sanchez A, Bobadilla Romero ER (Hospital General Reina Sofía); Allue M*, Ponchietti L, Latorre Tomey R (Hospital General San Jorge); Torres Ramos D*, Jimenez-Gomez LM, Valdés López-Linares S, Moreno Salazar YT, Soto Galán J, Moreno De Alboran C, Prosperi A, Osma JJ, Blanco A, Sánchez Rodríguez M, Valdivielso E, Cejudo Á, Perez Carpio C, Vasques Seabra Águas F, Sánchez Martín E, Pérez-Ajates S (Hospital General Universitario Gregorio Marañón); Macarulla E*, Álvarez Torrado A, Galmés C, Artigot M, Marin Garcia J (Hospital Igualada); Oliver Guillen JR*, López De Fernández A, Fernández-Velilla San-José BF (Hospital Santa Bárbara); Ambrona Zafra D*, Pérez Farré S, Ortega Alcaide J, Codina Corrons L, Sisó Soler E, Gómez Báez FD, López-Soler G, Gutiérrez Pérez E (Hospital universitari Arnau de Vilanova); Aix Molina S*, Vallve-Bernal M, Varona Mancilla A, Aldama Martín ÁM, Casanova R, Sevillano L, Muela JJ, Rodrigo Rodrigo M, Goujon I, Fructuoso Iniesta C (Hospital Universitari De Tarragona Joan XXIII); Pérez-Bertólez S*, Monclus E, Fernández L, Silva Hernández MG, Vicario F (Hospital Universitari Dexeus); Garcia Pimentel P*, García Álvarez R, Bouzó Molina N, Ramiro Á, Calderon Barajas ZA, Sanz R, Xia Z, Rodríguez P, Sarrais Polo C, Martínez López A, Estrada C, Gómez De Castro PE (Hospital Universitario 12 de Octubre); Fernández Martínez D*, García Flórez LJ, De Santiago Alvarez I, García Alonso L, García-Santos G, García González LA, Carrasco B, Silva-Cano DW, Del Val Ruiz P, Martínez Izquierdo G, Corteguera A, Iturbe J, López-Negrete Cueto E, Cembellin A, Ramos Montes C, Ibero Casadiego GP (Hospital Universitario Central de Asturias (HUCA)); Prieto M*, Villalabeitia Ateca I, Pascual Vicente T, Villota Tamayo B, Perfecto A, Mambrilla S (Hospital Universitario Cruces); Ramos Del Moral IL*, Jiménez Carneros V, Pastor-Riquelme P, Franco Lozano A, Alonso-Lamberti L, Lozano M, García-Quijada J, Zhang R, García A, Martín MM, Yeh Ahumada M, Carreño Pallarés A (Hospital Universitario de Getafe); Pérez Rubio Á*, Goran RE, Gómez Diez N, Zomeño Bravo G, Bernal-Sprekelsen JC, Martínez Fernández GS (Hospital Universitario Doctor Peset); Marquina González R* (Hospital Universitario Donostia); Toribio-Vazquez C*, Eguibar A, Perez-Chrzanowska H, Serrano Méndez P, Valderrabano Gonzalez S, Reinoso FJ, Yebes A, Alonso Bartolomé MB, Ayllón Blanco HR, Cristóbal I, Pellicer I, Arenal González R, Zafra Angulo JD (Hospital Universitario la Paz); Duque Mallén V*, Gracia-Roche C, Gascon Ferrer I, Aparicio-López D, Antonio M, Herrero Lopez M, Saudí-Moro S, Gascón Domínguez MÁ, Gimenez Maurel T (Hospital Universitario Miguel Servet); Matías-García B*, Morales Palacios N*, Diez Alonso M, Gutiérrez A, Soto Schütte S, Bru-Aparicio M, Jiménez Martín R, Sanchez Pellejero A, Vázquez Valdés S, Vera Mansilla C, Quiroga A, Mendoza-Moreno F, Diego García L, Serrano Yébenes E, Mañes Jiménez F, Lasa I, Ovejero Merino E, Casalduero L, Allaoua Y, Blazquez Martin A, Córdova García D, Alvarado Hurtado R, Laguna Hernández P, Garcia-Moreno Nisa F, Juara H (Hospital Universitario Principe de Asturias); Aldecoa C*, Tejero-Pintor FJ, García M, Aguado Saster E, Ruiz De Santos E, Bueno Cañones AD, Pérez Fernández S, Alija Garcia MG, Urruchi R, Rioja Garrido R, Madrid M, Bordell A, Arranz Chamorro I, Rodriguez Cañal P, Pacheco Sánchez D, Marcos-Santos P, Veleda Belanche S, Maestro De Castro JL, Blanco García MJ, Laita Jiménez E, Leal L, Vaquero Perez L, Barbosa C, Ramos Carrasco M, Garcia-Saiz I, Rodicio P, Acebes García F, Lizarralde A, Sanchez Diez A (Hospital Universitario Río Hortega); Ferrer-Inaebnit E*, Segura-Sampedro JJ, Alfonso Garcia M, Gutiérrez-Cañadas GGC, Oseira A, Torres Marí N, Loyola J, Villalonga B, Camporro P (Hospital Universitario Son Espases); Pagès N*, Colás-Ruiz E, Cifuentes Rodenas JA, Castro Suárez M, Moll-Amengual R, Mata J, Fernández Manzano J (Hospital Universitario Son Llàtzer); Gómez Pérez B*, Gil Martínez J, Cerezuela Fernández De Palencia Á, Aliaga Rodriguez A, Gómez Valles P, Delegido García A, Gil Gómez E, Montoya MJ, Navarro-Barrios A, Cayuela V, Ruiz Manzanera JJ, Jiménez Mascuñán MI, Gutiérrez Fernández AI, Sánchez Esquer I, Gil Vázquez PJ, Ferreras D, Balaguer Román A, Gómez-Bosch F, Alconchel F (Hospital Universitario Virgen de la Arrixaca); Romero A*, Durán Muñoz-Cruzado VM, García Sánchez CJ, Tejero Rosado A, Ruiz B, Roman MDC, Rubio Fernández JJ, De Jesús Á, Ostos Diaz M, Quintero-Pérez C, Sánchez Marín A, Alvarez Aguilera M, Leal Mérida M, Ager I (Hospital Universitario Virgen del Rocio); Sancho-Muriel J*, Frasson M, Castro B, Guerrero P, Nieto-Sanchez M, Cruz Q, Cholewa H, Hurtado-Pardo L, Plazas D (Hospital Universitario y Politécnico La Fe); Serrano-Navidad M*, Castillo J, Ballestero R, Valbuena Jabares V, García C, Garcia Formoso N, Herrero Blanco E, Suarez Pazos N, Cagigas Fernandez C, Gomez Ruiz M, Benic Yoris YT (Marqués de Valdecilla University Hospital); Merayo M*, Gonzalez Fernandez ME, Alonso Batanero S, Rodríguez Gómez J (Universitary Hospital El Bierzo); Landaluce-Olavarria A*, Estraviz B, Markinez I, Gómez D, Fernández Gómez Cruzado L, González De Miguel M, De Francisco Rios JM, Urigüen A (Urduliz Alfredo Espinosa Hospital); Gómez Del Pulgar A*, Espin-Basany E, Gil-Moreno A, Bellmunt-Montoya S, Dopazo C, González-Suárez S, Blanco-Colino R, Olid Mayoral P, González Antúnez M, Del Barco Martínez E, Bejar Dolcet Y, Gil-Sala D, Marrero Eligio De La Puente C, Sánchez Besalduch L, Barrio Zaragoza M, Lopez Lozano JF, Olaria C, Catalan Sanz S, De Ciria E, Segura Farran L, Montes N, Zanca Gómez JM, Puig Escobar I, Armengol Alsina M, San Nicolas M, Escura M, Sánchez M, Rivas Agudo M, Aalouf L, Maya Salas D, Hernando Marín L, Umpiérrez Mayor N, Sanchez Iglesias JL, Fernández Henriquez Y, Feixa Molina I, Gomez-Jurado MJ, Bebia V (Vall d’Hebron University Hospital).

Switzerland: Racine M*, Sauvain M, Mujcinovic A (Hopital de Pourtales); Bolla D*, Dimitrov Y (Hospital of Langenthal); Arenja N*, Riboni C, Marx JN, Kalisvaart M, Andreou C, Brandts P, Pfefferkorn U, Eisner L, Dietz U (Kantonsspital Olten); Gass J*, Metzger J, Scheiwiller A, Kremo V (Luzerner Kantonsspital); Gero D*, Bueter M, M F, Schulz R (Männedorf Hospital); Mongelli F*, Soave I*, La Regina D, Canonica C, Sabbatini F, Gaffuri P, Spampatti S, Santarelli C (Ospedale Regionale di Bellinzona e Valli); Di Giuseppe M*, Marengo M, Passoni S, Dellaferrera G, Provenzi D (Ospedale Regionale di Locarno La Carità); Bernardi L*, Papadia A*, Cristaudi A, Gasparri ML, Aurora P, Popeskou SG, Christian P, Antonilli M, Scalvi FA, Sevas V, Palumbo G, Maoloni L, Cencetti C, Pagnani G, Hitz M, Dürmüller J (Ospedale Regionale di Lugano); Däster S*, Hess GF, Aegerter NLE, Sedlaczek P, Soysal S, Zeindler J, Angehrn F, Müller L, Taha-Mehlitz S, Haak F, Nocera F, Karli T, Lazaridis I, Tampakis A, Lalos A, Friedrich U, Wiesler B, Varathan N, Frey R (University Hospital Basel).

Turkey: Kurtoglu GK*, Aghayeva A, Turam C, Kurtoglu GM, Maarouf M, Baca B, Ilhan SD, Koyluoglu YO, Seker ME, Ulufi M, Erkaya M, Kılıçkan FDB, Bayrakceken S, Ozmen BB, Togay B, Doğan M, Çetinkaya T (Acibadem Altunizade Hospital); Tuzuner E*, Karahasanoğlu T, Yavuz IE, Bilgin IA, Kekik SS, Turam C, Dönmez E, Dikeç M, Ramoğlu N, Yurtseven N, Takmaz O, Orhun Ö, Karadeniz N, Saylık O, Uğur EM, Ada E, Durbas A, Hamzaoglu I, Uzun K, Gungor M, Gebedek IY, Hayırlıoğlu EÇ, Halıcı M, Ceyhan GO (Acibadem Maslak Hospital); Mutlu AU*, Aytac E, Gulmez M, Capkinoglu E, Yilmaz TU, Ozer A, Keçeoğlu S, Eraslan ZC, Ozben V, Dülgeroğlu O, Saraçoğlu C, Erkan M, Uras C (Acıbadem Mehmet Ali Aydınlar Üniversitesi Atakent Hastanesi); Copur HD*, Dogan NU, Topal E, Demirsoy I, Korkmaz D (Akdeniz University Hospital); Can U*, Buğra D, Sobutay E, Çakıt H, Ergün E, Zenger S, Bilgic C, Gurbuz B (American Hospital); Sünter K*, Gecim I, Sefer S, Inceöz B, Genc MB, Uygun AK, Yücel E (Ankara University Hospital); Barcin A*, Altinel Y, Aktimur YE, Meriç S, Özdoğan K, Sayar AI, Durmaz AG, Çakır I (Bagcilar Research And Training Hospital); Ünlü ES*, Kiran G, Dal A, Ozkan E, Kalkan S, Öncel MM, Çetin Ç, Yılmaz G, Karimi H, Iskurt Y, Yardimci E, Eker M, Balkan AO, Gönül HB, Herdan E, Ertugrul M, Takmaz T, Kilinç SS, Pinar MS, Yıldıztekin F, Coşkun EC, Olgun I (Bezmialem Vakif University Hospital); Ince G*, Isik O, Şen M, Özata R, Eroğlu B, Büyükpolat B, Ishakoğlu G, Özen D, Aktaş MO, Kılıç B, Yılmazlar T, Koçbey MS, Işıklı N, Yıldız RG, Baghirov T, Uygun E, Yugaç M, Keskin E (Bursa Uludag University School of Medicine); Bozkurt BE*, Tas S, Türkmen ÖÖ, Ata B, Alan B, Işık N (Canakkale Onsekiz Mart University Hospital); Göver T*, Bisgin T, Özalp E, Sökmen S, Manoğlu B (Dokuz Eylul Univ. Hospital); Bakar C*, Bozbiyik O, Zorlu U, Zengin NN, Baki BE, Akbulut ES, Tunali S, Kutlu B, Oğuz YA, Küçükateş B, Yildiz E, Sakmar AN, Yoldas T, Akgün ZE, Korkut MA, Çalışkan C, Aksit E, Aksalman EF, Koçoğlu AO, Basci F (Ege University Hospital); Yigit B*, Ilhan O, Liman RK, Uzun M, Ağaçkıran I (Elazig Fethi Sekin City Hospital); Şahin C*, Leventoğlu S, Kubat Ö, Genç I, Oyanik A, Gurbuzer FI, Satilmis N, Cam S, Ekinci M, Gönül M, Chasan D, Zara SGF, Sök Ş, Yalcinkaya A, Acıpınar ET, Timurçin U, Afzal NB, Gillani SHA, Gillani S (Gazi University Medical Faculty Hospital); Yazıcı S*, Cennet O, Suer MS, Kaya Ö, Uslu EZ, Tanrisever A, Yildirim C, Gunenc IN, Gül B, Özkal I, Tileklioglu HI, Korkmaz MA, Şirin I, Özbayrak U, Mıdık A, Domaç E, Karakoç A, Urek S (Hacettepe university hospital); Yıldırım A*, Ugur M, Doğru M, Arslan ER, Ular B, Duymuş ME, Turhan AB (Hatay Mustafa Kemal University Training and Research Hospital); Dogan T*, Ozkan MB, Benek DE, Arslangilay M, Yildirim MB, Yazgan Y, Tıraş HN, Şahin BI, Çapar MA (Hitit University Faculty of Medicine Hospital); Tatar C*, Arı A, Batikan O, Güler M, Naycı AE, Sevinc MM, Şevik H, Oğuz C, Karagülle OO, Idiz UO, Demir A, Çakır E, Cakir C, Doğan S, Yıldız I, Gürlük M, Cengiz MB, Toptaş M, Akay O, Kaya R, Aydin E (Istanbul Education and Research Hospital); Çakıcı MÇ*, Yildirim A, Efiloğlu Ö, Demirtaş B, Iplikçi A, Atis G, Keser F, Culpan M, Çiçek M, Tahra A, Akalin MK (Istanbul Medeniyet University Hospital); Izgiş A*, Soytas M, Okkabaz N, Saylar A, Onaran E, Askin AE, Hashimov E, Bektas S, Sabuncu K, Alagöz AC (Istanbul Medipol University Hospital); Karatas I*, Gok AFK, Simsek E, Altunay ZI, Koç E, Iscan Y, Sormaz IC (Istanbul School of Medicine); Yabaci BI*, Ertürk MS, Nazli M, Saglam IE, Erginöz E, Ozcelik MF, Gokden M, Guler C, Gunaydin FI, Uludağ SS, Ibis B, Bozkir HO, Karaca MB, Ulucay MC, Oner KB, Akbas S, Calikusu FZ, Akcan H, Ugural S, Zengin AK, Ozdemir Z (Istanbul University- Cerrahpasa, Cerrahpasa Faculty of Medicine); Demirbas I*, Guner A, Tepe MD, Kıralı N, Karabağ BC, Pehlivan D, Yuzgec AN, Yıldız B, Akin B, Aktaş M, Karabulut E, Cepe H, Küçükaslan H, Salih SS, Uzun M, Yücesan Ö, Reis ME, Ulusahin M, Aşcioğlu K, Tufan E (Karadeniz Technical University Farabi Hospital); Saleem Q*, Saracoglu K, Ozbilen M, Kale A, Geçici ME, Bulun Y (Kartal Dr. Lutfi Kirdar Training and Research Hospital); Uysal Ö*, Atay A, Şuataman B, Guducu C, Çinar TN, Kul H, Canpolat D, Ipekoğlu M, Aydemir M, Karahan F, Hizli K, Kaya B, Yenen OC, Gümüş B, Turmuş K, Vatansever S, Candan I (Katip Celebi University Faculty of Medicine Hospital); Sucu S*, Balik E, Bozkurt E, Özata IH, Ozoran E, Tüfekçi T, Karahan SN, Sarıkaya AF, Agcaoglu O, Danacı S (Koç University Medical School); Ulutaş ME*, Hasirci I, Arslan K, Metin ŞH, Yılmaz A, Turan E, Şimşek G, Kılınç A, Şahin ME, Ozden S, Bayraktar YA, Maden AS, Şahin A, Acar N, Erşen O, Balcı E, Yaldız H (Konya City Hospital); Guldogan CE*, Ozmen MM, Gundogdu E, Moran M (Liv Hospital Ankara); Celik B*, Sivrikoz E, Karabulut K, Dural AC (Liv Hospital Vadistanbul); Ikizoğlu T*, Aydede H, Yalçın T, Dalkıran A (Manisa Celal Bayar Üniversitesi Hafsa Sultan Hastanesi); Yapici HB*, Uprak TK, Tatar Y, Aksahin Y, Aral C, Cetin SZ, Artvin D, Tatar Z, Karakuş IS, Çatal S, Nalbantoğlu B, Bettar S, Düzyol Z, Demir BC, Kırcalı MF, Taşcı ME, Secil F (Marmara University School of Medicine); Akkaya EN*, Kayılıoğlu I, Hekmatjou B, Çağlayan A, Dadaşoğlu MA, Fergar C, Beden MS, Kayhan E, Arslan I, Koçyiğit GZ, Beyoğlu B (Mugla Training and Research Hospital); Varan C*, Sungurtekin U, Sungurtekin H, Özgen U (Pamukkale University School of Medicine); Çetin B*, Pergel A, Uyan M, Erata E, Aldhahebi E, Aslan B, Kabalı Ş, Köse F, Keşaplı I (Recep Tayyip Erdoğan Üniversitesi Eğitim ve Araştırma Hastanesi); Dulger UC*, Altintoprak F, Akçay M, Cünük ES, Kamburoğlu MB, Küçük IF, Yigit M, Sabuncu E, Harmantepe AT, Aka BU, Baş E, Yavuz Akça T (Sakarya Faculty Of Medicine); Sarı AC*, Colak E, Candan M, Kara ME, Ciftci AB, Yemez K, Uyanik MS, Polat S, Kucuk GO, Ocak S (Samsun Training and Research Hospital); Tosun Y*, Unal E, Ofluoğlu CB, Üstbaş C, Gangal Ö (Sehit Prof.Dr. Ilhan Varank Training and Research Hospital); Sanli AN*, Sayır S, Karslioglu C, Ulusoy I (Silivri State Hospital); Tufan AE*, Demir U, Celayir MF, Baran E, Aksoy AB, Fırat DB, Yüksel A, Güven O, Ertaş I, Kuzu ZS, Köksal HM, Gok M, Görgün MN, Yazici P, Ömeroğlu S, Özdemir BB, Kabul Gürbulak E (Sisli Hamidiye Etfal Training and Research Hospital); Ajredini M*, Cakcak IE, Atakan IH, Göztepe A, Budak O, Aydoğdu EO, Erdem E, Yılmaz C, Özdemir J, Akin B, Alkan D, Cengiz E, Donmez CN, Akyüz E, Ergun YH, Ozer MG, Ak Ç, Bolat F, Kasapoğlu E, Tatlisu A, Yorulmaz Y, Enez A, Ozkan B, Yilmaz ZE, Altin SN, Gunes B, Kaban B, Korkmaz O, Demir IB, Öz GA, Tuluce AB, Turkmen ZN, Yildiz F, Kandil B, Ulkucu A, Kıral G (Trakya Üniversitesi Hastanesi); Yavuz D*, Köksoy ÜC, Kus ZC, Erten MB, Mutlu M, Hökelekli F, Aslan T, Orçan Akbuz Ş, Durmuş E, Gökçek TE (Ufuk Üniversitesi Dr. Rıdvan Ege Hastanesi); Ulgur HS*, Ozkan OF, Karakullukcu HK, Yıldız A, Ceyhan M, Çelik MŞ, Kalın M, Kirkan EF, Demirbilek A, Çelik MN, Karbuş Ö (University of Health Science Umraniye Education and Research Hospital); Demirli Atici S*, Abud B, Erdinç H (University of Health Sciences Tepecik Training and Research Hospital); Ön KB*, Sevinç B, Damburacı N, Altiner E, Özdemir B, Manisalı Ö, Ural G, Topal MK, Can T, Ercan S, Kaynar S (Usak University Medical School); Emanet O*, Javadov M, Demir UB, Erdem A, Demircan F, Yelmenoğlu M, Seçen E, Alkışer B, Arık F, Kaya EE, Akkad D, Karaçam D, Karacam U, Yücel MA, Hatipoğlu A, Mumcu N, Karima O, Dal MB, Aljobbeh M, Montaser J (Yeditepe University Hospital); Kara MA*, Gultekin FA, Deniz O, Kavak N, Kum B, Ecir MM, Yavuzer YG, Sezgin Z, Koçdemir U, Yirmibeş B, Atalay Ş, May A, Varna S, Aydoğdu I (Zonguldak Bulent Ecevit University School of Medicine Research and Training Hospital).

UK: Abdel-Fattah A*, Ramsay G, Bin Badekruzaman MA, Karvelyte M, Siamisang M, Clunie S, Mikalauskaite M, Nessa A, Joshi C, Jodeh N, Zafar R, Miller J, Basharat R, Singh T, Weston O, Loy R, Tsoi EHC, Chandiramani A, Sumarlie RJ, Mitchell L, Yousif M, Qadir M, Gannon M, Tong EYH, Tsang J, Elmarghani M, Kaczmarek J, Luangboriboon J, Mcewen R, Tse B, Wong MC, Wong ZX, Diffley T, Jaruwattanapradid O, Ng N, Mchaffie L, Wright C, Joyce D, May V, Emmanuel S, D’costa A, Kumar A (Aberdeen Royal Infirmary); Hanganu C*, Gourgiotis S, Townson A, Williams CY, Baines E, Smith L, Elks N, Simon N, Chintapalli R, Banerjee A, Bhogle S, Ryan B, Maghsoudi D, Clifton E, Nott T, Vickers H, Stewart GD, Zhong ZX, Ioannou K, Ne CKH, Conci R, Fitzsimons-West E, Ling XL, Patel R, Sanghera R, Dokubo I, Seago I, Fairhurst O, Amin B, Aniq M, O’keeffe E, James S, Chowaniec M, Rutigliani L, Hu M, Ferreira AF, Kalogeropoulou M, Hui G, Coakham L, Healy S, Gilanliogullari K, Nishimura G (Addenbrooke’s Hospital); Choi M*, Lunevicius R, Aje D (Aintree University Hospital); Mcnicholas R*, Newman C, Bollen E, Kumar A, Ferreira Xavier I, Baloch LG, Koduah-Sarpong NA, O’sullivan G, Saseendran S, Bin Sahl A, Murphy E, Hussain H, Nicholls A (Airedale General Hospital); O’dolan S*, Gidwani A, Patterson E, Loughrey L, Donaghy K, Hana K, Monaghan M, Ni Shandair S, Kulasingha N, Gormley N, Mcglinchey M, Dunlop E, Dunwoody B, Lian SH, Jayasangaran SC, Logue E, Goswami P (Altnagelvin Area Hospital); Ann Jacob P*, O’kane C, Ab Razak AS, Govender K, Mccann EJ, Perry S, Mckendry S, Pachchigar A (Antrim Area Hospital); Singh A*, Dindyal S, Pendyala S, Venkateswaran V, Alhoussan L, Feil F (Basildon University Hospital); Hughes T*, Gerçek Y, Choudry M, Haidar O, Rujeedawa T, Speirs T (Bedford Hospital); Odendaal J*, Chu J, Shahab A, Ranjithkumar S (Birmingham Women’s Hospital); Pillai Rajendra Prasad V*, Linn T, Clark J (Blackpool Victoria Hospital); Sharma M*, Lockwood S, Chawla S, Deshpande R (Bradford Royal Infirmary); Long N*, Rees J, Kobetic M, Fawcett-Till D, Ferris O, Harrison G, Hibbs T, Tsang CTW, Hurley L, Jichi T, Badrinath C, Applebee L, Ecott K, Sullivan T, Piecha B, Jagadish A, Sajjad Z, Griggs R, Joyce S, Spurgeon R, Ali Khan M (Bristol Royal Infirmary); Hobrok M*, Roberts R, Mckenna J, Davies D (Bronglais Hospital); Eaton P*, Bishop L, Magaway S, Denholm K, Doyle S, Deshpande R, Salter M (Calderdale Royal Hospital); Chan V*, Mockford K, Heinz J, Ahmed I, Coverdale M (Castle Hill Hospital); McKillen RJ*, Maguire M, Mclees N, Lau J, Mcguigan L, O‘Neill A, O’Hare D, Beggs C, Owolabi O, Hunter J (Causeway Hospital); Kinross J, Kotecha J, Doherty R, Patel V, Wagner E, Hodges L, Hassan H, Sribaskaran K, Pouris K, Jain S, Kim AJ, Gill S, Nand S, Toutouza O, Joshi A, Thornley L, Chen R, Ogunjimi M, Mahmood A, Chong R, Yanai M, Bae D, Dhaliwal J, Lovejoy A, Akhbari AR (Charing Cross Hospital); Mayor N*, Kontovounisios C, Yap SHK, Damarla M, Ooi B, Ng MA, Vargas Zhang A, Limbu S, Nyamakope K, Agarwala Y, Zingas N, Cleasby C, Smyth E, Drmota J, Yang QZC, Vedi C, Samarendra S, Nayak V, Rajendra S, Choudhry M, De Stadler K (Chelsea and Westminster Hospital); Bandyopadhyay S*, Bond-Smith G, Collart O, Baffour-Awuah K, Shah R, Mcnamara J, Tadikamalla G, Wilson B, Cossins C, Li H, Ismail L, Soleymani Majd H, Chang E, Jallow A, Baldelli S, Alberti P, Grant O, Doody M, Borawska Z, Bowman A, Clay H, Petit Y, Kimani JG, James M, Hussain SF, Nezhentsev A, Emmerson M, Vijjhalwar R, Shaw G, Holmes C, Ying Y, Farache Trajano L, Anis A, Dequaire J, Hunter A, Danvers R, El-Nemr M, Hammett C, Pikoula M, Thompson S, Lander B, Dierksmeier S, Sadeghi N, Suribhatla R, Ahmed R, Pandit S, Thornton W, Thornton-Swan T, Johal A, Khan M, More A, Tilahun Y, Hauperich A, Gidda R, Vorley I (Churchill Hospital); Khan Z*, Sintler M, Van Den Broeck A (City Hospital); Georgiou M*, Eardley N, Salem H, Yemparala S, Whittle L, Jagger C, Turner K, Vernon L, Aldred K, Manokar P, Moore A, Mistry N, Zgliczyńska J, Haylett C, Adeyanju A, Headford E, Khanna R, Deef M, Durkin T, Trayling E, Cowell R, Khan Z, Turner A, Noureldin K, Down W, Cyril A, O’halloran C (Countess of Chester Hospital); Shirke M*, Epanomeritakis M, Karnati A, Sreejith G, Sinton C, Oliver L, Ali Azamatullah Khan HF, O’kane E, Trouton J, French B, Campbell I, Curran R, Brines C, Mcdermott A (Craigavon Area Hospital); Yang N*, Vig S, Chowdhury S, Valecha R, Lau R, Nafis M, Thavanesan S, Marks J, Akaje-Macauley L, Patel N, Beard E, Nanda A, Mills C, Cheung A, Amalendran J (Croydon Hospital); Todd L*, Smolarek S, Warrener T, Sen S, Mudehwe A, Khan S, Wisden B, Lau G, Schanzer L, King Z, Giridhar K, Reed-Embleton M, White L, Filippidis P, Lee-Smith L, Carless O, King C, Herriott M, Saju N, Tin Maung YM, Deligianni A, Mumtaz A, Khan S, Hill H, Sahdev S, Thomas I, Shah A, Saji S, Hargreaves J, Khan R, Tharani A, Ullah R, Ludgate J (Derriford Hospital); Shrestha S*, Bota E, Abdullah A, Allison M, Patel Z, Clark A (Diana, Princess Of Wales Hospital); Suchett-Kaye I*, Stubbs B, Holmes B, Santaniello A, Watkinson T, Crimmins A, Gupta MK, Abraham S, Bellringer A, Clowes R, Chatzopoulou D, Kiran A, Eid J, Rao N (Dorset County Hospital, Dorchester); Caterson J*, Soggiu F, Malik H, Jose I, Kavallieros K, Rizk C, Thummala B, Li K, Goel A, Pantula R, Toh J, Fu MX, Ho CC, Vivek K (Ealing Hospital); Owen E*, Day A, Jamieson A, Hassani H, Thavarajah T, Vivekanandan M, Muneer A (East Surrey Hospital (Redhill)); Chauhan M*, Veeramootoo D, Ali S, Dosanjh J, Newing F, Jose E, Chauhan H, Kulshreshtha A (Frimley Park hospital); Thomas E*, Patel P, Chhabra R, Sajan B, Ragavan S, Hariharan R, Lam I, Fleet B, O’hara N, Wright A, Reddy V, Darweesh H, Khan A, Handa S, Kewada D, Rana F, Williams H, Bombieri F, Shah N, Pestotnik Stres D (Furness General Hospital); Menon A*, Selvam L, Pusok A, Street C, Zohdy M, Aguirrezabala Armbruster B, Okoye I, Potts L, Fayyad M, Bajaj K, Alfa H, Sivakumar N (George Eliot Hospital); Duncan N*, Roxburgh C, Huang L, Lukito K, Huang X, Baig S, Chan CY, Philip R, Shaheen L, Dodds J, Yeow HX, Devindran M, Sia ZC, Park JH, Furze B (Glasgow Royal Infirmary); Yung N*, Vipond M, Jones M, Asekun F, Dembinska-Kenner A, Saleh R, Larkai E, Harris J, Cherian M, Louden H, Bisbinas V, Behl A, Hughes R, Smith R, Zaman Z, Hoskyns O (Gloucestershire Royal Hospital); Carlton H*, Thorn C, Tourky M, Tan J, Sen K, Elsey J, Bevan J, Ko N, Kalidindi S (Great Western Hospital, Swindon); Rooney M*, El-Boghdadly K, Zafar S, Wyncoll A, Morillon P, Sennaraj M, Mahfouz H, Afzal A, Sibal R, Suji T, Headon H, Abdul A, Ahmed S, Mcnally-Reilly JP, Omran K, Mulcahy C, Aftab M, Haghighat Ghahfarokhi M, Farhangi M, To WY, Ho M, Patel T, Siu A (Guys and St.Thomas’ Hospital); Choudhry H*, Huntley M, Hussain Z, Wong G, Maguire R, Khan M, Potts A, Ahmed M, Ramesh G, Kolade O (Harrogate District Hospital); Siddique MH*, Griffiths E, Qureshi M, Hoque M (Heartlands Hospital, Birmingham); Laulloo S*, Das K, Waheed N, Tang TYT, Ahmed R, Habib R (Hereford County Hospital); Vyas R*, Watson S, Theodoropoulou K, Siu M, Yu N, Islam N, Burnett J, Besso-Cowan A, Hirani D, Zsolnai I, Chen YK, Dupere O, Keitley L, Park C, Verma I, Chan J, Tavner J, Nicolaou M, Lee NYS, Hegy R, Lee RBY, Yi Lun KYL, Reyes D, Dong S, Thornton J (Homerton Hospital); Eaton P*, Bishop L, Magaway S, Denholm K, Doyle S, Deshpande R, Salter M, Weir M, Gibbs A (Huddersfield Royal Infirmary); Al-Shaye A*, Alwahid M, Tait A, Smith S, Doye A (Inverclyde Royal Hospital); Law KL*, Groot-Wassink TG, Geller G, Seebah K, Cox O, Kalogeropoulou M, Shetty A, Oh DCJ, Lee E, Packham B, Aarons M, Saadeh K, Mui J (Ipswich Hospital); Huynh R*, Eid M, Honey N, Kaur J, Hand R, Lai L, Koubaesh C, Maqsood-Shah H, Suresh J (James Cook University Hospital); Collart O*, Bond-Smith G, Shah R, Mcnamara J, Tadikamalla G, Wilson B, Cossins C, Li H, Soleymani Majd H, John L, Chang E, Kimani JG, Jallow A, Bandyopadhyay S, Baldelli S, Grant O, Doody M, Borawska Z, Bowman A, Foster C, Clay H, Petit Y, James M, Hussain SF, Nezhentsev A, Emmerson M, Vijjhalwar R, Shaw G, Holmes C, Ying Y, Farache Trajano L, Anis A, Dequaire J, Hunter A, Danvers R, El-Nemr M, Hammett C, Pikoula M, Thompson S, Shah R, Dierksmeier S, Sadeghi N, Lander B, Suribhatla R, Ahmed R, Pandit S, Thornton W, Thornton-Swan T, Johal A, Khan M, More A, Tilahun Y, Hauperich A, Gidda R, Vorley I, Lewis-Orr E (John Radcliffe Hospital); Reilly S*, Bhatia A, Goves K, Blesson M, Purser L, Cobb L, Sarker MH, De Sousa N (Kettering General Hospital); Syed S*, Rajendran N, Tan Sue Wei J, Navakumar G, Butnari V, Senthilkumar M, Chew ZQ, Zekaite L, Paranietharan S, Haque M, Balenthiran S, Chin KJ, Ahuja S, Moothathamby T, Moniati F, Kong YZ, Lee EJH (King George Hospital); Sharma L*, Found P, Sivakumaar Y, Senior H, Simeen N, Arora A, Chu A, Mizori R, Beazer T, Patel N, Dudziak JM, Squeri T, Weston F, Zhou M, Van Ross J, Tian S, Bedford J, Lam R, Karami Tireh Shabankareh F, Donkin Everton H, Wilson K (King’s College Hospital); Richens A*, Bragg D, Akbari A, Awodiya A, Osula SA (King’s Mill Hospital); Gerges F*, Gerogiannis I, Daniels L, Seth S, Baxter Z, Nour E, Fitchford L, Perrott C, Abuelgasim M, Flanagan J, Gillani S, Lewis H, Dunscombe O, Steven Goodwin Moughton P, Mahmood A, Hirniak J, Moses A, Kapsampelis P (Kingston Hospital); Ahmed A*, Burke D, Anderton C, Lee E, Conley M, Khan L, Surti R, Waseem F, Sharma M, Imran A (Leeds General Infirmary); Motiwale R*, Singh B, D Sa S, Charuvila S, Lorgat MZ, Chowdhury R, Gogineni P, Finney A, Tzortzi De Paz A, Panesar S, Sidki A, Warwick C, Kadambande S, Patel J, Rao A, Sharma A, Ramewal S (Leicester General Hospital); Ramesh P*, Norwood M, Mehta P, Bhatia M, Tiwana S, Foote E, Rushton F, Asi S, Notaney R, Sinha R (Leicester Royal Infirmary); Vibhishanan J*, Gupta S, Conci R, Chintapalli R (Lister Hospital); Ahmed S*, Sagar J, Ragge N, Rizvi SS, Ashraf M, Cirocchi N, West R, Obiri-Darko E, Rai Y, Hussain S, Ul Ain N (Luton & Dunstable University Hospital); Amir FN*, Smart C, Melomud R, Saad D, Bengeri S, Kuna S, Chaudhary M, Olanite OG, Khan F, Nausheen S, Dodds R (Macclesfield District General Hospital); Boyden H*, Donnelly D, Adegboye O, Osborne L, El-Barraj S, Shukla J, Raza N, Khalid A, Agrawal D, Kyaw KT, Borhan S, Pangrazi G, Ioannou I, Ahmed AN, Savvides G, White M, Puthur SJ (Manchester Royal Infirmary); Bridgewater S*, Waraich N, Bryce R, Khosla R, Penhaligan L, Desai A, Ehsan Z, Mostafa O (Manor Hospital, Walsall); Kamel Y*, Keeler B, Rajivan R, Omar V, Gamadia S, Rana D, Srikumar S, Heidari A, Ahsan Akhtar A (Milton Keynes University Hospital); Tarafdar A*, Barry J, Davies D, Curr A, Jimenez-Reid I, Mckenna J, Garry E, Lallmahomed N, O’leary K, Barker E, Jones DR, Tabatabai R, Garg J, Henshaw E, Abdulshafea M, Prabaharan S (Morriston Hospital); Wiles H*, Bamford R, Blest F, Acquah L, Simpson H, Guerrero Enriquez J, Pringle H, Shahid M, Wharf O, Kumar M, Buadooh K, Hanson M, Mutanga I, Hardy A, Usman H, Shams S (Musgrove Park Hospital, Taunton); Schottler N*, Garg A, Sarodaya V, Ali S, Cullen I, Pregil L, Fernandes Silva Ramos M, Hao Y, Das Chagas E Silva AC, Sellahewa S, Li J, Cox J, Sinha P, Lee IJJ, Voniati E, Phillips H, Mooney O, Sockett M (Newham University Hospital); Patil P, Elsllabi M, Chan WKM, Grimble L, Richardson H, Johnston A, Kouli T, De Sousa JFM, Goh YK, Grant C, Martin L, Peh ZH, Yap T, Tang ETW, Lai HYM, Berry T, Rokia M, Tennant E, Lee I, Rahman MJ, Kamal A, Ali S, Hau HX, Harten I, Eloofy M, Caldwell C, Leckie R, Tasnim F, Ravintharan K, Mcauley J, Cameron F, Veerni RB, Looi KY, Ismaili A, Gresz R, Chong V, Tan LK, Singh K, Ashburn S, Shantha Kumar R, Kamal M, Kamal H (Ninewells Hospital); Aziz I*, Stather P, Mylvaganam L, Deliyannis E, Tompkins B, Sikorski E, Harris F, Sanders I (Norfolk and Norwich University Hospital); Fakhrul-Aldeen M*, Cross K, Tahir A, Yim P, Mayne I, Mahapatra S (North Devon District Hospital); Ng CE*, Ortega A, Supparamaniam S, Bowman M, Sadler M, Rogger M (North Durham University Hospital); Gendia A*, Ahmed J, Sivakumaran P, Brick C, Ansong E, Ha H, Sharma A, Wilson N, Manavi B, Zeze L, Gupta NP, Al-Hassani S, Williams C, Ramesh M, Shukla B (Northampton General Hospital); Drye E, Hussain H, Ghatauray E, Tan ACW, Adamu-Biu F, Arora J, Mcallister T, Fairclough S, Economou A, Utulu S, Fung KW, Epanomeritakis IE, Zaman SS, Olszewski E, Ballesteros P, Okpii EC (Peterborough City Hospital); Walkeden E*, Macklin C, Jamil S, Zhen S, Ahmed O, Saravana Kumar A, Uddin H, Hakim R, Mossanen Parsi M, Awoloto O, Rowe K, Mashadi R, Mohammed Z, Chong LC, Fairlie-Vogt S, Russell O, Ozarek W, Little A, O’farrell C, Miller R (Pinderfields Hospital); Froud J*, Thippeswamy K, Thomas R, Fitzpatrick A, Yoganathan S, Dunlop M, Ngai K, Liddell-Lowe T, Barrow A (Prince Charles Hospital, Merthyr); Sharma L*, Found P, Mizori R, Rabas S, Al-Housni R, Zhou M (Princess Royal University Hospital); Marton A*, Chen P, Hasan H, Chan CFB, Win Mar WW (Queen Elizabeth Hospital King’s Lynn); Kisiel A*, Griffiths E, Yusuf M, Sinha A, Blenkinsop J, Rawlings K, Chaudri T, Westland G, Umar H, Lee S, Cherian J, Gurhan Z, Chaudri T, Sheraz MS (Queen Elizabeth Hospital, Birmingham); Khan DMI*, Kucukmetin A, Malone D, Betts C, Ali M, Giblin M, Nowicki L, Korompelis P, Farley O, Fouweather M, Ioannides C, Yilmaz H, Landais R, Ngeyu EX, Erinjeri C (Queen Elizabeth Hospital, Gateshead); Badran D*, Glen P, Aamir J, Aziz A, Su PL, Choong I, Reece M, Kulasekaran A, Tengku Saifudin TNS, Luckhurst J, O’hare R, Smith R, Siu YT, Kwok PH, Sood V, Imran A, Fatima K, Munir Z (Queen Elizabeth University Hospital, Glasgow); Kisova T*, Rajendran N, Islam F, Teehan E, Sadik H, Patterson U, Rahman N, Harrisrhaj H, Govardhan D, Rajesh S, Mumtaz Z, Ghori OS, Nawaz H, Lysomirski A, Pikoula V, Qadir RMAB, Jones A, Chiang Y, Morsy H, Pazaiti C, Asardag AN, Ali I, Althonayan J, Badri A, Evans J, Chung E, Harriss L (Queens Hospital); Sinan L*, Maxwell-Armstrong C, De A, Altman R, Varghese F, Chua W, Ahmed S, Javed M, Aljubure R (Queens Medical Centre, Nottingham); Bradley A*, Moug S, Kemmett OEH, Underwood I, Fitzpatrick I, Wong LS, Vakeesan B, Potter K (Royal Alexandra Hospital, Paisley); Varghese T*, Daren S, Hazrin Fazail A, Farajzadeh Asl S, Nautiyal H, Saadeh A, Vial I, Karimi R, Akthar A, Egiz A, Masood R, Qadri A, Chawla T, Yoon H (Royal Blackburn Teaching Hospital); Liu X*, Battersby N, Davies E, Chhetri R, Mclean R, Leaper E, Taylor C, Nicolaisen C, Cochetti L, Zoumi EA, Annable D, Thomas H, James-Knights Z, Lee-Kim-Koon C, Akpinar E, Coelho MJ, Darke E, Morris T, Mayer F, Forbes J, Gold S, Sinha H, Batchelor E (Royal Cornwall Hospital); White L*, Lund J, Forrest S, Dial H, Chishty F, Oliver M, Meyer J, Gokhare Viswanath N, Ammar A, Lopez A, Minhas N (Royal Derby Hospital); Sunny N*, Haynes K, Alexiadis A, Doski S, Arbuthnott R, Memon R, Bukhashem K, Chaudhary A, Sheik-Ali A, Sebastiao A, Taylor C, Clarke E, Misztal S, Raja D, Chui SK, Khalil I, Metsel A, Whelan M, Forrester S, Oboh O, Bayley-Skinner B, Eastwood R, Akapo E (Royal Devon and Exeter Hospital); Jamshaid M*, Osborn G, Woods R, Wooler O, Chumley S, Cotton E, Jarrett N, Amanda N, Johnson J, Quhill M, Al-Ani M, Kandanearachchi S, Khan S, Barrow A, Dunlop M (Royal Glamorgan Hospital); Williams G, Phillips S, Faryad F, Hughes A (Royal Gwent Hospital, Newport); Wong Ching Hwai S*, Johnston C, Guest R, Kaddouri A, Ernst L, Szasz A, Gafrey C, Loy G, Roberts E, Baker S, Davies A, Khan Z, Leonidou M, Chow JK, Murugan R, Baker A, Burns R, Foo YC, Sikora F, Chong L, Chan Ah Song C, Cosgrove C, Reeve-Chen O, Ma J, Mccolm L, Zhang MK, Hussain F, Ng XY, Ingabire B, Wagner B, Bain T, Kovacs R, Small E, Lam S, Yao L, Ho L, G Paremes Sivam H, Dahal K, Stanley A, White M, Hayois L, Thillai M (Royal Infirmary of Edinburgh); Tay T*, Davies E, Kamaruzaman N, Robinson L, Sherrington R, Abid Sohail Z, Lawrence S, Sohail A, Brown D, Parker S, Ohr M, Jasniak D, Crowther B, Jadoon F, Melo LJ, Amin H (Royal Lancaster Infirmary); Lim RQH*, Thaha MA, Bath M, Saadqain SA, Benallal Y, Jin Z, Jackson A, Kuri A, Malik M, Jos H, Goh ET, Paraskevopoulou ME, Kythreotis A, Naim Ahmed Y, Glynou SP, Rehan H, Pereira Pai A, Georgiannakis A, Dworschak N, Cho S, Chow BJ, Khan Y, Enthoven T, Malik A, Latif I, Lakhani AH, Rahman N, Morgan K, Gkolia S, Khalessi N, May PLZ, Lwin A, Kanesaratnam P, Tudgee M, English R, Hu K, Pathanchaly N, Chien DCS, Holloway M, Gao K, Lekka G, Srinivasan R, Zafar S (Royal London Hospital); Peris MR*, Keiarash K, Kanesan H, Chong B, Kobus M, Dominic D, Do DA, Dennis D, Frankland S (Royal Preston Hospital); Shah V*, Youssef B, Sadia U, Hassouneh A, Jaipersad A, Chen T, Fulford M, Dawson A, Kapur K, Jha T, Zope P, Wilkinson M, Bhatnagar V, Knight J (Royal Stoke University Hospital); Madhuri TK*, Katsiari T, Pasternak-Albert N, Moussa E (Royal Surrey Hospital, Guildford); Emms L*, Lamah M, Batool Z, Sabesan A, Islam S, Baig M, Dave M, Liu X, Stuart L, Harvey S, Dam V, Ezuma P, Lee J, Suntharalingam S, Singh S, Preston P, Rickard F, Gupta T (Royal Sussex County Hospital); Madden S*, Jones C, Gidwani R, Wang X, Mcconnell M, Hanna S, Mcgettigan A, Brown K, Cios A, Ward-Bradley C, Hurrell O, Paice V, Mutsonziwa N, Pogoson S, Coulter R, Corry J, Mcclean L, Keenan F, O’dolan S (Royal Victoria Hospital, Belfast); Plonkowski A*, Brady R (Royal Victoria Infirmary, Newcastle); Ooi R*, Iles D, Guillemot LS, Low C (Saint Marys Hospital); El-Barraj S*, Epstein J, Pressler M, Ioannou I, Soliman N, Ktayen A, Macconnachie A, Menendez Lorenzo J, Tooke J, Noman K, Wilkins L, Warner-Levy JJ (Salford Royal Infirmary); Evans R*, Torrance A, Fear C, Vidis Humphries H, Velayudham G, Jefferson-Pillai Z, Brady J, Oza R, Singh D, Hamid A, Hartop P, Shojaie P, Mano J, Rajah T, Cabdi YMC, Singal A, Afzali M (Sandwell General Hospital); Lee JM*, Bandyopadhyay D, Bennett J, Mendenhall P, Millett A (Scarborough General Hospital); Davidson S*, Kallam R, Pishia M, Butler H, Isidore S, Boutsias D, Bryce K (Scunthorpe General Hospital); De Rancourt T*, Barry J, Henry H, Aspinall A, Smith L, Conway C, Shanmugathas H, Huang C, Doherty M, Ahmed K, Chung T, Benson T, Habiba A, Singh Bhangu J, Bingor B, Fice L, Premanandhan H, Mckenna J, Jimenez-Reid I (Singleton Hospital); Hoque M*, Griffiths E, Loughran J, Laskowski M, Mehmood A (Solihull Hospital); O’kane R*, Mullan M, Mcclenaghan R, Kettyle G, Hosty-Blaney F, Chan XHF, Sunny S, Kaur M, Vose S, George V, Killoran D, Doherty R (South West Acute Hospital); Zagorac Z*, Pullyblank A, Ismail R, Anderton N, Dwyer-Joyce L, Morgan R, Zhang N, French B, Cox H, Tetro J, Clarke A, Shaw K, Iau R, King E, Patel S, Ovakimian M, Zahid A, Rowley G, Tanner R, Davoudi K, Shear B, Xu M, Karan K, O’reilly A, Ahmed S, Iskrenova Kirilova T, Ezekiel A, Shapland M, Mitra M, Krum T, Higginbotham G, Winayak R, Mustow J, Yorke L, Gibb C, Ilyas A, Pound M (Southmead Hospital, Bristol); Abouharb A*, Burke D, Khan A, Aimar K, Bennett J, Kerman-Fiore E, Rao S, Dayal A, Chawla S, Wadey L, Smith C, Desor K, Surti R, Gul Z, Gourgiotis V, Kaselampao LM, Nguik C, Agrawal R, Liaw QY, Mckeown E, Hassan A, Krishnan K, Glover HO, Devassy M, Ogunyanwo D, Dickenson G, Elder G, Valdez G, White B, Hamilton I, Shah S, Sinha S, Punwani K, Peat I, Hayat M, Khalid N, Frankel E, Macgowan H, Braka A, Byfield A, Brooks J, Kashif A, Archer R, Akkaya S, Sood M, Akther S, Birkett O, Kadri T, Abdulmula S, Psefteli P (St James University Hospital, Leeds); Chadha S*, Reese G, Mark C, Asunramu M, Hess T, Mehta S, Tsang C, Syed K, Alocious A, Steinruecke M (St Mary’s Hospital IOW); Ashley M*, Newton R, Wainwright J, Ayathamattam J, Wong H (St Richards Hospital (Chichester)); Elahi Z*, Fleming M, Ali T, Lloyd D, Brooks K, Kwon C, Mckerrell A, Jamadar T, Barcelona MD, Polluk D, Kwon J, Henry F, Ayirookuzhi S, Mwipatayi M, Cheruiyot M, Adam S, Cruickshank T, Gollub K, Revell M, Taiwo S, Atchade A, Harewood A (Stoke Mandeville Hospital); Niina A*, Bosanquet D, Mckeon-Williams O, Szakmany T, Badhams E, Christensen N, Sammut J (THE GRANGE UNIVERSITY HOSPITAL); Tay N*, Pollok JM, Raptis D, Varcada M, Ganesananthan S, Chandrasekar R, Deshpande A, Ghoora LP, Hogg G, Staubli S, Banerjee M, Sunil Menon S, Sanz Pena A, Rana R, Mohamed A, Crabtree P, Flower A, Banerjee A, Talabi Y, Bishop N, Kupfuwa N, Ilangovan D, Qureshi H (The Royal Free Hospital); Minty I*, Baron R, Greasley L, Birkenhead P, Baron R, Shahid M, Rajasivam N, Chu WL, Punnoose N, Sundaresan V, El-Galley M, Prasansapakit K, Mitra S, Garlick M, Prakash M (The Royal Liverpool University Hospital); Gao H*, Parmar C, Lee F, Aich A, Ismail A, Kirupaharan M, Khan S, Patel K, Mclaverty D, Hammerton G, Walford H, Roberts H, Cautivo P (The Whittington Hospital); Abdel Kader H*, Kimble A, Manaf K, Mackonochie R, Mitchell V, Ghaznavi S, Taheem A, Firth E, Chandler H, Eldessouki K, Fyfe J, Bhat M, Cavlin J, Pearce E, Patterson S, Johnson C, El-Badawi K, Gariballa M, Hale E, Younis H, Brackenbury E, Chappell A, Poulton T, Dawson E (Torbay Hospital); Murray A*, Mcallister I, Hassan A, Cunningham B, Chan-A-Sue T, Rajpal N, Keane P, Walsh K, Mcgeoghan L, Neeson M, Brown H, Adams SM, Ladha S, Walsh A (Ulster Hospital); Alradhawi M*, Tarighi R, Miah MI, Dawas K, Mojadady A, Priestman-Degano A, Vellore Sasikumar SK, Suresh B, Fornasiero M, Csvila D, Kumar A, Adil ST, Adil HS, Kataria U, Jaibaji R, Patel N, Goch A, Quaye P, Gupte A, Mustafa H, Otti V (University College Hospital); Dewantoro D*, Ali M, Sood M, Wright H (University Hospital Ayr); Abusheba L*, Wong L, Docksey M, Abdinasir A, Karim MO, Kolaityte V, Vasileiadou Pelling M, Abdel Basit A, Pearson E, Hughes E, Millington AS (University Hospital Coventry and Warwickshire); Taylor L*, Borg C, Jagic K, Waheed A, Singh K, James V, Chowdhury T, Saxena A, Georgiou A (University Hospital Lewisham); Jones T*, Carpenter C, Hughes I, Aloul Z, Hanna-Davies C, Jacob A, Puthiyakunnel Saji A, Qureshi A, Ulfin Z, Dunlop M, Woods R, Hill N, Mohamed N, Vora A, Asharaf H, Maye E, Arif U, Srinivasan S, Prasad S, John B, Shivanand V, Singh A, Zahid A, Stewart E, Raketla M, Kokoricha O, Karwa D, Crisp J, Shehadeh R, Lee J, Webb B, Lepping S, Yusuf F, Pak E, Soo B, Pemmasani P, Goyal S (University Hospital of Wales); Binti Azad Bashir AB*, Komolafe S, Naeem A, Anbananthan HR, Au Yong LK (University Hospital Wishaw); Mookerjee S*, Ward N, Aniq M, Davis J, Al-Sukaini A, Anthony J (West Suffolk Hospital); Boyle J*, Laird A, Speake D, Beagan C, Stewart S, El-Koubani O, Chan R, Viswanath M, Janssens S, Shah I, Nguyen V, Mckee E, Ng CW, Balać M, Suresh Kumar DI, Seeva Balan S, Kirk R, Miles B, Kovacic LA, Collins VG, Low J, Sim W, Chua SJ, Narayanan N, Lee TY, Lo CMN (Western General Hospital, Edinburgh); Chauhan M*, Elkafsi J, Rasul S, Yammine R, Sattar Z, Dixit D (Wexham Park hospital); Rahman N*, Davies J, Dindyal S, Osborne L, Nip L, Wilding de Miranda N, Stevens A, Al-Diri B, Lim A, Kallikas G, Kim Y, Anjum M, Jeyapahan P, Hakim M, Singh S, Jamileh E, Sohrabi C, Baek SH, Sadik N, Mohammed A (Whipps Cross Hospital); Aje D*, Clifford R, O’halloran C, Mahajan K, Darke N (Whiston Hospital); Lloyd S*, Mlotshwa M, Melhuish K, Derex-Briggs J, Bell M, Varma P, Fray K, Garg Y (Worthing); Datta K, Mantel-Cooper T, Goodfellow M, Kazi B (Wrexham Maelor Hospital); Sievers N*, Telford K, Almansoor ZR, Craddock K, Tan JL, St John L, Singhania A, Dosani S, Mughal S, Bokhari N, Brooks L (Wythenshawe Hospital); Laid I*, Lala A, Ong CS, Wakefield S, Phillips S, Unwin H (Ysbyty Gwynedd, Bangor).

## Supplementary Material

zrag125_Supplementary_Data

## Data Availability

The data underlying this article will be shared on reasonable request to the corresponding author.

## Data sharing

Data sharing requests will be considered by the writing group upon written request to the corresponding author. De-identified participant data and/or other prespecified data are available, subject to a written proposal and an agreed data sharing agreement.
