# Supplementary material for: Disparities in pathways for anaemia care and recovery among women after major abdominal surgery: secondary analysis of a prospective cohort study in 29 countries
Source: BJS Open. 2026 Sep 29;10(5):zrag125. doi: 10.1093/bjsopen/zrag125 (PMC13623008; doi:10.1093/bjsopen/zrag125)
Supplement: Supplementary file 1 [file zrag125_supplementary_data.docx]

**Disparities in pathways for anaemia care and recovery among women after major abdominal surgery: secondary analysis of a prospective cohort study in 29 countries**

*STARSurg Collaborative*

**Corresponding authors:**

Mr Sivesh Kathir Kamarajah, NIHR Doctoral Fellow, Department of Applied Health Sciences, School of Health Sciences, College of Medicine and Health, University of Birmingham, Birmingham, United Kingdom. Email: [s.k.kamarajah@bham.ac.uk](mailto:s.k.kamarajah@bham.ac.uk)

and

Professor Dharani Hapangama, Professor of Gynaecology, Department of Health and Life Sciences, University of Liverpool, Liverpool, United Kingdom. Email: Dharani.Hapangama@liverpool.ac.uk.

**Number of words:** 3,055 words

**Abstract word count:** 365 words

**Number of tables:
Number of figures:**

**Key words:** anaemia; surgical systems; outcomes; healthcare quality; outcomes

**Funding:** The Student Audit and Research in Surgery collaborative is supported with a Strategic Partnership with BJS Society and sponsored by the Royal College of Surgeons of Edinburgh (no grant numbers). SKK was funded by the NIHR Doctoral Research Fellowship (NIHR303288). DB was funded via an NIHR Academic Clinical Fellowship and Wellbeing of Women’s ELS (ELS1317).

**Data sharing:** Data sharing requests will be considered by the writing group upon written request to the corresponding authors.

**Conflict of interest:** There are no conflicts of interest to declare.

Table of Contents

[METHODOLOGY	3](#_Toc237330384)

[**Figure S1.** Examples of days alive and out of hospital at 30-days (DAOH30) calculation	3](#_Toc237330385)

[Baseline characteristics	4](#_Toc237330386)

[**Table S1.** Baseline patient- and operative-level characteristics of women undergoing elective abdominal surgery by pre-operative anaemia status	4](#_Toc237330387)

[Unplanned readmission within 30-days	5](#_Toc237330388)

[**Table S2.** Baseline patient- and operative-level characteristics of women undergoing elective abdominal surgery by 30-day unplanned readmission	5](#_Toc237330389)

[**Table S3.** Multilevel logistic regression of patient and operative characteristics on 30-day unplanned readmission in women undergoing elective abdominal surgery. Multilevel model	6](#_Toc237330390)

[**Figure S2.** Directed acyclic graph illustrating the mediating role of perioperative iron treatment in the association between surgical pathway and 30-day unplanned readmission, adjusted for confounders including menopausal status, multimorbidity, and operative characteristics.	7](#_Toc237330391)

[Interaction subgroup analysis	8](#_Toc237330392)

[**Table S4.** Multivariable interaction model. Levels for co-variates held constant in bootstrap simulations.	8](#_Toc237330393)

[**Table S5.**  Multivariable interaction model. Simulations were undertaken with the following variables varied: preoperative anaemia status (Not anaemic/Anaemic), menopausal status (Pre-menopausal /Post-menopausal) and surgical pathway (Gynaecology/Non-gynaecology (with sensitivity analysis for GI and Liver surgery only)). Other model variables were held at their mode (Table S4). Bootstrap replications of the interaction model (n=2000) were used to calculate the conditional probability of the unplanned readmission. Relative and absolute risk differences calculated, together with 95% confidence intervals and a two-side p-value were calculated. Number-needed-to-treat (NNT) was defined as 1/absolute risk difference.	9](#_Toc237330394)

[Days alive and out-of-hospital within 30-days	10](#_Toc237330395)

[**Table S6.** Associations between patient- and operative-characteristics on days alive and out-of-hospital at 30-days from surgery. Results presented are based on quantile regression models. Estimates are presented as β coefficients with 95% confidence intervals and p-values. Quantile regression models were fitted at the 10th, 50th (median), and 90th percentiles to evaluate heterogeneity of effects across the DAOH30 distribution.	10](#_Toc237330396)

[Table S7: Sensitivity analysis by operative specialty	13](#_Toc237330397)

[Appendix	14](#_Toc237330398)

[**Appendix S1.** Summary of the ethics approval obtained of the main CASCADE study.	14](#_Toc237330399)

[**Appendix S2.** Detailed description of the CASCADE cohort study	16](#_Toc237330400)

[**Appendix S3.** STROBE Statement; checklist of items that should be included in reports of observational studies	17](#_Toc237330401)

[**Appendix S4.** References	19](#_Toc237330402)

[**Appendix S5.** Authorship group	20](#_Toc237330403)

[Writing group	20](#_Toc237330404)

[Data analysis	20](#_Toc237330405)

[Steering Committee	20](#_Toc237330406)

[External advisory group	20](#_Toc237330407)

[National leads	20](#_Toc237330408)

[Regional leads	21](#_Toc237330409)

[Collaborators	21](#_Toc237330410)

# METHODOLOGY

### **Figure S1.** Examples of days alive and out of hospital at 30-days (DAOH30) calculation

# Baseline characteristics

### **Table S1.** Baseline patient- and operative-level characteristics of women undergoing elective abdominal surgery by pre-operative anaemia status

|  |  | **No preoperative anaemia** | **Pre-operative anaemia** | **Total** | **p-value** |
| --- | --- | --- | --- | --- | --- |
| Total N (%) |  | 6802 (69.9) | 2933 (30.1) | 9735 |  |
| Age, years | Median (IQR) | 57.0 (46.0 to 68.0) | 62.0 (48.0 to 74.0) | 59.0 (47.0 to 70.0) | <0.0001 |
| Menopause status | Pre-menopausal | 2476 (36.4) | 917 (31.3) | 3393 (34.9) | <0.0001 |
|  | Post-menopausal | 4326 (63.6) | 2016 (68.7) | 6342 (65.1) |  |
|  | (Missing) | 0 (0.0) | 0 (0.0) | 0 (0.0) |  |
| Operative specialty | Gynaecology | 2328 (34.2) | 733 (25.0) | 3061 (31.4) | <0.0001 |
|  | Non-gynaecology | 4474 (65.8) | 2200 (75.0) | 6674 (68.6) |  |
|  | (Missing) | 0 (0.0) | 0 (0.0) | 0 (0.0) |  |
| ASA grade | I-II | 5254 (77.2) | 1778 (60.6) | 7032 (72.2) | <0.0001 |
|  | III-V | 1428 (21.0) | 1083 (36.9) | 2511 (25.8) |  |
|  | (Missing) | 120 (1.8) | 72 (2.5) | 192 (2.0) |  |
| Smoking status | Never smoked | 3778 (55.5) | 1827 (62.3) | 5605 (57.6) | <0.0001 |
|  | Current | 1148 (16.9) | 317 (10.8) | 1465 (15.0) |  |
|  | Ex-smoker (< 6 weeks) | 59 (0.9) | 36 (1.2) | 95 (1.0) |  |
|  | Ex-smoker (6 weeks - 1 year) | 105 (1.5) | 46 (1.6) | 151 (1.6) |  |
|  | Ex-smoker (>1 year) | 858 (12.6) | 362 (12.3) | 1220 (12.5) |  |
|  | (Missing) | 854 (12.6) | 345 (11.8) | 1199 (12.3) |  |
| Clinical frailty | 1-3 | 5522 (89.4) | 2103 (79.6) | 7625 (86.4) | <0.0001 |
|  | 4-6 | 631 (10.2) | 515 (19.5) | 1146 (13.0) |  |
|  | 7-9 | 27 (0.4) | 25 (0.9) | 52 (0.6) |  |
| Number of comorbidities | None | 2415 (35.5) | 628 (21.4) | 3043 (31.3) | <0.0001 |
|  | One | 2235 (32.9) | 906 (30.9) | 3141 (32.3) |  |
|  | Two | 1342 (19.7) | 711 (24.2) | 2053 (21.1) |  |
|  | Three | 535 (7.9) | 421 (14.4) | 956 (9.8) |  |
|  | Four or more | 272 (4.0) | 263 (9.0) | 535 (5.5) |  |
|  | (Missing) | 3 (0.0) | 4 (0.1) | 7 (0.1) |  |
| Indication | Benign | 4417 (64.9) | 1320 (45.0) | 5737 (58.9) | <0.0001 |
|  | Malignant | 2376 (34.9) | 1612 (55.0) | 3988 (41.0) |  |
|  | (Missing) | 9 (0.1) | 1 (0.0) | 10 (0.1) |  |
| Operative approach | Minimally-invasive | 4377 (64.3) | 1518 (51.8) | 5895 (60.6) | <0.0001 |
|  | Open | 2424 (35.6) | 1415 (48.2) | 3839 (39.4) |  |
|  | (Missing) | 1 (0.0) | 0 (0.0) | 1 (0.0) |  |
| Postoperative nadir Hb | Not anaemic | 1772 (26.1) | 81 (2.8) | 1853 (19.0) | <0.0001 |
|  | Anaemic | 2996 (44.0) | 2406 (82.0) | 5402 (55.5) |  |
|  | (Missing) | 2034 (29.9) | 446 (15.2) | 2480 (25.5) |  |
| Postoperative anaemia on discharge | No | 1727 (25.4) | 148 (5.0) | 1875 (19.3) | <0.0001 |
|  | Yes | 2325 (34.2) | 1980 (67.5) | 4305 (44.2) |  |
|  | (Missing) | 2750 (40.4) | 805 (27.4) | 3555 (36.5) |  |
| Preoperative anaemia iron treatment | No | 6497 (95.5) | 2426 (82.7) | 8923 (91.7) | <0.0001 |
|  | Yes | 305 (4.5) | 507 (17.3) | 812 (8.3) |  |
|  | (Missing) | 0 (0.0) | 0 (0.0) | 0 (0.0) |  |
| Postoperative anaemia iron treatment | No | 6443 (94.7) | 2475 (84.4) | 8918 (91.6) | <0.0001 |
|  | Yes | 359 (5.3) | 458 (15.6) | 817 (8.4) |  |
|  | (Missing) | 0 (0.0) | 0 (0.0) | 0 (0.0) |  |
| Post-operative transfusion | No | 6615 (97.3) | 2571 (87.7) | 9186 (94.4) | <0.0001 |
|  | Yes | 187 (2.7) | 362 (12.3) | 549 (5.6) |  |
|  | (Missing) | 0 (0.0) | 0 (0.0 | 0 (0.0) |  |

# Unplanned readmission within 30-days

### **Table S2.** Baseline patient- and operative-level characteristics of women undergoing elective abdominal surgery by 30-day unplanned readmission

|  |  | No | Yes | p-value |
| --- | --- | --- | --- | --- |
| Total N (%) |  | 9390 (92.8) | 724 (7.2) |  |
| Preoperative anaemia | No | 6368 (93.6) | 434 (6.4) | <0.0001 |
|  | Yes | 2664 (90.8) | 269 (9.2) |  |
|  | (Missing) | 358 (94.5) | 21 (5.5) |  |
| Menopause status | Pre-menopausal | 3282 (91.4) | 308 (8.6) | <0.0001 |
|  | Post-menopausal | 6108 (93.6) | 416 (6.4) |  |
|  | (Missing) | 0 (NaN) | 0 (NaN) |  |
| Surgical pathway | Gynaecology | 3010 (93.7) | 204 (6.3) | 0.03 |
|  | Non-gynaecology | 6380 (92.5) | 520 (7.5) |  |
|  | (Missing) | 0 (NaN) | 0 (NaN) |  |
| ASA physical status | I-II | 6795 (92.7) | 534 (7.3) | 0.6525 |
|  | III-V | 2394 (93.0) | 180 (7.0) |  |
|  | (Missing) | 201 (95.3) | 10 (4.7) |  |
| Smoking status | Never smoked | 5352 (92.4) | 442 (7.6) | 0.8877 |
|  | Current | 1407 (92.6) | 113 (7.4) |  |
|  | Ex-smoker (< 6 weeks) | 90 (92.8) | 7 (7.2) |  |
|  | Ex-smoker (6 weeks - 1 year) | 141 (91.6) | 13 (8.4) |  |
|  | Ex-smoker (>1 year) | 1171 (93.2) | 86 (6.8) |  |
|  | (Missing) | 1229 (95.1) | 63 (4.9) |  |
| Clinical frailty | 1-3 | 7347 (93.0) | 553 (7.0) | 0.0430 |
|  | 4-6 | 1084 (92.1) | 93 (7.9) |  |
|  | 7-9 | 45 (84.9) | 8 (15.1) |  |
| Number of comorbidities | None | 2970 (92.5) | 242 (7.5) | 0.6172 |
|  | One | 3044 (93.3) | 219 (6.7) |  |
|  | Two | 1965 (93.2) | 144 (6.8) |  |
|  | Three | 904 (92.3) | 75 (7.7) |  |
|  | Four or more | 501 (92.3) | 42 (7.7) |  |
|  | (Missing) | 6 (75.0) | 2 (25.0) |  |
| Indication | Benign | 5588 (92.5) | 452 (7.5) | 0.1412 |
|  | Malignant | 3792 (93.3) | 272 (6.7) |  |
|  | (Missing) | 10 (100.0) | 0 (0.0) |  |
| Operative approach | Minimally-invasive | 5747 (93.2) | 418 (6.8) | 0.0707 |
|  | Open | 3642 (92.2) | 306 (7.8) |  |
|  | (Missing) | 1 (100.0) | 0 (0.0) |  |
| Postoperative nadir Hb | Not anaemic | 1815 (95.2) | 92 (4.8) | <0.0001 |
|  | Anaemic | 5025 (91.4) | 474 (8.6) |  |
|  | (Missing) | 2550 (94.2) | 158 (5.8) |  |
| Postoperative anaemia on discharge | Not anaemic | 1822 (95.0) | 95 (5.0) | <0.0001 |
|  | Anaemic | 3990 (91.1) | 389 (8.9) |  |
|  | (Missing) | 3578 (93.7) | 240 (6.3) |  |
| Preoperative anaemia iron treatment | No | 8627 (92.8) | 668 (7.2) | 0.7636 |
|  | Yes | 763 (93.2) | 56 (6.8) |  |
|  | (Missing) | 0 (NaN) | 0 (NaN) |  |
| Postoperative anaemia iron treatment | No | 8634 (93.0) | 648 (7.0) | 0.0252 |
|  | Yes | 756 (90.9) | 76 (9.1) |  |
|  | (Missing) | 0 (NaN) | 0 (NaN) |  |
| Post-operative transfusion | No | 8894 (93.1) | 663 (6.9) | 0.0005 |
|  | Yes | 496 (89.0) | 61 (11.0) |  |
|  | (Missing) | 0 (NaN) | 0 (NaN) |  |

### **Table S3.** Multilevel logistic regression of patient and operative characteristics on 30-day unplanned readmission in women undergoing elective abdominal surgery. Multilevel model

includes hospital and country identifier as a random effect.

|  |  | **No** | **Yes** | **OR (univariable)** | **OR (multivariable full)** |
| --- | --- | --- | --- | --- | --- |
| Operative specialty | Gynaecology | 3010 (93.7) | 204 (6.3) | - | - |
|  | Non-gynaecology | 6380 (92.5) | 520 (7.5) | 1.20 (1.02-1.42, p=0.031) | 0.78 (0.62-0.97, p=0.028) |
| Pre-operative anaemia | Not anaemic | 6368 (93.6) | 434 (6.4) | - | - |
|  | Anaemic | 2664 (90.8) | 269 (9.2) | 1.48 (1.26-1.74, p<0.001) | 1.28 (1.06-1.56, p=0.011) |
| Menopause status | Pre-menopausal | 3282 (91.4) | 308 (8.6) | - | - |
|  | Post-menopausal | 6108 (93.6) | 416 (6.4) | 0.73 (0.62-0.85, p<0.001) | 0.77 (0.62-0.95, p=0.016) |
|  |  |  |  |  |  |
|  |  |  |  |  |  |
| ASA physical status | I-II | 6795 (92.7) | 534 (7.3) | - | - |
|  | III-V | 2394 (93.0) | 180 (7.0) | 0.96 (0.80-1.14, p=0.621) | 0.90 (0.71-1.15, p=0.407) |
| Smoking status | Never smoked | 5352 (92.4) | 442 (7.6) | - | - |
|  | Current | 1407 (92.6) | 113 (7.4) | 0.97 (0.78-1.21, p=0.799) | 1.03 (0.81-1.32, p=0.795) |
|  | Ex-smoker (< 6 weeks) | 90 (92.8) | 7 (7.2) | 0.94 (0.43-2.04, p=0.879) | 0.90 (0.40-2.04, p=0.796) |
|  | Ex-smoker (6 weeks - 1 year) | 141 (91.6) | 13 (8.4) | 1.12 (0.63-1.99, p=0.708) | 0.96 (0.50-1.84, p=0.902) |
|  | Ex-smoker (>1 year) | 1171 (93.2) | 86 (6.8) | 0.89 (0.70-1.13, p=0.337) | 1.04 (0.79-1.37, p=0.760) |
| Clinical frailty | 1-3 | 7347 (93.0) | 553 (7.0) | - | - |
|  | 4-6 | 1084 (92.1) | 93 (7.9) | 1.14 (0.91-1.43, p=0.262) | 1.07 (0.81-1.42, p=0.628) |
|  | 7-9 | 45 (84.9) | 8 (15.1) | 2.36 (1.11-5.04, p=0.026) | 2.70 (1.18-6.15, p=0.018) |
| Number of comorbidities | None | 2970 (92.5) | 242 (7.5) | - | - |
|  | One | 3044 (93.3) | 219 (6.7) | 0.88 (0.73-1.07, p=0.198) | 1.09 (0.86-1.39, p=0.480) |
|  | Two | 1965 (93.2) | 144 (6.8) | 0.90 (0.73-1.11, p=0.331) | 1.23 (0.91-1.66, p=0.178) |
|  | Three | 904 (92.3) | 75 (7.7) | 1.02 (0.78-1.33, p=0.896) | 1.35 (0.92-1.97, p=0.121) |
|  | Four or more | 501 (92.3) | 42 (7.7) | 1.03 (0.73-1.45, p=0.870) | 1.47 (0.90-2.38, p=0.120) |
| Indication | Benign | 5588 (92.5) | 452 (7.5) | - | - |
|  | Malignant | 3792 (93.3) | 272 (6.7) | 0.89 (0.76-1.04, p=0.131) | 0.96 (0.77-1.20, p=0.732) |
| Operative approach | Minimally-invasive | 5747 (93.2) | 418 (6.8) | - | - |
|  | Open | 3642 (92.2) | 306 (7.8) | 1.16 (0.99-1.35, p=0.065) | 1.34 (1.11-1.63, p=0.002) |
| Wound contamination | Clean | 4616 (94.2) | 282 (5.8) | - | - |
|  | Clean-Contaminated | 4468 (91.5) | 415 (8.5) | 1.52 (1.30-1.78, p<0.001) | 1.35 (1.12-1.63, p=0.002) |
|  | Contaminated/Dirty | 285 (92.2) | 24 (7.8) | 1.38 (0.89-2.13, p=0.147) | 1.19 (0.72-1.96, p=0.492) |

### **Figure S2.** Directed acyclic graph illustrating the mediating role of perioperative iron treatment in the association between surgical pathway and 30-day unplanned readmission, adjusted for confounders including menopausal status, multimorbidity, and operative characteristics.

## Interaction subgroup analysis

### **Table S4.** Multivariable interaction model. Levels for co-variates held constant in bootstrap simulations.

| Variable | Value |
| --- | --- |
| Multimorbidity | No |
| ASA grade | I-II |
| Frailty score | 1-3 |
| Operative indication | Benign |
| Operative approach | Minimally invasive |

### **Table S5.**  Multivariable interaction model. Simulations were undertaken with the following variables varied: preoperative anaemia status (Not anaemic/Anaemic), menopausal status (Pre-menopausal /Post-menopausal) and surgical pathway (Gynaecology/Non-gynaecology (with sensitivity analysis for GI and Liver surgery only)). Other model variables were held at their mode (Table S4). Bootstrap replications of the interaction model (n=2000) were used to calculate the conditional probability of the unplanned readmission. Relative and absolute risk differences calculated, together with 95% confidence intervals and a two-side p-value were calculated. Number-needed-to-treat (NNT) was defined as 1/absolute risk difference.

| Menopausal status | Operative specialty | No pre-operative anaemia | Pre-operative anaemia | Unplanned readmission **absolute risk difference** with no preoperative anaemia (%) | Unplanned readmission **number needed to treat** with no preoperative anaemia |
| --- | --- | --- | --- | --- | --- |
| Post-menopausal | Gynaecology | 3.40 (2.48 to 4.43) | 6.41 (4.63 to 8.04) | -2.94 (-4.36 to -1.68) | 34.0 (59.5 to 22.9) |
| Post-menopausal | Non-gynaecology | 3.91 (2.84 to 4.96) | 7.35 (5.96 to 8.86) | -3.43 (-4.62 to -2.31, p<0.001) | 29.2 (43.3 to 21.6) |
| Post-menopausal | GI and liver only | 3.97 (2.93 to 5.08) | 7.38 (5.91 to 9.08) | -3.37 (-4.93 to -2.08, p<0.001) | 29.7 (48.1 to 20.3) |
| Pre-menopausal | Gynaecology | 5.62 (4.22 to 7.05) | 10.38 (7.91 to 13.03) | -4.62 (-6.60 to -2.82, p<0.001) | 21.6 (35.5 to 15.2) |
| Pre-menopausal | Non-gynaecology | 6.44 (4.97 to 8.27) | 11.82 (9.91 to 13.80) | -5.32 (-7.36 to -3.59, p<0.001) | 18.8 (27.9 to 13.6) |
| Post-menopausal | GI and liver only | 3.97 (2.93 to 5.08) | 7.38 (5.91 to 9.08) | -3.37 (-4.93 to -2.08, p<0.001) | 29.7 (48.1 to 20.3) |

# Days alive and out-of-hospital within 30-days

### **Table S6.** Associations between patient- and operative-characteristics on days alive and out-of-hospital at 30-days from surgery. Results presented are based on quantile regression models. Estimates are presented as β coefficients with 95% confidence intervals and p-values. Quantile regression models were fitted at the 10th, 50th (median), and 90th percentiles to evaluate heterogeneity of effects across the DAOH30 distribution.

| **Characteristic** | OR 5% | | | QR 10% | | | QR 25% | | | QR 50% | | | OR 75% | | | QR 90% | | |
| --- | --- | --- | --- | --- | --- | --- | --- | --- | --- | --- | --- | --- | --- | --- | --- | --- | --- | --- |
|  | Beta | 95% CI | p-value | Beta | 95% CI | p-value | Beta | 95% CI | p-value | Beta | 95% CI | p-value | Beta | 95% CI | p-value | Beta | 95% CI | p-value |
| Operative Specialty |  |  |  |  |  |  |  |  |  |  |  |  |  |  |  |  |  |  |
| Non-gynaecology | — | — |  | — | — |  | — | — |  | — | — |  | — | — |  | — | — |  |
| Gynaecology | -4.3 | -5.1, -3.5 | **<0.001** | -3.7 | -4.3, -3.0 | **<0.001** | -2.2 | -2.4, -1.9 | **<0.001** | -1.1 | -1.3, -0.99 | **<0.001** | -1.0 | -1.2, -0.77 | **<0.001** | -1.0 | -1.9, -0.11 | **0.028** |
| Preoperative anaemia |  |  |  |  |  |  |  |  |  |  |  |  |  |  |  |  |  |  |
| Not anaemic | — | — |  | — | — |  | — | — |  | — | — |  | — | — |  | — | — |  |
| Anaemic | -2.9 | -3.8, -1.9 | **<0.001** | -2.3 | -3.1, -1.5 | **<0.001** | -1.3 | -1.7, -1.0 | **<0.001** | -0.88 | -1.1, -0.68 | **<0.001** | -1.0 | -1.2, -0.78 | **<0.001** | 0.00 | -0.85, 0.85 | >0.9 |
| Menopause status |  |  |  |  |  |  |  |  |  |  |  |  |  |  |  |  |  |  |
| Pre-menopausal | — | — |  | — | — |  | — | — |  | — | — |  | — | — |  | — | — |  |
| Post-menopausal | -0.57 | -1.5, 0.35 | 0.2 | 0.00 | -0.71, 0.71 | >0.9 | 0.00 | -0.25, 0.25 | >0.9 | 0.00 | -0.14, 0.14 | >0.9 | 0.00 | -0.21, 0.21 | >0.9 | 0.00 | -0.40, 0.40 | >0.9 |
| ASA physical status |  |  |  |  |  |  |  |  |  |  |  |  |  |  |  |  |  |  |
| I-II | — | — |  | — | — |  | — | — |  | — | — |  | — | — |  | — | — |  |
| III-V | -5.0 | -5.9, -4.1 | **<0.001** | -3.0 | -4.7, -1.3 | **<0.001** | -1.2 | -1.5, -0.81 | **<0.001** | -0.63 | -0.89, -0.36 | **<0.001** | -1.0 | -1.3, -0.72 | **<0.001** | 0.00 | -0.38, 0.38 | >0.9 |
| Smoking status |  |  |  |  |  |  |  |  |  |  |  |  |  |  |  |  |  |  |
| Never smoked | — | — |  | — | — |  | — | — |  | — | — |  | — | — |  | — | — |  |
| Current | -1.0 | -2.0, 0.00 | **0.049** | -0.67 | -1.4, 0.08 | 0.081 | 0.00 | -0.29, 0.29 | >0.9 | 0.00 | -0.16, 0.16 | >0.9 | 0.00 | -0.22, 0.22 | >0.9 | 0.00 | -0.43, 0.43 | >0.9 |
| Ex-smoker (< 6 weeks) | 0.43 | -1.5, 2.4 | 0.7 | 0.33 | -2.7, 3.4 | 0.8 | 0.83 | 0.10, 1.6 | **0.025** | -0.25 | -0.93, 0.43 | 0.5 | 0.00 | -1.0, 1.0 | >0.9 | 0.00 | -1.1, 1.1 | >0.9 |
| Ex-smoker (6 weeks - 1 year) | -1.7 | -3.5, 0.09 | 0.063 | -1.0 | -2.5, 0.53 | 0.2 | -0.50 | -2.4, 1.4 | 0.6 | -0.13 | -0.71, 0.46 | 0.7 | 0.00 | -0.67, 0.67 | >0.9 | 0.00 | -1.2, 1.2 | >0.9 |
| Ex-smoker (>1 year) | -0.57 | -1.8, 0.70 | 0.4 | 0.00 | -0.98, 0.98 | >0.9 | -0.17 | -0.47, 0.13 | 0.3 | -0.13 | -0.35, 0.10 | 0.3 | 0.00 | -0.28, 0.28 | >0.9 | 0.00 | -0.42, 0.42 | >0.9 |
| Clinical frailty |  |  |  |  |  |  |  |  |  |  |  |  |  |  |  |  |  |  |
| 1-3 | — | — |  | — | — |  | — | — |  | — | — |  | — | — |  | — | — |  |
| 4-6 | -2.3 | -3.6, -0.94 | **<0.001** | -3.3 | -5.4, -1.3 | **0.001** | -1.0 | -1.5, -0.55 | **<0.001** | -0.50 | -0.79, -0.21 | **<0.001** | 0.00 | -0.31, 0.31 | >0.9 | 0.00 | -0.46, 0.46 | >0.9 |
| 7-9 | -8.4 | -13, -4.3 | **<0.001** | -11 | -25, 2.0 | 0.094 | -3.3 | -7.1, 0.39 | 0.079 | -1.4 | -2.2, -0.59 | **<0.001** | 0.00 | -1.4, 1.4 | >0.9 | 0.00 | -1.7, 1.7 | >0.9 |
| Number of comorbidities |  |  |  |  |  |  |  |  |  |  |  |  |  |  |  |  |  |  |
| None | — | — |  | — | — |  | — | — |  | — | — |  | — | — |  | — | — |  |
| One | -0.29 | -1.3, 0.70 | 0.6 | -0.67 | -1.3, -0.06 | **0.031** | -0.17 | -0.43, 0.09 | 0.2 | -0.13 | -0.37, 0.12 | 0.3 | 0.00 | -0.23, 0.23 | >0.9 | 0.00 | -0.49, 0.49 | >0.9 |
| Two | -1.7 | -3.4, -0.04 | **0.044** | -1.0 | -2.2, 0.23 | 0.11 | -0.50 | -0.92, -0.08 | **0.020** | -0.25 | -0.54, 0.04 | 0.087 | 0.00 | -0.29, 0.29 | >0.9 | 0.00 | -0.56, 0.56 | >0.9 |
| Three | -2.7 | -5.3, -0.09 | **0.043** | -1.3 | -3.6, 0.91 | 0.2 | -0.83 | -1.4, -0.22 | **0.008** | -0.63 | -1.1, -0.17 | **0.007** | 0.00 | -0.39, 0.39 | >0.9 | 0.00 | -0.66, 0.66 | >0.9 |
| Four or more | -0.29 | -2.3, 1.7 | 0.8 | -2.3 | -6.0, 1.3 | 0.2 | -0.83 | -1.7, 0.05 | 0.064 | -0.88 | -1.3, -0.44 | **<0.001** | 0.00 | -0.62, 0.62 | >0.9 | 0.00 | -0.76, 0.76 | >0.9 |
| Indication |  |  |  |  |  |  |  |  |  |  |  |  |  |  |  |  |  |  |
| Benign | — | — |  | — | — |  | — | — |  | — | — |  | — | — |  | — | — |  |
| Malignant | -5.4 | -6.6, -4.3 | **<0.001** | -3.3 | -4.5, -2.2 | **<0.001** | -2.5 | -2.8, -2.2 | **<0.001** | -2.1 | -2.3, -1.9 | **<0.001** | -2.0 | -2.2, -1.8 | **<0.001** | -1.0 | -1.9, -0.05 | **0.039** |
| Operative approach |  |  |  |  |  |  |  |  |  |  |  |  |  |  |  |  |  |  |
| Minimally-invasive | — | — |  | — | — |  | — | — |  | — | — |  | — | — |  | — | — |  |
| Open | -6.0 | -6.9, -5.1 | **<0.001** | -4.7 | -5.5, -3.8 | **<0.001** | -3.3 | -3.6, -3.1 | **<0.001** | -2.6 | -2.8, -2.4 | **<0.001** | -2.0 | -2.2, -1.8 | **<0.001** | -2.0 | -2.9, -1.1 | **<0.001** |
| Wound contamination |  |  |  |  |  |  |  |  |  |  |  |  |  |  |  |  |  |  |
| Clean | — | — |  | — | — |  | — | — |  | — | — |  | — | — |  | — | — |  |
| Clean-Contaminated | -2.9 | -3.7, -2.0 | **<0.001** | -2.0 | -2.7, -1.3 | **<0.001** | -1.2 | -1.4, -0.94 | **<0.001** | -0.63 | -0.80, -0.45 | **<0.001** | 0.00 | -0.26, 0.26 | >0.9 | 0.00 | -0.31, 0.31 | >0.9 |
| Contaminated/Dirty | -9.0 | -13, -4.6 | **<0.001** | -9.3 | -12, -6.3 | **<0.001** | -4.3 | -7.2, -1.5 | **0.003** | -2.1 | -3.0, -1.2 | **<0.001** | -1.0 | -1.6, -0.42 | **<0.001** | -1.0 | -2.5, 0.51 | 0.2 |

# Table S7: Sensitivity analysis by operative specialty

| Operative specialty | N | Estimated blood loss, ml | Preoperative anaemia | Preoperative iron therapy | Postoperative iron therapy | 30-day readmission |
| --- | --- | --- | --- | --- | --- | --- |
| Gynaecology | 3214 | 200 (150 to 300) | 733/3061 (23.9%) | 268/3214 (8.3%) | 378/3214 (11.8%) | 204/3213 (6.3%) |
| GI and liver | 6208 | 200 (200 to 200) | 1950/5995 (32.5%) | 495/6208 (8%) | 396/6208 (6.4%) | 464/6208 (7.5%) |
| Urology | 373 | 200 (100 to 300) | 95/366 (26%) | 28/373 (7.5%) | 29/373 (7.8%) | 26/373 (7%) |
| Other/multivisceral | 180 | 300 (200 to 675) | 75/178 (42.1%) | 15/180 (8.3%) | 11/180 (6.1%) | 11/180 (6.1%) |
| Vascular/transplant | 139 | 300 (200 to 812) | 80/135 (59.3%) | 13/139 (9.4%) | 18/139 (12.9%) | 19/139 (13.7%) |
| Values are n/N (%) unless otherwise stated. Estimated blood loss is median (IQR). Vascular and transplant surgery were combined because of small numbers. | | | | | | |

# Appendix

### **Appendix S1.** Summary of the ethics approval obtained of the main CASCADE study.

| **Country** | **National Lead** | **Hospitals, n** | **Type of approval** | **Details (if applicable)** |
| --- | --- | --- | --- | --- |
| Albania | Irida Dajti | 1 | Local* | REC review at University Hospital Koco Gliozheni |
| Austria | Michael de Cillia | 4 | Regional^†^ | Ethikkommission Salzburg, Nr.: 1001/2022 |
| Belgium | Gabrielle van Ramshorst | 3 | National | REC committee approval from Ghent University Hospital |
| Bosnia and Herzegovina | Samir Delibegovic | 3 | Regional^†^ | REC review; 06-04-9-58055 |
| Bulgaria | Hannah Mughal | 7 | Local* | Each hospital submitted for local REC review based in their hospitals. |
| Croatia | Jakov Mihanovic | 3 | National | Ethical Committee for Zadar General Hospital; 02-1237/22-3/22. |
| Cyprus | Nikolaos Gouvas | 1 | National | Cyprus National Bioethics Committee; ΕΕΒΚ ΕΠ 2022.01.05 |
| Czech Republic | Petr Kocian | 3 | National | Motol University Hospital; 108/23. |
| France | - | 3 | Local* | Each hospital submitted for local REC review based in their hospitals. |
| Germany | Niall Brindl, Daniela Merz, Maximilian Joos | 13 | Local* | Each hospital submitted for local REC review based in their hospitals. |
| Greece | Argyrios Ioannidis | 25 | Local | Local approval was obtained at each hospital by the hospital leads. |
| Hungary | Gift Nnaji | 1 | Local | REC review by Debrecen University Clinical Center |
| Ireland | - | 7 | Local | Each hospital submitted for local REC review based in their hospitals. |
| Italy | Alice Gori, Francesco Pata, Gaetano Gallo | 84 | Local | Each hospital submitted for local REC review based in their hospitals (e.g., AVEC Bologna, Prot. n. 980/2022, 169/2022/Oss/AOUBo) |
| Latvia | Simone Oliver Senica | 1 | Local | REC review by the local hospital; 110122-10E |
| Lithuania | Albertas Dauksa | 4 | Local* | Each hospital submitted for local REC review based in their hospitals. |
| Malta | Josephine Psaila | 1 | Local | REC review Mater Dei Hospital |
| Netherlands | Fenja de Ruitjer | 2 | Local* | Each hospital submitted for local REC review based in their hospitals. |
| Poland | Piotr Major | 4 | Local* | Each hospital submitted for local REC review based in their hospitals. |
| Portugal | Mafalda Sampaio Alves, Irène Santos, Joana Simões | 27 | Local* | Each hospital submitted for local REC review based in their hospitals. |
| Republic of North Macedonia | - | 1 | Local* | Each hospital submitted for local REC review based in their hospitals. |
| Romania | Eduard-Alexandru Bonci | 12 | Local* | Each hospital submitted for local REC review based in their hospitals. |
| Russia | Anastasia Novikova | 10 | Local* | Each hospital submitted for local REC review based in their hospitals. |
| Serbia | Bojan Kovačević, Vladimir Milosavljevic, Boris Tadic | 6 | Local* | Each hospital submitted for local REC review based in their hospitals. |
| Slovenia | Jurij Kosir | 2 | Regional^†^ | Approval was obtained regionally for two hospitals; 0120-119/2022/4 |
| Spain | Ruth Blanco-Colino | 36 | Local* | REC review by individual hospitals involved. The lead hospital Val’d Hebron REC review (PR(AG)630/2021) facilitated all other hospitals to obtain REC approvals. |
| Switzerland | Andrea Papadia, Maria Luisa Gasparri | 9 | Regional^†^ | Canton approval; 2022-00033 |
| Turkey | Melik Kagan Aktas | 46 | National | Acibadem University Ethics Committee review; 2022-02/36 |
| United Kingdom | Sivesh Kamarajah | 126 | National | REC review at South East Scotland NR161/AB6; hospital leads registered the study locally as an audit or service evaluation. |

**In countries where local approvals for each hospital were obtained, the ethics committee were provided the protocol and translated protocols, patient consent form, and case report form to deliberate on the study, prior to making decisions. Only hospitals that received formal positive opinion were allowed to register and contribute data to the study. ^†^For regional approvals, a primary hospital jointly submitted ethics applications with other hospitals to regional REC.*

### **Appendix S2.** Detailed description of the CASCADE cohort study

The CASCADE cohort study included consecutive adult patients (aged ≥ 18 years) undergoing major abdominal surgeries across five surgical disciplines (abdominal and/or pelvic visceral resection; formation or reversal of stoma; open vascular surgery, anterior abdominal wall hernia repair; or transplant surgery) and through any surgical approach. Eligible patients were identified by local collaborators at each participating hospital during five data collection periods from 24 January to 3 April 2022. Hospitals performing elective and/or emergency major abdominal surgery in the UK, Republic of Ireland and Europe were eligible to enrol in the study. Recruitment of hospitals was primarily through the STARSurg(1) and EuroSurg collaborative group network, alongside open invitation through national scientific societies, individual contacts, and social media. Researchers (doctors, medical students and clinical research staff) in eligible hospitals were asked to form teams to prospectively enrol consecutive patients in up to seven, non-overlapping, pre-defined 14-day patient-inclusion periods between 24th January 2022 and 1st May 2022. Routine, anonymised data, with no change to clinical care pathways, was collected.

Adult patients (≥18 years) who underwent major abdominal surgery (procedures as defined by StEP-COMPAC definitions(2) were eligible. Trauma, planned day-case procedures, and procedures without an abdominal incision were not eligible for inclusion. Patients were identified at the time of operation and were followed up until their death or 30 days postoperatively, whichever came first. All data was stored in a secure database on the Research Electronic Data Capture (REDCap) web application(3) hosted by the University of Birmingham (Birmingham, UK) and only data collection periods with >95% data completeness were accepted for pooled analysis.

### **Appendix S3.** STROBE Statement; checklist of items that should be included in reports of observational studies

|  | Item No. | Recommendation | Page  No. |
| --- | --- | --- | --- |
| **Title and abstract** | 1 | (*a*) Indicate the study’s design with a commonly used term in the title or the abstract | 1 |
|  |  | (*b*) Provide in the abstract an informative and balanced summary of what was done and what was found | 2-3 |
| Introduction | | | |
| Background/rationale | 2 | Explain the scientific background and rationale for the investigation being reported | 6 |
| Objectives | 3 | State specific objectives, including any prespecified hypotheses | 6-7 |
| Methods | | | |
| Study design | 4 | Present key elements of study design early in the paper | 8 |
| Setting | 5 | Describe the setting, locations, and relevant dates, including periods of recruitment, exposure, follow-up, and data collection | 8 |
| Participants | 6 | (*a*) *Cohort study*—Give the eligibility criteria, and the sources and methods of selection of participants. Describe methods of follow-up  *Case-control study*—Give the eligibility criteria, and the sources and methods of case ascertainment and control selection. Give the rationale for the choice of cases and controls  *Cross-sectional study*—Give the eligibility criteria, and the sources and methods of selection of participants | 8 |
|  |  | (*b*) *Cohort study*—For matched studies, give matching criteria and number of exposed and unexposed  *Case-control study*—For matched studies, give matching criteria and the number of controls per case |  |
| Variables | 7 | Clearly define all outcomes, exposures, predictors, potential confounders, and effect modifiers. Give diagnostic criteria, if applicable | 9-10 |
| Data sources/ measurement | 8* | For each variable of interest, give sources of data and details of methods of assessment (measurement). Describe comparability of assessment methods if there is more than one group | *8* |
| Bias | 9 | Describe any efforts to address potential sources of bias | 10-11 |
| Study size | 10 | Explain how the study size was arrived at | 10 |

| Quantitative variables | 11 | Explain how quantitative variables were handled in the analyses. If applicable, describe which groupings were chosen and why |  | 10-11 |
| --- | --- | --- | --- | --- |
| Statistical methods | 12 | (*a*) Describe all statistical methods, including those used to control for confounding |  | 10-11 |
|  |  | (*b*) Describe any methods used to examine subgroups and interactions |  | 10-11 |
|  |  | (*c*) Explain how missing data were addressed |  | 10-11 |
|  |  | (*d*) *Cohort study*—If applicable, explain how loss to follow-up was addressed  *Case-control study*—If applicable, explain how matching of cases and controls was addressed  *Cross-sectional study*—If applicable, describe analytical methods taking account of sampling strategy |  | NA |
|  |  | (*e*) Describe any sensitivity analyses |  | 10-11 |
| Results | | | | |
| Participants | 13* | (a) Report numbers of individuals at each stage of study—eg numbers potentially eligible, examined for eligibility, confirmed eligible, included in the study, completing follow-up, and analysed |  | 12 |
|  |  | (b) Give reasons for non-participation at each stage |  | 12 |
|  |  | (c) Consider use of a flow diagram |  | 12 |
| Descriptive data | 14* | (a) Give characteristics of study participants (eg demographic, clinical, social) and information on exposures and potential confounders |  | 12 |
|  |  | (b) Indicate number of participants with missing data for each variable of interest |  | 12 |
|  |  | (c) *Cohort study*—Summarise follow-up time (eg, average and total amount) |  | 12 |
| Outcome data | 15* | *Cohort study*—Report numbers of outcome events or summary measures over time |  | 12-13 |
|  |  | *Case-control study—*Report numbers in each exposure category, or summary measures of exposure |  |  |
|  |  | *Cross-sectional study—*Report numbers of outcome events or summary measures |  |  |
| Main results | 16 | (*a*) Give unadjusted estimates and, if applicable, confounder-adjusted estimates and their precision (eg, 95% confidence interval). Make clear which confounders were adjusted for and why they were included |  | 12-13 |
|  |  | (*b*) Report category boundaries when continuous variables were categorized |  | 12-13 |
|  |  | (*c*) If relevant, consider translating estimates of relative risk into absolute risk for a meaningful time period |  | 12-13 |

| Other analyses | 17 | Report other analyses done—eg analyses of subgroups and interactions, and sensitivity analyses |  | 13 |
| --- | --- | --- | --- | --- |
| Discussion | | | | |
| Key results | 18 | Summarise key results with reference to study objectives |  | 14 |
| Limitations | 19 | Discuss limitations of the study, taking into account sources of potential bias or imprecision. Discuss both direction and magnitude of any potential bias |  | 16-17 |
| Interpretation | 20 | Give a cautious overall interpretation of results considering objectives, limitations, multiplicity of analyses, results from similar studies, and other relevant evidence |  | 14-16 |
| Generalisability | 21 | Discuss the generalisability (external validity) of the study results |  | 17-18 |
| Other information | |  | | |
| Funding | 22 | Give the source of funding and the role of the funders for the present study and, if applicable, for the original study on which the present article is based |  | 11 |

*Give information separately for cases and controls in case-control studies and, if applicable, for exposed and unexposed groups in cohort and cross-sectional studies.

**Note:** An Explanation and Elaboration article discusses each checklist item and gives methodological background and published examples of transparent reporting. The STROBE checklist is best used in conjunction with this article (freely available on the Web sites of PLoS Medicine at http://www.plosmedicine.org/, Annals of Internal Medicine at http://www.annals.org/, and Epidemiology at http://www.epidem.com/). Information on the STROBE Initiative is available at www.strobe-statement.org.

### **Appendix S4.** References

1.	Collaborative S. Students' participation in collaborative research should be recognised. Int J Surg. 2017;39:234-7.

2.	Beattie WS, Lalu M, Bocock M, Feng S, Wijeysundera DN, Nagele P, et al. Systematic review and consensus definitions for the Standardized Endpoints in Perioperative Medicine (StEP) initiative: cardiovascular outcomes. Br J Anaesth. 2021;126(1):56-66.

3.	Harris PA, Taylor R, Minor BL, Elliott V, Fernandez M, O'Neal L, et al. The REDCap consortium: Building an international community of software platform partners. J Biomed Inform. 2019;95:103208.

4.	Are C, Murthy SS, Sullivan R, Schissel M, Chowdhury S, Alatise O, et al. Global Cancer Surgery: pragmatic solutions to improve cancer surgery outcomes worldwide. Lancet Oncol. 2023;24(12):e472-e518.

5.	Dare AJ, Anderson BO, Sullivan R, Pramesh CS, Andre I, Adewole IF, et al. Surgical Services for Cancer Care. In: Gelband H, Jha P, Sankaranarayanan R, Horton S, editors. Cancer: Disease Control Priorities, Third Edition (Volume 3). Washington (DC)2015.

6.	Global Burden of Disease Healthcare Access Quality Collaborators. Assessing performance of the Healthcare Access and Quality Index, overall and by select age groups, for 204 countries and territories, 1990-2019: a systematic analysis from the Global Burden of Disease Study 2019. Lancet Glob Health. 2022;10(12):e1715-e43.

7.	GBD Universal Health Coverage Collaborators. Measuring universal health coverage based on an index of effective coverage of health services in 204 countries and territories, 1990-2019: a systematic analysis for the Global Burden of Disease Study 2019. Lancet. 2020;396(10258):1250-84.

8.	Holmer H, Lantz A, Kunjumen T, Finlayson S, Hoyler M, Siyam A, et al. Global distribution of surgeons, anaesthesiologists, and obstetricians. Lancet Glob Health. 2015;3 Suppl 2:S9-11.

9.	Bray F, Jemal A, Grey N, Ferlay J, Forman D. Global cancer transitions according to the Human Development Index (2008-2030): a population-based study. Lancet Oncol. 2012;13(8):790-801.

10.	Chiarugi M, Galatioto C, Panicucci S, Scassa F, Zocco G, Seccia M. Oncologic colon cancer resection in emergency: are we doing enough? Surg Oncol. 2007;16 Suppl 1:S73-7.

### **Appendix S5.** Authorship group

#### Writing group

Dania Badran*, Omar Kouli*, Ieva Jakaitytė, Setthasorn Ooi Zhi Yang, Aneel Bhangu, Kariem El-Boghdadly, James Glasbey, Ana Minaya Bravo, Gregory Lip, Shakila Thangaratinam, Dharani Hangapama^†^, Sivesh K Kamarajah^†^

*joint first authors

^†^joint senior authors

#### Data analysis

Omar Kouli, Sivesh Kamarajah (overall guarantor)

#### Steering Committee

Kamarajah SK (Chair), Blanco-Colino R, Brindl N, Brown S, Cambridge WA, Chaudhry D, Gressmann K, Gujjuri RR, Hilder A, Jaffer A, Jakaityte I, Kawka M, Kouli O, Matey E, McLean KA, Mergo A, Mills EC, Murray V, Ooi SZY, Pereira IS, Riad AM, Sgrò A, Shafi SQ, Trout I, Turňa A, Varghese C, Xu W

#### External advisory group

Kamarajah SK (Chair), Biccard BM, Docherty A, Martin J, El-Boghdadly K, Phull M, Mouton R, Bhangu A, Glasbey JC, Harrison EM, McLean KA, Smart N, Moug S, Pinkney T, Richards T

#### National leads

Kamarajah SK (Chair), Dajti I (Albania), de Cillia M (Austria), Van Ramshorst G (Belgium), Delibegovic S (Bosnia and Herzegovina), Mughal H (Bulgaria), Mihanovic J (Croatia), Gouvas N (Cyprus), Kocian P (Czech Republic), Levesen V (Denmark), Kaupilla J (Finland), Brindl N (Germany), Merz D (Germany), Joos M (Germany), Ioannidis A (Greece), Nnaji G (Hungary), Pata F (Italy), Pellino G (Italy), Gori A (Italy), Podda M (Italy), Riboni C (Italy), Fekaj E (Kosovo), Oliver Senica S (Latvia), Dauksa A (Lithuania), Psaila J (Malta), de Ruitjer F (Netherlands), Major P (Poland), Santos I (Portugal), Sampaio Alves M (Portugal), Simões J (Portugal), Bonci EA (Romania), Pașca A (Romania), Novikova A (Russia), Kovačević B (Serbia), Milosavljevic V (Serbia), Tadic B (Serbia), Kosir J (Slovenia), Blanco-Colino R (Spain), Pérez Ajates S (Spain), Ossola Revilla ME (Spain), Papadia A (Switzerland), Riboni C (Switzerland), Gasparri ML (Switzerland), Aktas MK (Turkey), Baki BE (Turkey), Tepe MD (Turkey), Mutlu AU (Turkey),

#### Regional leads

**(Kamarajah SK - Chair)**

**United Kingdom**: Singal A (Aston University Medical School); Osei-Bonsu J (Barts and The London School of Medicine and Dentistry); Lacey H (Brighton and Sussex Medical School); Chan SW (Cardiff University School of Medicine); Allison M (Hull York Medical School); Duah-Asante K (Imperial College London Faculty of Medicine); Chen D (Keele University School of Medicine); Ahmed N (King’s College London GKT School of Medical Education); Ejiz A (Lancaster University Medical School); Takyi C (Newcastle University School of Medical Education); Mujeeb M (Plymouth University Peninsula Schools of Medicine and Dentistry); Ravikumar N (Queen’s University Belfast School of Medicine); Khan M (Royal College of Surgeons in Ireland School of Medicine); Hayes J (St George’s, University of London); Mckenna J (Swansea University Medical School); Wang J (The University of Edinburgh Medical School); Essa N (Trinity College Dublin School of Medicine); Xianghan H (University College Cork School of Medicine); Ko L (University College Dublin School of Medicine); Aldabbagh Y (University College London Medical School); Plascevic J (University of Aberdeen School of Medicine and Dentistry); Zia N (University of Birmingham College of Medical and Dental Sciences); Ismail R (University of Bristol Medical School); Kamel Y (University of Buckingham Medical School); Epanomeritakis I (University of Cambridge School of Clinical Medicine); Tan R (University of Dundee School of Medicine); Chiu N (University of Exeter Medical School); Naeem A (University of Glasgow School of Medicine); Kakwani M (University of Leeds School of Medicine); Mehra R (University of Leicester Medical School); Feeney K (University of Limerick School of Medicine); Yan Naing CL (University of Liverpool School of Medicine); Qureshi A (University of Manchester Medical School); Richens A (University of Nottingham School of Medicine); Li H (University of Oxford Medical Sciences Division); Ahmed R (University of Sheffield Medical School); Wilson  L (University of Southampton School of Medicine); Abraha S (University of Warwick Medical School).

#### Collaborators

**Albania**: Dajti I* (University hospital Koco Gliozheni).

**Austria**: Mikalauskas S*, Kahn J, Kniepeiss D, Kušar M, Belarmino A, Frenk J, Mikalauskiene I, Angelis A, Waha JE, Al-Sharafy S, Sandano M, Schemmer P (Medical University of Graz); Reiterer C*, Horvath K, Taschner A, Riss S, Harpain F, Dawoud C, Hantáková N, Adamowitsch N, Schallmeiner S, Christian T, Xu V, Kuhrn MM, Widmann KM, Capek B, Kama B, Turgut S (Medical University of Vienna); Hauptmann L*, de Cillia M, Grünbart M, Hoi H (Saint John of God Hospital Salzburg); Binder A*, Gürtler T, Riedl P, Mayer D (Universitätsklinikum Tulln).

**Belgium**: Van Belle K*, Wautelet O, Dekkers E (Europe Hospitals); Van Daele E*, Apers T*, Van Ramshorst G, Berrevoet F, Rennie N, Dries P, Panta P, Denys A, Pype W, Bauwens T, Renard N (University Hospital of Ghent); Violon S*, Stijns J, Van De Winkel T, Van Eetvelde E (UZ Brussel).

**Bosnia and Herzegovina**: Salibasic M*, Delibegovic S, Pusina S, Hodžić E, Kruščica M, Žilić S, Bicakcic E, Rovcanin A, Kulovic E, Halilovic E, Vinčević Hodžić A (Clinical Center University of Sarajevo); Kalbić E*, Delibegovic S, Mujić A, Mešan M (General hospital Travnik); Matović E*, Delibegovic S, Kesetovic A (University Clinical Center Tuzla).

**Bulgaria**: Dardanov D*, Arabadzhieva E, Markov B, Спасова К, Biji C, Oliveira N, Miroslavov V, Korukov G, Ravendran K, Shah N, Mitkov Y, Arivanandan K, Gaikwad R, Gotru S, Saraff R, Sain N, Stanchev D, Gaydarski L, Ali U, Kandathil LJ, Krishnaveni A, Ivanov I, Ganev I, Krapeshki Т, Miza M, Mumu MSK, Bashliev I, Sajjad A, Chandel L, Yordanov D (Alexandrovska University Hospital); Hristova E*, Spassov K, Tsankov T (Fifth City Hospital); Ivanov T*, Filipov E, Georgiev D, Neykov V, Daleva E, Ilieva I, Krasimirova D, Hristov H, Арсова М, Soladoye O, Biswakarma A, Raj R, Negi A, Dey A, Adidela JK, Alionye UG, Ndukwe A (Heart and Brain Hospital); Dimov R*, Ivanov V, Sulcheva S, Mitrev S, Nikolov V (Kaspela); Karamanliev M*, Dimitrov D, Yordanov A, Vladova P, Shoshkova M, Karakadieva K, Gabarski A, Shanker A, Ehtisham H, Parambi S, Tranchev L, Vasileva A, Kareem J, Ezeabasili J, Madueke-Ediae OL, Muradia T, Saini A, Rana I, Kanagaratnam R, Thomas LS, Galasyuk M, Costa JJ, Majid N, Reddy P, Abdullahi M, Koshy Thomas S, Mehta A, Biswakarma A, Raj R, Shittu S, Theophilus J, Theophilus J, Ilyas-Uddin R, Ogunleye I, Naghavi E, Kiran A, Sundar A, Joy A, Ali F, Angelova I (UMHAT 'Georgi Stranski'); Slavchev M*, Belev N, Atanasov B, Krastev P (UMHAT Eurohospital Medical University of Plovdiv); Yotsov T*, Dimitrov D, Kamenova P, Mihaylov I, Stavrov A, Vricheva A (University Hospital Medika).

**Croatia**: Šantak G* (County General Hospital Pozega); Penezić L*, Kastelan Z, Zimak Z, Saić H, Knezevic N, Cikic B, Zekulić T, Hudolin T, Juric I, Anđelić J, Kulis T, Maric M (University Hospital Centre Zagreb); Mihanovic J*, Soric T, Surić Grčić D, Blagus L, Kovačević P, Vidić I, Miočić Juran A (Zadar General Hospital).

**Cyprus**: Ergatidou C*, Gravas S, Yiallourou A, Achilleos S, Vardas M, Kokkinos G, Panagiotou C, Kyprianou M, Kyriacou K, Papatheodorou P, Konstantinou K, Onisiforou D, Tsoutsouki F, Kasapi A, Demetriou E, Stylianou A, Xenophontos E, Georgiou P, Makrides P, Pozotou I, Constantinou M, Evangelou T, Varavina A, Charitonos S, Lampi M (Nicosia General Hospital).

**Czech Republic**: Attaalla M*, Dušek T, Ndukwe M, Kuzmane L, Moorlata E, Sillah H, Kotek J, Fazal J, Ssali E, Steingauer V, Vinklerova K, Ahmad O, Rodríguez Martínez Á, Hiebert E, Srivastava M, Ramesh Babu AS, Dobra O, Akiba A, Kalou MF, Sokolová T, Nepeřená N, Novotný P, Sedlackova J, Philips F, Al Atassi H, Jose P, Sunil Nair A, Ntashamaje L, Kaddah A, Antabi MA (Fakultní Nemocnice Hradec Králové); Kocián P*, Fiala R, Harustiak T, Vesely S, Haluza A, Štekrtová A, Král V, Zdichová K, Pastor J, Gorchakov A, Novák H, Novák V, Hornak J, Havova M, Marková M, Fořtová T, Lérias Bento D, Novysedlák R, Smetana JF, Novák T, Zdobinska T, Švarc M, Hejduková E, Havlová K, Vaishevich A, Čorňáková J, Zajíc P, Francúz P, Příman O, Havlíková Š, Zemlickova B (Motol University Hospital); Whitley A*, Gurlich R, Balaz P, Tomyak I, Kútik M, Molva O, Šturc D, Belbl M, Vinklarkova T (University Hospital Kralovske Vinohrady).

**France**: Villefranque V*, Police A*, Mikhael E, Mabilia A, Charre L, Volpin E, Braham H, Montero Macías R, Krief D, Boyer De Latour A (Hôpital Simone Veil); Angeles MA*, Martinez A, Navarro A, Ferron G, Del M, Ghiani M, Migliorelli F (Institut Claudius Regaud); Danguy Des Déserts M*, Johan S, Olivier B, Andro C (Military Hospital Clermont Tonnerre (Hôpital des Armées)).

**Germany**: Von Wedel D*, Kamphues C, Thiele M, Felber J, Neudecker J, Schineis C, Lauscher JC, Stephan R, Meyer LP, Wolff C, Bombera B (Charité University Medicine); Rolinger J*, Kirschniak A, Wilhelm P, Göller S, Van Den Hil L (Kliniken Maria Hilf); Reim D*, Kießler M, Buschhaus C, Naisar S, Zahlmann N, Walter RL, Weber M, Müller K, Schweden A, Berlet M, Jorek NA (Klinikum Rechts der Isar TUM School of Medicine); Seyfried S*, Rahbari N, Birgin E, Reissfelder C, Reeg A, Téoule P, Rasbach E (Mannheim University Medical Center (Universitätsmedizin Mannheim)); Pyrgidis N*, Hatzichristodoulou G, Sokolakis I (Martha-Maria Hospital); Strotmann JJ*, Fahlbusch T, Höhn P, Hinrichs J, Horn J, Jollet S (St. Josef); Binder J*, Förtsch T, Winterstein M, Hackner D, Girard A (Universitätsklinikum Erlangen); Wittmann M*, Vilz TO, Recker F, Velten M, Massoth G, Delis A, Brugués Villalba MI, Willis M, Soltau S, Kudaliyanage S, Eymael RL, Syring I, Neubauer R, Dahmen N, Zimmermann A, Straßberger-Nerschbach N, Puskarevic A, Wang X, Peyman L, Philippi RA, Schmidt K (University Hospital Bonn); Bork U*, Schepp F, Korn S, Radulova-Mauersberger O, Von Dem Bussche J, Von Bechtolsheim F, Praetorius C, Knobloch N, Oehme F, Weitz J, Mansfeld M, Distler M (University Hospital Carl Gustav Carus); Klingler F*, Fluegen G, Dávid S, Schneider M, Baba KA (University Hospital Duesseldorf); Ronellenfitsch U*, Kleeff J, Ukkat J, Klose J, Bayram O, Sommerer M, Rebelo A (University Hospital Halle); Gockel I*, Gentsch E, Rayes N, Thieme R, Kreuser N, Aktas B, Branzan D, Seehofer D (University Hospital Leipzig); Mallmann MR*, Mallmann C, Woldu S, Balci F, Domröse C, Diekhöner M (University Hospital of Cologne).

**Greece**: Micha G*, Stroumpoulis K, Dritsoulas L, Kalopita K, Bitzi G, Thessalonikefs P ('Elena Venizelou' General and Maternity hospital of Athens); Fradelos E*, Korkolis D, Sarafi A, Manatakis DK, Tasis N, Prountzopoulou A, Tziava E (Agios Savvas Anticancer Hospital); Pikoulas K*, Paramythiotis D, Tishukov M, Papadopoulos K, Chatziantoniou G, Tzikos G, Papamichail S, Bareka S, Sarafis A (AHEPA University General Hospital); Haidopoulos D*, Angelou K, Prodromidou A, Stamatakis E, Alexakis N, Pergialiotis V, Rodolakis A, Thomakos N (Alexandra General Hospital); Memos N*, Vlahos N*, Koutalas I, Kotsis T, Kalampokas E, Chardalias L, Kontopoulou C, Lignou C, Lykourgioti P, Bagiasta A, Petrolekas A, Savranakis O, Kozonis T, Themelidi V, Mccormac-Prekeze M, Nikolaou C, Bletsa E, Melissaridou N, Flamourakis K, Rammos P, Hadjizacharias T, Provata N, Politis D, Gkioulekas AP, Massaras D, Vakos I, Antonoglou A, Dimitra P, Kotzadimitriou A, Evangelou C, Psychogios D (Aretaieion Hospital); Konstantinidis MK*, Apostolou K*, Konstantinidis K, Patelis N, Konstantinidou S (Athens Medical Center); Kokoropoulos P*, Michalopoulos N, Kratiras Z, Drakakis P, Sidiropoulos T, Papadoliopoulou M, Vassiliu P, Arkadopoulos N, Koratzanis C, Vrachnis N, Dylja E, Chatzialis I, Sampanis D, Danias N, Fotiou A, Stavros S, Petropoulou Z, Tsaousis V, Papakonstantinou D, Lykoudis P, Nastos C, Charalampopoulos A, Papiri IE, Hatzaras I (Attikon University General Hospital); Paraskevas KI* (Central Clinic of Athens); Petrakis G*, Polenta E, Kaparounaki E (Chania General Hospital 'St George'); Sotiropoulou M*, Kapiris S, Mavrodimitraki E, Paraskeva A, Kolinioti A, Psarologos M, Stergiou D, Metaxas P, Stamatis K, Kyzeridis C, Kefalou E (Evaggelismos General Hospital); Vrakopoulou GZ*, Larentzakis A, Menenakos E, Maravgaki VR, Georgiadou C, Koutrouli M, Papageorgiou A, Skandali A (Evgenideio Hospital); Tzovaras G*, Baloyiannis I, Tzortzis V, Christodoulidis G, Perivoliotis K, Arnaoutoglou E, Ntalouka MP, Chatzis A, Daponte A, Samara A, Donoudis C, Zacharoulis D (General University Hospital of Larissa); Mulita F*, Bouchagier K, Verras G, Tchabashvili L (General University Hospital of Patras); Ioannidis O*, Koltsida A, Malliora A, Paparouni D, Bitsianis S, Loutzidou L, Anestiadou E, Χατζηανεστιάδου Χ, Simeonidis S, Tekelidis A, Zapsalis K, Skalidou S, Ouzounidis N, Ntampakis G, Kelepouri A, Kontidis F, Foutsitzis V, Kontaxi O, Barakakis G, Valaroutsou OM, Athanasiou C, Kotidis E, Pramateftakis M, Mantzoros I (George Papanikolaou General Hospital of Thessaloniki); Toutouzas K*, Frountzas M, Triantafyllou T, Triantafyllou A (Hippocratio General Hospital); Dagklis T*, Katsanos G*, Petousis S*, Athanasiadis A, Tsoulfas G, Dinas K, Tsakiridis I, Mamopoulos A, Kalogiannidis I, Rao F, Christou C, Vasileiadou S, Kapetanios G, Tsakiridis N, Kopatsaris S, Papanikolaou E, Karakasi KE, Tataridou TA, Antoniadis N, Zachomitros F, Arvanitaki A, Tsakiridis K, Anemoulis M, Neiros S, Ouranos K (Hippocratio Hospital); Tampaki EC*, Maltezos C, Maltezos K, Anastasiadou C, Chaveles A, Pachi A (KAT Athens General Hospital); Tsiantoula P*, Roditis K, Antoniou A, Bessias N, Papas T, Tzamtzidou S, Maras D, Papaioannou V (Korgialenio-Benakio Hellenic Red Cross Hospital); Koukoulis G*, Bouliaris K, Skriapas K, Kontopoulos G, Doudakmanis C, Kolla C, Efthimiou M, Kalfountzos C, Mitsakou D, Kardasi C, Zourntou S, Fountarlis LI, Bakalis A, Samarinas M, Chatzilamprou PL, Migdanis A, Karvouni K, Katsiafliaka K, Arvaniti C, Papazisi AR, Markatou V, Zervas K, Marsitopoulos K (Koutlimbaneio and Triantafylleio General Hospital of Larissa); Machairas N*, Dorovinis P, Kotsifa E, Keramida MD, Schizas D, Vailas M, Syllaios A, Mela E, Hasemaki N, Skotsimara A, Katsargyris A, Kykalos S, Tomara N, Palios I, Karniadakis I, Charalabopoulos A, Sakarellos P, Davakis S, Kydonakis N, Tsourouflis G, Stamopoulos P, Laios K, Kozadinos A (Laiko University Hospital); Katsaros I*, Kontis E, Iavazzo C, Katsiaras L, Kaouras E, Manikis P, Kokkali K, Theodorou G, Dragi A, Vorgias G (Metaxa Cancer Hospital); Tzelves L*, Skolarikos A, Manolitsis I (Sismanoglio General Hospital of Athens); Spartalis M*, Tzima I, Anastasiou A, Pantelidis P, Schismenou G, Spartalis E, Zakynthinos GE (Thoracic Diseases General Hospital Sotiria); Lasithiotakis K*, Petra G, Lampou M (University Hospital of Heraklion Crete).

**Hungary**: Toth D*, Varga Z, Pósán J, Illésy L, Váradi C (Debrecen University Clinical Center).

**Italy**: Santarelli M*, Puca L, Brignone L, Lisa Marie R, Silvia L, Silvia P, Ferguglia A, Piceni C, Improta M, Assanti F, Montanari E ('Città della Salute e della Scienza' di Torino); Ettore G*, Cannone F, Cormaci L (ARNAS Garibaldi "Nesima"); Nicastro V*, De Carlis R*, Ferrari G, Giani A, Grimaldi S, Gregorio L, Mazzola M, Ripamonti L, Lorusso L, Tartufari GA, Magistro C, Benedetti A, Gonta A, Maesano J, Bertoglio CL, Giusti I, Quagli O, Brucchi F, Bevilacqua E, De Carlis L, Lauterio A, Cerchione R, Migliorini M, Incarbone N, Centonze L, Darwish SS, Vella I, Buscemi V, Podestà A (ASST Grande Ospedale Metropolitano Niguarda); Desio M*, Berselli M, Megna S, Cocozza E, Livraghi L, Quintodei V, Marchionini V, Borroni G, Peverelli C, Corbella M (ASST Settelaghi); Ferrari F*, Odicino F, Gozzini E, Cisotto F (ASST Spedali Civili Ospedale di Brescia); Baronio G*, Del Giudice R, Sala G (ASST Valcamonica Ospedale di Esine); Iacomino A*, Vennarecci G, Ferraro D, Di Martino M, Pisaniello D, Falaschi F (Azienda Ospedaliera di rilievo nazionale Antonio Cardarelli); Petagna L*, Giuliani A, Di Lascio P, Coluzzi M, Pascale G, Gallicchio P, Frezza G, Dinatale G (Azienda Ospedaliera Regionale 'San Carlo'); Cerino G*, Bottari A*, Martellucci J, Maltinti G, Scheiterle M, Fortuna L, Staderini F, Coratti F (Azienda Ospedaliera Universitaria Careggi); Veroux M*, Giaquinta A*, Roscitano G*, Volpicelli A*, Veroux P, Palumbo M, Riccioli G, Stella L, Distefano C, Gioco R, Lomeo G, Lomeo E, Cavallo M, Virgilio C, Molino C, Giambra MM, Zerbo D, Santini R, Costa S, Incognito GG, Centonze DC (Azienda Ospedaliero); Currò G*, Ammendola M, Filippo R, Ammerata G (Azienda Ospedaliero Universitaria Mater Domini); Balestri R*, Morelli L*, Tartaglia D*, Puccini M, Asta VF, Conte V, Buccianti P, Sargenti B, Boni G, Signori S, Pezzati D, Urbani L, Casale G, Taddei G, Di Franco G, Bianchini M, Carpenito C, Furbetta N, Comandatore A, Tarasco F, Guadagni S, Mastrangelo M, Gianfaldoni C, Palmeri M, Fatucchi LM, Boato AB, Gianardi D, Stingone M, Sacco L, Giaquinto S, Lami L, Iuliano M, Annunziata EA, Chiarugi M, Strambi S, Coccolini F (Azienda Ospedaliero Universitaria Pisana); Anania G*, Chiozza M, Campagnaro A, Nevoso V (Azienda Ospedaliero Universitaria San'Anna); Carbone L*, Marano L, Micheletti G, Fontani A, Malerba S, Gambelli M, Bombino A, Grassi G (Azienda Ospedaliero Universitaria Senese); Mandato VD*, Aguzzoli L, Mastrofilippo V (Azienda Unità Sanitaria Locale); Fabbri N*, Feo CV, Ginestri M, Oppici D, Torchiaro M, Pesce A (Azienda Unità Sanitaria Locale di Ferrara); Catena F*, Vallicelli C (Bufalini Hospital); Murzi V*, Podda M, Pisanu A, Campus F, Pilia T, Saba A, Piras C, Pisano M, Locci E, Marongiu P, Gessa E (Cagliari University Hospital); D'Oria Mario*, Biloslavo A*, Lepidi S, Germani P, Nappi CJ, Grando B (Cattinara University Hospital); Lauretta A*, Pollesel S, Brollo PP (Centro di Riferimento Oncologico di Aviano (CRO)); Gallo G*, Trompetto M, Realis Luc A, Tiesi V (Clinica S. Rita); Porcu A*, Perra T, Madonia M, Tedde A, Scognamillo F, Tilocca PL, Delogu D, Mucci G, Drocchi G, Tedde M, Scanu AM, Rizzo G, Fancellu A, Feo CF, Farina G, Casu R, Cossu ML, Ginesu GC, Anania M, Dessole S, Capobianco G, Petrillo M, Dessole F, Angelone I (Cliniche San Pietro); Barberis A*, Azzinnaro A, Petrungaro A, Palli E, Mina E (E.O. Ospedali Galliera); Pipitone Federico NS*, Muratore A, Calabrò M, De Zuanni M, Herranz Van Nood E (Edoardo Agnelli); Licari L*, Callari C, Di Miceli D, Viola S (FBF Buccheri La Ferla Palermo); Manigrasso M*, Caricato C*, Milone M, Anoldo P, Vertaldi S, D'amore A, De Palma GD, Marello A, Cantore CG, Chini A, Maione F, Luglio G, Pagano G, Tropeano FP, Cricrì M, Aprea G, Palomba G, Capuano M, Basile R, Bracale U, Peltrini R, Visconti M, Magno G, Di Santo Albini AG (Federico II University of Naples); Fransvea P*, Sganga G, Tropeano G, Puccioni C, Altieri G, Fico V, Mirco P, Bianchi V, Cozza V, Pepe G, Alfieri S, Rosa F, Schena CA, Laterza V, Pascale MM, Agnes S, Bianco G, Frongillo F, Ferracci F, Santullo F (Fondazione Policlinico Universitario Agostino Gemelli); Balla A*, Lepiane P, Saraceno F (Hospital "San Paolo"); Mastroianni R*, Simone G, Tuderti G (IRCCS 'Regina Elena' National Cancer Institute); Marino F*, Lantone G, Lantone G, Isernia R, Pezzolla F, Aquilino F (IRCCS 'Saverio de Bellis'); Raimondo D*, Gori A*, Seracchioli R, Rottoli M, Belvedere A, Maurino L, Russo IS, Orsini B, Maletta M, Romano A, Bernante P, Violante T, Morezzi D, Degli Esposti E, Raffone A, Poggioli G, Canavese A, Dajti G, Cardelli S, Casadio P, Arena A, Sanna G, Cuicchi D, Angelicchio LF, Catalioto C, Torre B, De Iaco P, Tesei M, De Crescenzo E, Larotonda C, Greco F, Minghetti M, Sissa L, Perrone AM, Dondi G, Di Stanislao M, Gaetani L, Serafini L, Prosperi E, Perone D, Isopi C, Bruno F, Alexa ID (IRCCS Azienda Ospedaliero); Milito P*, Lovece A, Chiappini G, Froiio C, Panici Tonucci T, Saletta G, Scardino A (IRCCS Policlinico San Donato); Belli A*, Izzo F, Patrone R, Cutolo C, Palaia R, Granata V, Albino V, Leongito M, Piccirillo M, Pasta G, Delrio P, Rega D, Rho M, Aversano A, Angelone IA, D'amico M, Di Marzo M, De Franciscis S (Istituto Nazionale Tumori IRCSS Fondazione G. Pascale); Cianci P*, Restini E, Conversano I, Scialandrone G (Lorenzo Bonomo); Di Saverio S*, Cardinali L, Travaglini G, Sebastiani E, Marziali I (Madonna del Soccorso Hospital); Bellocchia AB* (Maria Vittoria Hospital); Raimondo I*, Di Giorgio D*, Garganese G, Tondolo V, Dore A, Pacini G, Turco LC, Campennì P, Amara MA, Rubattu A, Verri D, Sole G, Bove S, Inzaina F, Franceschi F, Grande G, Pili DP, Campus F, Delogu G, Sula V, Ez Zinabi O, Congiu M, Pira E (Mater Olbia Hospital); Ercolani G*, D'acapito F, Annunziata D, Solaini L (Morgagni); Giordano A*, Cantafio S (Nuovo Ospedale di Prato); Novello S*, Romano M, Armellin C, Zanus G, Milardi F, Grossi U, Baldan R, Brizzolari M, Scolari F, Brun-Peressut A (Ospedale Ca' Foncello); Broglia A*, Martorana M, Fazzini N (Ospedale Civile di Voghera); Marafante C*, Garino M, Moggia E, Agosti SC, Battaglia A, Borello A, Birolo SL, Barone R, Mosca C, Shakhova K, Apostu AL (Ospedale degli Infermi di Rivoli); Giaccari S*, Pavanello M, Migliori E, Sambucci D (Ospedale di Conegliano); Corbellini C*, Sampietro GM, Germiniasi G, Bischeri M (Ospedale Di Rho); Colombo F*, Danelli P, Cammarata F, Zingale RS, Pezzoli I, Carbonaro M, Albanesi F (Ospedale Luigi Sacco Milano); Cozzani F*, Rossini M, De Gennaro F, Giuffrida M, Del Rio P (Ospedale Maggiore di Parma); Inama M*, Moretto G, Iudici L, Vitali A, Creciun M, Impellizzeri H, Piazza M (Ospedale Pederzoli); Biancafarina A*, De Prizio M, Malatesti R, Sulce R, Pellicano’ GA, D'ignazio A, Mariottini V, Mura G, Angelini M, Scricciolo M, Barbara F, Tofani F, Nenci L, Borgogni V, Vece C (Ospedale San Donato USL Toscana Sud Est); Sagnotta A*, Solinas L, Rossi M, Mancini S (Ospedale San Filippo Neri); Fruscio R*, Ciulli C*, Braga M, Romano F, Garancini M, Carissimi F, Vico E, Delle Grazie CDG, Negri S, Corti J, Tamini N, Cigagna L, Giordano R, Davolio A, Trezzi G, Grassi T, Procaccianti C, Nespoli LC, Pitoni L, Fogliati A, Cordaro G, Passoni P, Fantauzzi M, Villa S, Scotti MA, Di Lucca GM, Benedetti F, Masseria P, Frigerio M, Barba M, Re I, Ceresoli M, Ferraina F, Adjei Antwi SK, Fumagalli C, Di Martino G, Di Meo ML, Bazzano L (Ospedale San Gerardo); Nelli T*, Masciello F*, Canonico G, Chisci E, Adinolfi E, Borreca R, Damigella A, Di Martino C, Mottola GIL, Fontani G, Michelagnoli S (Ospedale San Giovanni di Dio); Fedi M*, Leo F, De Vincenti R, Cecchi C, Piombetti L, Giannessi S, Benedetta B, Pagani M (Ospedale San Jacopo); Epis L*, Percivale A, Malerba M (Ospedale Santa Corona); Tonini V*, Cervellera M, Shahu J, Sartarelli L (Ospedale Santissima Annunziata); Luzzi A*, Romairone E, Carrabetta S, Tuminello F, Floris F, Ré F, Marzorati S, Ballari F, Meola C, Filippelli A, Ribeca UG, Viacava A (Ospedale Villa Scassi); Lizzi V*, Montagna M, Giuliani A, Tartaglia N, Vovola F, Maffei F, Cotoia A, Pacilli M, Pavone G (Ospedali Riuniti Azienda Ospedaliera Universitaria); Berardi G*, Depalma N, Maruccio M, Spampinato MG, D'ugo S, Marchese T, Basurto F, De Giorgi C, Garritano S, Manoochehri F, Perrone F, Botrugno I, Rizzi A, Sergi W (P.O.'Vito Fazzi'); Russo GI*, Cappellani A, Cimino S, Asmundo MG, Matarazzo MG, Di Vita M (Policlinic Hospital 'G. Rodolico'); Venturelli P*, Cocorullo G, Zarcone F, Dominici DM, Salamone G, Tutino R, Corigliano A, Guercio R, Giuseppe C, D'arpa F, Melfa G, Proclamà MP, Scerrino G, Canfora I, Marcianò M, Guercio G, Barbera S, Amato C, Agostara AV, Pantuso G, Orlando G, Finocchiaro N (Policlinico 'P. Giaccone'); Picciariello A*, Altomare DF, Vincenti L, Tomasicchio G, Paradiso N, Dezi A (Policlinico di Bari); Pinotti E*, Montuori M (Policlinico San Pietro); Siragusa L*, Pathirannehalage Don C*, Sica G, Costanzo G, Usai V, Carrubba I, Pellicciaro M, Franceschilli M, Pirozzi BM, Santori F, Busso MB, Mariani Ivanikhin A, Guida AM, Sensi B, Divizia A, Blasi F, Giudice V, Orecchia L, Angelico R, Bacchiocchi G, Tariciotti L, Billeci F, Quaranta C, Cascone C, Materazzo M, Toti L, Manzia TM, Di Lorenzo N, Monaco A, Pedini D, Bagaglini G, Keçi L (Policlinico Tor Vergata Hospital); Lapolla P*, Mingoli A, Brachini G, Cirillo B, Sapienza P, Cicerchia PM, Leonardo C, Bologna E, Licari LC, Zambon M, Mazzarella G, Falasca A, Duranti G, Flammia RS, Asero V, Bernardotto A, Scarno F, Meneghini S, Giovampietro S, Binda B, Tufano A, Palombi V, De Meis E, Rocchetti M, De Padua C, Mansi M, Bruzzaniti P, Familiari P, Nottola SA (Policlinico Umberto I); Fleres F*, Cucinotta E, Biondo SA, Viscosi F, Catarsini N, Mazzeo C, Iannello M (Policlinico Universitario G. Martino of Messina); Testa V*, Gattolin A, Rimonda R, Riente F, Travaglio E, Allisiardi F, Guffanti P (Regina Montis Regalis Hospital); Ferrara F* (San Carlo Borromeo); Barbieri MR*, Puzziello A, Mollo A, Donnarumma E, Steccanella F, Oliviero A, Lopez G, Di Maio CM (San Giovanni di Dio e Ruggi d'Aragona); Olmi S*, Di Capua F, Camillo' A, Carresi A, Uccelli M, Rubicondo C (San Marco Hospital GSD); Rosati R*, Elmore U, Puccetti F, Socci D (San Raffaele Scientific Institute); Clementi M*, Giuliani A, Tontoli S, Nisi A, Grasso A, Derraj S, Di Biasi J, Tucceri Cimini I (San Salvatore Hospital); De Nunzio C*, D'annunzio S, Nacchia A, Gallo G (Sant'Andrea Hospital); Lisi G*, Spoletini D, Signore S, Menditto R, Carlini M (Sant'Eugenio Hospital); Sasia D*, Dalmasso E*, Trucco A*, Olearo E*, Giuffrida MC, Morra I, Maione M, Puppo A, Marano A, Preve G, Vercellone B, Frola E, Beltrami E, Peluttiero I, Giraudo G, Migliore M, Armentano S, Alessandria E, Daniele A, Elisa P, Bonino L, Borghese M (Santa Croce e Carle Hospital); Bono D*, Canova G, Zago M, Nicotera A, Bonomo LD, Gattoni L (Santi Pietro e Paolo); Cillara N*, Deserra A, De Lisa A, Coccollone E, Cannavera A, Cabula R, D'agostino F, Deplano M, Chillura C, Robuschi ML, Borzacchelli A, Margiani C, Murru MC (Santissima Trinità); Pellino G*, Lieto E, Cardella F, Auricchio A, Erario SMC, Del Sorbo G, Lidia O, D'ambrosio M, Menegon Tasselli F, Mastroianni S, Selvaggi F (Universitá della Campania 'Luigi Vanvitelli'); Calini G*, Terrosu G, Clocchiatti L, Morinelli V, Valotto C, Traunero F, Vizzielli G, Restaino S, De Gennaro E, Baccarani U, Andriani A, Mauro J, Frigatti P, Martin E, Pregnolato S, Driul L (University Hospital of Udine); Zullo A*, Carcano G, Latham L, Inversini D, Vigezzi A, Ietto G, Marta R, Marzorati A, Colombo EM, Garbarino S, Palamara N, Tozzi M, Piffaretti G, Franchin M, Festi LF, Odeh M, Fanelli MC, Costa J, Pappalardo V, Scorza A, Cannavò M, Ballabio A (University of Insubria Ospedale di Circolo e Fondazione Macchi (Varese)); Barina A*, Franzato B, Rubin L, Brugnolo D, De Simoni O (Veneto Institute of Oncology (IOV) IRCCS).

**Latvia**: Senica SO*, Ozoliņš A, Vjaters E, Jain N, Apse RR, Truskovs A, Apse IA, Jukonis P, Jukonis J, Tumova A, Sivapalan H, Piirtola OSS, Berga AE, Tumegård H (Paula Stradiņa Klīniskā universitātes slimnīca).

**Lithuania**: Samalavicius N*, Nutautiene V, Aliosin O, Eismontas V (Klaipeda University Hospital); Dauksa A*, Venclauskas L, Jasaitis K, Lazauskaitė D, Urbonas K, Šlenfuktas V, Grikytė I, Jokubauskas M, Simanaitis V, Dauksa Z, Gudaityte R, Riauka R, Krunkaitytė U, Svagzdys S, Šikarskė A, Pakrosnyte J (Lithuanian University of Health Sciences Kaunas Clinics); Stratilatovas E*, Piscikaite A (National Cancer Institute); Žaldokaite G*, Poskus T, Olekaitė V (Vilnius University Hospital).

**Malta**: Psaila J*, Andrejevic P, Cini C, Cassar K, Mattocks S, Sammut M, Mizzi S, Abela L, Micallef A, Pace K, Schembri B, Cini Custo R, Harmsworth M, Farrugia B, Theuma F, Gatt R, Montebello G, Spiteri N, Galea J, Vassallo L, Fava L, Ebejer A, Carabott K, Iles K, Casingena L, Apap Bologna G, Debono G, Galea J, Sammut M (Mater Dei Hospital).

**Netherlands**: Huisman S*, Leclercq W (Máxima Medical Center); De Ruijter F*, Konsten J (VieCuri Medisch Centrum).

**North Macedonia**: Adramanova D*, Nikolovski A, Otljanski A (University Surgery Clinic "Sv. Naum Ohridski").

**Poland**: Kisielewski M*, Richter K, Kłos N, Stefura T, Macheta K, Alsoubie I, Tempski J, Wolińska N, Wysocki W (5th Military Clinical Hospital); Starek E*, Nowak NG*, Tarkowski R, Wallner G, Kobak J, Mączka E, Żak K, Ziółkiewicz A, Kułak K, Langa E, Łaba Ł, Krzesińska I, Bobiński M, Frankowska K, Dziurda S, Martyna J, Radulski J, Lulko O, Tomczyk J, Jasiński M (Independent Public Teaching Hospital No 1 in Lublin); Major P*, Rymarowicz J, Zielińska Z, Siuda K, Kęska M, Wites P, Błasiak P, Sierżęga A, Kuciel J, Tomczak D (Jagiellonian University Medical College); Molasy B*, Nieroda P, Buras E (St. Alexander Hospital).

**Portugal**: Gonçalves Pereira R*, Patrocínio S, Reis S, Rolo Santos C, Moniz L, Bernardo P (Centro Hospitalar Barreiro Montijo); Osório C*, Matos L, Carvalho L, Marques Antunes J, Alçada N (Centro Hospitalar de Entre o Douro e Vouga - Hospital de São Sebastião); Marçal A*, Tenreiro N, Martins D, Leal C, Ferreira C, Marques R, Freitas F, Marques C, Melo A, Silva S, Vieira B, Fernandes U (Centro Hospitalar de Trás-Os-Montes e Alto Douro); Freire B*, Fonseca S, Reis M, Macedo Cardoso De Oliveira C, Mendes J, Carvalho M, Macedo J (Centro Hospitalar do Medio Ave); Oliveira J*, Mexedo C, Sousa Silva D, Davide J, Emanuel Do Vale Gonçalves De Castro Alves E, Ginestal M, Santos MD, Santos J, Robalo C, Valente V, Fernandes Dos Santos JP, Mesquita I, Teixeira B, Marques Monteiro M, Silva MI (Centro Hospitalar do Porto); Nunes Coelho M*, Teixeira M, Silva RL, Lopes L, Ribeiro A, Lima Da Silva C, Correia De Sá T, Martins M, Costa M, Gil CG, Barros M, Santos RF, Silva A, Pedro J (Centro Hospitalar do Tamega e Sousa); Costa M*, Ventura Antunes TA, Pascoal M, Andrade R, Duque M, Prior I, Lemos M, Pinto P, Guerreiro P (Centro Hospitalar e Universitário de Coimbra - polo HUC); Castanheira Rodrigues S*, Barbosa E, Martins AL, Pereira A, Vaz S, Fareleira A, Rocha-Neves J, Dias L, Girão De Caires F, Pereira-Neves A, Oliveira AI, Araujo Teixeira JP, Ferreira J, Almeida M, Vb Machado M, Nogueiro J, Gomes F, Campos E, Jácome F, Coutinho C, Ribeiro Dias R, Vieira De Sousa JP (Centro Hospitalar e Universitário de São João); Francisco E*, Borges C, Pereira S, Pereira C, Machado N, Calaia R, Pinto J, Corvelo Pavão T, Santos Sousa LG, Cunha F, Melo Pinto D, Pereira R, Dinis I, Ferros C, Pinheiro JL, Santos AJ, Barbosa B, Gaspar D, Pinto M, Pereira D, Araújo N, Alves B (Centro Hospitalar Tondela); Barbosa H*, Silva Araújo D, Afonso M, Guimarães B, Campos Coroa M (Centro Hospitalar Vila Nova de Gaia/Espinho); Susano MJ*, Azevedo J, Pereira M (Fundação Champalimaud); Miranda P*, Garrido R, Oliveira Mourão M, Ferreira M, Duarte A, Félix P, Antunes I, Fernandes N, Gil Corrêa Figueira C, Figueiredo I, Gututui M, Oliveira S (Hospital Beatriz Angelo); Silva A*, Madeira Martins F, Cabral A, Rodrigues CS, Gama B, Silva Borges R (Hospital da Horta); Lourenço R*, Longras AC, Araújo R, Peixoto I, Santos A, Matos D, Lopes A, Claro L (Hospital da Senhora da Oliveira); Cardoso D*, Martins A, Silvestre M, Sousa G, Santos Bessa M, Cardoso V (Hospital de São Francisco Xavier); Velez C*, Machado A, Pedroso De Lima R, Matias MI, V. Matos S, Rocha Melo M, Bolota J, Rente M, Santos A, Antunes ML, Bárbara A, Rosin E, Oliveira J, Leandro S, Damasio Cotovio M, Morgado S (Hospital do Espirito Santo); Fialho G*, Andrade N, Valente Costa Pinto F, Capote H, Morais S, Buruian M, Taré F, Santos G, Rosado D, Costa C, Mogne T, Pratas N, Cordeiro B, Brito M, Salvador D (Hospital Doutor José Maria Grande); Carvalho S*, Capella V, Carracha M, Alves R, Miranda C, Rebelo F, Teles T, Ferreira M (Hospital Dr. Antonio José de Almeida); Cismasiu B*, Costa PM, Henriques S, Trindade M, Vaz J, Gomes Vieira M, André M, Macedo A, Gonçalves Nobre JG, Prosil Sampaio J, Mira AR, Preto Barreira AL, Botelho P (Hospital Garcia de Orta); Melo Pinto D*, Quintela C, Quelhas MJ, Pereira D, Cruz A, Mesquita Guimarães C, Pinto C, Maldonado F, Sales F, Marrana F, Reis Aguiar AR, Freire L, Farias L, Faria-Costa G, Castro A, Moreira Marques T, Cardoso G (Hospital Pedro Hispano); Ribeiro J*, Fragoso M, Silva C, Vasconcelos M, Picciochi M, Sampaio Soares A, Frazão J, Miranda Pera R, Gaboleiro F, Ramalho De Almeida F, Mendes A, Afonso F, Fontaínhas J (Hospital Professor Doutor Fernando da Fonseca); Reia M*, Angel M, Aguero C, Guerrero M, Dominguez J (Hospital Santa Luzia de Elvas); Borges Da Costa I*, Ramos L, Fernandes J, Assis C (Hospital Vila Franca de Xira); Fonseca F*, Carvalhal S, Caiado A, Brito Da Silva F, Miguel B, Moniz J, Pires M, Leal B, Nunes J, Matos Sousa F (Instituto Português de Oncologia de Lisboa Francisco Gentil); Vieira Caroço T*, Romano M, Ângelo M, Rodrigues Ferreira A, Prata Saraiva AR, Mano F, Rodrigues F, Branco T, Gaspar S, Neves F, Alves M (IPO Coimbra); Ferreira Pinto AP*, Peyroteo M, Baía C, Marques M, Correia AM, Silva JO (IPO Porto); Monteiro R*, Silva-Vaz P, Gomes J, Moutinho Teixeira MJ, Brito Neves T, Meruje F (Unidade Local de Saúde de Castelo Branco).

**Republic of Ireland**: Sheahan C*, Macnamara D, Linares Gómez JE, Clare S, Walmsley A, Tiwari A, Khan T, Dowling G, Hudak K, Craig E, Dhannoon A, Saeedi S, Shah J, Jordan M, Buckley T, Engler J, Reilly M, Ariaratnam J, Hinn MA, Benn K, Petropoulos S, Hansen HS, Browne S, Keelan S, Basta M, Pornsakulpaisal R, Tan G, Niyaz AN, Lakic N, Warhust T, Chang YQ, Qaoud Y (Beaumont Hospital, Dublin); Mwipatayi D*, Kearney D, O'reilly C, Gamper Y, Dhillon R, Patel V, Gallo K, Mulvaney H, Edwards P, Tiller S, Gorecki E, Jordan M, Daswani S, Jones V, Asekomhe I, Clausen N, Khan M, Feldiorean A, Pezeshki A, Khedro T, Beyer J, Byrne G, Irfan W, Poluha A, Fager C, Gilligan-Steinberg L, Yarp J, Byrne E, Lorico-Rappa M, Hassan A, Keogh T, Denning W, Horton-Schleicher K (Connolly Hospital, Dublin); Deane T*, Haren A, Md Hamsan AD, Mukerji N, Dwivedi M, Cheah SML, Kwek PX, Arshad S, Alzaki A, Cornila I, Feizal HM, Barry M, Semar A, Gorman P, Gordon A, Mcsweeny C, Layyous N, Peiris D, Pan S (St Vincent's University Hospital); Larkin J, Umar MM, Doherty C, Mitchell C, Mcelvaney H, Sabz Ali Z, Adesokan S, Murphy A, Yeo A, Hayes L, Owens C, Crawley N, Macinnis E, Elekes S, Oderoha KE, Mac Mahon I, Moran B, Matreja P (St. James' Hospital); Conlon K, Piankova P, Farran MZ, Kashyap SV, Puntambekar M, Sampat M, Heaslip Owens S, Maheshwari A, Soo A, Butt J, O'brien K, Almutairi M (Tallaght University Hospital); Mcfeetors C*, Kerin M, Davey MG, Murray W, Nasehi A, Graham A, Mathew C, Azam S, Chua Vi Long K, Stafford-Johnson S, Ejaz D, Siddiqi Z, Kon AMH (University Hospital Galway); Parmar M*, Peirce C, Shomoye D, Alabi A, Salzberg N, Gallippi V, Aziz J, Melly C, Gill H, Yang HW, Eltahir Ibrahim SE, Rugber Singh JSK (University Hospital Limerick).

**Romania**: Bacalbasa N*, Balescu I (Clinical Hospital Dr. Ion Cantacuzino); Muresan M*, Dudric V, Imihteev I, Chelaru V, Băiceanu TM, Alexandra M, Bashimov A, Voda M, Muresan D, Varadi R, Muntean M, Macovei Ș, Checiu R, Buf D (Cluj-Napoca Municipal Hospital); Grama F*, Chitul A, Bezede C, Ciofic E (Coltea Clinical Hospital); Beuca A*, Bintintan V, Muresan D, Raluca-Cristina A, Rad R, Șușman S, Suciu C, Abu Arif J, David A, Blaga M, Olesea B, Butnariu DA, Scurtu RR, Rad R, Claudia-Mihaela P, Girlovanu M, Costina D, Drasovean R, Tartiu G, Tache S, Irina N, Streang A, Trif A, Romascan I, Stănică E, Vass E, Cucoreanu C (County Emergency Hospital); Toma EA*, Calu V, Enciu O, Laceanu A, Maria-Zamfirescu R, Matache IM, Mușat I, Piriianu DC, Georgescu AA, Miron A, Tartalea M, Al-Ugeily N, Bogdan-Gabriel B, Alexiadi A, Zaharescu A (Elias Emergency Hospital); Ciubotaru C*, Negoi I, Paunescu M, Iancu I, Pop IV, Diaconescu B, Dao-Zamfirescu P, Patel NB, Rafi S, Popescu D, Girbaciu M, Rădoi A, Breuer C, Braun AC, Stoica B, Gîlia I, Gheorghe A, Popa EC, Brichius F, Bucurica A, Negoita VM, Radu M, Bianca Ștefania R, Socol CL, Baiceanu AM (Emergency Clinical Hospital Bucharest); Bubenek Turconi SI*, Valeanu L, Girel M, Gavrila MR, Pirvu M, Morosanu B, Ioniana M, Barbu A, Cornel R, Bogdan B, Bute T, Madec L, Burla E, Balan C, Alexandru E (Emergency Institute for Cardiovascular Diseases 'Prof. Dr. C.C. Iliescu'); Tomescu D*, Popescu M, Dumitru M, Marinescu A, Petrusca I (Fundeni Clinical Institute); Pasca A*, Achimas-Cadariu P, Irimie A, Petrusan A, Puscas M, Titu S, Jiboc NM, Vlad IC, Eniu DT, Ciubotariu P, Gata VA, Rancea A, Iulia C, Morariu DS, Nistor-Ciurba CC, Gale I, Lazar G, Bonci E, Ignat F, Lisencu C, Dumitraș C, Faur FI, Butuza MO, Marian MC, Paul Luchian A (Prof. Dr Ion Chiricuta Institute of Oncology); Bocse H*, Puia IC, Andra C, Pop P, Moldovan S, Ursu S, Sorin M (Regional Institute of Gastroenterology & Hepatology "Prof Dr Octavian Fodor"); Lunca S*, Dimofte M, Musina A, Velenciuc N, Roata CE, Morarasu S (Regional institute of Oncology Iasi); Velescu M*, Mureșan F, Zaha I, Rusu IM, Mircea B, Tauberg DG (University Hospital).

**Russia**: Kirov M*, Kuzkov V, Nikonov A, Tishchenko A, Kulin D, Mironov V (City Hospital #1); Litvin A*, Gunko I, Mazhega I, Bilenko R (Immanuel Kant Baltic Federal University); Khrykov G*, Манкевич Н, Karpenko M, Zagaynov E, Shilyaev A (Leningrad Regional Clinical Oncology Dispensary); Butyrskii A*, Tatidze A, Seytnebieva Z, Aliev A, Rumyantseva M (Municipal Emegency Hospital No.6); Yanishev A*, Abelevich A, Doktorov S, Zuev K, Lazareva A, Malov A (Nizhny Novgorod regional clinical hospital); Borisov D*, Борисов Е, Андрийчук И (Northern Medical Clinical Centre of them. N.A. Semashko); Galchikova Z*, Shemetova M*, Maltsagova K (P. HERTSEN MOSCOW ONCOLOGY RESEARCH INSTITUTE); Bedzhanyan A*, Temirsultanova K, Efremov D, Volkova A, Frolova Y, Minenkova A, Petrenko K, Sumbaev A, Bredikhin M, Bedzhanyan E, Mikhailova A (Petrovsky National Research Centre of Surgery); Тен В*, Кудрявцев Ю (Private Healthcare Institution "Russian Railways-Medicine""); Novikova A*, Pavlov R, Chernykh M, Boiko N, Данилин В (Saint Petersburg State University Hospital).

**Serbia**: Stanojevic G*, Brankovic B, Nestorovic M, Milutinovic N, Vukadinović A, Vukadinović A (Clinical Center Nis); Stojanovic B*, Radovanovic D, Cvetkovic A, Radosavljevic I, Sreckovic M, Milosevic B, Markovic N, Spasic M, Pavlovic M, Lazic D, Vucic R (Clinical Centre of Kragujevac); Tadic B*, Kajmaković B*, Vilendecic Z*, Knezevic D, Bumbasirevic U, Stefanovic A, Gregoric P, Perišić Z, Micic D, Ratkovic S, Vasiljević J, Bulat PR, Jovanovic V, Mitrović O, Gunjic J, Potkonjak D, Grubor N, Reljic M, Palibrk I, Milutinovic V, Zivkovic M, Škrijelj D, Grubač Ž, Kadija S, Cerovic Popovic R, Andric L, Aleksić L, Kmezić S, Pavlovic I, Djukanovic M, Milojevic B, Dzamic Z, Petrovic M, Jeremic K, Pilic I, Milosevic B, Krstic J, Šljukić V, Veselinović M, Ivanović N, Janičić A, Jovanovic A, Cvijanovic D, Ladjevic Likic I, Radojevic M, Ćupić J, Dimitrijevic I, Miladinov M, Sekulić A, Nektarijevic D, Pantovic S, Šljivančanin D, Dugalic S (Clinical Centre of Serbia); Đermanović A*, Radovanovic Z, Radovanovic D, Đurić M, Zahorjanski S (Oncology Institute of Vojvodina); Milosavljevic V*, Zdravkovic D, Toskovic B, Marjanović U, Zdravkovic M, Petricevic S, Crnokrak B, Maric S, Sekulic A, Colakovic (Ristic) N (University Hospital Medical Center Bezanijska kosa); Kovacevic B*, Krdzic I, Bojičić J, Sparic T, Ostojic A (Zvezdara University Medical Center).

**Slovenia**: Cokan A*, Košir JA*, Pakiž M, Potrč S, Kavčič N, Ivanecz A, Horvat M, Jagric T, Kacjan U, Perić I, Timošek E, Plahuta I, Turk Š, Crnobrnja B, Kovačič R, Dovnik A, Al Mahdawi L, Knez J, Grosek J, Košir Božič T, Tomazic A (University Medical Centre).

**Spain**: Suarez Gonzalez LA*, Busto Suarez S, De La Infiesta García C (Complejo Asistencial Universitario de León); Arencibia O*, Gonzalez Garcia-Cano D, Laseca M, Martin Martinez A, Guijarro Guedes JA, Rave Ramirez AF, Gil Gonzalez Y, Hernández Socorro AM, Ponce Arrocha D, Cruz García A, Mendoza Rodriguez C (Complejo Hospitalario Universitario Insular); Catala E*, Romero Garcia CS, García Trueba A, Georgieva V, Hernández Álvarez AI, Martinez-Perez C, De Valera E, Gascón JJ, Lopez Alcina E, Samper Monton L, Aghababyan K, Valero Tarin A, Salinas Lozano C, Cervera A, Catalá Bauset JC, Ramia B, Marques Peiro F, De Andres J, Iserte Juan SP, Peñalver Gaspar M, Gilabert-Estellés J (Consorcio Hospital General Universitario); De La Rosa-Estadella M*, Fernandez-Colorado A, Serrano-Martin M, Gasulla-Rodriguez A, Juarez-Pomes M (Corporación Sanitaria Parc Taulí); Ossola ME*, Sanz Ortega G, Gómez J, Del Campo M, Corrochano R, Galan Hernandez M, Molano Nogueira FJ, Ibañez Vazquez L, Dziakova J, Zarza Martín A, Infante S (Hospital Clinico San Carlos); Moro-Valdezate D*, Pérez Santiago L, Molina AI, Vinuesa Huesca A, Gadea Mateo R, Navarro-Sanchis L, Aracil-Boigues P (Hospital Clínico Universitario de Valencia); Jezieniecki C*, De Andrés-Asenjo B, Zamora Horcajada Á, Natal Álvarez F, Cuellar Martin LA, Montes-Manrique M, Cabezudo G, Gómez Sanz T, Herranz Arriero A, Sierrasesumaga N, Rodriguez-Lopez M, Ruiz Soriano M (Hospital Clínico Universitario de Valladolid); Sánchez Gollarte A*, Minaya Bravo AM, Guijarro Moreno C, Galvan A, González E, García Ureña MÁ, Robin Valle De Lersundi A, Ruiz-Tovar J (Hospital del Henares); López Campillo A*, Jimenez Toscano M, Salvans S, Jaume Böttcher SM, Godó À, Busquet Raich G, Téllez C, Sánchez García JA, Ribas Ardanuy M, Saavedra P, Sánchez Cabrero A, Clivillé-Pérez J, Durante Escutia M, Rabasa Rodríguez A, Aguilera P (Hospital del Mar); Giménez Francés C*, Ruiz-Marín M, García Escudero D, Valero Soriano M, Ramirez Faraco M, Galán Jiménez S, Alcón Cerro P, Rodríguez Lucas JM, Carrasco Prats M, Peña Ros E, López Sánchez MDP, García Porcel VJ, Tamayo M, Muñoz Camarena JM, Albarracin Garcia R, Molina O, Moreno Sánchez P, Jiménez I, Pastor Perez P, Agea Jiménez MB, Candela Mas D, Artés Artés M, Benavides Buleje JA, González Valverde FM, López-Morales P, Sanchez A, Bobadilla Romero ER (Hospital General Reina Sofía); Allue M*, Ponchietti L, Latorre Tomey R (Hospital General San Jorge); Torres Ramos D*, Jimenez-Gomez LM, Valdés López-Linares S, Moreno Salazar YT, Soto Galán J, Moreno De Alboran C, Prosperi A, Osma JJ, Blanco A, Sánchez Rodríguez M, Valdivielso E, Cejudo Á, Perez Carpio C, Vasques Seabra Águas F, Sánchez Martín E, Pérez-Ajates S (Hospital General Universitario Gregorio Marañón); Macarulla E*, Álvarez Torrado A, Galmés C, Artigot M, Marin Garcia J (Hospital Igualada); Oliver Guillen JR*, López De Fernández A, Fernández-Velilla San-José BF (Hospital Santa Bárbara); Ambrona Zafra D*, Pérez Farré S, Ortega Alcaide J, Codina Corrons L, Sisó Soler E, Gómez Báez FD, López-Soler G, Gutiérrez Pérez E (Hospital universitari Arnau de Vilanova); Aix Molina S*, Vallve-Bernal M, Varona Mancilla A, Aldama Martín ÁM, Casanova R, Sevillano L, Muela JJ, Rodrigo Rodrigo M, Goujon I, Fructuoso Iniesta C (Hospital Universitari De Tarragona Joan XXIII); Pérez-Bertólez S*, Monclus E, Fernández L, Silva Hernández MG, Vicario F (Hospital Universitari Dexeus); Garcia Pimentel P*, García Álvarez R, Bouzó Molina N, Ramiro Á, Calderon Barajas ZA, Sanz R, Xia Z, Rodríguez P, Sarrais Polo C, Martínez López A, Estrada C, Gómez De Castro PE (Hospital Universitario 12 de Octubre); Fernández Martínez D*, García Flórez LJ, De Santiago Alvarez I, García Alonso L, García-Santos G, García González LA, Carrasco B, Silva-Cano DW, Del Val Ruiz P, Martínez Izquierdo G, Corteguera A, Iturbe J, López-Negrete Cueto E, Cembellin A, Ramos Montes C, Ibero Casadiego GP (Hospital Universitario Central de Asturias (HUCA)); Prieto M*, Villalabeitia Ateca I, Pascual Vicente T, Villota Tamayo B, Perfecto A, Mambrilla S (Hospital Universitario Cruces); Ramos Del Moral IL*, Jiménez Carneros V, Pastor-Riquelme P, Franco Lozano A, Alonso-Lamberti L, Lozano M, García-Quijada J, Zhang R, García A, Martín MM, Yeh Ahumada M, Carreño Pallarés A (Hospital Universitario de Getafe); Pérez Rubio Á*, Goran RE, Gómez Diez N, Zomeño Bravo G, Bernal-Sprekelsen JC, Martínez Fernández GS (Hospital Universitario Doctor Peset); Marquina González R* (Hospital Universitario Donostia); Toribio-Vazquez C*, Eguibar A, Perez-Chrzanowska H, Serrano Méndez P, Valderrabano Gonzalez S, Reinoso FJ, Yebes A, Alonso Bartolomé MB, Ayllón Blanco HR, Cristóbal I, Pellicer I, Arenal González R, Zafra Angulo JD (Hospital Universitario la Paz); Duque Mallén V*, Gracia-Roche C, Gascon Ferrer I, Aparicio-López D, Antonio M, Herrero Lopez M, Saudí-Moro S, Gascón Domínguez MÁ, Gimenez Maurel T (Hospital Universitario Miguel Servet); Matías-García B*, Morales Palacios N*, Diez Alonso M, Gutiérrez A, Soto Schütte S, Bru-Aparicio M, Jiménez Martín R, Sanchez Pellejero A, Vázquez Valdés S, Vera Mansilla C, Quiroga A, Mendoza-Moreno F, Diego García L, Serrano Yébenes E, Mañes Jiménez F, Lasa I, Ovejero Merino E, Casalduero L, Allaoua Y, Blazquez Martin A, Córdova García D, Alvarado Hurtado R, Laguna Hernández P, Garcia-Moreno Nisa F, Juara H (Hospital Universitario Principe de Asturias); Aldecoa C*, Tejero-Pintor FJ, García M, Aguado Saster E, Ruiz De Santos E, Bueno Cañones AD, Pérez Fernández S, Alija Garcia MG, Urruchi R, Rioja Garrido R, Madrid M, Bordell A, Arranz Chamorro I, Rodriguez Cañal P, Pacheco Sánchez D, Marcos-Santos P, Veleda Belanche S, Maestro De Castro JL, Blanco García MJ, Laita Jiménez E, Leal L, Vaquero Perez L, Barbosa C, Ramos Carrasco M, Garcia-Saiz I, Rodicio P, Acebes García F, Lizarralde A, Sanchez Diez A (Hospital Universitario Río Hortega); Ferrer-Inaebnit E*, Segura-Sampedro JJ, Alfonso Garcia M, Gutiérrez-Cañadas GGC, Oseira A, Torres Marí N, Loyola J, Villalonga B, Camporro P (Hospital Universitario Son Espases); Pagès N*, Colás-Ruiz E, Cifuentes Rodenas JA, Castro Suárez M, Moll-Amengual R, Mata J, Fernández Manzano J (Hospital Universitario Son Llàtzer); Gómez Pérez B*, Gil Martínez J, Cerezuela Fernández De Palencia Á, Aliaga Rodriguez A, Gómez Valles P, Delegido García A, Gil Gómez E, Montoya MJ, Navarro-Barrios A, Cayuela V, Ruiz Manzanera JJ, Jiménez Mascuñán MI, Gutiérrez Fernández AI, Sánchez Esquer I, Gil Vázquez PJ, Ferreras D, Balaguer Román A, Gómez-Bosch F, Alconchel F (Hospital Universitario Virgen de la Arrixaca); Romero A*, Durán Muñoz-Cruzado VM, García Sánchez CJ, Tejero Rosado A, Ruiz B, Roman MDC, Rubio Fernández JJ, De Jesús Á, Ostos Diaz M, Quintero-Pérez C, Sánchez Marín A, Alvarez Aguilera M, Leal Mérida M, Ager I (Hospital Universitario Virgen del Rocio); Sancho-Muriel J*, Frasson M, Castro B, Guerrero P, Nieto-Sanchez M, Cruz Q, Cholewa H, Hurtado-Pardo L, Plazas D (Hospital Universitario y Politécnico La Fe); Serrano-Navidad M*, Castillo J, Ballestero R, Valbuena Jabares V, García C, Garcia Formoso N, Herrero Blanco E, Suarez Pazos N, Cagigas Fernandez C, Gomez Ruiz M, Benic Yoris YT (Marqués de Valdecilla University Hospital); Merayo M*, Gonzalez Fernandez ME, Alonso Batanero S, Rodríguez Gómez J (Universitary Hospital El Bierzo); Landaluce-Olavarria A*, Estraviz B, Markinez I, Gómez D, Fernández Gómez Cruzado L, González De Miguel M, De Francisco Rios JM, Urigüen A (Urduliz Alfredo Espinosa Hospital); Gómez Del Pulgar A*, Espin-Basany E, Gil-Moreno A, Bellmunt-Montoya S, Dopazo C, González-Suárez S, Blanco-Colino R, Olid Mayoral P, González Antúnez M, Del Barco Martínez E, Bejar Dolcet Y, Gil-Sala D, Marrero Eligio De La Puente C, Sánchez Besalduch L, Barrio Zaragoza M, Lopez Lozano JF, Olaria C, Catalan Sanz S, De Ciria E, Segura Farran L, Montes N, Zanca Gómez JM, Puig Escobar I, Armengol Alsina M, San Nicolas M, Escura M, Sánchez M, Rivas Agudo M, Aalouf L, Maya Salas D, Hernando Marín L, Umpiérrez Mayor N, Sanchez Iglesias JL, Fernández Henriquez Y, Feixa Molina I, Gomez-Jurado MJ, Bebia V (Vall d'Hebron University Hospital).

**Switzerland**: Racine M*, Sauvain M, Mujcinovic A (Hopital de Pourtales); Bolla D*, Dimitrov Y (Hospital of Langenthal); Arenja N*, Riboni C, Marx JN, Kalisvaart M, Andreou C, Brandts P, Pfefferkorn U, Eisner L, Dietz U (Kantonsspital Olten); Gass J*, Metzger J, Scheiwiller A, Kremo V (Luzerner Kantonsspital); Gero D*, Bueter M, M F, Schulz R (Männedorf Hospital); Mongelli F*, Soave I*, La Regina D, Canonica C, Sabbatini F, Gaffuri P, Spampatti S, Santarelli C (Ospedale Regionale di Bellinzona e Valli); Di Giuseppe M*, Marengo M, Passoni S, Dellaferrera G, Provenzi D (Ospedale Regionale di Locarno La Carità); Bernardi L*, Papadia A*, Cristaudi A, Gasparri ML, Aurora P, Popeskou SG, Christian P, Antonilli M, Scalvi FA, Sevas V, Palumbo G, Maoloni L, Cencetti C, Pagnani G, Hitz M, Dürmüller J (Ospedale Regionale di Lugano); Däster S*, Hess GF, Aegerter NLE, Sedlaczek P, Soysal S, Zeindler J, Angehrn F, Müller L, Taha-Mehlitz S, Haak F, Nocera F, Karli T, Lazaridis I, Tampakis A, Lalos A, Friedrich U, Wiesler B, Varathan N, Frey R (University Hospital Basel).

**Turkey**: Kurtoglu GK*, Aghayeva A, Turam C, Kurtoglu GM, Maarouf M, Baca B, Ilhan SD, Koyluoglu YO, Seker ME, Ulufi M, Erkaya M, Kılıçkan FDB, Bayrakceken S, Ozmen BB, Togay B, Doğan M, Çetinkaya T (Acibadem Altunizade Hospital); Tuzuner E*, Karahasanoğlu T, Yavuz IE, Bilgin IA, Kekik SS, Turam C, Dönmez E, Dikeç M, Ramoğlu N, Yurtseven N, Takmaz O, Orhun Ö, Karadeniz N, Saylık O, Uğur EM, Ada E, Durbas A, Hamzaoglu I, Uzun K, Gungor M, Gebedek IY, Hayırlıoğlu EÇ, Halıcı M, Ceyhan GO (Acibadem Maslak Hospital); Mutlu AU*, Aytac E, Gulmez M, Capkinoglu E, Yilmaz TU, Ozer A, Keçeoğlu S, Eraslan ZC, Ozben V, Dülgeroğlu O, Saraçoğlu C, Erkan M, Uras C (Acıbadem Mehmet Ali Aydınlar Üniversitesi Atakent Hastanesi); Copur HD*, Dogan NU, Topal E, Demirsoy I, Korkmaz D (Akdeniz University Hospital); Can U*, Buğra D, Sobutay E, Çakıt H, Ergün E, Zenger S, Bilgic C, Gurbuz B (American Hospital); Sünter K*, Gecim I, Sefer S, Inceöz B, Genc MB, Uygun AK, Yücel E (Ankara University Hospital); Barcin A*, Altinel Y, Aktimur YE, Meriç S, Özdoğan K, Sayar AI, Durmaz AG, Çakır I (Bagcilar Research And Training Hospital); Ünlü ES*, Kiran G, Dal A, Ozkan E, Kalkan S, Öncel MM, Çetin Ç, Yılmaz G, Karimi H, Iskurt Y, Yardimci E, Eker M, Balkan AO, Gönül HB, Herdan E, Ertugrul M, Takmaz T, Kilinç SS, Pinar MS, Yıldıztekin F, Coşkun EC, Olgun I (Bezmialem Vakif University Hospital); Ince G*, Isik O, Şen M, Özata R, Eroğlu B, Büyükpolat B, Ishakoğlu G, Özen D, Aktaş MO, Kılıç B, Yılmazlar T, Koçbey MS, Işıklı N, Yıldız RG, Baghirov T, Uygun E, Yugaç M, Keskin E (Bursa Uludag University School of Medicine); Bozkurt BE*, Tas S, Türkmen ÖÖ, Ata B, Alan B, Işık N (Canakkale Onsekiz Mart University Hospital); Göver T*, Bisgin T, Özalp E, Sökmen S, Manoğlu B (Dokuz Eylul Univ. Hospital); Bakar C*, Bozbiyik O, Zorlu U, Zengin NN, Baki BE, Akbulut ES, Tunali S, Kutlu B, Oğuz YA, Küçükateş B, Yildiz E, Sakmar AN, Yoldas T, Akgün ZE, Korkut MA, Çalışkan C, Aksit E, Aksalman EF, Koçoğlu AO, Basci F (Ege University Hospital); Yigit B*, Ilhan O, Liman RK, Uzun M, Ağaçkıran I (Elazig Fethi Sekin City Hospital); Şahin C*, Leventoğlu S, Kubat Ö, Genç I, Oyanik A, Gurbuzer FI, Satilmis N, Cam S, Ekinci M, Gönül M, Chasan D, Zara SGF, Sök Ş, Yalcinkaya A, Acıpınar ET, Timurçin U, Afzal NB, Gillani SHA, Gillani S (Gazi University Medical Faculty Hospital); Yazıcı S*, Cennet O, Suer MS, Kaya Ö, Uslu EZ, Tanrisever A, Yildirim C, Gunenc IN, Gül B, Özkal I, Tileklioglu HI, Korkmaz MA, Şirin I, Özbayrak U, Mıdık A, Domaç E, Karakoç A, Urek S (Hacettepe university hospital); Yıldırım A*, Ugur M, Doğru M, Arslan ER, Ular B, Duymuş ME, Turhan AB (Hatay Mustafa Kemal University Training and Research Hospital); Dogan T*, Ozkan MB, Benek DE, Arslangilay M, Yildirim MB, Yazgan Y, Tıraş HN, Şahin BI, Çapar MA (Hitit University Faculty of Medicine Hospital); Tatar C*, Arı A, Batikan O, Güler M, Naycı AE, Sevinc MM, Şevik H, Oğuz C, Karagülle OO, Idiz UO, Demir A, Çakır E, Cakir C, Doğan S, Yıldız I, Gürlük M, Cengiz MB, Toptaş M, Akay O, Kaya R, Aydin E (Istanbul Education and Research Hospital); Çakıcı MÇ*, Yildirim A, Efiloğlu Ö, Demirtaş B, Iplikçi A, Atis G, Keser F, Culpan M, Çiçek M, Tahra A, Akalin MK (Istanbul Medeniyet University Hospital); Izgiş A*, Soytas M, Okkabaz N, Saylar A, Onaran E, Askin AE, Hashimov E, Bektas S, Sabuncu K, Alagöz AC (Istanbul Medipol University Hospital); Karatas I*, Gok AFK, Simsek E, Altunay ZI, Koç E, Iscan Y, Sormaz IC (Istanbul School of Medicine); Yabaci BI*, Ertürk MS, Nazli M, Saglam IE, Erginöz E, Ozcelik MF, Gokden M, Guler C, Gunaydin FI, Uludağ SS, Ibis B, Bozkir HO, Karaca MB, Ulucay MC, Oner KB, Akbas S, Calikusu FZ, Akcan H, Ugural S, Zengin AK, Ozdemir Z (Istanbul University- Cerrahpasa, Cerrahpasa Faculty of Medicine); Demirbas I*, Guner A, Tepe MD, Kıralı N, Karabağ BC, Pehlivan D, Yuzgec AN, Yıldız B, Akin B, Aktaş M, Karabulut E, Cepe H, Küçükaslan H, Salih SS, Uzun M, Yücesan Ö, Reis ME, Ulusahin M, Aşcioğlu K, Tufan E (Karadeniz Technical University Farabi Hospital); Saleem Q*, Saracoglu K, Ozbilen M, Kale A, Geçici ME, Bulun Y (Kartal Dr. Lutfi Kirdar Training and Research Hospital); Uysal Ö*, Atay A, Şuataman B, Guducu C, Çinar TN, Kul H, Canpolat D, Ipekoğlu M, Aydemir M, Karahan F, Hizli K, Kaya B, Yenen OC, Gümüş B, Turmuş K, Vatansever S, Candan I (Katip Celebi University Faculty of Medicine Hospital); Sucu S*, Balik E, Bozkurt E, Özata IH, Ozoran E, Tüfekçi T, Karahan SN, Sarıkaya AF, Agcaoglu O, Danacı S (Koç University Medical School); Ulutaş ME*, Hasirci I, Arslan K, Metin ŞH, Yılmaz A, Turan E, Şimşek G, Kılınç A, Şahin ME, Ozden S, Bayraktar YA, Maden AS, Şahin A, Acar N, Erşen O, Balcı E, Yaldız H (Konya City Hospital); Guldogan CE*, Ozmen MM, Gundogdu E, Moran M (Liv Hospital Ankara); Celik B*, Sivrikoz E, Karabulut K, Dural AC (Liv Hospital Vadistanbul); Ikizoğlu T*, Aydede H, Yalçın T, Dalkıran A (Manisa Celal Bayar Üniversitesi Hafsa Sultan Hastanesi); Yapici HB*, Uprak TK, Tatar Y, Aksahin Y, Aral C, Cetin SZ, Artvin D, Tatar Z, Karakuş IS, Çatal S, Nalbantoğlu B, Bettar S, Düzyol Z, Demir BC, Kırcalı MF, Taşcı ME, Secil F (Marmara University School of Medicine); Akkaya EN*, Kayılıoğlu I, Hekmatjou B, Çağlayan A, Dadaşoğlu MA, Fergar C, Beden MS, Kayhan E, Arslan I, Koçyiğit GZ, Beyoğlu B (Mugla Training and Research Hospital); Varan C*, Sungurtekin U, Sungurtekin H, Özgen U (Pamukkale University School of Medicine); Çetin B*, Pergel A, Uyan M, Erata E, Aldhahebi E, Aslan B, Kabalı Ş, Köse F, Keşaplı I (Recep Tayyip Erdoğan Üniversitesi Eğitim ve Araştırma Hastanesi); Dulger UC*, Altintoprak F, Akçay M, Cünük ES, Kamburoğlu MB, Küçük IF, Yigit M, Sabuncu E, Harmantepe AT, Aka BU, Baş E, Yavuz Akça T (Sakarya Faculty Of Medicine); Sarı AC*, Colak E, Candan M, Kara ME, Ciftci AB, Yemez K, Uyanik MS, Polat S, Kucuk GO, Ocak S (Samsun Training and Research Hospital); Tosun Y*, Unal E, Ofluoğlu CB, Üstbaş C, Gangal Ö (Sehit Prof.Dr. Ilhan Varank Training and Research Hospital); Sanli AN*, Sayır S, Karslioglu C, Ulusoy I (Silivri State Hospital); Tufan AE*, Demir U, Celayir MF, Baran E, Aksoy AB, Fırat DB, Yüksel A, Güven O, Ertaş I, Kuzu ZS, Köksal HM, Gok M, Görgün MN, Yazici P, Ömeroğlu S, Özdemir BB, Kabul Gürbulak E (Sisli Hamidiye Etfal Training and Research Hospital); Ajredini M*, Cakcak IE, Atakan IH, Göztepe A, Budak O, Aydoğdu EO, Erdem E, Yılmaz C, Özdemir J, Akin B, Alkan D, Cengiz E, Donmez CN, Akyüz E, Ergun YH, Ozer MG, Ak Ç, Bolat F, Kasapoğlu E, Tatlisu A, Yorulmaz Y, Enez A, Ozkan B, Yilmaz ZE, Altin SN, Gunes B, Kaban B, Korkmaz O, Demir IB, Öz GA, Tuluce AB, Turkmen ZN, Yildiz F, Kandil B, Ulkucu A, Kıral G (Trakya Üniversitesi Hastanesi); Yavuz D*, Köksoy ÜC, Kus ZC, Erten MB, Mutlu M, Hökelekli F, Aslan T, Orçan Akbuz Ş, Durmuş E, Gökçek TE (Ufuk Üniversitesi Dr. Rıdvan Ege Hastanesi); Ulgur HS*, Ozkan OF, Karakullukcu HK, Yıldız A, Ceyhan M, Çelik MŞ, Kalın M, Kirkan EF, Demirbilek A, Çelik MN, Karbuş Ö (University of Health Science Umraniye Education and Research Hospital); Demirli Atici S*, Abud B, Erdinç H (University of Health Sciences Tepecik Training and Research Hospital); Ön KB*, Sevinç B, Damburacı N, Altiner E, Özdemir B, Manisalı Ö, Ural G, Topal MK, Can T, Ercan S, Kaynar S (Usak University Medical School); Emanet O*, Javadov M, Demir UB, Erdem A, Demircan F, Yelmenoğlu M, Seçen E, Alkışer B, Arık F, Kaya EE, Akkad D, Karaçam D, Karacam U, Yücel MA, Hatipoğlu A, Mumcu N, Karima O, Dal MB, Aljobbeh M, Montaser J (Yeditepe University Hospital); Kara MA*, Gultekin FA, Deniz O, Kavak N, Kum B, Ecir MM, Yavuzer YG, Sezgin Z, Koçdemir U, Yirmibeş B, Atalay Ş, May A, Varna S, Aydoğdu I (Zonguldak Bulent Ecevit University School of Medicine Research and Training Hospital).

**United Kingdom**: Abdel-Fattah A*, Ramsay G, Bin Badekruzaman MA, Karvelyte M, Siamisang M, Clunie S, Mikalauskaite M, Nessa A, Joshi C, Jodeh N, Zafar R, Miller J, Basharat R, Singh T, Weston O, Loy R, Tsoi EHC, Chandiramani A, Sumarlie RJ, Mitchell L, Yousif M, Qadir M, Gannon M, Tong EYH, Tsang J, Elmarghani M, Kaczmarek J, Luangboriboon J, Mcewen R, Tse B, Wong MC, Wong ZX, Diffley T, Jaruwattanapradid O, Ng N, Mchaffie L, Wright C, Joyce D, May V, Emmanuel S, D'costa A, Kumar A (Aberdeen Royal Infirmary); Hanganu C*, Gourgiotis S, Townson A, Williams CY, Baines E, Smith L, Elks N, Simon N, Chintapalli R, Banerjee A, Bhogle S, Ryan B, Maghsoudi D, Clifton E, Nott T, Vickers H, Stewart GD, Zhong ZX, Ioannou K, Ne CKH, Conci R, Fitzsimons-West E, Ling XL, Patel R, Sanghera R, Dokubo I, Seago I, Fairhurst O, Amin B, Aniq M, O’keeffe E, James S, Chowaniec M, Rutigliani L, Hu M, Ferreira AF, Kalogeropoulou M, Hui G, Coakham L, Healy S, Gilanliogullari K, Nishimura G (Addenbrooke's Hospital); Choi M*, Lunevicius R, Aje D (Aintree University Hospital); Mcnicholas R*, Newman C, Bollen E, Kumar A, Ferreira Xavier I, Baloch LG, Koduah-Sarpong NA, O'sullivan G, Saseendran S, Bin Sahl A, Murphy E, Hussain H, Nicholls A (Airedale General Hospital); O'dolan S*, Gidwani A, Patterson E, Loughrey L, Donaghy K, Hana K, Monaghan M, Ni Shandair S, Kulasingha N, Gormley N, Mcglinchey M, Dunlop E, Dunwoody B, Lian SH, Jayasangaran SC, Logue E, Goswami P (Altnagelvin Area Hospital); Ann Jacob P*, O'kane C, Ab Razak AS, Govender K, Mccann EJ, Perry S, Mckendry S, Pachchigar A (Antrim Area Hospital); Singh A*, Dindyal S, Pendyala S, Venkateswaran V, Alhoussan L, Feil F (Basildon University Hospital); Hughes T*, Gerçek Y, Choudry M, Haidar O, Rujeedawa T, Speirs T (Bedford Hospital); Odendaal J*, Chu J, Shahab A, Ranjithkumar S (Birmingham Women's Hospital); Pillai Rajendra Prasad V*, Linn T, Clark J (Blackpool Victoria Hospital); Sharma M*, Lockwood S, Chawla S, Deshpande R (Bradford Royal Infirmary); Long N*, Rees J, Kobetic M, Fawcett-Till D, Ferris O, Harrison G, Hibbs T, Tsang CTW, Hurley L, Jichi T, Badrinath C, Applebee L, Ecott K, Sullivan T, Piecha B, Jagadish A, Sajjad Z, Griggs R, Joyce S, Spurgeon R, Ali Khan M (Bristol Royal Infirmary); Hobrok M*, Roberts R, Mckenna J, Davies D (Bronglais Hospital); Eaton P*, Bishop L, Magaway S, Denholm K, Doyle S, Deshpande R, Salter M (Calderdale Royal Hospital); Chan V*, Mockford K, Heinz J, Ahmed I, Coverdale M (Castle Hill Hospital); McKillen RJ*, Maguire M, Mclees N, Lau J, Mcguigan L, O'Neill A, O'Hare D, Beggs C, Owolabi O, Hunter J (Causeway Hospital); Kinross J, Kotecha J, Doherty R, Patel V, Wagner E, Hodges L, Hassan H, Sribaskaran K, Pouris K, Jain S, Kim AJ, Gill S, Nand S, Toutouza O, Joshi A, Thornley L, Chen R, Ogunjimi M, Mahmood A, Chong R, Yanai M, Bae D, Dhaliwal J, Lovejoy A, Akhbari AR (Charing Cross Hospital); Mayor N*, Kontovounisios C, Yap SHK, Damarla M, Ooi B, Ng MA, Vargas Zhang A, Limbu S, Nyamakope K, Agarwala Y, Zingas N, Cleasby C, Smyth E, Drmota J, Yang QZC, Vedi C, Samarendra S, Nayak V, Rajendra S, Choudhry M, De Stadler K (Chelsea and Westminster Hospital); Bandyopadhyay S*, Bond-Smith G, Collart O, Baffour-Awuah K, Shah R, Mcnamara J, Tadikamalla G, Wilson B, Cossins C, Li H, Ismail L, Soleymani Majd H, Chang E, Jallow A, Baldelli S, Alberti P, Grant O, Doody M, Borawska Z, Bowman A, Clay H, Petit Y, Kimani JG, James M, Hussain SF, Nezhentsev A, Emmerson M, Vijjhalwar R, Shaw G, Holmes C, Ying Y, Farache Trajano L, Anis A, Dequaire J, Hunter A, Danvers R, El-Nemr M, Hammett C, Pikoula M, Thompson S, Lander B, Dierksmeier S, Sadeghi N, Suribhatla R, Ahmed R, Pandit S, Thornton W, Thornton-Swan T, Johal A, Khan M, More A, Tilahun Y, Hauperich A, Gidda R, Vorley I (Churchill Hospital); Khan Z*, Sintler M, Van Den Broeck A (City Hospital); Georgiou M*, Eardley N, Salem H, Yemparala S, Whittle L, Jagger C, Turner K, Vernon L, Aldred K, Manokar P, Moore A, Mistry N, Zgliczyńska J, Haylett C, Adeyanju A, Headford E, Khanna R, Deef M, Durkin T, Trayling E, Cowell R, Khan Z, Turner A, Noureldin K, Down W, Cyril A, O'halloran C (Countess of Chester Hospital); Shirke M*, Epanomeritakis M, Karnati A, Sreejith G, Sinton C, Oliver L, Ali Azamatullah Khan HF, O’kane E, Trouton J, French B, Campbell I, Curran R, Brines C, Mcdermott A (Craigavon Area Hospital); Yang N*, Vig S, Chowdhury S, Valecha R, Lau R, Nafis M, Thavanesan S, Marks J, Akaje-Macauley L, Patel N, Beard E, Nanda A, Mills C, Cheung A, Amalendran J (Croydon Hospital); Todd L*, Smolarek S, Warrener T, Sen S, Mudehwe A, Khan S, Wisden B, Lau G, Schanzer L, King Z, Giridhar K, Reed-Embleton M, White L, Filippidis P, Lee-Smith L, Carless O, King C, Herriott M, Saju N, Tin Maung YM, Deligianni A, Mumtaz A, Khan S, Hill H, Sahdev S, Thomas I, Shah A, Saji S, Hargreaves J, Khan R, Tharani A, Ullah R, Ludgate J (Derriford Hospital); Shrestha S*, Bota E, Abdullah A, Allison M, Patel Z, Clark A (Diana, Princess Of Wales Hospital); Suchett-Kaye I*, Stubbs B, Holmes B, Santaniello A, Watkinson T, Crimmins A, Gupta MK, Abraham S, Bellringer A, Clowes R, Chatzopoulou D, Kiran A, Eid J, Rao N (Dorset County Hospital, Dorchester); Caterson J*, Soggiu F, Malik H, Jose I, Kavallieros K, Rizk C, Thummala B, Li K, Goel A, Pantula R, Toh J, Fu MX, Ho CC, Vivek K (Ealing Hospital); Owen E*, Day A, Jamieson A, Hassani H, Thavarajah T, Vivekanandan M, Muneer A (East Surrey Hospital (Redhill)); Chauhan M*, Veeramootoo D, Ali S, Dosanjh J, Newing F, Jose E, Chauhan H, Kulshreshtha A (Frimley Park hospital); Thomas E*, Patel P, Chhabra R, Sajan B, Ragavan S, Hariharan R, Lam I, Fleet B, O'hara N, Wright A, Reddy V, Darweesh H, Khan A, Handa S, Kewada D, Rana F, Williams H, Bombieri F, Shah N, Pestotnik Stres D (Furness General Hospital); Menon A*, Selvam L, Pusok A, Street C, Zohdy M, Aguirrezabala Armbruster B, Okoye I, Potts L, Fayyad M, Bajaj K, Alfa H, Sivakumar N (George Eliot Hospital); Duncan N*, Roxburgh C, Huang L, Lukito K, Huang X, Baig S, Chan CY, Philip R, Shaheen L, Dodds J, Yeow HX, Devindran M, Sia ZC, Park JH, Furze B (Glasgow Royal Infirmary); Yung N*, Vipond M, Jones M, Asekun F, Dembinska-Kenner A, Saleh R, Larkai E, Harris J, Cherian M, Louden H, Bisbinas V, Behl A, Hughes R, Smith R, Zaman Z, Hoskyns O (Gloucestershire Royal Hospital); Carlton H*, Thorn C, Tourky M, Tan J, Sen K, Elsey J, Bevan J, Ko N, Kalidindi S (Great Western Hospital, Swindon); Rooney M*, El-Boghdadly K, Zafar S, Wyncoll A, Morillon P, Sennaraj M, Mahfouz H, Afzal A, Sibal R, Suji T, Headon H, Abdul A, Ahmed S, Mcnally-Reilly JP, Omran K, Mulcahy C, Aftab M, Haghighat Ghahfarokhi M, Farhangi M, To WY, Ho M, Patel T, Siu A (Guys and St.Thomas' Hospital); Choudhry H*, Huntley M, Hussain Z, Wong G, Maguire R, Khan M, Potts A, Ahmed M, Ramesh G, Kolade O (Harrogate District Hospital); Siddique MH*, Griffiths E, Qureshi M, Hoque M (Heartlands Hospital, Birmingham); Laulloo S*, Das K, Waheed N, Tang TYT, Ahmed R, Habib R (Hereford County Hospital); Vyas R*, Watson S, Theodoropoulou K, Siu M, Yu N, Islam N, Burnett J, Besso-Cowan A, Hirani D, Zsolnai I, Chen YK, Dupere O, Keitley L, Park C, Verma I, Chan J, Tavner J, Nicolaou M, Lee NYS, Hegy R, Lee RBY, Yi Lun KYL, Reyes D, Dong S, Thornton J (Homerton Hospital); Eaton P*, Bishop L, Magaway S, Denholm K, Doyle S, Deshpande R, Salter M, Weir M, Gibbs A (Huddersfield Royal Infirmary); Al-Shaye A*, Alwahid M, Tait A, Smith S, Doye A (Inverclyde Royal Hospital); Law KL*, Groot-Wassink TG, Geller G, Seebah K, Cox O, Kalogeropoulou M, Shetty A, Oh DCJ, Lee E, Packham B, Aarons M, Saadeh K, Mui J (Ipswich Hospital); Huynh R*, Eid M, Honey N, Kaur J, Hand R, Lai L, Koubaesh C, Maqsood-Shah H, Suresh J (James Cook University Hospital); Collart O*, Bond-Smith G, Shah R, Mcnamara J, Tadikamalla G, Wilson B, Cossins C, Li H, Soleymani Majd H, John L, Chang E, Kimani JG, Jallow A, Bandyopadhyay S, Baldelli S, Grant O, Doody M, Borawska Z, Bowman A, Foster C, Clay H, Petit Y, James M, Hussain SF, Nezhentsev A, Emmerson M, Vijjhalwar R, Shaw G, Holmes C, Ying Y, Farache Trajano L, Anis A, Dequaire J, Hunter A, Danvers R, El-Nemr M, Hammett C, Pikoula M, Thompson S, Shah R, Dierksmeier S, Sadeghi N, Lander B, Suribhatla R, Ahmed R, Pandit S, Thornton W, Thornton-Swan T, Johal A, Khan M, More A, Tilahun Y, Hauperich A, Gidda R, Vorley I, Lewis-Orr E (John Radcliffe Hospital); Reilly S*, Bhatia A, Goves K, Blesson M, Purser L, Cobb L, Sarker MH, De Sousa N (Kettering General Hospital); Syed S*, Rajendran N, Tan Sue Wei J, Navakumar G, Butnari V, Senthilkumar M, Chew ZQ, Zekaite L, Paranietharan S, Haque M, Balenthiran S, Chin KJ, Ahuja S, Moothathamby T, Moniati F, Kong YZ, Lee EJH (King George Hospital); Sharma L*, Found P, Sivakumaar Y, Senior H, Simeen N, Arora A, Chu A, Mizori R, Beazer T, Patel N, Dudziak JM, Squeri T, Weston F, Zhou M, Van Ross J, Tian S, Bedford J, Lam R, Karami Tireh Shabankareh F, Donkin Everton H, Wilson K (King's College Hospital); Richens A*, Bragg D, Akbari A, Awodiya A, Osula SA (King's Mill Hospital); Gerges F*, Gerogiannis I, Daniels L, Seth S, Baxter Z, Nour E, Fitchford L, Perrott C, Abuelgasim M, Flanagan J, Gillani S, Lewis H, Dunscombe O, Steven Goodwin Moughton P, Mahmood A, Hirniak J, Moses A, Kapsampelis P (Kingston Hospital); Ahmed A*, Burke D, Anderton C, Lee E, Conley M, Khan L, Surti R, Waseem F, Sharma M, Imran A (Leeds General Infirmary); Motiwale R*, Singh B, D Sa S, Charuvila S, Lorgat MZ, Chowdhury R, Gogineni P, Finney A, Tzortzi De Paz A, Panesar S, Sidki A, Warwick C, Kadambande S, Patel J, Rao A, Sharma A, Ramewal S (Leicester General Hospital); Ramesh P*, Norwood M, Mehta P, Bhatia M, Tiwana S, Foote E, Rushton F, Asi S, Notaney R, Sinha R (Leicester Royal Infirmary); Vibhishanan J*, Gupta S, Conci R, Chintapalli R (Lister Hospital); Ahmed S*, Sagar J, Ragge N, Rizvi SS, Ashraf M, Cirocchi N, West R, Obiri-Darko E, Rai Y, Hussain S, Ul Ain N (Luton & Dunstable University Hospital); Amir FN*, Smart C, Melomud R, Saad D, Bengeri S, Kuna S, Chaudhary M, Olanite OG, Khan F, Nausheen S, Dodds R (Macclesfield District General Hospital); Boyden H*, Donnelly D, Adegboye O, Osborne L, El-Barraj S, Shukla J, Raza N, Khalid A, Agrawal D, Kyaw KT, Borhan S, Pangrazi G, Ioannou I, Ahmed AN, Savvides G, White M, Puthur SJ (Manchester Royal Infirmary); Bridgewater S*, Waraich N, Bryce R, Khosla R, Penhaligan L, Desai A, Ehsan Z, Mostafa O (Manor Hospital, Walsall); Kamel Y*, Keeler B, Rajivan R, Omar V, Gamadia S, Rana D, Srikumar S, Heidari A, Ahsan Akhtar A (Milton Keynes University Hospital); Tarafdar A*, Barry J, Davies D, Curr A, Jimenez-Reid I, Mckenna J, Garry E, Lallmahomed N, O'leary K, Barker E, Jones DR, Tabatabai R, Garg J, Henshaw E, Abdulshafea M, Prabaharan S (Morriston Hospital); Wiles H*, Bamford R, Blest F, Acquah L, Simpson H, Guerrero Enriquez J, Pringle H, Shahid M, Wharf O, Kumar M, Buadooh K, Hanson M, Mutanga I, Hardy A, Usman H, Shams S (Musgrove Park Hospital, Taunton); Schottler N*, Garg A, Sarodaya V, Ali S, Cullen I, Pregil L, Fernandes Silva Ramos M, Hao Y, Das Chagas E Silva AC, Sellahewa S, Li J, Cox J, Sinha P, Lee IJJ, Voniati E, Phillips H, Mooney O, Sockett M (Newham University Hospital); Patil P, Elsllabi M, Chan WKM, Grimble L, Richardson H, Johnston A, Kouli T, De Sousa JFM, Goh YK, Grant C, Martin L, Peh ZH, Yap T, Tang ETW, Lai HYM, Berry T, Rokia M, Tennant E, Lee I, Rahman MJ, Kamal A, Ali S, Hau HX, Harten I, Eloofy M, Caldwell C, Leckie R, Tasnim F, Ravintharan K, Mcauley J, Cameron F, Veerni RB, Looi KY, Ismaili A, Gresz R, Chong V, Tan LK, Singh K, Ashburn S, Shantha Kumar R, Kamal M, Kamal H (Ninewells Hospital); Aziz I*, Stather P, Mylvaganam L, Deliyannis E, Tompkins B, Sikorski E, Harris F, Sanders I (Norfolk and Norwich University Hospital); Fakhrul-Aldeen M*, Cross K, Tahir A, Yim P, Mayne I, Mahapatra S (North Devon District Hospital); Ng CE*, Ortega A, Supparamaniam S, Bowman M, Sadler M, Rogger M (North Durham University Hospital); Gendia A*, Ahmed J, Sivakumaran P, Brick C, Ansong E, Ha H, Sharma A, Wilson N, Manavi B, Zeze L, Gupta NP, Al-Hassani S, Williams C, Ramesh M, Shukla B (Northampton General Hospital); Drye E, Hussain H, Ghatauray E, Tan ACW, Adamu-Biu F, Arora J, Mcallister T, Fairclough S, Economou A, Utulu S, Fung KW, Epanomeritakis IE, Zaman SS, Olszewski E, Ballesteros P, Okpii EC (Peterborough City Hospital); Walkeden E*, Macklin C, Jamil S, Zhen S, Ahmed O, Saravana Kumar A, Uddin H, Hakim R, Mossanen Parsi M, Awoloto O, Rowe K, Mashadi R, Mohammed Z, Chong LC, Fairlie-Vogt S, Russell O, Ozarek W, Little A, O'farrell C, Miller R (Pinderfields Hospital); Froud J*, Thippeswamy K, Thomas R, Fitzpatrick A, Yoganathan S, Dunlop M, Ngai K, Liddell-Lowe T, Barrow A (Prince Charles Hospital, Merthyr); Sharma L*, Found P, Mizori R, Rabas S, Al-Housni R, Zhou M (Princess Royal University Hospital); Marton A*, Chen P, Hasan H, Chan CFB, Win Mar WW (Queen Elizabeth Hospital King's Lynn); Kisiel A*, Griffiths E, Yusuf M, Sinha A, Blenkinsop J, Rawlings K, Chaudri T, Westland G, Umar H, Lee S, Cherian J, Gurhan Z, Chaudri T, Sheraz MS (Queen Elizabeth Hospital, Birmingham); Khan DMI*, Kucukmetin A, Malone D, Betts C, Ali M, Giblin M, Nowicki L, Korompelis P, Farley O, Fouweather M, Ioannides C, Yilmaz H, Landais R, Ngeyu EX, Erinjeri C (Queen Elizabeth Hospital, Gateshead); Badran D*, Glen P, Aamir J, Aziz A, Su PL, Choong I, Reece M, Kulasekaran A, Tengku Saifudin TNS, Luckhurst J, O'hare R, Smith R, Siu YT, Kwok PH, Sood V, Imran A, Fatima K, Munir Z (Queen Elizabeth University Hospital, Glasgow); Kisova T*, Rajendran N, Islam F, Teehan E, Sadik H, Patterson U, Rahman N, Harrisrhaj H, Govardhan D, Rajesh S, Mumtaz Z, Ghori OS, Nawaz H, Lysomirski A, Pikoula V, Qadir RMAB, Jones A, Chiang Y, Morsy H, Pazaiti C, Asardag AN, Ali I, Althonayan J, Badri A, Evans J, Chung E, Harriss L (Queens Hospital); Sinan L*, Maxwell-Armstrong C, De A, Altman R, Varghese F, Chua W, Ahmed S, Javed M, Aljubure R (Queens Medical Centre, Nottingham); Bradley A*, Moug S, Kemmett OEH, Underwood I, Fitzpatrick I, Wong LS, Vakeesan B, Potter K (Royal Alexandra Hospital, Paisley); Varghese T*, Daren S, Hazrin Fazail A, Farajzadeh Asl S, Nautiyal H, Saadeh A, Vial I, Karimi R, Akthar A, Egiz A, Masood R, Qadri A, Chawla T, Yoon H (Royal Blackburn Teaching Hospital); Liu X*, Battersby N, Davies E, Chhetri R, Mclean R, Leaper E, Taylor C, Nicolaisen C, Cochetti L, Zoumi EA, Annable D, Thomas H, James-Knights Z, Lee-Kim-Koon C, Akpinar E, Coelho MJ, Darke E, Morris T, Mayer F, Forbes J, Gold S, Sinha H, Batchelor E (Royal Cornwall Hospital); White L*, Lund J, Forrest S, Dial H, Chishty F, Oliver M, Meyer J, Gokhare Viswanath N, Ammar A, Lopez A, Minhas N (Royal Derby Hospital); Sunny N*, Haynes K, Alexiadis A, Doski S, Arbuthnott R, Memon R, Bukhashem K, Chaudhary A, Sheik-Ali A, Sebastiao A, Taylor C, Clarke E, Misztal S, Raja D, Chui SK, Khalil I, Metsel A, Whelan M, Forrester S, Oboh O, Bayley-Skinner B, Eastwood R, Akapo E (Royal Devon and Exeter Hospital); Jamshaid M*, Osborn G, Woods R, Wooler O, Chumley S, Cotton E, Jarrett N, Amanda N, Johnson J, Quhill M, Al-Ani M, Kandanearachchi S, Khan S, Barrow A, Dunlop M (Royal Glamorgan Hospital); Williams G, Phillips S, Faryad F, Hughes A (Royal Gwent Hospital, Newport); Wong Ching Hwai S*, Johnston C, Guest R, Kaddouri A, Ernst L, Szasz A, Gafrey C, Loy G, Roberts E, Baker S, Davies A, Khan Z, Leonidou M, Chow JK, Murugan R, Baker A, Burns R, Foo YC, Sikora F, Chong L, Chan Ah Song C, Cosgrove C, Reeve-Chen O, Ma J, Mccolm L, Zhang MK, Hussain F, Ng XY, Ingabire B, Wagner B, Bain T, Kovacs R, Small E, Lam S, Yao L, Ho L, G Paremes Sivam H, Dahal K, Stanley A, White M, Hayois L, Thillai M (Royal Infirmary of Edinburgh); Tay T*, Davies E, Kamaruzaman N, Robinson L, Sherrington R, Abid Sohail Z, Lawrence S, Sohail A, Brown D, Parker S, Ohr M, Jasniak D, Crowther B, Jadoon F, Melo LJ, Amin H (Royal Lancaster Infirmary); Lim RQH*, Thaha MA, Bath M, Saadqain SA, Benallal Y, Jin Z, Jackson A, Kuri A, Malik M, Jos H, Goh ET, Paraskevopoulou ME, Kythreotis A, Naim Ahmed Y, Glynou SP, Rehan H, Pereira Pai A, Georgiannakis A, Dworschak N, Cho S, Chow BJ, Khan Y, Enthoven T, Malik A, Latif I, Lakhani AH, Rahman N, Morgan K, Gkolia S, Khalessi N, May PLZ, Lwin A, Kanesaratnam P, Tudgee M, English R, Hu K, Pathanchaly N, Chien DCS, Holloway M, Gao K, Lekka G, Srinivasan R, Zafar S (Royal London Hospital); Peris MR*, Keiarash K, Kanesan H, Chong B, Kobus M, Dominic D, Do DA, Dennis D, Frankland S (Royal Preston Hospital); Shah V*, Youssef B, Sadia U, Hassouneh A, Jaipersad A, Chen T, Fulford M, Dawson A, Kapur K, Jha T, Zope P, Wilkinson M, Bhatnagar V, Knight J (Royal Stoke University Hospital); Madhuri TK*, Katsiari T, Pasternak-Albert N, Moussa E (Royal Surrey Hospital, Guildford); Emms L*, Lamah M, Batool Z, Sabesan A, Islam S, Baig M, Dave M, Liu X, Stuart L, Harvey S, Dam V, Ezuma P, Lee J, Suntharalingam S, Singh S, Preston P, Rickard F, Gupta T (Royal Sussex County Hospital); Madden S*, Jones C, Gidwani R, Wang X, Mcconnell M, Hanna S, Mcgettigan A, Brown K, Cios A, Ward-Bradley C, Hurrell O, Paice V, Mutsonziwa N, Pogoson S, Coulter R, Corry J, Mcclean L, Keenan F, O'dolan S (Royal Victoria Hospital, Belfast); Plonkowski A*, Brady R (Royal Victoria Infirmary, Newcastle); Ooi R*, Iles D, Guillemot LS, Low C (Saint Marys Hospital); El-Barraj S*, Epstein J, Pressler M, Ioannou I, Soliman N, Ktayen A, Macconnachie A, Menendez Lorenzo J, Tooke J, Noman K, Wilkins L, Warner-Levy JJ (Salford Royal Infirmary); Evans R*, Torrance A, Fear C, Vidis Humphries H, Velayudham G, Jefferson-Pillai Z, Brady J, Oza R, Singh D, Hamid A, Hartop P, Shojaie P, Mano J, Rajah T, Cabdi YMC, Singal A, Afzali M (Sandwell General Hospital); Lee JM*, Bandyopadhyay D, Bennett J, Mendenhall P, Millett A (Scarborough General Hospital); Davidson S*, Kallam R, Pishia M, Butler H, Isidore S, Boutsias D, Bryce K (Scunthorpe General Hospital); De Rancourt T*, Barry J, Henry H, Aspinall A, Smith L, Conway C, Shanmugathas H, Huang C, Doherty M, Ahmed K, Chung T, Benson T, Habiba A, Singh Bhangu J, Bingor B, Fice L, Premanandhan H, Mckenna J, Jimenez-Reid I (Singleton Hospital); Hoque M*, Griffiths E, Loughran J, Laskowski M, Mehmood A (Solihull Hospital); O'kane R*, Mullan M, Mcclenaghan R, Kettyle G, Hosty-Blaney F, Chan XHF, Sunny S, Kaur M, Vose S, George V, Killoran D, Doherty R (South West Acute Hospital); Zagorac Z*, Pullyblank A, Ismail R, Anderton N, Dwyer-Joyce L, Morgan R, Zhang N, French B, Cox H, Tetro J, Clarke A, Shaw K, Iau R, King E, Patel S, Ovakimian M, Zahid A, Rowley G, Tanner R, Davoudi K, Shear B, Xu M, Karan K, O’reilly A, Ahmed S, Iskrenova Kirilova T, Ezekiel A, Shapland M, Mitra M, Krum T, Higginbotham G, Winayak R, Mustow J, Yorke L, Gibb C, Ilyas A, Pound M (Southmead Hospital, Bristol); Abouharb A*, Burke D, Khan A, Aimar K, Bennett J, Kerman-Fiore E, Rao S, Dayal A, Chawla S, Wadey L, Smith C, Desor K, Surti R, Gul Z, Gourgiotis V, Kaselampao LM, Nguik C, Agrawal R, Liaw QY, Mckeown E, Hassan A, Krishnan K, Glover HO, Devassy M, Ogunyanwo D, Dickenson G, Elder G, Valdez G, White B, Hamilton I, Shah S, Sinha S, Punwani K, Peat I, Hayat M, Khalid N, Frankel E, Macgowan H, Braka A, Byfield A, Brooks J, Kashif A, Archer R, Akkaya S, Sood M, Akther S, Birkett O, Kadri T, Abdulmula S, Psefteli P (St James University Hospital, Leeds); Chadha S*, Reese G, Mark C, Asunramu M, Hess T, Mehta S, Tsang C, Syed K, Alocious A, Steinruecke M (St Mary's Hospital IOW); Ashley M*, Newton R, Wainwright J, Ayathamattam J, Wong H (St Richards Hospital (Chichester)); Elahi Z*, Fleming M, Ali T, Lloyd D, Brooks K, Kwon C, Mckerrell A, Jamadar T, Barcelona MD, Polluk D, Kwon J, Henry F, Ayirookuzhi S, Mwipatayi M, Cheruiyot M, Adam S, Cruickshank T, Gollub K, Revell M, Taiwo S, Atchade A, Harewood A (Stoke Mandeville Hospital); Niina A*, Bosanquet D, Mckeon-Williams O, Szakmany T, Badhams E, Christensen N, Sammut J (THE GRANGE UNIVERSITY HOSPITAL); Tay N*, Pollok JM, Raptis D, Varcada M, Ganesananthan S, Chandrasekar R, Deshpande A, Ghoora LP, Hogg G, Staubli S, Banerjee M, Sunil Menon S, Sanz Pena A, Rana R, Mohamed A, Crabtree P, Flower A, Banerjee A, Talabi Y, Bishop N, Kupfuwa N, Ilangovan D, Qureshi H (The Royal Free Hospital); Minty I*, Baron R, Greasley L, Birkenhead P, Baron R, Shahid M, Rajasivam N, Chu WL, Punnoose N, Sundaresan V, El-Galley M, Prasansapakit K, Mitra S, Garlick M, Prakash M (The Royal Liverpool University Hospital); Gao H*, Parmar C, Lee F, Aich A, Ismail A, Kirupaharan M, Khan S, Patel K, Mclaverty D, Hammerton G, Walford H, Roberts H, Cautivo P (The Whittington Hospital); Abdel Kader H*, Kimble A, Manaf K, Mackonochie R, Mitchell V, Ghaznavi S, Taheem A, Firth E, Chandler H, Eldessouki K, Fyfe J, Bhat M, Cavlin J, Pearce E, Patterson S, Johnson C, El-Badawi K, Gariballa M, Hale E, Younis H, Brackenbury E, Chappell A, Poulton T, Dawson E (Torbay Hospital); Murray A*, Mcallister I, Hassan A, Cunningham B, Chan-A-Sue T, Rajpal N, Keane P, Walsh K, Mcgeoghan L, Neeson M, Brown H, Adams SM, Ladha S, Walsh A (Ulster Hospital); Alradhawi M*, Tarighi R, Miah MI, Dawas K, Mojadady A, Priestman-Degano A, Vellore Sasikumar SK, Suresh B, Fornasiero M, Csvila D, Kumar A, Adil ST, Adil HS, Kataria U, Jaibaji R, Patel N, Goch A, Quaye P, Gupte A, Mustafa H, Otti V (University College Hospital); Dewantoro D*, Ali M, Sood M, Wright H (University Hospital Ayr); Abusheba L*, Wong L, Docksey M, Abdinasir A, Karim MO, Kolaityte V, Vasileiadou Pelling M, Abdel Basit A, Pearson E, Hughes E, Millington AS (University Hospital Coventry and Warwickshire); Taylor L*, Borg C, Jagic K, Waheed A, Singh K, James V, Chowdhury T, Saxena A, Georgiou A (University Hospital Lewisham); Jones T*, Carpenter C, Hughes I, Aloul Z, Hanna-Davies C, Jacob A, Puthiyakunnel Saji A, Qureshi A, Ulfin Z, Dunlop M, Woods R, Hill N, Mohamed N, Vora A, Asharaf H, Maye E, Arif U, Srinivasan S, Prasad S, John B, Shivanand V, Singh A, Zahid A, Stewart E, Raketla M, Kokoricha O, Karwa D, Crisp J, Shehadeh R, Lee J, Webb B, Lepping S, Yusuf F, Pak E, Soo B, Pemmasani P, Goyal S (University Hospital of Wales); Binti Azad Bashir AB*, Komolafe S, Naeem A, Anbananthan HR, Au Yong LK (University Hospital Wishaw); Mookerjee S*, Ward N, Aniq M, Davis J, Al-Sukaini A, Anthony J (West Suffolk Hospital); Boyle J*, Laird A, Speake D, Beagan C, Stewart S, El-Koubani O, Chan R, Viswanath M, Janssens S, Shah I, Nguyen V, Mckee E, Ng CW, Balać M, Suresh Kumar DI, Seeva Balan S, Kirk R, Miles B, Kovacic LA, Collins VG, Low J, Sim W, Chua SJ, Narayanan N, Lee TY, Lo CMN (Western General Hospital, Edinburgh); Chauhan M*, Elkafsi J, Rasul S, Yammine R, Sattar Z, Dixit D (Wexham Park hospital); Rahman N*, Davies J, Dindyal S, Osborne L, Nip L, Wilding de Miranda N, Stevens A, Al-Diri B, Lim A, Kallikas G, Kim Y, Anjum M, Jeyapahan P, Hakim M, Singh S, Jamileh E, Sohrabi C, Baek SH, Sadik N, Mohammed A (Whipps Cross Hospital); Aje D*, Clifford R, O'halloran C, Mahajan K, Darke N (Whiston Hospital); Lloyd S*, Mlotshwa M, Melhuish K, Derex-Briggs J, Bell M, Varma P, Fray K, Garg Y (Worthing); Datta K, Mantel-Cooper T, Goodfellow M, Kazi B (Wrexham Maelor Hospital); Sievers N*, Telford K, Almansoor ZR, Craddock K, Tan JL, St John L, Singhania A, Dosani S, Mughal S, Bokhari N, Brooks L (Wythenshawe Hospital); Laid I*, Lala A, Ong CS, Wakefield S, Phillips S, Unwin H (Ysbyty Gwynedd, Bangor).
